# Synthesis of Enantiostructured Triacylglycerol Prodrugs Constituting an Active Drug Located at Terminal *sn*-1 and *sn*-3 Positions of the Glycerol Backbone

**DOI:** 10.3390/molecules30050991

**Published:** 2025-02-21

**Authors:** Lena Rós Jónsdottir, Gudmundur G. Haraldsson

**Affiliations:** Science Institute, Chemistry Department, University of Iceland, Dunhaga 3, 107 Reykjavik, Iceland; lrj@hi.is

**Keywords:** asymmetric synthesis, enantiostructured triacylglycerols, lipase, n-3 PUFAs, acylglycerol prodrugs, (*S*)-ibuprofen, (*S*)-naproxen

## Abstract

The current paper reports the asymmetric synthesis of a focused library of enantiostructured triacylglycerols (TAGs) constituting a potent drug of the NSAID type (ibuprofen or naproxen) along with a pure bioactive n-3 polyunsaturated fatty acid (PUFA) intended as a novel type of prodrug. In this second category, a TAG prodrug of the terminal *sn*-1 or *sn*-3 position of the glycerol skeleton is acylated with a single saturated medium-chain fatty acid (C6, C8, C10, or C12), and another with the drug entity; the PUFA (EPA or DHA) is located in the *sn*-2 position. This was accomplished by a six-step chemoenzymatic approach, two of which were promoted by a lipase, starting from enantiopure (*R*)- and (*S*)-solketals. The highly regioselective immobilized *Candida antarctica* lipase (CAL-B) played a crucial role in the regiocontrol of the synthesis. The most challenging key step involved the incorporation of the drugs that were activated as oxime esters by the lipase exclusively in the terminal position of glycerol that is protected as a benzyl ether. All combinations, a total of 32 such prodrug TAGs, were prepared, isolated, and fully characterized, along with 24 acylglycerol intermediates, obtained in very-high-to-excellent yields in the majority of cases.

## 1. Introduction

**Figure 1:**
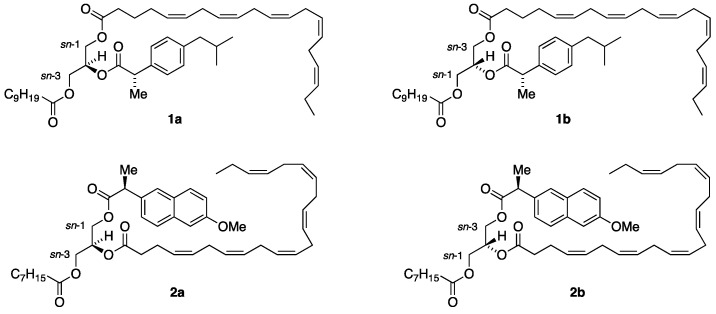
The structure of TAG prodrug diastereomers **1a** and **1b** belongs to the first category prodrugs, and TAG prodrug diastereomers **2a** and **2b** belong to the second category prodrugs.

In a very recent report, we proposed a novel type of lipid-based prodrugs possessing an active drug, a bioactive long-chain n-3 polyunsaturated fatty acid (PUFA), and a saturated fatty acid, all attached to predetermined regio- and stereospecific positions of the glycerol backbone of triacylglycerol (TAG) molecular species (see Figure 1) [1]. Prodrug is a term used for a compound that delivers an active drug after undergoing a bioconversion (intra- or extracellular) within the body. The design of a prodrug aims to improve the bioavailability of a drug by exerting influence on its absorption, distribution, metabolism, and excretion (ADME) [2,3,4]. Prodrugs based on lipids offer advantages, such as increased absorption through the intestines, which may result in increased drug availability and targeting [5,6,7,8].

As described in detail in the report, the design of the TAG prodrugs is based on several important concepts and features associated with fatty acids and TAGs. The first is structured MLM (medium–long–medium)-type TAGs possessing a medium-chain fatty acid (MCFA) located at the terminal positions of the glycerol backbone with a bioactive n-3 PUFA (EPA or DHA) located at the 2-position. Such structured TAGs have gained a growing interest among scientists because of their absorption properties in the digestive tract and nutritional values [9,10,11]. Here, we benefitted from our previous synthesis of such MLM-type TAGs by a two-step chemoenzymatic synthesis, starting from glycerol, with the aid of a highly regioselective immobilized *Candida antarctica* lipase (CAL-B) that introduced the MCFAs exclusively onto the terminal primary alcohol positions of the glycerol [12,13].

The second one is the bioactive n-3 PUFA’s EPA and DHA, which are claimed to offer numerous beneficial effects on human health, including cardiovascular disease, cognitive health, inflammatory diseases, and so forth [14,15,16,17]. EPA and DHA are also precursors to various highly potent lipid mediators, such as the specialized pro-resolving mediators (SPMs) that display potent anti-inflammatory and pro-resolving activities and include resolvins, protectins, and maresins [18,19,20]. As precursors to the SPMs, EPA and DHA may be regarded as anti-inflammatory prodrugs [21]. Furthermore, EPA and DHA as ethyl esters are also available as prescription drugs to treat hypertriglyceridemia [22], both as a mixture [23,24] and a pure EPA [25,26,27].

The third concept behind the prodrug design is termed enantiostructured TAGs [1,28,29,30]. They are based on glycerol being prochiral and the consequent chirality of TAGs possessing selected fatty acyl groups that occupy predetermined stereospecific positions [1,31,32] of their glycerol skeleton. Their involvement is predicated on our belief that the location of the active drug or the bioactive n-3 PUFAs, not only at the regiospecific (terminal versus mid positions) but also stereospecific positions (*sn*-1, *sn*-2, *sn*-3) [1,31,32] within the TAGs, may influence the timing and site-specificity of their delivery from the proposed TAG prodrugs in or after the digestive tract, as has been described in detail [1].

Finally, it was thought appropriate to base the demonstration of the idea of enantiostructured TAG prodrugs on the use of the non-stereoidal anti-inflammatory drugs (NSAIDs) (*S*)-ibuprofen and (*S*)-naproxen, to which EPA and DHA may offer some synergistic effects as precursors to the anti-inflammatory SPMs [1]. To our knowledge, there are no reports on prodrug design that is based on acylglycerols constituting active drugs along with the n-3 PUFAs. The advantage offered by the presence of the MCFAs has also been addressed [1].

This resulted in constructing two proposed enantiostructured TAG prodrug regioisomeric forms, as depicted in Figure 1. The first form is represented by structures **1a** and **1b**, where the active drug (*S*)-ibuprofen is placed in the *sn*-2 position. In structure **1a**, the n-3 PUFA EPA is located at the *sn*-1 position, along with capric acid (C10:0) in the *sn*-3 position. In structure **1b**, the positions of EPA and DHA have been interconverted. Consequently, structures **1a** and **1b** are diastereomers. The previous report [1] described the synthesis of TAG prodrugs that belong to this first category of TAG prodrugs, with all combinations of the two drugs (*S*)-ibuprofen and (*S*)-naproxen, EPA and DHA, and the saturated fatty acids ranging from C6:0 to C16:0; there is a total of 48 such TAG prodrug molecular species (24 diastereomer pairs).

The current paper describes the corresponding asymmetric synthesis of the second category of TAG prodrugs to which the diastereomeric structures **2a** and **2b** belong. In this category, the drugs are placed in the terminal positions with the PUFAs in the *sn*-2 position. As noticed from Figure 1, (*S*)-naproxen represents the drugs, DHA represents the n-3 PUFAs, and caprylic acid (C8:0) represents the MCFA in structures **2a** and **2b**, where the acyl groups in the terminal positions have been swapped. We decided to limit the task to MCFAs only; that is, caproic, caprylic, capric, and lauric acids (C6:0, C8:0, C10:0, and C12:0, respectively). All combinations of such TAG prodrug molecular species were prepared, with a total of 32 TAG products (16 such diastereomeric pairs). This has resulted in our establishment of a large, focused library of enantiostructured TAG prodrugs that may soon be screened for new and interesting properties to increase drug bioavailability and targeting.

## 2. Results and Discussion

**Figure 2:**
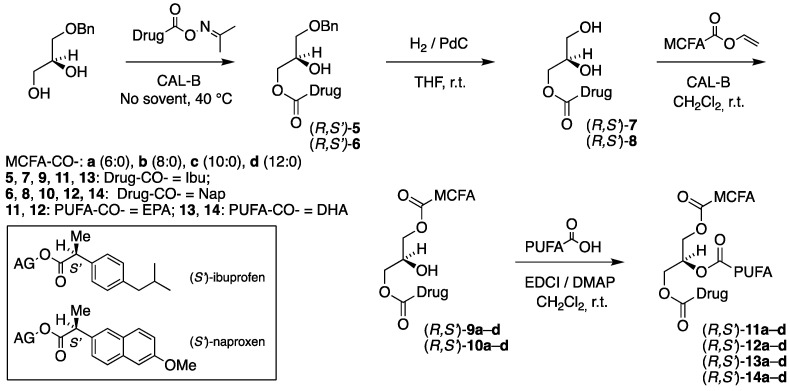
Chemoenzymatic synthesis of the second category TAG prodrug diastereomer series (*R*,*S*′)-**11a**–**d**–**14a**–**d**, starting from 1-*O*-benzyl-*sn*-glycerol. In the scheme MCFA-CO-, PUFA-CO- and Drug-CO- refer to the corresponding saturated medium-chain fatty acyl, polyunsaturated fatty acyl and drug acyl group substituents, respectively. In box: (*S*′)-ibuprofen and (*S*′)-naproxen attached as esters to acylglycerols (AG). The obtained yields of all individual intermediates and products are revealed in Table 1, Table 2, Table 3, Table 4, Table 5, Table 6, Table 7 and Table 8.

A four-step chemoenzymatic approach was designed for the synthesis of the second category of TAG prodrugs, which is depicted in Figure 2, and involves two enzymatic steps. As before, it is based on the use of 1-*O*-benzyl-*sn*-glycerol (prepared in two steps from (*R*)-solketal [1]) as a chiral precursor, with the *sn*-1 position protected as a benzyl ether. The first step involves a lipase-promoted regioselective acylation of the *sn*-3 hydroxyl group of the diol, with the drug activated as an acetoxime ester. After the removal of the benzyl protective group in the second step, the MCFA is introduced into the *sn*-1 position by using lipase. The final step involves the incorporation of the n-3 PUFA into the *sn*-2 position of the glycerol backbone, brought about by a chemical coupling agent to complete the synthesis.

As can be noticed in Figure 2, the synthesis covers all combinations of the (*S*)-ibuprofen (Ibu) and (*S*)-naproxen (Nap) placed in the *sn*-3 position, with EPA and DHA in the *sn*-2 position and the four saturated medium-chain fatty acids, caproic, caprylic, capric, and lauric acids (C6:0, C8:0, C10:0 and C12:0, respectively), located in the remaining *sn*-1 terminal position of the glycerol backbone. This results in a focused library of a total of 16 targeted enantiostructured TAG prodrugs (*R*,*S*′)-**11a**–**d**–**14a**–**d** and also involving a total of 12 enantiopure acylglycerol intermediates.

**Figure 3:**
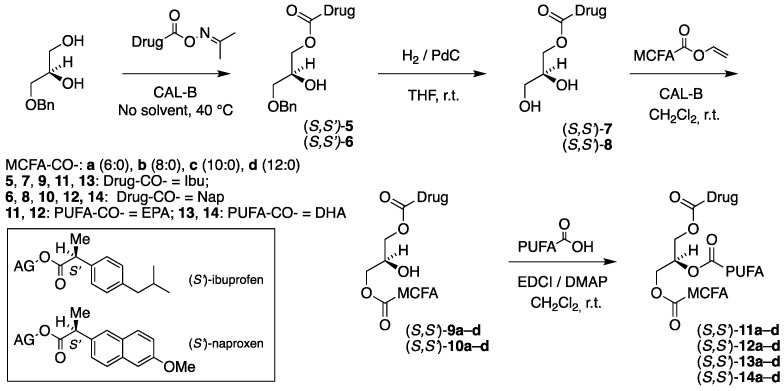
Chemoenzymatic synthesis of the first category TAG prodrug diastereomer series (*S*,*S*′)-**11a**–**d–14a**–**d**, starting from 3-*O*-benzyl-*sn*-glycerol. In the scheme SFA-CO-, PUFA-CO- and Drug-CO- refer to the corresponding saturated medium-chain fatty acyl, polyunsaturated fatty acyl and drug acyl group substituents, respectively. In box: (*S*′)-ibuprofen and (*S*′)-naproxen attached as esters to acylglycerols (AG). The obtained yields of all individual intermediates and products are revealed in Table 1, Table 2, Table 3, Table 4, Table 5, Table 6, Table 7 and Table 8.

The synthesis of the corresponding TAG prodrug diastereomers (*S*,*S*′)-**11a**–**d**–**14a**–**d**, where the enantiospecific location of the MCFAs and the drugs has been interchanged with the PUFAs still located at the *sn*-2 position, was of equal interest. Their synthetic route illustrated in Figure 3 is identical to the above one shown in Figure 2, this time starting from 3-*O*-benzyl-*sn*-gycerol (prepared in two steps from (*S*)-solketal [29]) as a chiral precursor.

### 2.1. The Enzymatic Coupling of the Drugs

The first step involved enzymatic coupling of the drugs activated as acetoxime esters exclusively to the terminal position of the benzyl-protected glycerols. This was the main challenge to overcome in the second category of TAG prodrug synthesis. Acetoxime esters have previously been used to activate esters [33,34,35], including the n-3 PUFAs EPA and DHA [36], to ensure faster reactions in biotransformations involving lipase. Faster reactions, along with the mildness offered by lipase, are the key parameters in controlling the regioselectivity of the lipase and unwanted acyl migration [1,37], which is detrimental to the regioisomeric outcome of the reactions.

**Figure 4:**
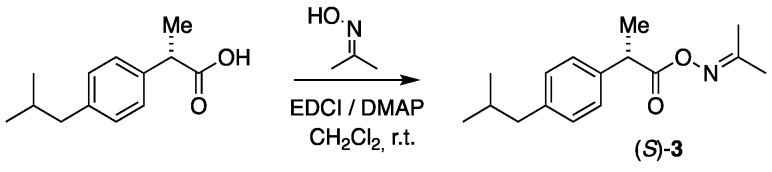
Preparation of an activated acetoxime ester (*S*)-**3** of ibuprofen by chemical coupling of acetoxime to (*S*)-ibuprofen.

The acetoxime esters were prepared through the chemical coupling of acetoxime to the drugs by using 1-ethyl-3-(3-dimethylaminopropyl)carbodiimide (EDCI) in the presence of 4-dimethylaminopyridine (DMAP) in dichloromethane at r.t. by a previously reported protocol [36]. The reaction for (S)-ibuprofen is shown in Figure 4. The acetoxime ester of (*S*)-ibuprofen, (*S*)-**3**, was obtained as a liquid in a 96% yield, whereas the corresponding ester of (*S*)-naproxen (*S*)-**4** was obtained as a white solid in a quantitative yield. Both derivatives underwent noteworthy changes in their specific optical activity values: (*S*)-ibuprofen from +58.2 to −7.18 for its oxime ester and (*S*)-naproxen from +78.3 to −12.3 for its corresponding ester.

Acetoxime esters have been successfully used to activate EPA and DHA [36] to accomplish excellent regioselectivity in their lipase-promoted reactions involving the CAL-B with glycerol- and 1-*O*-acylglycerol-type ether lipids [36]. However, the oximes are clearly less reactive than the vinyl esters, and their irreversibility is not as explicit as when using the enol esters [30,36].

After the successful activation of the drugs as oxime esters, the task was to have them incorporated exclusively into the terminal positions of the benzyl-protected glycerols. As in our previous cases, with EPA and DHA activated as oxime esters to acylate glycerol and ether lipids [36], we tried to perform the reaction in dry dichloromethane at room temperature by using the immobilized *Candida antarctica* lipase B (CAL-B) with a 1.2-fold molar excess of the ibuprofen acetoxime (*S*)-**3**. There were no indications of a reaction taking place, and the situation remained the same when the temperature was increased to 40 °C.

When, in one of the attempts, the solvent accidentally evaporated off from the reaction mixture, we noticed that a reaction had indeed taken place. This prompted us to perform the reaction without a solvent at 40 °C. Since the starting material and the product displayed identical elution properties in all solvent systems tried, TLC was not an option to monitor the progress of the reaction. Instead, we had to depend on ^1^H NMR spectroscopy and the characteristic peaks belonging to the glyceryl protons of individual acylglycerol derivatives involved. This became very useful for monitoring the progress of the reaction. This is illustrated in Figure S1 in the Supplementary Materials.

This is evident from the figure’s dramatic changes that occurred in the glyceryl proton region of the spectra upon acylation with the drug entity. From our previous studies, the typical patterns of all five glyceryl protons of the starting material and the product are easily recognised and assigned, making it quite straightforward to monitor the progress of the reaction. It is evident that after 6 h, a substantial part of the starting material had already been converted into the product. After 24 h, there was evidently some starting material present, but since virtually no changes took place in the spectrum after 30 h, it was decided to work up the reaction.

The product (*R*,*S*′)-**5** was furnished as a liquid in an 86% yield after purification by flash column chromatography using boric-acid-impregnated silica gel to avoid acyl migration [1,30,38]. Similarly, the corresponding (*S*,*S*′)-**5** diastereomer was obtained from 3-*O*-benzyl-*sn*-glycerol in a 92% yield. The yields, along with the specific rotation of these intermediates, are shown in Table 1. In the reactions involving the (*S*)-ibuprofen acetoxime (*S*)-**3** and the benzyl-protected glycerols, there were no signs of any acyl migration taking place despite the heating at 40 °C. This may be surprising, but it should be pointed out that 1-*O*-alkylglycerols are less prone to undergo acyl migration than mono- and diacylated glycerols [36].

**Table 1:** Summary of the yields and specific rotation of the intermediates (*R*,*S*′)-**5**, (*S*,*S*′)-**5**, (*R*,*S*′)-**6** and (*S*,*S*′)-**6** obtained from the lipase-promoted acylation with the drugs.

Compound	*sn*-1	*sn*-2	*sn*-3	Yields	${\left[\alpha \right]}_{D}^{20}$
(*R*,*S*′)-**5**	OBn	OH	Ibu	86%	+25.0
(*S*,*S*′)-**5**	Ibu	OH	OBn	92%	+20.7
(*R*,*S*′)-**6**	OBn	OH	Nap	69%	+53.8
(*S*,*S*′)-**6**	Nap	OH	OBn	65%	+47.7

The corresponding acylation of the benzyl-protected glycerols with the acetoxime-activated naproxen (*S*)-**4** became far more of a challenge. The main reason was that (*S*)-**4**, unlike (*S*)-**3**, is a solid with a melting point of 42.3–43.4 °C and did not mix well with the glycerol substrate. This resulted in a significantly lower reaction rate compared to the previous ibuprofen case, even though the excess of the acetoxime was lowered to aid the solubility. Extreme care was also needed in terms of acyl migration, which became noticeable before the reaction proceeded to completion and resulted in significantly lower yields. This was clearly related to the prolonged heating at 40 °C over 40 h.

As before, we were dependent on the ^1^H NMR spectroscopy to monitor the progress of the reaction. Figure S2 in the Supplementary Materials shows the progress of the reaction based on the pattern of peaks characteristic of the glycerol derivatives involved. It took a long time for the reaction to start, and after 6 h, virtually no product had formed. The reaction mixture kept solidifying, but as the reaction gradually started, this problem decreased. After 23 h, the conversion reached 25%, and after 31 h, it reached 46%.

The conversion kept increasing as more of the acetoxime ester underwent a reaction, and after 47 h, it had reached 68%, but at that stage, an unwanted product of acyl migration started to appear in the spectrum, as is clearly evident from the corresponding spectrum in Figure S2. Therefore, we decided to terminate the reaction at this stage, even though it had not proceeded to completion. After work up and purification by column flash chromatography on silica gel impregnated with boric acid, as before, the product (*R*,*S*′)-**6** was a white solid free of the unwanted acyl migrated product, with a 69% yield. The corresponding diastereomer (*S*,*S*′)-**6** was similarly obtained as a white solid in a 65% yield. The yields, along with the specific rotation of these intermediates, are shown in Table 1.

Alternatively, the possibility of a regioselective acylation of 1-*O*-benzyl-*sn*-glycerol with (*S*)-ibuprofen) as a free acid by using the EDCI/DMAP coupling agent was investigated. This resulted in a mixture comprised of the desired product (*R*,*S*′)-**5** possessing the drug acylated at the *sn*-3 position, obtained as the major product (62%), along with its undesired monoacylated regioisomer possessing the drug at the *sn*-2 position (15%) and the diacylated product (23%). This was established by a ^1^H NMR analysis of the product mixture from the reaction.

### 2.2. The Removal of the Benzyl Protective Group

In the second step of the second category of prodrug synthesis, all four ibuprofen and naproxen derivatives (*R*,*S*′)-**5**, (*S*,*S*′)-**5**, (*R*,*S*′)-**6** and (*R*,*S*′)-**6** were subjected to catalytic hydrogenolysis for the removal of the benzyl protective group. A protocol identical to that of the previous synthesis of the first category of TAG prodrugs was followed by using a Pd/C catalyst in a mixture of THF and n-hexane under atmospheric pressure at r.t., using a catalytic amount of perchloric acid to initiate the reaction [1].

The reactions proceeded very smoothly to produce the monoacylglycerol (MAG) products in excellent yields (93–99%) after only a 12 min reaction time. The ibuprofen derivatives were obtained as liquids, whereas the corresponding naproxen derivatives were obtained as crystalline material after purification by boric-acid-impregnated flash silica gel chromatography. The yields and specific optical rotation values are revealed in Table 2 for all four MAG products obtained from the deprotection reactions.

**Table 2:** Summary of the yields and specific rotation of the intermediates (*R*,*S*′)-**7**, (*S*,*S*′)-**7**, (*R*,*S*′)-**8** and (*S*,*S*′)-**8** obtained from the debenzylation reaction.

Compound	*sn*-1	*sn*-2	*sn*-3	Yields	${\left[\alpha \right]}_{D}^{20}$
(*R*,*S*′)-**7**	OH	OH	Ibu	98%	+42.9
(*S*,*S*′)-**7**	Ibu	OH	OH	93%	+33.9
(*R*,*S*′)-**8**	OH	OH	Nap	93%	+43.5
(*S*,*S*′)-**8**	Nap	OH	OH	99%	+34.3

Despite using perchloric acid, no acyl migration was observed to take place, but like before [1], care was taken in neutralising the reaction mixture with sodium bicarbonate after the reaction was completed. This is evident from the glyceryl proton region of the ^1^H NMR spectra in Figure S3 in the Supplementary Materials, providing a comparison between the glyceryl proton region of the product (*R*,*S*′)-**7** and its precursor (*R*,*S*′)-**5**, showing a spectrum typical of 1-MAGs with no signs of acyl migration.

### 2.3. The Enzymatic Coupling of the SFAs

The third step involved a second lipase-promoted acylation of the medium-chain caproic, caprylic, capric, and lauric acids (C6:0, C8:0, C10:0, and C12:0), which were activated as vinyl esters, on the terminal position of the MAGs already acylated with the drugs obtained from the previous step. The advantages offered by using vinyl esters as acylating agents in terms of faster irreversible reactions and milder conditions to maintain the excellent regioselectivity of the lipase and to avoid acyl migration have been discussed in detail in previous reports [1,30,36].

As anticipated, the immobilized CAL-B acylated the drug derivatives (*R*,*S*′)-**6** and (*S*,*S*′)-**6** exclusively at the primary alcohol position to accomplish the ibuprofen-containing products (*R*,*S*′)-**9a**–**d** and (*S*,*S*′)-**9a**–**d**, as was confirmed by ^1^H NMR spectroscopy. Like before, the reactions were performed in dry dichloromethane at r.t., but it took the lipase significantly longer time to complete the reactions (4–6 h) compared to the previous case of the first category of TAG prodrug synthesis [1] involving the benzyl-protected glycerols (90 min). The yields after purification by boric-acid-impregnated silica gel flash chromatography were, in most cases, very-high-to-excellent and varied from 74 to 97%. Table 3 shows the identity, yields, and specific optical activity of the resulting 1,3-diacylglycerol (1,3-DAG) products involving ibuprofen in accordance with the reaction schemes in Figure 2 and Figure 3.

**Table 3:** Summary of the yields and specific rotation of the intermediates (*R*,*S*′)-**9a**–**d** and (*S*,*S*′)-**9a**–**d** obtained from the second lipase-promoted reaction.

Compound	*sn*-1	*sn*-2	*sn*-3	Yields	${\left[\alpha \right]}_{D}^{20}$
(*R*,*S*′)-**9a**	C6:0	OH	Ibu	80%	+22.7
(*R*,*S*′)-**9b**	C8:0	OH	Ibu	80%	+22.9
(*R*,*S*′)-**9c**	C10:0	OH	Ibu	88%	+25.3
(*R*,*S*′)-**9d**	C12:0	OH	Ibu	88%	+26.8
(*S*,*S*′)-**9a**	Ibu	OH	C6:0	94%	+21.0
(*S*,*S*′)-**9b**	Ibu	OH	C8:0	94%	+21.2
(*S*,*S*′)-**9c**	Ibu	OH	C10:0	74%	+22.6
(*S*,*S*′)-**9d**	Ibu	OH	C12:0	97%	+24.2

All ibuprofen-containing products, (*R*,*S*′)-**9a**–**c** and (*S*,*S*′)-**9a**–**c**, were obtained as colourless oils, whereas (*R*,*S*′)-**9d** and (*S*,*S*′)-**9d**, possessing the longest chain (C12:0), were obtained as crystalline material.

The corresponding reactions involving the naproxen derivatives (*R*,*S*′)-**7** and (*S*,*S*′)-**7** to accomplish the 1,3-DAG products (*R*,*S*′)-**10a**–**d** and (*S*,*S*′)-**10a**–**d** provided results quite comparable to those obtained for the ibuprofen 1,3-DAG products in terms of reaction time (4–6 h) and yields (somewhat lower, 70–92%). In accordance with the reaction schemes in Figure 2 and Figure 3, the identity, yields, and specific optical activity of the resulting 1,3-DAG products involving naproxen are shown in Table 4.

**Table 4:** Summary of the yields and specific rotation of the intermediates (*R*,*S*′)-**10a**–**d** and (*S*,*S*′)-**10a**–**d** obtained from the second lipase-promoted reaction.

Compound	*sn*-1	*sn*-2	*sn*-3	Yields	${\left[\alpha \right]}_{D}^{20}$
(*R*,*S*′)-**10a**	C6:0	OH	Nap	92%	+22.6
(*R*,*S*′)-**10b**	C8:0	OH	Nap	91%	+38.0
(*R*,*S*′)-**10c**	C10:0	OH	Nap	90%	+22.9
(*R,S*′)-**10d**	C12:0	OH	Nap	83%	+27.5
(*S*,*S*′)-**10a**	Nap	OH	C6:0	70%	+21.5
(*S*,*S*′)-**10b**	Nap	OH	C8:0	70%	+23.1
(*S*,*S*′)-**10c**	Nap	OH	C10:0	75%	+23.6
(*S*,*S*′)-**10d**	Nap	OH	C12:0	91%	+25.5

As in the case of the ibuprofen 1,3-DAG derivatives, all corresponding naproxen-containing products (*R*,*S*′)-**10a**–**c** and (*S*,*S*′)-**10a**–**c** were obtained as colourless oils, whereas (*R*,*S*′)-**10d** and (*S*,*S*′)-**10d** possessing the longest saturated chain were obtained as crystalline materials. It is evident that the task of the lipase to acylate the primary position of the 1-MAGs containing the drugs was more of a challenge compared to the corresponding 1-*O*-benzylglycerols involved in the corresponding synthesis of the first category of TAG prodrugs. This is clearly reflected in longer reaction times and lower yields.

The structures of the 1,3-DAGs were confirmed by the characteristic pattern for the glyceryl proton segment of their ^1^H-NMR spectra. Figure S4 in the Supplementary Materials presents a comparison of the glyceryl proton segment of the MAG starting material (*R*,*S*′)-**7** and the 1,3-DAG product (*R*,*S*′)-**9c**. The characteristic pattern of peaks for the two types of acylglycerols is clear. Upon the second acylation, the three protons belonging to the *sn*-1 and *sn*-2 positions underwent a significant down-field shift, merging with the *sn*-3 protons, to form a multiplet at δ 4.23–3.94 ppm that is characteristic of 1,3-DAGs. No sign of acyl migration was detected in the spectra, which would certainly distort the peak pattern and result in additional peaks in the glyceryl proton region of these products.

### 2.4. The Coupling of the PUFA

The fourth and last step of the second category of TAG prodrug synthesis involved a chemical coupling of EPA and DHA in the open *sn*-2 position of the 1,3-DAGs possessing the drug and the MCFA obtained from the previous step. Procedures already described from the synthesis of the first category of TAG prodrugs were followed using approximately 5–10% excess of EPA and DHA, with EDCI as a coupling agent in the presence of DMAP in dichloromethane at r.t. As before, no acyl migration was observed to take place [12,13,29].

All TAG products were obtained as yellowish to yellow oils in very-high-to-excellent yields in the majority of cases. The reactions involving DHA were observed to require a somewhat longer reaction time than those of EPA and produced somewhat lower yields. Table 5, Table 6, Table 7 and Table 8 outline the identity, yields, and specific optical activity of the products in accordance with the reaction schemes in Figure 2 and Figure 3. The TAG prodrug products (*R*,*S*′)-**11a**–**d** and (*S*,*S*′)-**11a**–**d**, possessing MCFA, EPA, and ibuprofen, are shown in Table 5.

**Table 5:** Summary of the yields and specific rotation of the TAG prodrug products (*R*,*S*′)-**11a**–**d** and (*S*,*S*′)-**11a**–**d**.

Compound	*sn*-1	*sn*-2	*sn*-3	Yields	${\left[\alpha \right]}_{D}^{20}$
(*R*,*S*′)-**11a**	C6:0	EPA	Ibu	96%	+8.29
(*R*,*S*′)-**11b**	C8:0	EPA	Ibu	88%	+12.0
(*R*,*S*′)-**11c**	C10:0	EPA	Ibu	83%	+12.2
(*R*,*S*′)-**11d**	C12:0	EPA	Ibu	87%	+8.35
(*S*,*S*′)-**11a**	Ibu	EPA	C6:0	89%	+8.27
(*S*,*S*′)-**11b**	Ibu	EPA	C8:0	90%	+8.60
(*S*,*S*′)-**11c**	Ibu	EPA	C10:0	84%	+9.74
(*S*,*S*′)-**11d**	Ibu	EPA	C12:0	89%	+10.4

Similarly, the corresponding TAG prodrug products (*R*,*S*′)-**12a**–**d** and (*R*,*S*′)-**12a**–**d** possessing an MCFA, EPA, and naproxen are shown in Table 6.

**Table 6:** Summary of the yields and specific rotation of the TAG prodrug products (*R*,*S*′)-**12a**–**d** and (*S*,*S*′)-**12a**–**d**.

Compound	*sn*-1	*sn*-2	*sn*-3	Yields	${\left[\alpha \right]}_{D}^{20}$
(*R*,*S*′)-**12a**	C6:0	EPA	Nap	80%	+9.29
(*R*,*S*′)-**12b**	C8:0	EPA	Nap	78%	+9.17
(*R*,*S*′)-**12c**	C10:0	EPA	Nap	77%	+9.62
(*R*,*S*′)-**12d**	C12:0	EPA	Nap	86%	+5.38
(*S*,*S*′)-**12a**	Nap	EPA	C6:0	95%	+12.4
(*S*,*S*′)-**12b**	Nap	EPA	C8:0	95%	+11.2
(*S*,*S*′)-**12c**	Nap	EPA	C10:0	86%	+10.5
(*S*,*S*′)-**12d**	Nap	EPA	C12:0	86%	+9.60

Table 7 outlines the TAG prodrug products (*R*,*S*′)-**13a**–**d** and (*S*,*S*′)-**13a**–**d** possessing an MCFA, DHA, and ibuprofen.

**Table 7:** Summary of the yields and specific rotation of the TAG prodrug products (*R*,*S*′)-**13a**–**d** and (*S*,*S*′)-**13a**–**d**.

Compound	*sn*-1	*sn*-2	*sn*-3	Yields	${\left[\alpha \right]}_{D}^{20}$
(*R*,*S*′)-**13a**	C6:0	DHA	Ibu	79%	+6.90
(*R*,*S*′)-**13b**	C8:0	DHA	Ibu	85%	+5.77
(*R*,*S*′)-**13c**	C10:0	DHA	Ibu	72%	+7.91
(*R*,*S*′)-**13d**	C12:0	DHA	Ibu	84%	+5.42
(*S*,*S*′)-**13a**	Ibu	DHA	C6:0	65%	+8.22
(*S*,*S*′)-**13b**	Ibu	DHA	C8:0	77%	+8.57
(*S*,*S*′)-**13c**	Ibu	DHA	C10:0	80%	+9.60
(*S*,*S*′)-**13d**	Ibu	DHA	C12:0	79%	+9.67

Finally, the corresponding TAG prodrug products (*R*,*S*′)-**14a**–**d** and (*S*,*S*′)-**14a**–**d** possessing an MCFA, EPA, and naproxen are shown in Table 8.

**Table 8:** Summary of the yields and specific rotation of the TAG prodrug products (*R*,*S*′)-**14a**–**d** and (*S*,*S*′)-**14a**–**d**.

Compound	*sn*-1	*sn*-2	*sn*-3	Yields	${\left[\alpha \right]}_{D}^{20}$
(*R*,*S*′)-**14a**	C6:0	DHA	Nap	84%	+8.00
(*R*,*S*′)-**14b**	C8:0	DHA	Nap	83%	+8.37
(*R*,*S*′)-**14c**	C10:0	DHA	Nap	91%	+4.20
(*R*,*S*′)-**14d**	C12:0	DHA	Nap	86%	+6.71
(*S*,*S*′)-**14a**	Nap	DHA	C6:0	79%	+12.6
(*S*,*S*′)-**14b**	Nap	DHA	C8:0	68%	+11.8
(*S*,*S*′)-**14c**	Nap	DHA	C10:0	79%	+10.8
(*S*,*S*′)-**14d**	Nap	DHA	C12:0	83%	+10.0

Figure S5 of the Supplementary Materials provides a comparison of the glyceryl proton region of the product (*R*,*S*′)-**11c** and the precursor (*R*,*S*′)-**9c**. As may be noticed, changes anticipated for TAGs have taken place, with a dramatic down-field shift of the protons belonging to the *sn*-2 position upon acylation into that position. The remaining *sn*-1 and *sn*-3 protons now resonate as two well-dispersed doublets characteristic of TAGs.

As indicated earlier, the glyceryl proton segment of the ^1^H NMR spectra (δ 5.40–3.45 ppm) is of high utility to authenticate the structure and establish the purity of individual acylglycerol derivatives engaged in the TAG synthesis. This relies on the distinctive patterns of proton peaks representing the acylglycerols. This is also of uttermost importance for maintaining the regiocontrol through the accurate detection of unwanted products related to acyl migration, as has been discussed and described in detail in previous reports [1,12,13,36]. In the presented work, we have benefited from the spectral details obtained from the ^1^H NMR and the 2D-NMR ^1^H-^1^H-COSY spectroscopy that has enabled a full assignment of the ^1^H NMR data to confirm the chemical purity of all intermediates and products involved.

## 3. Materials and Methods

### 3.1. General Information

The ^1^H- and ^13^C-NMR analysis was performed on a 400 MHz Bruker Avance NEO 400 spectrometer (Bruker Switzerland AG, Faellanden, Switzerland) by using deuterochloroform as a solvent. Prior to use, the solvent was treated by filtration through aluminum oxide to remove acid impurities. Chemical shifts (δ) are indicated in parts per million (ppm) from tetramethylsilane (TMS) using the solvent resonance as an internal standard. The coupling constants (*J*) are reported in Hertz (Hz) with the following abbreviations to describe the multiplicity: s, singlet; d, doublet; t, triplet; q, quartet; dd, doublet of doublets; dt, doublet of triplets; AB q, AB-quartet; m, multiplet. Regarding ^13^C-NMR, multiple carbon nuclei contributing to a signal are indicated in parentheses after the chemical shift value. A Nicolet Avatar FT-IR (E.S.P.) spectrometer (Thermo Scientific, Madison, WI, USA) was used to record infrared spectra with sodium chloride windows (NaCl) for liquids or potassium bromide pellets (KBr) for solid compounds. The peaks are described as follows: s, strong; vs, very strong; m, medium; w, weak; br, broad. A Bruker OTOF-Q Compact ESI mass spectrometer (Bruker Daltonic, Bremen, Germany) was used to record the high-resolution mass spectra. An Autopol V automatic Polarimeter from Rudolph Research Analytical (Hacketstown, NJ, USA) was used for the optical activity measurements utilizing a 40T-2.5-100-0.7 TempTrol polarimetric cell with a 2.5 mm inside diameter, 100 mm optical length, and 0.7 mL volume, with c (concentration) referring to g sample/100 mL. A Büchi m-560 melting point apparatus (Büchi, Uster, Switzerland) was used to determine melting points. Silica plates from SiliCycle (Québec, QC, Canada) were used to perform TLC monitoring with the use of a 4% PMA solution in methanol to develop the plates. The silica gel impregnated with boric acid was prepared as follows: Boric acid (4 g) was dissolved in methanol (100 mL), followed by the addition of silica gel (55 g). The resulting slurry was swirled for a few minutes, the methanol evaporated off, and the resulting silica preparation dried in vacuo for 6 h at 40 °C.

All solvents were purchased from Sigma-Aldrich (Steinheim, Germany) and were used without purification unless otherwise stated. They include deuterated chloroform (99.8% D), dichloromethane (99.8%), diethyl ether (≥99.8%), ethyl acetate (≥99.7%), ethanol (≥99.8%), hexane (>99%), methanol (99.9%), and tetrahydrofuran (THF) (99.9%), which was dried over sodium wire in the presence of benzophenone under a dry nitrogen atmosphere before use. Dichloromethane was kept over molecular sieves under dry nitrogen after it was brought to use. All chemicals were used without further purification. The following chemicals were purchased from Sigma-Aldrich: acetone oxime (98%), benzyl bromide (98%), boric acid (≥99.5%), DMAP (4-dimethylaminopyridine, >99%), EDCI (1-ethyl-3-(3-dimethylaminopropyl)carbodiimide, >99%), hydrochloric acid (37%), (S)-ibuprofen (99%), magnesium sulfate (≥99.5%), palladium (10%) on activated charcoal catalyst, perchloric acid (>70%), phosphomolybdic acid, sodium bicarbonate (≥99.0%), sodium hydride (60% dispersion in mineral oil), sodium sulfate (≥99%), (R)-solketal (98%, 98% ee), (S)-solketal (98%, 99% ee), and vinyl dodecanoate (≥99%). The following chemicals were obtained from TCI Europe (Zwinderecht, Belgium): vinyl decanoate (>99%), vinyl hexanoate (>99%), and vinyl octanoate (>99%). Novozymes Denmark (Bagsvaerd, Denmark) donated the immobilized Candida antarctica lipase B (CAL-B) as a gift. Ethyl esters of EPA (98%) and DHA (≥95%) were donated as gifts from Pronova Biopharma (Sandefjord, Norway). They were hydrolyzed to their corresponding free acids [1]. (S)-Naproxen was obtained from Prof. Thorsteinn Loftsson at the Faculty of Pharmaceutical Sciences at the University of Iceland (Reykjavik, Iceland). The silica gel for the column chromatography (40–63 µm, 0.060–0.300, F60) was obtained from SiliCycle. The TLC plates used for monitoring the reactions were dipped into a methanol solution of phosphomolybdic acid (PMA) for developing the spots.

### 3.2. Activation of Drugs as Oximes

#### 3.2.1. Synthesis of (*S*)-Propan-2-one-*O*-(2-(4-isobutylphenyl)propanoyl oxime, (*S*)-**3**

To a solution of (*S*)-ibuprofen (94 mg, 0.456 mmol), DMAP (16 mg, 0.131 mmol), and EDCI (105 mg, 0.553 mmol) in CH_2_Cl_2_ (2 mL) were added to acetoxime (34 mg, 0.465 mmol), and the solution was stirred on a magnetic stirrer at room temperature for 3–4 h. The reaction was disconnected by passing the reaction mixture through a short column packed with silica gel by using ethyl acetate/petroleum ether (3:2) as an eluent. The solvent was removed in vacuo on a rotary evaporator, and the crude product was applied to a silica gel flash chromatography using ethyl acetate/petroleum ether (1:1) as an eluent, which produced the product (*S*)-**3** as a slightly yellow liquid in a 96% yield (115 mg, 0.440 mmol). ${\left[\alpha \right]}_{D}^{20}$ = −7.18 (c. 7.3, CH_2_Cl_2_). IR (NaCl, ν_max_/cm^−1^): 3058 (vs), 2958 (vs), 2931 (vs), 2853 (vs), 1758 (vs), 1653 (s). ^1^H NMR (400 MHz, CDCl_3_) δ_H_: 7.27–7.17 (m, 2H, Ibu-2,6), 7.14–7.01 (m, 2H, Ibu-3,5), 3.79 (q, *J* = 7.2 Hz, 1H, C*H*CH_3_), 2.44 (d, *J* = 7.2 Hz, 2H, C*H*_2_CH), 1.99 (s, 3H, NC(CH_3_)_2_), 1.83 (nonet, *J* = 6.7 Hz, 1H, C*H*(CH_3_)_2_), 1.83 (s, 3H, NC(CH_3_)_2_), 1.56 (d, *J* = 7.2 Hz, 3H, CHC*H*_3_), 0.88 (d, *J* = 6.7 Hz, 6H, CH(C*H*_3_)_2_) ppm. ^13^C{H} NMR (101 MHz, CDCl_3_) δ_C_: 171.9, 164.4 (N=C), 140.7, 137.5 (2), 129.4 (2), 127.3, 45.1, 44.3, 30.3, 22.5 (2), 22.1, 18.5, 16.9 ppm. HRMS (ESI) *m*/*z*: [M + Na]^+^ calcd for C_16_H_23_NO_2_Na 284.1621; found, 284.1618.

#### 3.2.2. Synthesis of (*S*)-Propan-2-one-*O*-(2-(6-methoxynaphthalen-2-yl)propanoyl oxime, (*S*)-**4**

The same procedure was followed as described for (*S*)-**3** using (*S*)-naproxen (105 mg, 0.456 mmol), DMAP (14 mg, 0.115 mmol), EDCI (109 mg, 0.569 mmol) and acetoxime (33 mg, 0.451 mol) in CH_2_Cl_2_ (2 mL) were added acetoxime (33 mg, 0.451 mmol). Purification on silica gel flash chromatography using ethyl acetate/petroleum ether (1:1) as an eluent, followed by recrystallization from n-hexane, produced the product (*S*)-**4** as a white solid in a quantitative yield (130 mg, 0.456 mmol). M.p. 42.3–43.4 °C. ${\left[\alpha \right]}_{D}^{20}$ = −12.3 (c. 16.5, CH_2_Cl_2_). IR (NaCl, ν_max_/cm^−1^): 3052 (vs), 2956 (vs), 2932 (vs), 2863 (vs), 2848 (vs), 1753 (vs), 1652 (s). ^1^H NMR (400 MHz, CDCl_3_) δ_H_: 7.72–7.67 (m, 3H, Nap-1,4,8), 7.44 (dd, *J* = 8.5, 1.9 Hz, 1H, Nap-3), 7.14 (dd, *J* = 8.9, 2.5 Hz, 1H, Nap-7), 7.11 (d, *J* = 2.5 Hz, 1H, Nap-5), 3.96 (q, *J* = 7.2 Hz, 1H, C*H*CH_3_), 3.91 (s, 3H, OCH_3_), 1.99 (s, 3H, NC(CH_3_)_2_), 1.83 (s, 3H, NC(CH_3_)_2_), 1.65 (d, *J* = 7.2 Hz, 3H, CHC*H*_3_) ppm. ^13^C{H} NMR (101 MHz, CDCl_3_) δ_C_: 171.9, 164.4, 157.8, 135.4, 133.8, 129.4, 129.0, 127.3, 126.4, 126.1, 119.1, 105.7, 55.4, 44.6, 22.1, 18.7, 17.0 ppm. HRMS (ESI) *m*/*z*: [M + Na]^+^ calcd for C_17_H_19_NO_3_Na 308.1257; found, 308.1251.

### 3.3. Enzymatic Coupling of the Drugs: Synthesis of (R,S′)-***5***, (S,S′)-***5***, (R,S′)-***6***, and (S,S′)-***6***

#### 3.3.1. Synthesis of 1-*O*-Benzyl-3-[(*S*)-2-(4-isobutylphenyl)propanoyl]-*sn*-glycerol, (*R*,*S*′)-**5**

Immobilized CAL-B (40 mg) was added to a mixture of 1-*O*-benzyl-*sn*-glycerol (100 mg, 0.549 mmol) and ibuprofen acetoxime ester (*S*)-**3** (163 mg, 0.659 mmol). The resulting mixture was stirred at 40 °C for 31 h in a nitrogen atmosphere. The lipase preparation was removed by filtration, and the solvent was distilled off in vacuo on a rotary evaporator. The concentrate was applied to a 4% boric-acid-impregnated flash silica gel chromatography using petroleum ether/ethyl acetate (3:2) as an eluent. The first fraction from the column was contaminated with some oxime starting material, and repeated chromatography was required. The product (*R*,*S*′)-**5** was produced as a colorless liquid in an 86% yield (175 mg, 0.472 mmol) from the combined fractions. ${\left[\alpha \right]}_{D}^{20}$ = +25.0 (c. 14.0, CH_2_Cl_2_). IR (NaCl, ν_max_/cm^−1^): 3458 (br s), 3089 (s), 3462 (br s), 3089 (s), 3062 (s), 3028 (s), 2954 (vs), 2925 (vs), 2868 (vs), 1736 (vs), 1607. ^1^H NMR (400 MHz, CDCl_3_) δ_H_: 7.38–7.27 (m, 5H, Ph-H), 7.21–7.16 (m, 2H, Ibu-2,6), 7.10–7.06 (m, 2H, Ibu-3,5), 4.47 (s, 2H, CH_2_Ph), 4.18 (dd, J = 11.4, 4.7 Hz, 1H, CH_2_ *sn*-3), 4.13 (dd, *J* = 11.4, 6.1 Hz, 1H, CH_2_ *sn*-3), 3.99–3.95 (m, 1H, CH *sn*-2), 3.72 (q, *J* = 7.2 Hz, 1H, C*H*CH_3_), 3.44 (dd, *J* = 9.6, 4.5 Hz, 1H, CH_2_ *sn*-1), 3.37 (dd, *J* = 9.6, 5.9 Hz, 1H, CH_2_ *sn*-1), 2.44 (d, *J* = 7.2 Hz, 2H, C*H*_2_CH(CH_3_)_2_), 1.84 (nonet, *J* = 6.8 Hz, 1H, C*H*(CH_3_)_2_), 1.49 (d, *J* = 7.2 Hz, 3H, CHC*H*_3_), 0.89 (d, *J* = 6.8 Hz, 6H, CH(C*H*_3_)_2_) ppm. ^13^C{H} NMR (101 MHz, CDCl_3_) δ_C_: 174.9, 140.8, 137.8, 137.7, 129.5 (2), 128.6 (2), 128.0 (2), 127.8 (2), 127.3, 73.6, 70.8, 69.0, 65.7, 45.2, 30.3, 22.5, 18.53 (2), 18.47 ppm. HRMS (ESI) *m*/*z*: [M + Na]^+^ calcd for C_23_H_30_O_4_Na 393.2036; found, 393.2030.

#### 3.3.2. Synthesis of 3-*O*-Benzyl-1-[(*S*)-2-(4-isobutylphenyl)propanoyl]-*sn*-glycerol, (*S*,*S*′)-**5**

The same procedure was followed as described for (*R*,*S*′)-**5** using 3-*O*-benzyl-*sn*-glycerol (100 mg, 0.549 mmol), ibuprofen acetoxime ester (*S*)-**3** (163 mg, 0.659 mmol), and immobilized CAL-B (45 mg). Purification on a 4% boric-acid-impregnated flash silica gel column using petroleum ether/ethyl acetate (3:2) as an eluent produced the product (*S*,*S*′)-**5** as a colorless liquid in a 92% yield (187 mg, 0.505 mmol). As before, the first fraction from the column was contaminated with some oxime starting material and required repeated chromatography. ${\left[\alpha \right]}_{D}^{20}$ = +20.7 (c. 11.0, CH_2_Cl_2_). IR (NaCl, ν_max_/cm^−1^): 3458 (br s), 3089 (s), 3062 (s), 3028 (s), 2954 (vs), 2925 (vs), 2865 (vs), 1740 (vs). ^1^H NMR (400 MHz, CDCl_3_) δ_H_: 7.37–7.27 (m, 5H, Ph-H), 7.18 (d, *J* = 8.1 Hz, 2H, Ibu-2,6), 7.08 (d, *J* = 8.1 Hz, 2H, Ibu-3,5), 4.47 (s, 2H, CH_2_Ph), 4.16 (d, *J* = 5.2 Hz, 2H, CH_2_ *sn*-1), 3.98–3.94 (m, 1H, CH *sn*-2), 3.72 (q, *J* = 7.2 Hz, 1H, C*H*CH_3_), 3.42 (dd, *J* = 9.6, 4.5 Hz, 1H, CH_2_ *sn*-3), 3.36 (dd, *J* = 9.6, 5.9 Hz, 1H, CH_2_ *sn*-3), 2.44 (d, *J* = 7.2 Hz, 2H, C*H*_2_CH(CH_3_)_2_), 1.84 (nonet, *J* = 6.8 Hz, 1H, C*H*(CH_3_)_2_), 1.49 (d, *J* = 7.2 Hz, 3H, CHC*H*_3_), 0.89 (d, *J* = 6.8 Hz, 6H, CH(C*H*_3_)_2_) ppm. ^13^C{H} NMR (101 MHz, CDCl_3_) δ_C_: 174.9, 140.8, 137.9, 137.7, 129.5 (2), 128.6 (2), 128.0 (2), 127.9 (2), 127.3, 73.6, 70.8, 69.0, 65.6, 45.2, 30.3, 22.5, 18.52 (2), 18.46 ppm. HRMS (ESI) *m*/*z*: [M + Na]^+^ calcd for C_23_H_30_O_4_Na 393.2036; found, 393.2031.

#### 3.3.3. Synthesis of 1-*O*-Benzyl-3-[(*S*)-2-(6-methoxynaphthalen-2-yl)]-*sn*-glycerol, (*R*,*S*′)-**6**

The same procedure was followed as described for (*R*,*S*′)-**5** using 1-*O*-benzyl-*sn*-glycerol (100 mg, 0.549 mmol), naproxen acetoxime ester (*S*)-**4** (172 mg, 0.604 mmol), and immobilized CAL-B (38 mg). Purification on a 4% boric-acid-impregnated flash silica gel column using petroleum ether/ethyl acetate (3:2) as an eluent resulted in a first fraction contaminated with the starting material that, as before, required repeated chromatography. Recrystallization of the combined fractions from n-hexane produced the product (*R*,*S*′)-**6** as a white solid in a 69% yield (149 mg, 0.378 mmol). M.p. 51.7–52.1 °C. ${\left[\alpha \right]}_{D}^{20}$ = +53.8 (c. 1.9, CH_2_Cl_2_). IR (NaCl, ν_max_/cm^−1^): 3538 (br s), 3057 (s), 2973 (vs), 2936 (vs), 2909 (vs), 2864 (vs), 1719 (vs), 1632 (s), 1605 (vs). ^1^H NMR (400 MHz, CDCl_3_) δ_H_: 7.71–7.65 (m, 3H, Nap-1,4,8), 7.38 (dd, *J* = 8.4, 1.9 Hz, 1H, Nap-3), 7.34–7.22 (m, 5H, Ph-H), 7.14 (dd, *J* = 8.9, 2.5 Hz, 1H, Nap-7), 7.10 (d, *J* = 2.5 Hz, 1H, Nap-5), 4.40 (s, 2H, CH_2_Ph), 4.19 (dd, *J* = 11.5, 4.8 Hz, 1H, CH_2_ *sn*-3), 4.14 (dd, *J* = 11.5, 6.0 Hz, 1H, CH_2_ *sn*-3), 4.00–3.87 (m, 1H, CH *sn*-2), 3.91 (s, 3H, OCH_3_), 3.87 (q, *J* = 7.2 Hz, 1H, C*H*CH_3_), 3.40 (dd, *J* = 9.6, 4.4 Hz, 1H, CH_2_ *sn*-1), 3.32 (dd, *J* = 9.6, 6.0 Hz, 1H, CH_2_ *sn*-1), 2.31 (d, *J* = 5.2 Hz, 1H, OH), 1.58 (d, *J* = 7.2 Hz, 3H, CHC*H*_3_) ppm. ^13^C{H} NMR (101 MHz, CDCl_3_) δ_C_: 174.8, 157.9, 137.8, 135.6, 133.9, 129.4, 129.1, 128.6, 128.0 (2), 127.8 (2), 127.4, 126.3, 129.1, 119.2, 105.8, 73.6, 70.9, 69.0, 65.8, 55.5, 45.5, 18.5 ppm. HRMS (ESI) *m*/*z*: [M + Na]^+^ calcd for C_24_H_26_O_5_Na 417.1672; found, 417.1663.

#### 3.3.4. Synthesis of 3-*O*-Benzyl-1-[(*S*)-2-(6-methoxynaphthalen-2-yl)]-*sn*-glycerol, (*S*,*S*′)-**6**

The same procedure was followed as described for (*R*,*S*′)-**5** using 3-*O*-benzyl-*sn*-glycerol (76 mg, 0.417 mmol), naproxen acetoxime ester (*S*)-**4** (172 mg, 0.439 mmol), and immobilized CAL-B (42 mg). Purification on a 4% boric-acid-impregnated flash silica gel column using petroleum ether/ethyl acetate (3:2) as an eluent resulted in a first fraction contaminated with the starting material that, as before, required repeated chromatography. Recrystallization of the combined fractions from n-hexane produced the product (*S*,*S*′)-**6** as a white solid in a 65% yield (107 mg, 0.272 mmol). M.p. 63.2–63.5 °C. ${\left[\alpha \right]}_{D}^{20}$ = +47.7 (c. 1.7, CH_2_Cl_2_). IR (NaCl, ν_max_/cm^−1^): 3540 (br s), 3053 (s), 2972 (vs), 2940 (vs), 2904 (vs), 2862 (vs), 1718 (vs), 1630 (s), 1607 (vs). ^1^H NMR (400 MHz, CDCl_3_) δ_H_: 7.71–7.64 (m, 3H, Nap-1,4,8), 7.38 (dd, *J* = 8.5, 1.9 Hz, 1H, Nap-3), 7.34–7.21 (m, 5H, Ph-H), 7.14 (dd, *J* = 8.9, 2.5 Hz, 1H, Nap-7), 7.10 (d, *J* = 2.5 Hz, 1H, Nap-5), 4.37 (s, 2H, CH_2_Ph), 4.17 (d, *J* = 5.5 Hz, 2H, CH_2_ *sn*-1), 4.00–3.90 (m, 1H, CH *sn*-2), 3.91 (s, 3H, OCH_3_), 3.88 (q, *J* = 7.2 Hz, 1H, C*H*CH_3_), 3.37 (dd, *J* = 9.6, 4.4 Hz, 1H, CH_2_ *sn*-3), 3.31 (dd, *J* = 9.6, 6.2 Hz, 1H, CH_2_ *sn*-3), 2.32 (d, *J* = 4.8 Hz, 1H, OH), 1.58 (d, *J* = 7.2 Hz, 3H, CHC*H*_3_) ppm. ^13^C{H} NMR (101 MHz, CDCl_3_) δ_C_: 174.8, 157.8, 137.8, 135.6, 133.9, 129.4, 129.1, 128.6, 128.0 (2), 127.8 (2), 127.4, 126.3, 126.1, 119.2, 105.8, 73.5, 70.8, 68.9, 65.7, 55.5, 45.5, 18.5 ppm. HRMS (ESI) *m*/*z*: [M + Na]^+^ calcd for C_24_H_26_O_5_Na 417.1672; found, 417.1671.

### 3.4. Removal of the Benzyl Protective Group: Synthesis of (R,S′)-***7***, (S,S′)-***7***, (R,S′)-***8***, and (S,S′)-***8***

#### 3.4.1. Synthesis of 3-[(*S*)-2-(4-Isobutylphenyl)propanoyl]-*sn*-glycerol, (*R*,*S*′)-**7**

A Pd/C catalyst (8 mg) was added to a 25 mL flame-dried two-necked round-bottom flask equipped with a magnetic stirrer under nitrogen atmosphere at room temperature. The flask was sealed with a septum, and a solution of 1-*O*-benzyl-3-[(*S*)-2-(4-isobutylphenyl)-propanoyl]-*sn*-glycerol (*R*,*S*′)-**5** (40 mg, 0.108 mmol) dissolved in dry THF (3.2 mL) was added with a syringe, followed by n-hexane (5.2 mL). A balloon filled with hydrogen gas that was mounted on a syringe was then stuck through the septum. Through stirring, the nitrogen atmosphere was replaced with hydrogen from the balloon by blowing it through the system. Then, a tiny drop of perchloric acid was added, and the solution was stirred vigorously at room temperature while being monitored with TLC. When the reaction had proceeded to an end, according to the TLC (approximately 12 min), the flask was promptly opened, and the acid was neutralized by adding NaHCO_3_ (s). Then, the solution was filtered, and the solvent was removed in vacuo on a rotary evaporator. The crude product was applied to a 4% boric-acid-impregnated flash silica gel chromatography using petroleum ether/ethyl acetate (2:3) as an eluent to produce the product (*R*,*S*′)-**7** as a pale-yellow oil in a 98% yield (30 mg, 0.107 mmol). ${\left[\alpha \right]}_{D}^{20}$ = +42.9 (c. 3.5, CH_2_Cl_2_). IR (NaCl, ν_max_/cm^−1^): 3423 (br s), 3063 (s), 3025 (s), 2954 (vs), 2923 (vs), 2867 (vs), 1740 (vs) ^1^H NMR (400 MHz, CDCl_3_) δ_H_: 7.19 (d, *J* = 8.1 Hz, 2H, Ibu-2,6), 7.10 (d, *J* = 8.1 Hz, 2H, H-4,6 Ibu), 4.22 (dd, *J* = 11.6, 4.6 Hz, 1H, CH_2_ *sn*-3), 4.10 (dd, *J* = 11.4, 6.1 Hz, 1H, CH_2_ *sn*-3), 3.87–3.80 (m, 1H, CH *sn*-2), 3.74 (q, *J* = 7.2 Hz, 1H, C*H*CH_3_), 3.57 (dd, *J* = 11.5, 4.0 Hz, 1H, CH_2_ *sn*-1), 3.45 (dd, *J* = 11.5, 5.6 Hz, 1H, CH_2_ *sn*-1), 3.10–2.75 (bm, 2H, OH), 2.45 (d, *J* = 7.2 Hz, 2H, C*H*_2_CH(CH_3_)_2_), 1.85 (nonet, *J* = 6.8 Hz, 1H, C*H*(CH_3_)_2_), 1.51 (d, *J* = 7.2 Hz, 3H, CHC*H*_3_), 0.89 (d, *J* = 6.8 Hz, 6H, CH(C*H*_3_)_2_) ppm. ^13^C{H} NMR (101 MHz, CDCl_3_) δ_C_: 175.2, 140.8, 137.4, 129.5 (2), 127.1 (2), 70.2, 65.4, 63.2, 45.1, 45.1, 30.2, 22.4 (2), 18.4 ppm. HRMS (ESI) *m*/*z*: [M + Na]^+^ calcd for C_16_H_24_O_4_Na 303.1567; found, 303.1563.

#### 3.4.2. Synthesis of 1-[(*S*)-2-(4-Isobutylphenyl)propanoyl]-*sn*-glycerol, (*S*,*S*′)-**7**

The same procedure was followed as described for (*R*,*S*′)-**7** using Pd/C (15 mg), 3-*O*-benzyl-1-[(*S*)-2-(4-isobutylphenyl)-propanoyl]-*sn*-glycerol (*S*,*S*′)-**5** (50 mg, 0.135 mmol), THF (4.0 mL) and n-hexane (6.5 mL). Purification on a 4% boric-acid-impregnated flash silica gel column using petroleum ether/ethyl acetate (2:3) as an eluent produced the product (*S*,*S*′)-**7** as a colorless liquid in a 93% yield (35 mg, 0.125 mmol). ${\left[\alpha \right]}_{D}^{20}$ = +33.9 (c. 2.0, CH_2_Cl_2_). IR (NaCl, ν_max_/cm^−1^): 3455 (br s), 3060 (s), 3028 (s), 2954 (vs), 2925 (vs), 2865 (vs), 1742 (vs). ^1^H NMR (400 MHz, CDCl_3_) δ_H_: 7.19 (d, *J* = 8.1 Hz, 2H, Ibu-2,6), 7.10 (d, *J* = 8.1 Hz, 2H, H-4,6 Ibu), 4.22 (dd, *J* = 11.6, 4.6 Hz, 1H, CH_2_ *sn*-1), 4.10 (dd, *J* = 11.4, 6.2 Hz, 1H, CH_2_ *sn*-1), 3.87–3.80 (m, 1H, CH *sn*-2), 3.74 (q, *J* = 7.2 Hz, 1H, C*H*CH_3_), 3.57 (dd, *J* = 11.5, 4.0 Hz, 1H, CH_2_ *sn*-3), 3.45 (dd, *J* = 11.5, 5.6 Hz, 1H, CH_2_ *sn*-3), 2.45 (d, *J* = 7.2 Hz, 2H, C*H*_2_CH(CH_3_)_2_), 2.35–2.19 (bs, 1H, OH), 1.82–1.92 (bs, 1H, OH), 1.85 (nonet, *J* = 6.8 Hz, 1H, C*H*(CH_3_)_2_), 1.51 (d, *J* = 7.2 Hz, 3H, CHC*H*_3_), 0.89 (d, *J* = 6.8 Hz, 6H, CH(C*H*_3_)_2_) ppm. ^13^C{H} NMR (101 MHz, CDCl_3_) δ_C_: 175.2, 140.8, 137.5, 129.5 (2), 127.1 (2), 70.2, 65.4, 63.2, 45.1, 45.1, 30.2, 22.4 (2), 18.4 ppm. HRMS (ESI) *m*/*z*: [M + Na]^+^ calcd for C_16_H_24_O_4_Na 303.1567; found, 303.1569.

#### 3.4.3. Synthesis of 3-[(*S*)-2-(6-Methoxynaphthalen-2-yl)]-*sn*-glycerol, (*R*,*S*′)-**8**

The same procedure was followed as described for (*R*,*S*′)-**7** using 1-*O*-benzyl-3-[(*S*)-2-(6-methoxynaphthalen-2-yl)]-*sn*-glycerol (*R*,*S*′)-**6** (130 mg, 0.330 mmol), THF (9.5 mL), n-hexane (16.5 mL) and Pd/C catalyst (25 mg). Purification on a 4% boric-acid-impregnated flash silica gel column using petroleum ether/ethyl acetate (2:3) as an eluent, followed by recrystallization from n-hexane, produced the product (*R*,*S*′)-**8** as white, thin, needle-like crystals in a 93% yield (93 mg, 0.306 mmol). M.p. 42.7–43.4 °C. ${\left[\alpha \right]}_{D}^{20}$ = +43.5 (c. 2.2, CH_2_Cl_2_). IR (NaCl, ν_max_/cm^−1^): 3459 (br), 3058 (vs), 2980 (vs), 2940 (vs), 2878 (vs), 1732 (vs), 1634 (s), 1606 (vs). ^1^H NMR (400 MHz, CDCl_3_) δ_H_: 7.71–7.61 (m, 3H, Nap-1,4,8), 7.38 (dd, *J* = 8.6, 1.9 Hz, 1H, Nap-3), 7.14 (dd, *J* = 8.9, 2.5 Hz, 1H, Nap-7), 7.10 (d, *J* = 2.5 Hz, 1H, Nap-5), 4.23–4.07 (m, 2H, CH_2_ *sn*-3), 3.90 (s, 3H, OCH_3_), 3.96–3.84 (m, 1H, CH *sn*-2), 3.81 (q, *J* = 7.2 Hz, 1H, C*H*CH_3_), 3.57 (m, 1H, CH_2_ *sn*-1), 3.45 (m, 1H, CH_2_ *sn*-1), 1.59 (d, *J* = 7.2 Hz, 3H, CHC*H*_3_) ppm. ^13^C{H} NMR (101 MHz, CDCl_3_) δ_C_: 175.2, 157.9, 135.4, 133.9, 129.4, 129.0, 127.4, 126.1 (2), 119.3, 105.8, 70.2, 65.7, 63.3, 55.4, 45.5, 18.5 ppm. HRMS (ESI) *m*/*z*: [M + Na]^+^ calcd for C_17_H_20_O_5_Na 327.1203; found, 327.1201.

#### 3.4.4. Synthesis of 1-[(*S*)-2-(6-Methoxynaphthalen-2-yl)]-*sn*-glycerol, (*S*,*S*′)-**8**

The same procedure was followed as described for (*R*,*S*′)-**7** using 3-*O*-benzyl-1-[(*S*)-2-(6-methoxynaphthalen-2-yl)]-*sn*-glycerol (*S*,*S*′)-**6** (126 mg, 0.319 mmol), THF (9.5 mL), n-hexane (15 mL) and Pd/C catalyst (17 mg). Purification on a 4% boric-acid-impregnated flash silica gel column using petroleum ether/ethyl acetate (2:3) as an eluent, followed by recrystallization from n-hexane, produced the product (*S,S*′)-**8** as white solid in a 99% yield (96 mg, 0.315 mmol). M.p. 58.9–59.7 °C. ${\left[\alpha \right]}_{D}^{20}$ = +34.3 (c. 1.0, CH_2_Cl_2_). IR (NaCl, ν_max_/cm^−1^): 3455 (br s), 3056 (vs), 2982 (vs), 2945 (vs), 2874 (vs), 1734 (vs), 1633 (s), 1605 (vs). ^1^H NMR (400 MHz, CDCl_3_) δ_H_: 7.71–7.61 (m, 3H, Nap-1,4,8), 7.39 (dd, *J* = 8.6, 1.5 Hz, 1H, Nap-3), 7.15 (dd, *J* = 8.9, 2.4 Hz, 1H, Nap-7), 7.12 (d, *J* = 2.4 Hz, 1H, Nap-5), 4.18 (d, *J* = 4.9 Hz, 2H, CH_2_ *sn*-1), 3.92 (s, 3H, OCH_3_), 3.92–3.88 (m, 1H, C*H*CH_3_), 3.88–3.82 (m, 1H, CH *sn*-2), 3.56 (dd, *J* = 11.1, 4.9 Hz, 1H, CH_2_ *sn*-3), 3.45 (dd, *J* = 11.1, 5.6 Hz, 1H, CH_2_ *sn*-3), 2.30–184 (m, 2H, OH), 1.59 (d, *J* = 7.2 Hz, 3H, CHC*H*_3_) ppm. ^13^C{H} NMR (101 MHz, CDCl_3_) δ_C_: 175.2, 157.9, 135.4, 133.9, 129.4, 129.1, 127.5, 126.1 (2), 119.3, 105.8, 70.2, 65.7, 63.3, 55.5, 45.5, 18.5 ppm. HRMS (ESI) *m*/*z*: [M + Na]^+^ calcd for C_17_H_20_O_5_Na 327.1203; found, 327.1212.

### 3.5. The Enzymatic Coupling of the MCFAs: Synthesis of (R,S′)-***9a***, (S,S′)-***9a***, (R,S′)-***10a***, and (S,S′)-***10a***

For synthesis of *(R*,*S*′)-**9b**–**d**, (*S*,*S*′)-**9b**–**d**, (*R*,*S*′)-**10b**–**d** and (*S*,*S*′)-**10b**–**d** see Supplementary Materials.

#### 3.5.1. Synthesis of 1-Hexanoyl-3-[(*S*)-2-(4-isobutylphenyl)propanoyl]-*sn*-glycerol, (*R*,*S*′)-**9a**

Immobilized CAL-B (18 mg) was added to a solution of 3-[(*S*)-2-(4-isobutylphenyl)propanoyl]-*sn*-glycerol (*R*,*S*′)-**7** (37 mg, 0.132 mmol), and vinyl hexanoate (21 mg, 0.145 mmol) in CH_2_Cl_2_ (3.5 mL). The resulting mixture was stirred at room temperature for 7 h. The lipase preparation was separated by filtration, and the solvent was removed in vacuo on a rotary evaporator. The concentrate was applied to a 4% boric-acid-impregnated flash silica gel chromatography using petroleum ether/ethyl acetate (7:3) as an eluent. This produced the product (*R*,*S*′)-**9a** as a colorless liquid in an 80% yield (40 mg, 0.106 mmol). ${\left[\alpha \right]}_{D}^{20}$ = +22.7 (c. 3.0, CH_2_Cl_2_). IR (NaCl, ν_max_/cm^−1^): 3321 (br s), 2956 (vs), 2931 (vs), 2870 (vs), 1740 (vs). ^1^H NMR (400 MHz, CDCl_3_) δ_H_: 7.19 (d, *J* = 8.1 Hz, 2H, Ibu-2,6), 7.10 (d, *J* = 8.1 Hz, 2H, H-4,6 Ibu), 4.21–3.94 (m, 5H, CH_2_ *sn*-1/3, CH *sn*-2), 3.74 (q, *J* = 7.2 Hz, 1H, C*H*CH_3_), 2.44 (d, *J* = 6.8 Hz, 2H, C*H*_2_CH(CH_3_)_2_), 2.34–2.27 (m, 2H, CH_2_COO SFA), 1.84 (nonet, *J* = 6.8 Hz, 1H, C*H*(CH_3_)_2_), 1.67–1.57 (m, 2H, C*H*_2_CH_2_COO), 1.50 (d, *J* = 7.2 Hz, 3H, CHC*H*_3_), 1.37–1.22 (m, 4H, CH_2_), 0.90 (t, *J* = 6.9 Hz, 3H, CH_2_C*H*_3_), 0.89 (d, *J* = 6.8 Hz, 6H, CH(C*H*_3_)_2_) ppm. ^13^C{H} NMR (101 MHz, CDCl_3_) δ_C_: 174.9 (C=O Ibu), 174.0 (C=O SFA), 140.9, 137.6, 129.6 (2), 127.2 (2), 68.5, 65.5, 65.0, 45.2 (2), 34.2, 31.4, 30.3, 24.7, 22.5 (2), 22.4, 18.5, 14.0 ppm. HRMS (ESI) *m*/*z*: [M + Na]^+^ calcd for C_22_H_34_O_5_Na 401.2298; found, 401.2300.

#### 3.5.2. Synthesis of 3-Hexanoyl-1-[(*S*)-2-(4-isobutylphenyl)propanoyl]-*sn*-glycerol, (*S*,*S*′)-**9a**

Immobilized CAL-B (17 mg) was added to a solution of 1-[(*S*)-2-(4-isobutylphenyl)propanoyl]-*sn*-glycerol (*S*,*S*′)-**7** (25 mg, 0.089 mmol), and vinyl hexanoate (14 mg, 0.098 mmol) in CH_2_Cl_2_ (2 mL). The resulting mixture was stirred at room temperature for 7 h. The lipase preparation was separated by filtration, and the solvent was removed in vacuo on a rotary evaporator. The concentrate was applied to a 4% boric-acid-impregnated flash silica gel chromatography using petroleum ether/ethyl acetate (4:1) as an eluent. This produced the product (*S*,*S*′)-**9a** as a colorless liquid in a 94% yield (32 mg, 0.085 mmol). ${\left[\alpha \right]}_{D}^{20}$ = +21.0 (c. 0.4, CH_2_Cl_2_). IR (NaCl, ν_max_/cm^−1^): 3465 (br s), 2975 (vs), 2941 (vs), 2864 (vs), 2834 (vs), 1738 (vs). ^1^H NMR (400 MHz, CDCl_3_) δ_H_: 7.20 (d, *J* = 8.1 Hz, 2H, Ibu-2,6), 7.10 (d, *J* = 8.1 Hz, 2H, H-4,6 Ibu), 4.21–3.97 (m, 5H, CH_2_ *sn*-1/3, CH *sn*-2), 3.74 (q, *J* = 7.2 Hz, 1H, C*H*CH_3_), 2.44 (d, *J* = 6.8 Hz, 2H, C*H*_2_CH(CH_3_)_2_), 2.39–2.28 (m, 1H, OH), 2.34–2.27 (m, 2H, CH_2_COO SFA), 1.84 (nonet, *J* = 6.8 Hz, 1H, C*H*(CH_3_)_2_), 1.68–1.55 (m, 2H, C*H*_2_CH_2_COO), 1.51 (d, *J* = 7.2 Hz, 3H, CHC*H*_3_), 1.38–1.24 (m, 4H, CH_2_), 0.90 (t, *J* = 6.9 Hz, 3H, CH_2_C*H*_3_), 0.89 (d, *J* = 6.8 Hz, 6H, CH(C*H*_3_)_2_) ppm. ^13^C{H} NMR (101 MHz, CDCl_3_) δ_C_: 174.9 (C=O Ibu), 174.0 (C=O SFA), 140.9, 137.6, 129.6 (2), 127.2 (2), 68.5, 65.4, 65.0, 45.2, 45.2, 34.2, 31.4, 30.3, 24.7, 22.5 (2), 22.4, 18.5, 14.0 ppm. HRMS (ESI) *m*/*z*: [M + Na]^+^ calcd for C_22_H_34_O_5_Na 401.2298; found, 401.2294.

#### 3.5.3. Synthesis of 1-Hexanoyl-3-[(*S*)-2-(6-methoxynaphthalen-2-yl)]-*sn*-glycerol, (*R*,*S*′)-**10a**

Immobilized CAL-B (15 mg) was added to a solution of 3-[(*S*)-2-(6-methoxynaphthalen-2-yl)]-*sn*-glycerol (*R*,*S*′)-**8** (27 mg, 0.089 mmol), and vinyl hexanoate (14 mg, 0.098 mmol) in CH_2_Cl_2_ (2.4 mL). The resulting mixture was stirred at room temperature for 2 h after which more CAL-B (5 mg) was added to speed up the reaction. After a further 3.5 h of reaction, TLC monitoring indicated a complete reaction. The lipase preparation was separated by filtration, and the solvent was removed in vacuo on a rotary evaporator. The concentrate was applied to a 4% boric-acid-impregnated flash silica gel chromatography using petroleum ether/ethyl acetate (7:3) as an eluent. This produced the product (*R,S*′)-**10a** as a colorless liquid in a 92% yield (33 mg, 0.082 mmol). ${\left[\alpha \right]}_{D}^{20}$ = +22.6 (c. 2.5, CH_2_Cl_2_). IR (NaCl, ν_max_/cm^−1^): 3459 (br s), 2946 (vs), 2930 (vs), 2870 (vs), 1740 (vs), 1635 (s), 1605 (vs). ^1^H NMR (400 MHz, CDCl_3_) δ_H_: 7.86–7.58 (m, 3H, Nap-1,4,8), 7.47–7.30 (m, 1H, Nap-3), 7.19–7.04 (m, 2H, Nap-5,7), 4.33–3.62 (m, 9H, CH_2_ *sn*-1/3, CH *sn*-2, OCH_3_, C*H*CH_3_), 2.24–2.19 (m, 3H, OH, CH_2_COO), 1.61–1.52 (m, 5H, C*H*_2_CH_2_COO, CHC*H*_3_), 1.32–1.23 (m, 4H, CH_2_), 0.87 (t, *J* = 6.7 Hz, 3H, CH_2_C*H*_3_) ppm. ^13^C{H} NMR (101 MHz, CDCl_3_) δ_C_: 174.3 (C=O Nap), 173.3 (C=O SFA), 157.9, 135.4, 133.9, 129.4, 129.1, 127.4, 126.10, 126.07, 119.2, 105.8, 68.4, 65.6, 65.0, 55.4, 45.4, 34.1, 31.4, 24.7, 22.4, 18.5, 14.0 ppm. HRMS (ESI) *m*/*z*: [M + Na]^+^ calcd for C_23_H_30_O_6_Na 425.1935; found, 425.1933.

#### 3.5.4. Synthesis of 3-Hexanoyl-1-[(*S*)-2-(6-methoxynaphthalen-2-yl)]-*sn*-glycerol, (*S*,*S*′)-**10a**

Immobilized CAL-B (15 mg) was added to a solution of 1-[(*S*)-2-(6-methoxynaphthalen-2-yl)]-*sn*-glycerol (*S*,*S*′)-**8** (33 mg, 0.108 mmol), and vinyl hexanoate (28 mg, 0.198 mmol) in CH_2_Cl_2_ (3 mL). The resulting mixture was stirred at room temperature for 3 h after which more CAL-B (5 mg) was added to speed up the reaction. After a further 5.5 h of reaction, TLC monitoring indicated a complete reaction. The lipase preparation was separated by filtration, and the solvent was removed in vacuo on a rotary evaporator. The concentrate was applied to a 4% boric-acid-impregnated flash silica gel chromatography using petroleum ether/ethyl acetate (7:3) as an eluent. This produced the product (*S*,*S*′)-**10a** as a colorless liquid in a 70% yield (30 mg, 0.075 mmol). ${\left[\alpha \right]}_{D}^{20}$ = +21.5 (c. 0.6, CH_2_Cl_2_). IR (NaCl, ν_max_/cm^−1^): 3466 (br s), 2969 (vs), 2972 (vs), 1735 (vs). ^1^H NMR (400 MHz, CDCl_3_) δ_H_: 7.73–7.68 (m, 2H, Nap-4,8), 7.66 (d, *J* = 1.9 Hz, 1H, Nap-1), 7.39 (dd, *J* = 8.5, 1.9 Hz, 1H, Nap-3), 7.14 (dd, *J* = 8.9, 2.5 Hz, 1H, Nap-7), 7.11 (d, *J* = 2.5 Hz, 1H, Nap-5), 4.21–3.98 (m, 5H, CH_2_ *sn*-1/3, CH *sn*-2), 3.91 (s, 3H, OCH_3_), 3.90 (q, *J* = 7.2 Hz, 1H, C*H*CH_3_), 2.28 (t, *J* = 7.6 Hz, 2H, CH_2_COO), 1.61–1.57 (m, 2H, C*H*_2_CH_2_COO), 1.59 (d, *J* = 7.2 Hz, 3H, CHC*H*_3_), 1.32–1.23 (m, 4H, CH_2_), 0.88 (t, *J* = 6.9 Hz, 3H, CH_2_C*H*_3_) ppm. ^13^C{H} NMR (101 MHz, CDCl_3_) δ_C_: 174.3 (C=O Nap), 174.8 (C=O Nap), 174.0 (C=O SFA), 157.9, 135.4, 133.9, 129.4, 129.1, 127.5, 126.2, 126.1, 119.3, 105.8, 68.5, 65.6, 65.0, 55.5, 45.5, 34.2, 31.4, 24.7, 22.4, 18.6, 14.0 ppm. HRMS (ESI) *m*/*z*: [M + Na]^+^ calcd for C_23_H_30_O_6_Na 425.1935; found, 425.1939.

### 3.6. Coupling of EPA: Synthesis of (R,S′)-***11a***, (S,S′)-***11a***, (R,S′)-***12a*** and (S,S′)-***12a***

For synthesis of *(R*,*S*′)-**11b**–**d**, (*S*,*S*′)-**11b**–**d**, (*R*,*S*′)-**12b**–**d** and (*S*,*S′*)-**12b**–**d** see Supplementary Materials.

#### 3.6.1. Synthesis of 2-[5Z,8Z,11Z,14Z,17Z)-Eicosa-5,8,11,14,17-pentaenoyl]-1-hexanoyl-3-[(*S*)-2-(4-isobutylphenyl)propanoyl]-*sn*-glycerol, (*R*,*S*′)-**11a**

To a solution of 1-hexanoyl-3-[(*S*)-2-(4-isobutylphenyl)propanoyl]-*sn*-glycerol (*R*,*S*′)-**9a** (15 mg, 0.040 mmol) and EPA as a free acid (13 mg, 0.044 mmol) in CH_2_Cl_2_ (2 mL) were added DMAP (6 mg, 0.043 mmol) and EDCI (12 mg, 0.058 mmol). The solution was stirred on a magnetic stirrer at room temperature for 23 h. The reaction was disconnected by passing the reaction mixture through a short column packed with silica gel by using Et_2_O/CH_2_Cl_2_ (1:9). The solvent was removed in vacuo on a rotary evaporator. The residue was applied to a silica gel chromatography using petroleum ether/ethyl acetate (9:1) as an eluent, which produced the product (*R*,*S*′)-**11a** as a yellow oil, in a 96% yield (26 mg, 0.039 mmol). ${\left[\alpha \right]}_{D}^{20}$ = +8.29 (c. 2.8, CH_2_Cl_2_). IR (NaCl, ν_max_/cm^−1^): 3012 (vs), 2958 (vs), 2927 (vs), 2871 (vs), 1744 (vs). ^1^H NMR (400 MHz, CDCl_3_) δ_H_: 7.18 (d, *J* = 8.1 Hz, 2H, Ibu-2,6), 7.08 (d, *J* = 8.1 Hz, 2H, Ibu-3,5), 5.40–5.28 (m, 10H, =CH), 5.23–5.17 (m, 1H, CH *sn*-2), 4.29 (dd, *J* = 11.9, 4.4 Hz, 1H, CH_2_ *sn*-1/3), 4.21 (dd, *J* = 11.9, 5.4 Hz, 1H, CH_2_ *sn*-1/3), 4.14 (dd, *J* = 11.9, 5.8 Hz, 1H, CH_2_ *sn*-1/3), 4.00 (dd, *J* = 11.9, 5.9 Hz, 1H, CH_2_ *sn*-1/3), 3.70 (q, *J* = 7.1 Hz, 1H, C*H*CH_3_), 2.89–2.77 (m, 8H, =CHC*H*_2_CH=), 2.44 (d, *J* = 7.2 Hz, 2H, C*H*_2_CH(CH_3_)_2_), 2.31–2.20 (m, 4H, CH_2_COO EPA, CH_2_COO SFA), 2.13–2.03 (m, 4H, CH_2_C*H*_2_CH= and =CHC*H*_2_CH_3_), 1.84 (nonet, *J* = 6.9 Hz, 1H, C*H*(CH_3_)_2_), 1.69–1.62 (m, 2H, C*H*_2_CH_2_COO EPA), 1.62–1.55 (m, 2H, C*H*_2_CH_2_COO SFA), 1.49 (d, *J* = 7.1 Hz, 3H, CHC*H*_3_), 1.33–1.21 (m, 4H, CH_2_), 0.97 (t, *J* = 7.5 Hz, 3H, CH_3_ EPA), 0.89 (d, *J* = 6.7 Hz, 6H, CH(C*H*_3_)_2_), 0.88 (t, *J* = 7.0 Hz, 3H, CH_3_ SFA) ppm. ^13^C{H} NMR (101 MHz, CDCl_3_) δ_C_: 174.3 (C=O Ibu), 173.3 (C=O SFA), 172.6 (C=O EPA), 140.8, 137.4, 132.2, 129.5 (2), 129.1, 129.0, 128.7, 128.5, 128.4, 128.3, 128.2, 128.0, 127.3 (2), 127.2, 69.1, 62.4, 62.1, 45.2, 45.1, 34.7, 34.1, 33.7, 31.4, 26.7, 26.4 (3), 25.8, 24.8, 24.7, 22.5 (2), 22.4, 20.7, 18.4, 14.4, 14.0 ppm. HRMS (ESI) *m*/*z*: [M + Na]^+^ calcd for C_42_H_62_O_6_Na 685.4439; found, 685.4412.

#### 3.6.2. Synthesis of 2-[5Z,8Z,11Z,14Z,17Z)-Eicosa-5,8,11,14,17-pentaenoyl]-3-hexanoyl-1-[(*S*)-2-(4-isobutylphenyl)propanoyl]-*sn*-glycerol, (*S*,*S*′)-**11a**

To a solution of 3-hexanoyl-1-[(*S*)-2-(4-isobutylphenyl)propanoyl]-*sn*-glycerol (*S*,*S*′)-**9a** (11 mg, 0.029 mmol) and EPA as a free acid (10 mg, 0.032 mmol) in CH_2_Cl_2_ (1.5 mL) were added DMAP (4 mg, 0.031 mmol) and EDCI (8 mg, 0.042 mmol). The solution was stirred on a magnetic stirrer at room temperature for 25 h. The reaction was disconnected by passing the reaction mixture through a short column packed with silica gel by using Et_2_O/CH_2_Cl_2_ (1:9). The solvent was removed in vacuo on a rotary evaporator. The residue was applied to a silica gel chromatography using petroleum ether/ethyl acetate (4:1) as an eluent, which produced the product (*S*,*S*′)-**11a** as a yellow oil, in an 89% yield (17 mg, 0.026 mmol). ${\left[\alpha \right]}_{D}^{20}$ = +8.27 (c. 2.2, CH_2_Cl_2_). IR (NaCl, ν_max_/cm^−1^): 3013 (vs), 2970 (vs), 2873 (vs), 2829 (vs), 1744 (vs). ^1^H NMR (400 MHz, CDCl_3_) δ_H_: 7.18 (d, *J* = 8.1 Hz, 2H, Ibu-2,6), 7.08 (d, *J* = 8.1 Hz, 2H, Ibu-3,5), 5.40–5.26 (m, 10H, =CH), 5.23–5.17 (m, 1H, CH *sn*-2), 4.29 (dd, *J* = 11.9, 4.3 Hz, 1H, CH_2_ *sn*-1/3), 4.19 (dd, *J* = 11.9, 4.3 Hz, 1H, CH_2_ *sn*-1/3), 4.12 (dd, *J* = 11.9, 6.1 Hz, 1H, CH_2_ *sn*-1/3), 4.05 (dd, *J* = 11.9, 5.9 Hz, 1H, CH_2_ *sn*-1/3), 3.70 (q, *J* = 7.1 Hz, 1H, C*H*CH_3_), 2.87–2.77 (m, 8H, =CHC*H*_2_CH=), 2.44 (d, *J* = 7.2 Hz, 2H, C*H*_2_CH(CH_3_)_2_), 2.37–2.20 (m, 4H, CH_2_COO EPA, CH_2_COO SFA), 2.13–2.02 (m, 4H, CH_2_C*H*_2_CH= and =CHC*H*_2_CH3), 1.85 (nonet, *J* = 6.9 Hz, 1H, C*H*(CH_3_)_2_), 1.70–1.54 (m, 4H, C*H*_2_CH_2_COO EPA, C*H*_2_CH_2_COO SFA), 1.49 (d, *J* = 7.1 Hz, 3H, CHC*H*_3_), 1.33–1.21 (m, 4H, CH_2_), 0.97 (t, *J* = 7.5 Hz, 3H, CH_3_ EPA), 0.89 (d, *J* = 6.8 Hz, 6H, CH(C*H*_3_)_2_), 0.88 (t, *J* = 7.0 Hz, 3H, CH_3_ SFA) ppm. ^13^C{H} NMR (101 MHz, CDCl_3_) δ_C_: 174.3 (C=O Ibu), 173.3 (C=O SFA), 172.7 (C=O EPA), 140.8, 137.5, 132.2, 129.5 (2), 129.1, 129.0, 128.7, 128.5, 128.4, 128.3, 128.2, 128.0, 127.3 (2), 127.2, 69.0, 62.6, 62.1, 45.2, 45.1, 34.7, 34.1, 33.7, 31.4, 26.7, 25.8 (3), 25.7, 24.9, 24.7, 22.5 (2), 22.4, 20.7, 18.4, 14.4, 14.0 ppm. HRMS (ESI) *m*/*z*: [M + Na]^+^ calcd for C_42_H_62_O_6_Na 685.4439; found, 685.4436.

#### 3.6.3. Synthesis of 2-[5Z,8Z,11Z,14Z,17Z)-Eicosa-5,8,11,14,17-pentaenoyl]-1-hexanoyl-3-[(*S*)-2-(6-methoxynaphthalen-2-yl)propanoyl]-*sn*-glycerol, (*R*,*S*′)-**12a**

To a solution of 1-hexanoyl-3-[(*S*)-2-(6-methoxynaphthalen-2-yl)propanoyl]-*sn*-glycerol (*R*,*S*′)-**10a** (10 mg, 0.025 mmol) and EPA as a free acid (8 mg, 0.027 mmol) in CH_2_Cl_2_ (1.3 mL) were added DMAP (3 mg, 0.027 mmol) and EDCI (8 mg, 0.037 mmol). The solution was stirred on a magnetic stirrer at room temperature for 24 h. The reaction was disconnected by passing the reaction mixture through a short column packed with silica gel by using Et_2_O/CH_2_Cl_2_ (1:9). The solvent was removed in vacuo on a rotary evaporator. The residue was applied to a silica gel chromatography using petroleum ether/ethyl acetate (4:1) as an eluent, which produced the product (*R*,*S*′)-**12a** as a yellow oil, in an 80% yield (14 mg, 0.020 mmol). ${\left[\alpha \right]}_{D}^{20}$ = +9.29 (c. 1.4, CH_2_Cl_2_). IR (NaCl, ν_max_/cm^−1^): 3013 (vs), 2970 (vs), 2940 (vs), 2853 (vs), 1743 (vs), 1635 (s), 1607 (vs). ^1^H NMR (400 MHz, CDCl_3_) δ_H_: 7.72–7.66 (m, 2H, Nap-4,8), 7.64 (d, *J* = 1.9 Hz, 1H, Nap-1), 7.37 (dd, *J* = 8.5, 1.9 Hz, 1H, Nap-3), 7.14 (dd, *J* = 8.9, 2.5 Hz, 1H, Nap-7), 7.10 (d, *J* = 2.5 Hz, 1H, Nap-5), 5.48–5.27 (m, 10H, =CH), 5.20 (m, 1H, CH *sn*-2), 4.30 (dd, *J* = 11.9, 4.3 Hz, 1H, CH_2_ *sn*-1/3), 4.22 (dd, *J* = 11.9, 4.4 Hz, 1H CH_2_ *sn*-1/3), 4.16 (dd, *J* = 11.9, 6.0 Hz, 1H, CH_2_ *sn*-1/3), 4.03 (dd, *J* = 11.9, 5.8 Hz, 1H, CH_2_ *sn*-1/3), 3.90 (s, 3H, OCH_3_), 3.86 (q, *J* = 7.2 Hz, 1H, C*H*CH_3_), 2.90–2.75 (m, 8H, =CHC*H*_2_CH=), 2.24 (t, *J* = 7.5 Hz, 2H, CH_2_COO EPA), 2.12–2.04 (m, 2H, CH_2_COO SFA), 2.08 (td, *J* = 7.4, 1.6 Hz, 2H, CH_2_C*H*_2_CH=), 2.05–1.96 (m, 2H, =CHC*H*_2_CH_3_), 1.83–1.75 (m, 2H, C*H*_2_CH_2_COO EPA), 1.60–1.51 (m, 5H, C*H*_2_CH_2_COO SFA and CHC*H*_3_), 1.34–1.21 (m, 4H, CH_2_), 0.98 (t, *J* = 7.5 Hz, 3H, CH_3_ EPA), 0.88 (t, *J* = 7.0 Hz, 3H, CH_3_ SFA) ppm. ^13^C{H} NMR (101 MHz, CDCl_3_) δ_C_: 174.2 (C=O Nap), 173.3 (C=O SFA), 172.6 (C=O EPA), 157.9, 135.3, 133.9, 132.2, 129.4, 129.1, 129.0, 128.7, 128.6, 128.5, 128.4, 128.3, 128.2, 128.0, 127.3, 127.2, 126.3, 126.1, 119.2, 105.7, 69.0, 62.5, 62.1, 55.4, 45.5, 34.1, 33.6, 31.4, 26.4, 25.8 (3), 25.7, 24.6, 24.2, 22.4, 20.7, 18.4, 14.4, 14.0 ppm. HRMS (ESI) *m*/*z*: [M + Na]^+^ calcd for C_43_H_58_O_7_Na 709.4075; found, 709.4059.

#### 3.6.4. Synthesis of 2-[5Z,8Z,11Z,14Z,17Z)-Eicosa-5,8,11,14,17-pentaenoyl]-3-hexanoyl-1-[(*S*)-2-(6-methoxynaphthalen-2-yl)propanoyl]-*sn*-glycerol, (*S*,*S*′)-**12a**

To a solution of 3-hexanoyl-1-[(*S*)-2-(6-methoxynaphthalen-2-yl)propanoyl]-*sn*-glycerol (*S*,*S*′)-**10a** (11 mg, 0.027 mmol) and EPA as a free acid (9 mg, 0.030 mmol) in CH_2_Cl_2_ (1.3 mL) were added DMAP (4 mg, 0.029 mmol) and EDCI (8 mg, 0.040 mmol). The solution was stirred on a magnetic stirrer at room temperature for 30 h. The reaction was disconnected by passing the reaction mixture through a short column packed with silica gel by using Et_2_O/CH_2_Cl_2_ (1:9). The solvent was removed in vacuo on a rotary evaporator. The residue was applied to a silica gel chromatography using petroleum ether/ethyl acetate (4:1) as an eluent, which produced the product (*S*,*S*′)-**12a** as a yellow oil, in a 95% yield (18 mg, 0.026 mmol). ${\left[\alpha \right]}_{D}^{20}$ = +12.4 (c. 1.5, CH_2_Cl_2_). IR (NaCl, ν_max_/cm^−1^): 3012 (vs), 2962 (vs), 2934 (vs), 2873 (vs), 1743 (vs), 1635 (s), 1607 (vs). ^1^H NMR (400 MHz, CDCl_3_) δ_H_: 7.72–7.66 (m, 2H, Nap-4,8), 7.65 (d, *J* = 1.9 Hz, 1H, Nap-1), 7.37 (dd, *J* = 8.5, 1.9 Hz, 1H, Nap-3), 7.14 (dd, *J* = 8.9, 2.5 Hz, 1H, Nap-7), 7.10 (d, *J* = 2.5 Hz, 1H, Nap-5), 5.44–5.28 (m, 10H, =CH), 5.24 (m, 1H, CH *sn*-2), 4.30 (dd, *J* = 11.9, 4.1 Hz, 1H, CH_2_ *sn*-1/3), 4.20 (dd, *J* = 11.9, 4.4 Hz, 1H CH_2_ *sn*-1/3), 4.13 (dd, *J* = 11.9, 6.3 Hz, 1H, CH_2_ *sn*-1/3), 4.06 (dd, *J* = 11.9, 5.8 Hz, 1H, CH_2_ *sn*-1/3), 3.91 (s, 3H, OCH_3_), 3.86 (q, *J* = 7.2 Hz, 1H, C*H*CH_3_), 2.89–2.75 (m, 8H, =CHC*H*_2_CH=), 2.24 (t, *J* = 7.5 Hz, 2H, CH_2_COO EPA), 2.19–2.11 (m, 2H, CH_2_COO SFA), 2.07 (td, *J* = 7.4, 1.4 Hz, 2H, CH_2_C*H*_2_CH=), 2.05–1.97 (m, 2H, =CHC*H*_2_CH_3_), 1.85–1.71 (m, 2H, C*H*_2_CH_2_COO EPA), 1.60–1.53 (m, 2H, C*H*_2_CH_2_COO SFA), 1.58 (d, *J* = 7.2 Hz, 3H, CHC*H*_3_), 1.34–1.21 (m, 4H, CH_2_), 0.98 (t, *J* = 7.5 Hz, 3H, CH_3_ EPA), 0.88 (t, *J* = 6.9 Hz, 3H, CH_3_ SFA) ppm. ^13^C{H} NMR (101 MHz, CDCl_3_) δ_C_: 174.2 (C=O Nap), 173.3 (C=O SFA), 172.6 (C=O EPA), 157.9, 135.4, 133.9, 132.2, 129.4, 129.1, 129.0, 128.44, 128.36, 128.3, 128.2, 128.0, 127.3, 127.2, 126.3, 126.1, 119.2, 105.7, 69.0, 62.7, 62.1, 55.4, 45.4, 34.1, 33.6, 31.4, 29.9, 26.6, 25.78, 25.75 (3), 25.7, 24.8, 24.6, 22.4, 20.7, 18.5, 14.4, 14.0 ppm. HRMS (ESI) *m*/*z*: [M + Na]^+^ calcd for C_43_H_58_O_7_Na 709.4059; found, 709.4059.

### 3.7. Coupling of DHA: Synthesis of (R,S′)-***13a***, (S,S′)-***13a***, (R,S′)-***14a*** and (S,S′)-***14a***

For synthesis of *(R*,*S*′)-**13b**–**d**, (*S*,*S*′)-**13b**–**d**, (*R*,*S*′)-**14b**–**d** and (*S*,*S*′)-**14b**–**d** see Supplementary Materials.

#### 3.7.1. Synthesis of 2-[4Z,7Z,10Z,13Z,16Z,19Z)-Docosa-4,7,10,13,16,19-hexaenoyl]-1-hexanoyl-3-[(*S*)-2-(4-isobutylphenyl)propanoyl]-*sn*-glycerol, (*R*,*S*′)-**13a**

To a solution of 1-hexanoyl-3-[(*S*)-2-(4-isobutylphenyl)propanoyl]-*sn*-glycerol (*R*,*S*′)-**9a** (15 mg, 0.040 mmol) and DHA as a free acid (15 mg, 0.044 mmol) in CH_2_Cl_2_ (2 mL) were added DMAP (6 mg, 0.043 mmol) and EDCI (12 mg, 0.058 mmol). The solution was stirred on a magnetic stirrer at room temperature for 23 h. The reaction was disconnected by passing the reaction mixture through a short column packed with silica gel by using Et_2_O/CH_2_Cl_2_ (1:9). The solvent was removed in vacuo on a rotary evaporator. The residue was applied to a silica gel chromatography using petroleum ether/ethyl acetate (9:1) as an eluent, which produced the product (*R*,*S*′)-**13a** as a yellow oil, in a 79% yield (22 mg, 0.032 mmol). ${\left[\alpha \right]}_{D}^{20}$ = +6.90 (c. 1.0, CH_2_Cl_2_). IR (NaCl, ν_max_/cm^−1^): 3013 (vs), 2954 (vs), 2925 (vs), 2854 (vs), 1743 (vs). ^1^H NMR (400 MHz, CDCl_3_) δ_H_: 7.18 (d, *J* = 7.8 Hz, 2H, Ibu-2,6), 7.08 (d, *J* = 7.8 Hz, 2H, Ibu-3,5), 5.50–5.24 (m, 12H, =CH), 5.23–5.17 (m, 1H, CH *sn*-2), 4.29 (dd, *J* = 11.9, 4.3 Hz, 1H, CH_2_ *sn*-1/3), 4.21 (dd, *J* = 11.9, 4.3 Hz, 1H, CH_2_ *sn*-1/3), 4.14 (dd, J = 11.9, 5.7 Hz, 1H, CH_2_ *sn*-1/3), 4.01 (dd, *J* = 11.9, 5.9 Hz, 1H, CH_2_ *sn*-1/3), 3.70 (q, *J* = 7.2 Hz, 1H, C*H*CH_3_), 2.89–2.79 (m, 10H, =CHC*H*_2_CH=), 2.44 (d, *J* = 7.2 Hz, 2H, C*H*_2_CH(CH_3_)_2_), 2.37–2.19 (m, 6H, CH_2_CH_2_COO DHA, CH_2_COO SFA), 2.08 (quint., *J* = 7.6 Hz, 2H, =CHC*H*_2_CH_3_), 1.83 (nonet, *J* = 6.8 Hz, 1H, C*H*(CH_3_)_2_), 1.62–1.56 (m, 2H, C*H*_2_CH_2_COO SFA), 1.49 (d, *J* = 7.2 Hz, 3H, CHC*H*_3_), 1.36–1.13 (m, 4H, CH_2_), 0.97 (t, *J* = 7.5 Hz, 3H, CH_3_ DHA), 0.89 (d, *J* = 6.4 Hz, 6H, CH(C*H*_3_)_2_), 0.88 (t, *J* = 7.0 Hz, 3H, CH_3_ SFA) ppm. ^13^C{H} NMR (101 MHz, CDCl_3_) δ_C_: 174.3 (C=O Ibu), 173.3 (C=O SFA), 172.9 (C=O DHA), 140.8, 137.4, 132.2, 129.5 (2), 128.7, 128.5, 128.4, 128.3, 128.2, 128.1, 128.0, 127.9, 127.8, 127.6 (2), 127.3, 127.2, 69.00, 62.5, 62.1, 45.2, 45.1, 34.2, 34.1, 31.4, 30.3, 25.8 (3), 25.7, 25.5, 24.7, 22.7, 22.5 (2), 22.4, 20.7, 18.4, 14.4, 14.0 ppm. HRMS (ESI) *m*/*z*: [M + Na]^+^ calcd for C_44_H_64_O_6_Na 711.4595; found, 711.4577.

#### 3.7.2. Synthesis of 2-[4Z,7Z,10Z,13Z,16Z,19Z)-Docosa-4,7,10,13,16,19-hexaenoyl]-3-hexanoyl-1-[(*S*)-2-(4-isobutylphenyl)propanoyl]-*sn*-glycerol, (*S*,*S*′)-**13a**

To a solution of 3-hexanoyl-1-[(*S*)-2-(4-isobutylphenyl)propanoyl]-*sn*-glycerol (*S*,*S*′)-**9a** (11 mg, 0.029 mmol) and DHA as a free acid (11 mg, 0.032 mmol) in CH_2_Cl_2_ (1.5 mL) were added DMAP (4 mg, 0.031 mmol) and EDCI (8 mg, 0.042 mmol). The solution was stirred on a magnetic stirrer at room temperature for 25 h. The reaction was disconnected by passing the reaction mixture through a short column packed with silica gel by using Et_2_O/CH_2_Cl_2_ (1:9). The solvent was removed in vacuo on a rotary evaporator. The residue was applied to a silica gel chromatography using petroleum ether/ethyl acetate (4:1) as an eluent, which produced the product (*S*,*S*′)-**13a** as a yellow oil, in a 65% yield (13 mg, 0.019 mmol). ${\left[\alpha \right]}_{D}^{20}$ = +8.22 (c. 0.9, CH_2_Cl_2_). IR (NaCl, ν_max_/cm^−1^): 3013 (vs), 2972 (vs), 2874 (vs), 1748 (vs). ^1^H NMR (400 MHz, CDCl_3_) δ_H_: 7.18 (d, *J* = 7.8 Hz, 2H, Ibu-2,6), 7.08 (d, *J* = 7.8 Hz, 2H, Ibu-3,5), 5.46–5.27 (m, 12H, =CH), 5.23 (tt, *J* = 6.0, 4.3 Hz, 1H, CH *sn*-2), 4.29 (dd, *J* = 11.9, 4.3 Hz, 1H, CH_2_ *sn*-1/3), 4.19 (dd, *J* = 11.9, 4.3 Hz, 1H, CH_2_ *sn*-1/3), 4.12 (dd, *J* = 11.9, 6.1 Hz, 1H, CH_2_ *sn*-1/3), 4.05 (dd, *J* = 11.9, 5.9 Hz, 1H, CH_2_ *sn*-1/3), 3.70 (q, *J* = 7.2 Hz, 1H, C*H*CH_3_), 2.90–2.80 (m, 10H, =CHC*H*_2_CH=), 2.44 (d, *J* = 7.2 Hz, 2H, C*H*_2_CH(CH_3_)_2_), 2.39–2.18 (m, 6H, CH_2_CH_2_COO DHA, CH_2_COO SFA), 2.13–2.03 (m, 2H, =CHC*H*_2_CH_3_), 1.84 (nonet, *J* = 6.8 Hz, 1H, C*H*(CH_3_)_2_), 1.64–1.57 (m, 2H, C*H*_2_CH_2_COO SFA), 1.49 (d, *J* = 7.2 Hz, 3H, CHC*H*_3_), 1.36–1.23 (m, 4H, CH_2_), 0.97 (t, *J* = 7.5 Hz, 3H, CH_3_ DHA), 0.89 (d, *J* = 6.4 Hz, 6H, CH(C*H*_3_)_2_), 0.88 (t, *J* = 7.0 Hz, 3H, CH_3_ SFA) ppm. ^13^C{H} NMR (101 MHz, CDCl_3_) δ_C_: 174.3 (C=O Ibu), 173.3 (C=O SFA), 172.2 (C=O DHA), 140.9, 137.5, 132.2, 129.5 (2), 128.7, 128.5, 128.4, 128.3, 128.2, 128.1, 128.0, 127.9, 127.8, 127.6 (2), 127.3, 127.2, 69.1, 62.5, 62.1, 45.2, 45.1, 34.2, 34.1, 31.4, 30.3, 25.8 (3), 25.8, 25.7, 24.7, 22.8, 22.5 (2), 22.4, 20.7, 18.4, 14.4, 14.0 ppm. HRMS (ESI) *m*/*z*: [M + Na]^+^ calcd for C_44_H_64_O_6_Na 711.4595; found, 711.4581.

#### 3.7.3. Synthesis of 2-[4Z,7Z,10Z,13Z,16Z,19Z)-Docosa-4,7,10,13,16,19-hexaenoyl]-1-hexanoyl-3-[(*S*)-2-(6-methoxynaphthalen-2-yl)propanoyl]-*sn*-glycerol, (*R*,*S*′)-**14a**

To a solution of 1-hexanoyl-3-[(*S*)-2-(6-methoxynaphthalen-2-yl)propanoyl]-*sn*-glycerol (*R*,*S*′)-**10a** (10 mg, 0.025 mmol) and DHA as a free acid (9 mg, 0.027 mmol) in CH_2_Cl_2_ (1.3 mL) were added DMAP (3 mg, 0.027 mmol) and EDCI (8 mg, 0.037 mmol). The solution was stirred on a magnetic stirrer at room temperature for 24 h. The reaction was disconnected by passing the reaction mixture through a short column packed with silica gel by using Et_2_O/CH_2_Cl_2_ (1:9). The solvent was removed in vacuo on a rotary evaporator. The residue was applied to a silica gel chromatography using petroleum ether/ethyl acetate (4:1) as an eluent, which produced the product (*R*,*S*′)-**14a** as a yellow oil, in an 84% yield (15 mg, 0.021 mmol). ${\left[\alpha \right]}_{D}^{20}$ = +8.00 (c. 1.5, CH_2_Cl_2_). IR (NaCl, ν_max_/cm^−1^): 3009 (vs), 2979 (vs), 2941 (vs), 2837 (vs), 1740 (vs), 1634 (s), 1609 (vs). ^1^H NMR (400 MHz, CDCl_3_) δ_H_: 7.72–7.66 (m, 2H, Nap-4,8), 7.65 (d, *J* = 1.9 Hz, 1H, Nap-1), 7.37 (dd, *J* = 8.5, 1.9 Hz, 1H, Nap-3), 7.14 (dd, *J* = 8.9, 2.5 Hz, 1H, Nap-7), 7.10 (d, *J* = 2.5 Hz, 1H, Nap-5), 5.44–5.24 (m, 12H, =CH), 5.21 (tt, *J* = 5.9, 4.5 Hz, 1H, CH *sn*-2), 4.30 (dd, *J* = 11.9, 4.4 Hz, 1H, CH_2_ *sn*-1/3), 4.21 (dd, *J* = 11.9, 4.4 Hz, 1H, CH_2_ *sn*-1/3), 4.16 (dd, *J* = 11.9, 5.9 Hz, 1H, CH_2_ *sn*-1/3), 4.03 (dd, *J* = 11.9, 5.9 Hz, 1H, CH_2_ *sn*-1/3), 3.91 (s, 3H, OCH_3_), 3.87 (q, *J* = 7.2 Hz, 1H, C*H*CH_3_), 2.89–2.76 (m, 10H, =CHC*H*_2_CH=), 2.31–2.16 (m, 6H, CH_2_CH_2_COO DHA, =CHC*H*_2_CH_3_), 2.13–2.03 (m, 2H, CH_2_COO SFA), 1.63–1.50 (m, 2H, C*H*_2_CH_2_COO SFA), 1.58 (d, *J* = 7.1 Hz, 3H, CHC*H*_3_), 1.33–1.20 (m, 4H, CH_2_), 0.97 (t, *J* = 7.5 Hz, 3H, CH_3_ DHA), 0.88 (t, *J* = 7.0 Hz, 3H, CH_3_ SFA) ppm. ^13^C{H} NMR (101 MHz, CDCl_3_) δ_C_: 174.2 (C=O Nap), 173.3 (C=O SFA), 172.1 (C=O DHA), 157.9, 135.3, 133.9, 132.2, 129.5, 129.4, 129.1, 128.7, 128.47 (2), 128.45, 128.4, 128.3, 128.23, 128.16, 128.0, 127.8, 127.3, 127.2, 126.3, 126.1, 119.2, 105.8, 69.2, 62.5, 62.1, 55.5, 45.5, 34.1, 34.0, 31.4, 25.8 (3), 25.73, 25.70, 24.6, 22.7, 22.4, 20.7, 18.4, 14.4, 14.0 ppm. HRMS (ESI) *m*/*z*: [M + Na]^+^ calcd for C_45_H_60_O_7_Na 735.4231; found, 735.4210.

#### 3.7.4. Synthesis of 2-[4Z,7Z,10Z,13Z,16Z,19Z)-Docosa-4,7,10,13,16,19-hexaenoyl]-3-hexanoyl-1-[(*S*)-2-(6-methoxynaphthalen-2-yl)propanoyl]-*sn*-glycerol, (*S*,*S*′)-**14a**

To a solution of 3-hexanoyl-1-[(*S*)-2-(6-methoxynaphthalen-2-yl)propanoyl]-*sn*-glycerol (*S*,*S*′)-**10a** (11 mg, 0.027 mmol) and DHA as a free acid (10 mg, 0.030 mmol) in CH_2_Cl_2_ (1.3 mL) were added DMAP (4 mg, 0.029 mmol) and EDCI (8 mg, 0.037 mmol). The solution was stirred on a magnetic stirrer at room temperature for 30 h. The reaction was disconnected by passing the reaction mixture through a short column packed with silica gel by using Et_2_O/CH_2_Cl_2_ (1:9). The solvent was removed in vacuo on a rotary evaporator. The residue was applied to a silica gel chromatography using petroleum ether/ethyl acetate (4:1) as an eluent, which produced the product (*S*,*S*′)-**14a** as a yellow oil, in a 79% yield (15 mg, 0.021 mmol). ${\left[\alpha \right]}_{D}^{20}$ = +12.6 (c. 0.5, CH_2_Cl_2_). IR (NaCl, ν_max_/cm^−1^): 3012 (vs), 2977 (vs), 2941 (vs), 2878 (vs), 2834 (vs), 1741 (vs), 1635 (s), 1607 (vs). ^1^H NMR (400 MHz, CDCl_3_) δ_H_: 7.72–7.66 (m, 2H, Nap-4,8), 7.65 (d, *J* = 1.9 Hz, 1H, Nap-1), 7.37 (dd, *J* = 8.5, 1.9 Hz, 1H, Nap-3), 7.14 (dd, *J* = 8.9, 2.5 Hz, 1H, Nap-7), 7.10 (d, *J* = 2.5 Hz, 1H, Nap-5), 5.44–5.24 (m, 12H, =CH), 5.21 (tt, *J* = 6.0, 4.4 Hz, 1H, CH *sn*-2), 4.30 (dd, *J* = 11.9, 4.2 Hz, 1H, CH_2_ *sn*-1/3), 4.19 (dd, *J* = 11.9, 4.5 Hz, 1H, CH_2_ *sn*-1/3), 4.13 (dd, *J* = 11.9, 5.9 Hz, 1H, CH_2_ *sn*-1/3), 4.03 (dd, *J* = 11.9, 5.9 Hz, 1H, CH_2_ *sn*-1/3), 3.91 (s, 3H, OCH_3_), 3.85 (q, *J* = 7.1 Hz, 1H, C*H*CH_3_), 2.89–2.77 (m, 10H, =CHC*H*_2_CH=), 2.30–2.16 (m, 6H, CH_2_CH_2_COO DHA, =CHC*H*_2_CH_3_), 2.10–2.03 (m, 2H, CH_2_COO SFA), 1.60–1.52 (m, 2H, C*H*_2_CH_2_COO SFA), 1.58 (d, *J* = 7.1 Hz, 3H, CHC*H*_3_), 1.33–1.20 (m, 4H, CH_2_), 0.97 (t, *J* = 7.5 Hz, 3H, CH_3_ DHA), 0.88 (t, *J* = 7.0 Hz, 3H, CH_3_ SFA) ppm. ^13^C{H} NMR (101 MHz, CDCl_3_) δ_C_: 174.3 (C=O Nap), 173.3 (C=O SFA), 172.2 (C=O DHA), 157.9, 135.3, 133.9, 132.2, 129.5, 129.4, 129.1, 128.7, 128.5 (2), 128.5, 128.4, 128.3, 128.2, 128.0, 127.8, 127.4, 127.2, 126.3, 126.1, 119.2, 105.8, 69.1, 62.7, 62.1, 55.5, 45.4, 34.1, 34.0, 31.4, 25.8 (3), 25.74, 25.71, 24.6, 22.7, 22.4, 20.7, 18.5, 14.4, 14.0 ppm. HRMS (ESI) *m*/*z*: [M + Na]^+^ calcd for C_45_H_60_O_7_Na 735.4231; found, 735.4217.

## 4. Conclusions

The successful asymmetric synthesis of a focused library of two 16-sample diastereomeric series of enantiostructured TAGs constituting an MCFA, a bioactive PUFA, and a potent drug has been completed by a six-step chemoenzymatic approach. All combinations of MCFAs, ranging from C6:0 to C12:0, EPA and DHA, and (*S*)-ibuprofen and (*S*)-naproxen, were prepared. They belong to the second category of enantiostructured TAG prodrugs with the MCFA and the drug attached to each of the terminal positions and the PUFA to the mid-position of the glycerol skeleton of the molecule.

All of the TAG products (32) and intermediates (24) were isolated, purified, and fully characterized and accomplished in a high chemical, regio- and stereoisomeric purity in high-to-excellent yields in most cases. They are added to the corresponding first category of 48 enantiostructured TAG molecular species that have recently been reported. It is anticipated that the resulting enantiostructured TAG library may be useful as a collection of interesting and novel types of prodrugs applicable to site-specific release profiling and bioavailability studies.

## Data Availability

The data underlying this study are available in the published article and its online Supplementary Materials.

## Supplementary Materials

The following supporting information can be downloaded at https://www.mdpi.com/article/10.3390/molecules30050991/s1, Figure S1: Progress of the lipase-promoted acylation of 1-*O*-benzyl-*sn*-glycerol with (*S*)-**3** as monitored by ^1^H NMR spectroscopy (p*S1*); Figure S2: Progress of the lipase-promoted acylation of 1-*O*-benzyl-*sn*-glycerol with (*S*)-**4** as monitored by ^1^H NMR spectroscopy (p*S2*); Figure S3: Comparison of the glyceryl proton region of the ^1^H NMR spectra for the drug adduct (*R*,*S*′)-**5** starting material and the deprotected monoacylglycerol (*R*,*S*′)-**7** (p*S3*); Figure S4: Comparison of the glyceryl proton region of the ^1^H NMR spectra for (*R*,*S*′)-**7** and (*R*,*S*′)-**9c** possessing (*S*)-ibuprofen (p*S4*); Figure S5: Comparison of the glyceryl proton region of the ^1^H NMR spectra for (*R*,*S*′)-**9c** and its acylated product (*R*,*S*′)-**11c** (p*S5*); Experimental Information: p*S6*–*S19*; NMR spectra (^1^H and ^13^C NMR for (*S*)-**3** and (*S*)-**4**; ^1^H and ^13^C NMR, ^1^H-^1^H COSY and ^13^C-^1^H HSQC shown for all compounds belonging to **5**–**8** (except (*S*,*S*′)-**8**, where there is only ^1^H NMR available), **9a** and **10a**, and (*S*,*S*′)-**11c**–(*S*,*S*′)-**14c**): p*S20*–*S52*.
