# Supplementary material for: Synthesis of Enantiostructured Triacylglycerol Prodrugs Constituting an Active Drug Located at Terminal sn-1 and sn-3 Positions of the Glycerol Backbone
Source: Molecules. 2025 Feb 21;30(5):991. doi: 10.3390/molecules30050991 (PMC11902219; doi:10.3390/molecules30050991)
Supplement: Supplementary file 1 [file molecules-30-00991-s001.zip › molecules-3430960-supplementary.pdf]

Supplementary Materials

Synthesis of Enantiostructured Triacylglycerol Prodrugs Constituting an Active Drug Located at Terminal sn-1 and sn-3 Positions of the Glycerol Backbone

Lena Rós Jónsdottir and Gudmundur G. Haraldsson *

Table of Contents

1. **Figures *S1 – S5*** *S1-S5*
2. **Experimental Information** *S6-S19*
3. **NMR Spectra** *S20-S52*

Compound (*S*)-**3** *S20*

Compound (*S*)-**4** *S21*

Compound (*R,S′*)-**5** *S22*

Compound (*S,S′*)-**5** *S24*

Compound (*R,S′*)-**6** *S26*

Compound (*S,S′*)-**6** *S28*

Compound (*R,S′*)-**7** *S30*

Compound (*S,S′*)-**7** *S32*

Compound (*R*,*S′*)-**8** *S33*

Compound (*S*,*S′*)-**8** *S35*

Compound (*R*,*S′*)-**9a** *S37*

Compound (*S*,*S′*)-**9a** *S39*

Compound (*R*,*S′*)-**10a** *S41*

Compound (*S*,*S′*)-**10a** *S43*

Compound (R,S′)-**11c** *S45*

Compound (S,S′)-**12c** *S47*

Compound (*R*,*S′*)-**13c** *S49*

Compound (*S*,*S′*)-**14c** *S51*

1. Figures *S1* – *S5*

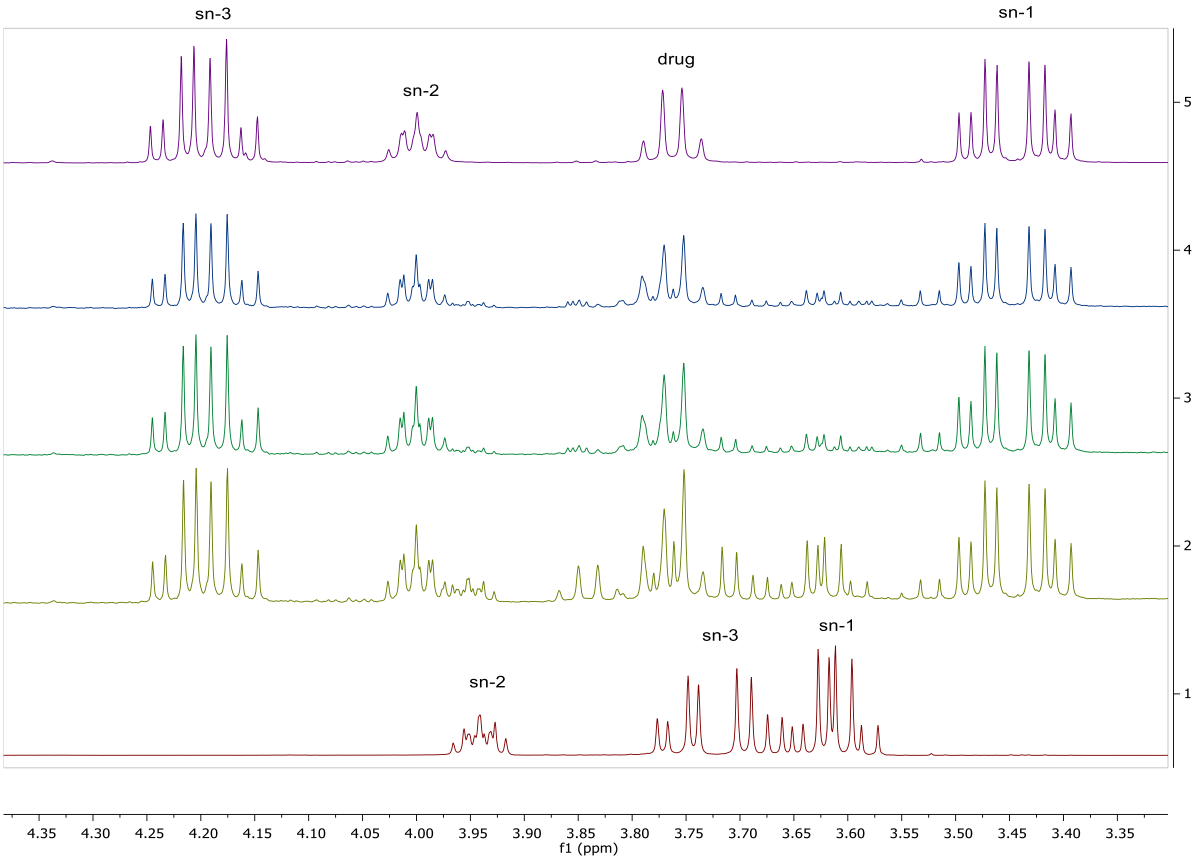

Figure *S1*. The progress of the lipase promoted acylation of 1-*O*-benzyl-*sn*-glycerol with (*S*)-**3** as monitored by ^{1}H NMR spectroscopy showing the glyceryl proton segment of the spectra as the reaction proceeded. Starting material (bottom, in red), after 6 h (second from bottom, in yellow), after 24 h (third from bottom, in green), after 30 h (second from top, in blue) and the purified product (*R*,*S*')-**5** (top, in purple).

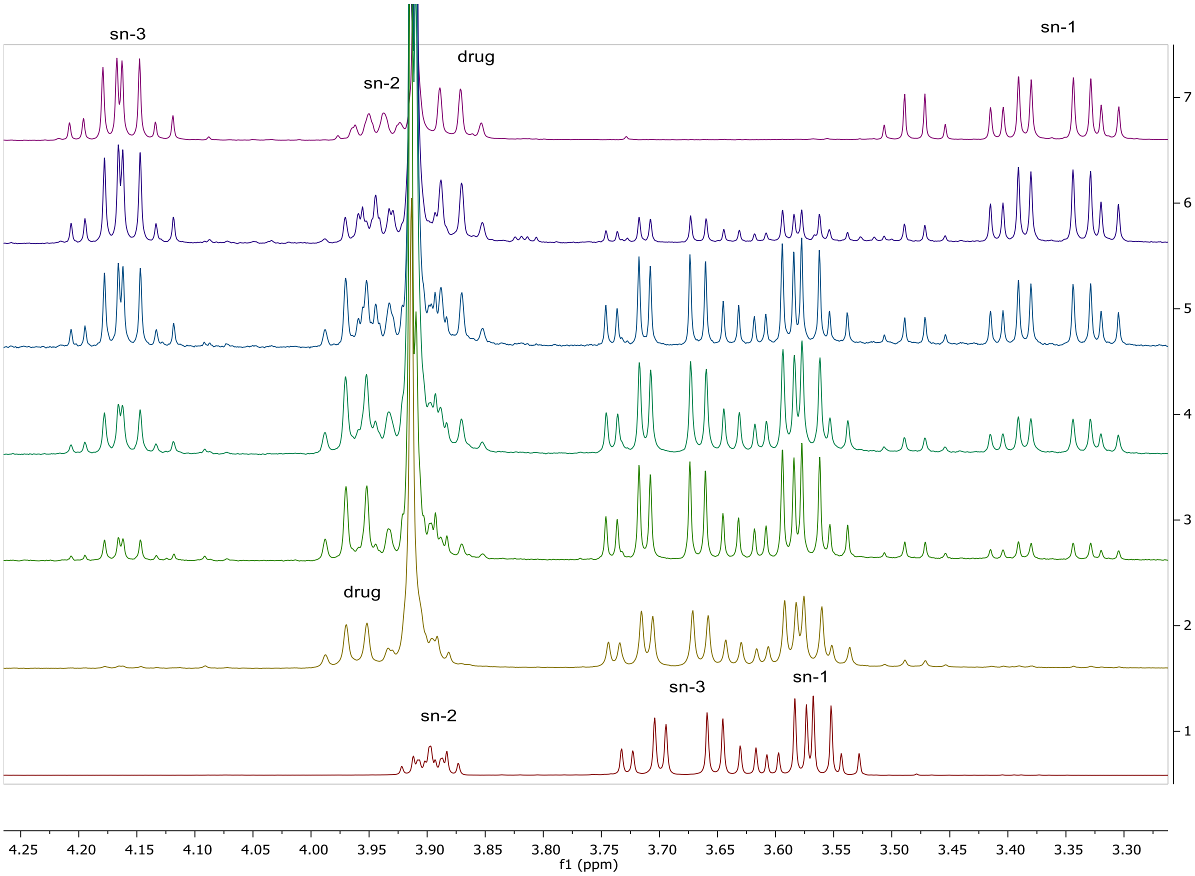

Figure ***S*2.** The progress of the lipase promoted acylation of 1-*O*-benzyl-*sn*-glycerol with (*S*)-**4** as monitored by ^{1}H NMR spectroscopy showing the glyceryl proton segment of the spectra as the reaction proceeded. Starting material (bottom, in red), after 6 h (second from bottom, in yellow), after 23 h (third from bottom, in green), after 28 h (fourth from bottom, in blue), after 31 h (third from top, purple, after 47 h (second from top, purple), and the purified product (*R*,*S*')-**6** (top, in pink).

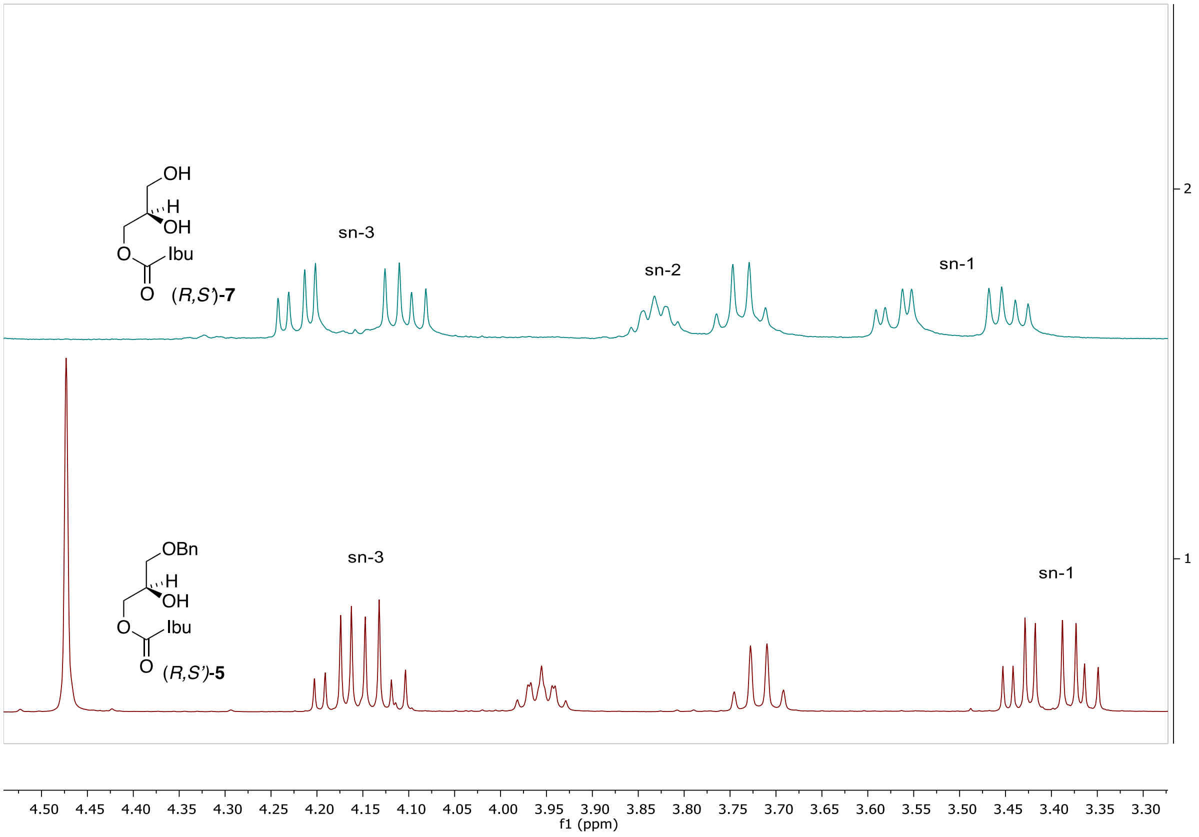

Figure *S3*. Comparison of the glyceryl proton region of the ^{1}H NMR spectra for the drug adduct (*R*,*S*′)-**5** starting material (bottom) and the deprotected monoacylglycerol (*R*,*S*′)-**7** (top).

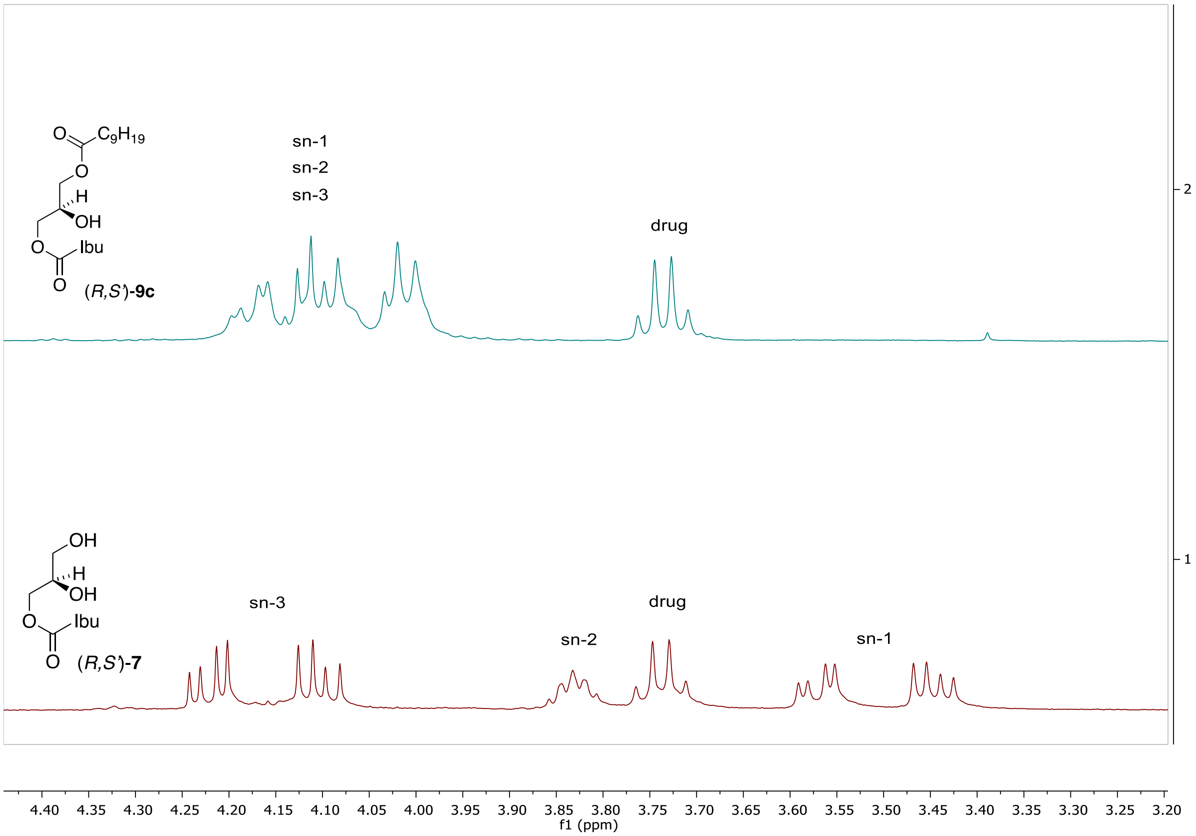

Figure *S4*. Comparison of the glyceryl proton region of the ^{1}H NMR spectra for (*R,S′*)-**7** (bottom) and (*R,S′*)-**9c** possessing (*S*)-ibuprofen (top).

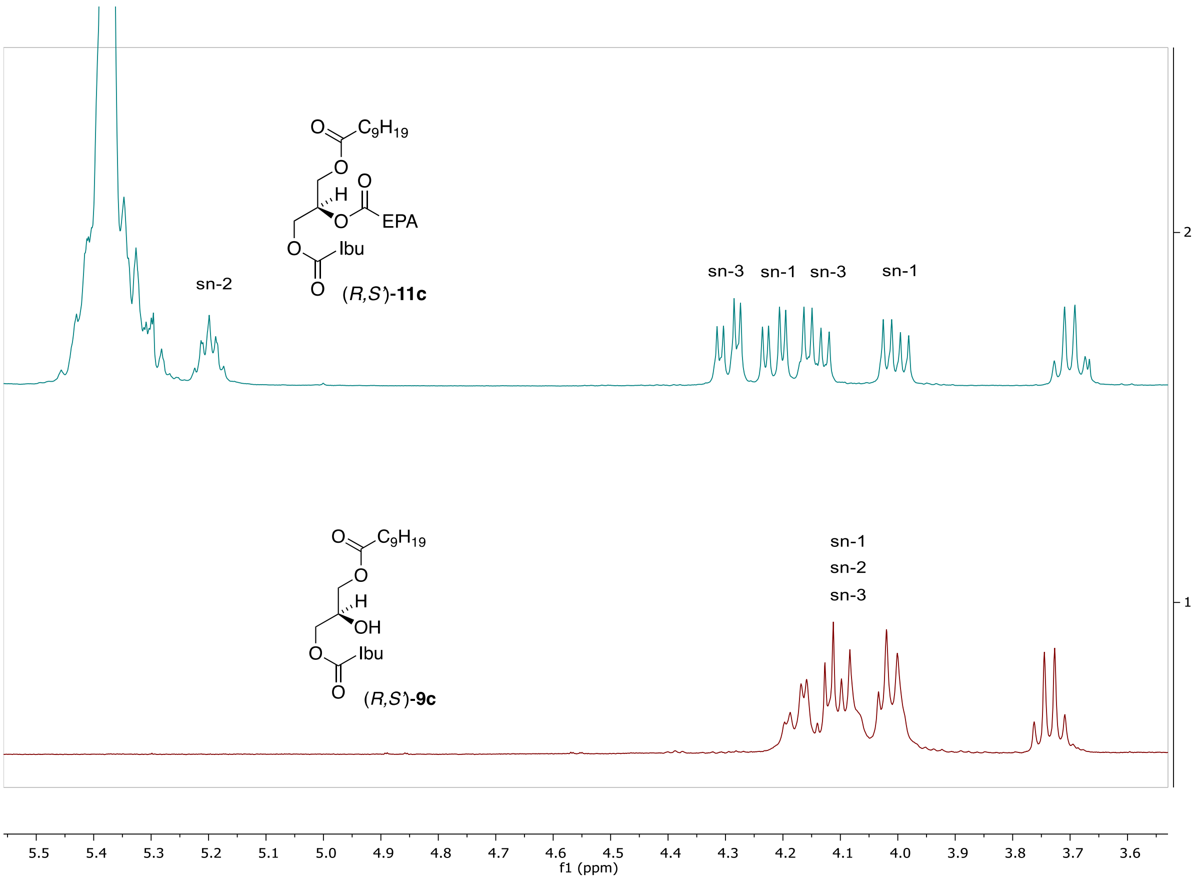

Figure S5. Comparison of the glyceryl proton region of the ^{1}H NMR spectra for (*R,S'*)-**9c** (bottom) and its acylated product (*R,S'*)-**11c** (top).

2. **Experimental Information**

*2.1. The enzymatic coupling of the saturated fatty acids: Synthesis of (R,S′)-**9b–d**, (S,S′)-**9b–d**, (R,S′)-**10b–d** and (S,S′)-**10b–d***

2.1.1. Synthesis of 3-[(*S*)-2-(4-isobutylphenyl)propanoyl]-1-octanoyl-*sn*-glycerol, (*R*,*S*')-**9b**

The same procedure was followed as described for (*R*,*S*')-**9a** using immobilized CAL-B (18 mg), 3-[(*S*)-2-(4-isobutylphenyl)propanoyl]-*sn*-glycerol (*R*,*S*')-**7** (34 mg, 0.123 mmol), vinyl octanoate (23 mg, 0.135 mmol) and CH_2Cl_2 (3.3 mL). Purification on a silica gel chromatography using pet. ether/ethyl acetate (7:3) as eluent afforded the product (*R*,*S*')-**9b** as a colorless liquid in 80% yield (40 mg, 0.098 mmol). $[\alpha]^{20}_{D}$ = +22.9 (c. 2.3, CH_2Cl_2). IR (NaCl, ν_{max} / cm^{-1}): 3227 (br s), 2984 (vs), 2926 (vs), 2856 (vs), 1743 (vs). ^1H NMR (400 MHz, $CDCl_3$) δ_H: 7.19 (d, *J*=8.1 Hz, 2H, Ibu-2,6), 7.10 (d, *J*=8.1 Hz, 2H, H-4,6 Ibu), 4.21-3.94 (m, 5H, CH_2 *sn*-1/3, CH *sn*-2), 3.74 (q, *J*=7.2 Hz, 1H, C*H*CH_3), 2.44 (d, *J*=6.8 Hz, 2H, C*H*$_2$CH(CH_3)$_2$), 2.34-2.27 (m, 2H, CH_2COO SFA), 1.84 (nonet, *J*=6.8 Hz, 1H, C*H*(CH_3)$_2$), 1.67-1.57 (m, 2H, C*H*$_2$$CH_2$COO), 1.50 (d, *J*=7.2 Hz, 3H, CHC*H*$_3$), 1.37-1.22 (m, 8H, CH_2), 0.90 (t, *J*=6.9 Hz, 3H, CH_2C*H*$_3$), 0.89 (d, *J*=6.8 Hz, 6H, CH(C*H*$_3$)$_2$) ppm. $^{13}C\{H\}$ NMR (101 MHz, $CDCl_3$) δ_C: 174.9 (C=O Ibu), 174.0 (C=O SFA), 140.9, 137.6, 129.6 (2), 127.2 (2), 68.5, 65.5, 65.0, 45.2 (2), 34.2, 31.8, 30.3, 29.22, 29.17, 24.9, 22.7, 22.5 (2), 18.5, 14.2 ppm. HRMS (ESI) *m/z*: $[M + Na]^+$ calcd for $C_{24}H_{38}O_5Na$ 429.2611; found, 429.2613.

2.1.2. Synthesis of 1-decanoyl-3-[(*S*)-2-(4-isobutylphenyl)propanoyl]-*sn*-glycerol, (*R*,*S*')-**9c**

The same procedure was followed as described for (*R*,*S*')-**9a** using immobilized CAL-B (20 mg), 3-[(*S*)-2-(4-isobutylphenyl)propanoyl]-*sn*-glycerol (*R*,*S*')-**7** (37 mg, 0.132 mmol), vinyl decanoate (29 mg, 0.145 mmol) and CH_2Cl_2 (3.3 mL). Purification on a silica gel chromatography using pet. ether/ethyl acetate (7:3) as eluent afforded the product (*R*,*S*')-**9c** as a colorless liquid in 88% yield (50 mg, 0.115 mmol). $[\alpha]^{20}_{D}$ = +25.3 (c. 2.5, CH_2Cl_2). IR (NaCl, ν_{max} / cm^{-1}): 3234 (br s), 2981 (vs), 2943 (vs), 2864 (vs), 2840 (vs), 1737 (vs). ^1H NMR (400 MHz, $CDCl_3$) δ_H: 7.20 (d, *J*=8.1 Hz, 2H, Ibu-2,6), 7.10 (d, *J*=8.1 Hz, 2H, H-4,6 Ibu), 4.23-3.94 (m, 5H, CH_2 *sn*-1/3, CH *sn*-2), 3.74 (q, *J*=7.2 Hz, 1H, C*H*CH_3), 2.45 (d, *J*=6.8 Hz, 2H, C*H*$_2$CH(CH_3)$_2$), 2.39-2.28 (m, 1H, OH), 2.30 (t, *J*=7.5 Hz, 2H, CH_2COO SFA), 1.84 (m, 1H, C*H*(CH_3)$_2$), 1.69-1.55 (m, 2H, C*H*$_2$$CH_2$COO), 1.50 (d, *J*=7.2 Hz, 3H, CHC*H*$_3$), 1.38-1.19 (m, 12H, CH_2), 0.90 (t, *J*=6.9 Hz, 3H, CH_2C*H*$_3$), 0.89 (d, *J*=6.7 Hz, 6H, CH(C*H*$_3$)$_2$) ppm. $^{13}C\{H\}$ NMR (101 MHz, $CDCl_3$) δ_C: 174.9 (C=O Ibu), 174.0 (C=O SFA), 140.9, 137.6, 129.6 (2), 127.2 (2), 68.5, 65.5, 65.0, 45.2 (2), 34.2, 31.8, 30.3, 29.4, 29.2, 29.2, 24.9, 22.7, 22.5 (2), 18.5, 14.2 ppm. HRMS (ESI) *m/z*: $[M + Na]^+$ calcd for $C_{26}H_{42}O_5Na$ 457.2924; found, 457.2929.

2.1.3. Synthesis of 1-dodecanoyl-3-[(*S*)-2-(4-isobutylphenyl)propanoyl]-*sn*-glycerol, (*R*,*S*')-**9d**

The same procedure was followed as described for (*R*,*S*')-**9a** using immobilized CAL-B (17 mg), 3-[(*S*)-2-(4-isobutylphenyl)propanoyl]-*sn*-glycerol (*R*,*S*')-**7** (30 mg, 0.108 mmol), vinyl dodecanoate (27 mg, 0.119 mmol) and CH_2Cl_2 (3 mL). Purification on a silica gel chromatography using pet. ether/ethyl acetate (7:3) as eluent, followed by recrystallization from n-hexane, afforded the product (*R*,*S*')-**9d** as a white crystalline solid in 88% yield (44 mg, 0.095 mmol). M.p. 26.5-26.8°C. $[\alpha]^{20}_{D}$ = +26.8 (c. 2.9, CH_2Cl_2). IR (NaCl, ν_{max} / cm^{-1}): 3329 (br s), 2955 (vs), 2924 (vs), 2860 (vs), 1742 (vs). ^1H NMR (400 MHz, $CDCl_3$) δ_H: 7.19 (d, *J*=8.1 Hz, 2H, Ibu-2,6), 7.10 (d, *J*=8.1 Hz, 2H, H-4,6 Ibu), 4.20-3.98 (m, 5H, CH_2 *sn*-1/3, CH *sn*-2), 3.74 (q, *J*=7.2 Hz, 1H, C*H*CH_3), 2.44 (d, *J*=6.8 Hz, 2H, C*H*$_2$CH(CH_3)$_2$), 2.33 (t, *J*=7.5 Hz, 2H, CH_2COO SFA), 1.84 (nonet, *J*=6.8 Hz, 1H, C*H*(CH_3)$_2$), 1.62 (quint., *J*=6.9 Hz, 2H, C*H*$_2$$CH_2$COO), 1.50 (d, *J*=7.2 Hz, 3H, CHC*H*$_3$), 1.37-1.22 (m, 16H, CH_2), 0.90 (t, *J*=6.9 Hz, 3H, CH_2C*H*$_3$), 0.89 (d, *J*=6.8 Hz, 6H, CH(C*H*$_3$)$_2$) ppm. $^{13}C\{H\}$ NMR (101 MHz, $CDCl_3$) δ_C: 174.9 (C=O Ibu), 174.0 (C=O SFA), 140.9, 137.6, 129.6 (2), 127.2 (2), 68.5, 65.5, 65.0, 45.2 (2), 34.2,

32.1, 30.0, 29.7, 29.6, 29.5, 29.4, 29.3, 29.2, 24.9, 22.8 (2), 22.5, 18.4, 14.2 ppm. HRMS (ESI) *m/z*: $[M + Na]^+$ calcd for $C_{28}H_{46}O_5Na$ 485.2337; found, 485.2334.

2.1.4. Synthesis of 1-[(*S*)-2-(4-isobutylphenyl)propanoyl]-3-octanoyl-*sn*-glycerol, (*S,S'*)-**9b**

The same procedure was followed as described for (*S,S'*)-**9a** using immobilized CAL-B (16 mg), 1-[(*S*)-2-(4-isobutylphenyl)propanoyl]-*sn*-glycerol (*S,S'*)-**7** (22 mg, 0.078 mmol), vinyl octanoate (15 mg, 0.086 mmol) and CH_2Cl_2 (2 mL). Purification on a silica gel chromatography using pet. ether/ethyl acetate (4:1) as eluent afforded the product (*S,S'*)-**9b** as a colorless liquid in 94% yield (30 mg, 0.074 mmol). $[\alpha]^{20}_D$ = +21.2 (c. 0.6, CH_2Cl_2). IR (NaCl, ν_{max} / cm^{-1}): 3466 (br s), 2968 (vs), 2872 (vs), 1734 (vs). ^1H NMR (400 MHz, $CDCl_3$) δ_H: 7.20 (d, *J*=8.1 Hz, 2H, Ibu-2,6), 7.10 (d, *J*=8.1 Hz, 2H, H-4,6 Ibu), 4.23-3.96 (m, 5H, CH_2 *sn*-1/3, CH *sn*-2), 3.74 (q, *J*=7.2 Hz, 1H, C*H*CH_3), 2.44 (d, *J*=6.8 Hz, 2H, C*H*$_2$CH(CH_3)$_2$), 2.39-2.28 (m, 1H, OH), 2.38-2.28 (m, 2H, CH_2COO SFA), 1.84 (nonet, *J*=6.8 Hz, 1H, C*H*(CH_3)$_2$), 1.69-1.54 (m, 2H, C*H*$_2$$CH_2$COO), 1.51 (d, *J*=7.2 Hz, 3H, CHC*H*$_3$), 1.37-1.21 (m, 8H, CH_2), 0.90 (t, *J*=6.9 Hz, 3H, CH_2C*H*$_3$), 0.89 (d, *J*=6.8 Hz, 6H, CH(C*H*$_3$)$_2$) ppm. $^{13}C\{H\}$ NMR (101 MHz, $CDCl_3$) δ_C: 174.9 (C=O Ibu), 174.0 (C=O SFA), 140.9, 137.6, 129.6 (2), 127.2 (2), 68.5, 65.5, 65.0, 45.2 (2), 34.2, 31.8, 30.3, 29.22, 29.18, 24.9, 22.7 (2), 22.5, 18.5, 14.2 ppm. HRMS (ESI) *m/z*: $[M + Na]^+$ calcd for $C_{24}H_{38}O_5Na$ 429.2611; found, 429.2611.

2.1.5. Synthesis of 3-decanoyl-1-[(*S*)-2-(4-isobutylphenyl)propanoyl]-*sn*-glycerol, (*S,S'*)-**9c**

The same procedure was followed as described for (*S,S'*)-**9a** using immobilized CAL-B (18 mg), 1-[(*S*)-2-(4-isobutylphenyl)propanoyl]-*sn*-glycerol (*S,S'*)-**7** (35 mg, 0.125 mmol), vinyl decanoate (27 mg, 0.134 mmol) and CH_2Cl_2 (2 mL). Purification on a silica gel chromatography using pet. ether/ethyl acetate (4:1) as eluent afforded the product (*S,S'*)-**9c** as a colorless liquid in 74% yield (40 mg, 0.092 mmol). $[\alpha]^{20}_D$ = +22.6 (c. 3.2, CH_2Cl_2). IR (NaCl, ν_{max} / cm^{-1}): 3325 (br s), 2970 (vs), 2941 (vs), 2864 (vs), 1742 (vs). ^1H NMR (400 MHz, $CDCl_3$) δ_H: 7.20 (d, *J*=8.1 Hz, 2H, Ibu-2,6), 7.10 (d, *J*=8.1 Hz, 2H, H-4,6 Ibu), 4.23-3.96 (m, 5H, CH_2 *sn*-1/3, CH *sn*-2), 3.74 (q, *J*=7.2 Hz, 1H, C*H*CH_3), 2.45 (d, *J*=6.8 Hz, 2H, C*H*$_2$CH(CH_3)$_2$), 2.39-2.28 (m, 1H, OH), 2.30 (t, *J*=7.5 Hz, 2H, CH_2COO SFA), 1.84 (nonet, *J*=6.9 Hz, 1H, C*H*(CH_3)$_2$), 1.69-1.55 (m, 2H, C*H*$_2$$CH_2$COO), 1.50 (d, *J*=7.2 Hz, 3H, CHC*H*$_3$), 1.38-1.19 (m, 12H, CH_2), 0.90 (t, *J*=6.9 Hz, 3H, CH_2C*H*$_3$), 0.89 (d, *J*=6.7 Hz, 6H, CH(C*H*$_3$)$_2$) ppm. $^{13}C\{H\}$ NMR (101 MHz, $CDCl_3$) δ_C: 174.9 (C=O Ibu), 174.0 (C=O SFA), 140.9, 137.6, 129.6 (2), 127.2 (2), 68.5, 65.5, 65.0, 45.2 (2), 33.6, 32.1, 30.3, 29.8, 29.6, 29.5, 29.4, 29.3, 29.2, 24.9, 22.8 (2), 22.2, 18.4, 14.3 ppm. HRMS (ESI) *m/z*: $[M + Na]^+$ calcd for $C_{26}H_{42}O_5Na$ 457.2924; found, 457.2922.

2.1.6. Synthesis of 3-dodecanoyl-1-[(*S*)-2-(4-isobutylphenyl)propanoyl]-*sn*-glycerol, (*S,S'*)-**9d**

The same procedure was followed as described for (*S,S'*)-**9a** using immobilized CAL-B (16 mg), 1-[(*S*)-2-(4-isobutylphenyl)propanoyl]-*sn*-glycerol (*S,S'*)-**7** (18 mg, 0.064 mmol), vinyl dodecanoate (16 mg, 0.070 mmol) and CH_2Cl_2 (2 mL). Purification on a silica gel chromatography using pet. ether/ethyl acetate (4:1) as eluent, followed by recrystallization from n-hexane, afforded the product (*S,S'*)-**9d** as a white solid in 97% yield (29 mg, 0.063 mmol). M.p. 41.7-42.3°C. $[\alpha]^{20}_D$ = +24.2 (c. 0.6, CH_2Cl_2). IR (NaCl, ν_{max} / cm^{-1}): 3524 (br s), 2975 (vs), 2942 (vs), 2865 (vs), 2834 (vs), 1741 (vs). ^1H NMR (400 MHz, $CDCl_3$) δ_H: 7.19 (d, *J*=8.1 Hz, 2H, Ibu-2,6), 7.10 (d, *J*=8.1 Hz, 2H, H-4,6 Ibu), 4.21-3.98 (m, 5H, CH_2 *sn*-1/3, CH *sn*-2), 3.74 (q, *J*=7.2 Hz, 1H, C*H*CH_3), 2.44 (d, *J*=6.8 Hz, 2H, C*H*$_2$CH(CH_3)$_2$), 2.30 (t, *J*=7.5 Hz, 2H, CH_2COO SFA), 1.84 (m, 1H, C*H*(CH_3)$_2$), 1.69-1.55 (m, 2H, C*H*$_2$$CH_2$COO), 1.50 (d, *J*=7.2 Hz, 3H, CHC*H*$_3$), 1.38-1.19 (m, 16H, CH_2), 0.90 (t, *J*=6.9 Hz, 3H, CH_2C*H*$_3$), 0.89 (d, *J*=6.8 Hz, 6H, CH(C*H*$_3$)$_2$) ppm. $^{13}C\{H\}$ NMR (101 MHz, $CDCl_3$) δ_C: 174.9 (C=O Ibu), 174.0 (C=O SFA), 140.9, 137.6, 129.6 (2), 127.2 (2), 68.5, 65.5, 65.0, 45.2 (2), 33.6, 32.1, 30.3, 29.8, 29.6, 29.5, 29.4, 29.3, 29.2, 24.9, 22.8 (2), 22.5, 18.4, 14.3 ppm. HRMS (ESI) *m/z*: $[M + Na]^+$ calcd for $C_{28}H_{46}O_5Na$ 485.2337; found, 485.3226.

2.1.7. Synthesis of 3-[(*S*)-2-(6-methoxynaphthalen-2-yl)]-1-octanoyl-*sn*-glycerol, (*R,S*')-**10b**

The same procedure was followed as described for (*R,S*')-**10a** using immobilized CAL-B (15 mg), 3-[(*S*)-2-(6-methoxynaphthalen-2-yl)]-*sn*-glycerol (*R,S*')-**8** (24 mg, 0.079 mmol), vinyl octanoate (15 mg, 0.087 mmol) and CH_2Cl_2 (2.1 mL). Purification on a silica gel chromatography using pet. ether/ethyl acetate (7:3) as eluent afforded the product (*R,S*')-**10b** as a colorless liquid in 91% yield (31 mg, 0.072 mmol). $[\alpha]^{20}_D$ = +38.0 (c. 2.2, CH_2Cl_2). IR (NaCl, ν_{max} / cm^{-1}): 3227 (br s), 2950 (vs), 2926 (vs), 2858 (vs), 1743 (vs), 1636 (s), 1608 (vs). ^{1}H NMR (400 MHz, $CDCl_3$) δ_H: 7.75-7.54 (m, 3H, Nap-1,4,8), 7.43-7.28 (m, 1H, Nap-3), 7.20-6.99 (m, 2H, Nap-5,7), 4.22-3.75 (m, 9H, CH_2 *sn*-1/3, CH *sn*-2, OCH_3, C*H*CH_3), 2.29-2.17 (m, 3H, OH, CH_2COO), 1.60-1.52 (m, 5H, C*H*$_2$$CH_2$COO, CHC*H*$_3$), 1.29-1.21 (m, 8H, CH_2), 0.87 (t, *J*=6.7 Hz, 3H, CH_2C*H*$_3$) ppm. ^{13}C{H} NMR (101 MHz, $CDCl_3$) δ_C: 174.3 (C=O Nap), 173.3 (C=O SFA), 157.9, 135.4, 133.9, 129.4, 129.1, 127.3, 126.3, 126.1, 119.2, 105.8, 68.4, 65.6, 65.0, 55.4, 45.5, 34.2, 31.8, 29.7, 29.0, 24.7, 22.4, 18.5, 14.0 ppm. HRMS (ESI) *m*/*z*: $[M + Na]^+$ calcd for $C_{25}H_{34}O_6Na$ 453.2248; found, 453.2250.

2.1.8. Synthesis of 1-decanoyl-3-[(*S*)-2-(6-methoxynaphthalen-2-yl)]-*sn*-glycerol, (*R,S*')-**10c**

The same procedure was followed as described for (*R,S*')-**10a** using immobilized CAL-B (15 mg), 3-[(*S*)-2-(6-methoxynaphthalen-2-yl)]-*sn*-glycerol (*R,S*')-**8** (20 mg, 0.066 mmol), vinyl decanoate (15 mg, 0.073 mmol) and CH_2Cl_2 (1.8 mL). Purification on a silica gel chromatography using pet. ether/ethyl acetate (7:3) as eluent afforded the product (*R,S*')-**10c** as a colorless liquid in 90% yield (27 mg, 0.059 mmol). $[\alpha]^{20}_D$ = +22.9 (c. 2.0, CH_2Cl_2). IR (NaCl, ν_{max} / cm^{-1}): 3350 (br s), 2981 (vs), 2942 (vs), 2864 (vs), 2840 (vs), 1737 (vs), 1633 (s), 1605 (vs). ^{1}H NMR (400 MHz, $CDCl_3$) δ_H: 7.75-7.58 (m, 3H, Nap-1,4,8), 7.42-7.30 (m, 1H, Nap-3), 7.18-6.99 (m, 2H, Nap-5,7), 4.21-3.75 (m, 9H, CH_2 *sn*-1/3, CH *sn*-2, OCH_3, C*H*CH_3), 2.33-2.13 (m, 3H, OH, CH_2COO), 1.64-1.46 (m, 5H, C*H*$_2$$CH_2$COO, CHC*H*$_3$), 1.36-1.22 (m, 12H, CH_2), 0.88 (t, *J*=6.8 Hz, 3H, CH_2C*H*$_3$) ppm. ^{13}C{H} NMR (101 MHz, $CDCl_3$) δ_C: 74.3 (C=O Nap), 173.3 (C=O SFA), 157.9, 135.4, 133.9, 129.4, 129.1, 127.3, 126.3, 126.1, 119.2, 105.8, 68.4, 65.6, 65.0, 55.4, 45.5, 34.2, 31.8, 29.5, 29.39, 29.38, 29.3, 24.7, 22.4, 18.5, 14.0 ppm. HRMS (ESI) *m*/*z*: $[M + Na]^+$ calcd for $C_{27}H_{38}O_6Na$ 481.2561; found, 481.2558.

2.1.9. Synthesis of 1-dodecanoyl-3-[(*S*)-2-(6-methoxynaphthalen-2-yl)]-*sn*-glycerol, (*R,S*')-**10d**

The same procedure was followed as described for (*R,S*')-**10a** using immobilized CAL-B (15 mg), 3-[(*S*)-2-(6-methoxynaphthalen-2-yl)]-*sn*-glycerol (*R,S*')-**8** (19 mg, 0.062 mmol), vinyl dodecanoate (16 mg, 0.068 mmol) and CH_2Cl_2 (1.6 mL). Purification on a silica gel chromatography using pet. ether/ethyl acetate (7:3) as eluent afforded the product (*R,S*')-**10d** as a colorless liquid from which upon standing crystals formed. Recrystallization from n-hexane afforded the product as a white solid in 83% yield (25 mg, 0.051 mmol). M.p. 39.8-40.6°C. $[\alpha]^{20}_D$ = +27.5 (c. 2.5, CH_2Cl_2). IR (NaCl, ν_{max} / cm^{-1}): 3227 (br s), 2954 (vs), 2924 (vs), 2854 (vs), 1742 (vs), 1635 (s), 1607 (vs). ^{1}H NMR (400 MHz, $CDCl_3$) δ_H: 7.71-7.61 (m, 3H, Nap-1,4,8), 7.40-7.30 (m, 1H, Nap-3), 7.15-7.05 (m, 2H, Nap-5,7), 4.23-3.76 (m, 9H, CH_2 *sn*-1/3, CH *sn*-2, OCH_3, C*H*CH_3), 2.33-2.16 (m, 3H, OH, CH_2COO), 1.64-1.49 (m, 5H, C*H*$_2$$CH_2$COO, CHC*H*$_3$), 1.35-1.16 (m, 16H, CH_2), 0.88 (t, *J*=6.8 Hz, 3H, CH_2C*H*$_3$) ppm. ^{13}C{H} NMR (101 MHz, $CDCl_3$) δ_C: 174.3 (C=O Nap), 173.3 (C=O SFA), 157.9, 135.4, 133.9, 129.4, 129.1, 127.3, 126.3, 126.1, 119.2, 105.8, 68.4, 65.6, 65.0, 55.4, 45.5, 34.2, 31.8, 29.5, 29.39, 29.38, 29.3, 24.7, 22.4, 18.5, 14.0 ppm. HRMS (ESI) *m*/*z*: $[M + Na]^+$ calcd for $C_{29}H_{42}O_6Na$ 509.2874; found, 509.2872.

2.1.10. Synthesis of 1-[(*S*)-2-(6-methoxynaphthalen-2-yl)]-3-octanoyl-*sn*-glycerol, (*S,S*')-**10b**

The same procedure was followed as described for (*S,S*')-**10a** using immobilized CAL-B (15 mg), 1-[(*S*)-2-(6-methoxynaphthalen-2-yl)]-*sn*-glycerol (*S,S*')-**8** (30 mg, 0.108 mmol), vinyl octanoate (19 mg, 0.109 mmol) and CH_2Cl_2 (2.7 mL). Purification on a silica gel chromatography using pet. ether/ethyl acetate (7:3) as eluent afforded the product (*S,S*')-**10b** as a colorless liquid in 70% yield (30 mg, 0.070 mmol). $[\alpha]^{20}_D$ = +23.1 (c. 1.1, CH_2Cl_2). IR (NaCl, ν_{max} / cm^{-1}): 3467 (br s), 2975 (vs), 2942 (vs), 2865 (vs), 1736 (vs). ^{1}H NMR (400 MHz, $CDCl_3$) δ_H: 7.74-7.68 (m, 2H, Nap-4,8), 7.66 (d, *J*=1.9 Hz, 1H, Nap-1), 7.39 (dd, *J*=8.5, 1.9 Hz, 1H,

Nap-3), 7.14 (dd, *J*=8.9, 2.5 Hz, 1H, Nap-7), 7.11 (d, *J*=2.5 Hz, 1H, Nap-5), 4.21-3.99 (m, 5H, CH_2 *sn*-1/3, CH *sn*-2), 4.04 (d, *J*=5.4 Hz, 1H, OH), 3.95-3.85 (m, 1H, C*H*CH_3), 3.91 (s, 3H, OCH_3), 2.28 (t, *J*=7.6 Hz, 2H, CH_2COO), 1.65-1.52 (m, 2H, C*H*$_2$$CH_2$COO), 1.59 (d, *J*=7.2 Hz, 3H, CHC*H*$_3$), 1.34-1.21 (m, 8H, CH_2), 0.88 (t, *J*=6.9 Hz, 3H, CH_2C*H*$_3$) ppm. ^{13}C{H} NMR (101 MHz, $CDCl_3$) δ_C: 174.8 (C=O Nap), 174.0 (C=O SFA), 157.9, 135.4, 133.9, 129.4, 129.1, 127.5, 126.2, 126.1, 119.3, 105.8, 68.5, 65.6, 65.0, 55.5, 45.5, 34.2, 31.8, 29.2, 29.0, 25.0, 22.7, 18.6, 14.2 ppm. HRMS (ESI) *m*/*z*: [M + Na]$^+$ calcd for $C_{25}H_{34}O_6Na$ 453.2248; found, 453.2251.

2.1.11. Synthesis of 3-decanoyl-1-[(*S*)-2-(6-methoxynaphthalen-2-yl)]-*sn*-glycerol, (*S*,*S*')-**10c**

The same procedure was followed as described for (*S*,*S*')-**10a** using immobilized CAL-B (15 mg), 1-[(*S*)-2-(6-methoxynaphthalen-2-yl)]-*sn*-glycerol (*S*,*S*')-**8** (35 mg, 0.115 mmol), vinyl decanoate (25 mg, 0.127 mmol) and CH_2Cl_2 (3 mL). Purification on a silica gel chromatography using pet. ether/ethyl acetate (7:3) as eluent afforded the product (*S*,*S*')-**10c** as a colorless liquid in 75% yield (40 mg, 0.087 mmol). $[\alpha]^{20}_D$ = +23.6 (c. 2.0, CH_2Cl_2). IR (NaCl, ν_{max} / cm^{-1}): 3456 (br s), 2964 (vs), 2873 (vs), 1740 (vs). ^{1}H NMR (400 MHz, $CDCl_3$) δ_H: 7.72-7.68 (m, 2H, Nap-4,8), 7.66 (d, *J*=1.9 Hz, 1H, Nap-1), 7.39 (dd, *J*=8.5, 1.9 Hz, 1H, Nap-3), 7.14 (dd, *J*=8.9, 2.5 Hz, 1H, Nap-7), 7.11 (d, *J*=2.5 Hz, 1H, Nap-5), 4.21-3.97 (m, 5H, CH_2 *sn*-1/3, CH *sn*-2), 3.92 (s, 3H, OCH_3), 3.90 (q, *J*=7.2 Hz, 1H, C*H*CH_3), 2.28 (t, *J*=7.6 Hz, 2H, CH_2COO), 1.61-1.56 (m, 2H, C*H*$_2$$CH_2$COO), 1.60 (d, *J*=7.2 Hz, 3H, CHC*H*$_3$), 1.34-1.21 (m, 12H, CH_2), 0.89 (t, *J*=6.9 Hz, 3H, CH_2C*H*$_3$) ppm. ^{13}C{H} NMR (101 MHz, $CDCl_3$) δ_C: 174.8 (C=O Nap), 174.0 (C=O SFA), 157.9, 135.4, 133.9, 129.4, 129.1, 127.5, 126.2, 126.1, 119.3, 105.8, 68.5, 65.6, 65.0, 55.5, 45.5, 34.2, 31.8, 29.5, 29.5, 29.3, 29.2, 25.0, 22.7, 18.6, 14.2 ppm. HRMS (ESI) *m*/*z*: [M + Na]$^+$ calcd for $C_{27}H_{38}O_6Na$ 481.2561; found, 481.2567.

2.1.12. Synthesis of 3-dodecanoyl-1-[(*S*)-2-(6-methoxynaphthalen-2-yl)]-*sn*-glycerol, (*S*,*S*')-**10d**

The same procedure was followed as described for (*S*,*S*')-**10a** using immobilized CAL-B (15 mg), 1-[(*S*)-2-(6-methoxynaphthalen-2-yl)]-*sn*-glycerol (*S*,*S*')-**8** (29 mg, 0.095 mmol), vinyl dodecanoate (24 mg, 0.105 mmol) and CH_2Cl_2 (2.6 mL). Purification on a silica gel chromatography using pet. ether/ethyl acetate (7:3) as eluent afforded the product (*S*,*S*')-**10d** as a colorless liquid from which crystals formed. Recrystallization from n-hexane afforded the product as a white solid in 91% yield (42 mg, 0.087 mmol). M.p. 38.7-39.2°C. $[\alpha]^{20}_D$ = +25.5 (c. 0.7, CH_2Cl_2). IR (NaCl, ν_{max} / cm^{-1}): 3453 (br s), 2987 (vs), 2925 (vs), 2875 (vs), 1741 (vs). ^{1}H NMR (400 MHz, $CDCl_3$) δ_H: 7.72-7.68 (m, 2H, Nap-4,8), 7.66 (d, *J*=1.9 Hz, 1H, Nap-1), 7.39 (dd, *J*=8.5, 1.9 Hz, 1H, Nap-3), 7.14 (dd, *J*=8.9, 2.5 Hz, 1H, Nap-7), 7.11 (d, *J*=2.5 Hz, 1H, Nap-5), 4.21-3.98 (m, 5H, CH_2 *sn*-1/3, CH *sn*-2), 3.91 (s, 3H, OCH_3), 3.90 (q, *J*=7.2 Hz, 1H, C*H*CH_3), 2.28 (t, *J*=7.6 Hz, 2H, CH_2COO), 1.61-1.56 (m, 2H, C*H*$_2$$CH_2$COO), 1.59 (d, *J*=7.2 Hz, 3H, CHC*H*$_3$), 1.35-1.23 (m, 16H, CH_2), 0.89 (t, *J*=6.7 Hz, 3H, CH_2C*H*$_3$) ppm. ^{13}C{H} NMR (101 MHz, $CDCl_3$) δ_C: 174.8 (C=O Nap), 174.0 (C=O SFA), 157.9, 135.4, 133.9, 129.4, 129.1, 127.5, 126.2, 126.1, 119.3, 105.8, 68.5, 65.6, 65.0, 55.5, 45.5, 34.2, 32.1, 29.8 (2), 29.6, 29.5, 29.4, 29.3, 25.0, 22.8, 18.6, 14.3 ppm. HRMS (ESI) *m*/*z*: [M + Na]$^+$ calcd for $C_{29}H_{42}O_6Na$ 509.2874; found, 509.2874.

*2.2. Coupling of EPA: Synthesis of (R,S')-**11b–d**, (S,S')-**11b–d**, (R,S')-**12b–d** and (S,S')-**12b–d***

2.2.1. Synthesis of 2-[5Z,8Z,11Z,14Z,17Z)-eicosa-5,8,11,14,17-pentaenoyl]-3-[(*S*)-2-(4-isobutylphenyl)propanoyl]-1-octanoyl-*sn*-glycerol, (*R*,*S*')-**11b**

The same procedure was followed as described for (*R*,*S*')-**11a** using 3-[(*S*)-2-(4-isobutylphenyl)-propanoyl]-1-octanoyl-*sn*-glycerol (*R*,*S*')-**9b** (15 mg, 0.037 mmol), EPA (13 mg, 0.041 mmol), CH_2Cl_2 (2 mL), DMAP (5 mg, 0.040 mmol) and EDCI (11 mg, 0.054 mmol). Purification on a silica gel chromatography using pet. ether/ethyl acetate (9:1) as eluent afforded the product (*R*,*S*')-**11b** as a yellow oil, in 88% yield (23 mg, 0.033 mmol). $[\alpha]^{20}_D$ = +12.0 (c. 2.0, CH_2Cl_2). IR (NaCl, ν_{max} / cm^{-1}): 3013 (vs), 2955 (vs), 2925 (vs), 2855 (vs), 1744 (vs). ^{1}H NMR (400 MHz, $CDCl_3$) δ_H: 7.18 (d, *J*=8.1 Hz, 2H, Ibu-2,6), 7.08 (d, *J*=8.1 Hz, 2H, Ibu-3,5), 5.40-5.28 (m, 10H, =CH), 5.23-5.17 (m, 1H, CH *sn*-2), 4.29 (dd, *J*=11.9, 4.4 Hz, 1H, CH_2 *sn*-1/3), 4.21 (dd, *J*=11.9,

4.4 Hz, 1H, CH_2 *sn*-1/3), 4.14 (dd, J=11.9, 5.8 Hz, 1H, CH_2 *sn*-1/3), 4.00 (dd, J=11.9, 5.9 Hz, 1H, CH_2 *sn*-1/3), 3.70 (q, *J*=7.1 Hz, 1H, C*H*CH_3), 2.89-2.77 (m, 8H, =CHC*H*$_2$CH=), 2.44 (d, *J*=7.2 Hz, 2H, C*H*$_2$CH(CH_3)$_2$), 2.31-2.20 (m, 4H, CH_2COO EPA, CH_2COO SFA), 2.13-2.03 (m, 4H, CH_2C*H*$_2$CH= and =CHC*H*$_2$$CH_3$), 1.84 (nonet, *J*=6.7 Hz, 1H, C*H*(CH_3)$_2$), 1.69-1.62 (m, 2H, C*H*$_2$$CH_2$COO EPA), 1.62-1.55 (m, 2H, C*H*$_2$$CH_2$COO SFA), 1.49 (d, *J*=7.1 Hz, 3H, CHC*H*$_3$), 1.33-1.21 (m, 8H, CH_2), 0.97 (t, *J*=7.5 Hz, 3H, CH_3 EPA), 0.89 (d, *J*=6.7 Hz, 6H, CH(C*H*$_3$)$_2$), 0.88 (t, *J*=7.0 Hz, 3H, CH_3 SFA) ppm. ^{13}C{H} NMR (101 MHz, $CDCl_3$) δ_C: 174.3 (C=O Ibu), 173.3 (C=O SFA), 172.6 (C=O EPA), 140.8, 137.4, 132.2, 129.5 (2), 129.1, 129.0, 128.7, 128.5, 128.4, 128.3, 128.2, 128.0, 127.3 (2), 127.2, 69.1, 62.4, 62.1, 45.2, 45.1, 34.7, 34.2, 33.7, 31.8, 30.3, 29.2, 29.1, 26.6, 26.4 (3), 25.8, 25.0, 24.8, 22.7, 22.5 (2), 20.7, 18.4, 14.4, 14.2 ppm. HRMS (ESI) *m/z*: $[M + Na]^+$ calcd for $C_{44}H_{66}O_6Na$ 713.4752; found, 713.4745.

2.2.2. Synthesis of 1-decanoyl-2-[5Z,8Z,11Z,14Z,17Z)-eicosa-5,8,11,14,17-pentaenoyl]-3-[(*S*)-2-(4-isobutyl-phenyl)propanoyl]-*sn*-glycerol, (*R,S'*)-**11c**

The same procedure was followed as described for (*R,S'*)-**11a** using 1-decanoyl-3-[(*S*)-2-(4-isobutylphenyl)propanoyl]-*sn*-glycerol (*R,S'*)-**9c** (25 mg, 0.058 mmol), EPA (19 mg, 0.064 mmol), CH_2Cl_2 (2.5 mL), DMAP (8 mg, 0.063 mmol) and EDCI (16 mg, 0.085 mmol). Purification on a silica gel chromatography using pet. ether/ethyl acetate (9:1) as eluent afforded the product (*R,S'*)-**11c** as a yellow oil, in 83% yield (35 mg, 0.049 mmol). $[\alpha]^{20}_D$ = +12.2 (c. 3.0, CH_2Cl_2). IR (NaCl, ν_{max} / cm^{-1}): 3013 (vs), 2956 (vs), 2926 (vs), 2855 (vs), 1744 (vs). ^{1}H NMR (400 MHz, $CDCl_3$) δ_H: 7.18 (d, *J*=8.1 Hz, 2H, Ibu-2,6), 7.08 (d, *J*=8.1 Hz, 2H, Ibu-3,5), 5.43-5.30 (m, 10H, =CH), 5.22-5.17 (m, 1H, CH *sn*-2), 4.29 (dd, *J*=11.9, 4.4 Hz, 1H, CH_2 *sn*-1/3), 4.21 (dd, *J*=11.9, 4.4 Hz, 1H, CH_2 *sn*-1/3), 4.14 (dd, J=11.9, 5.8 Hz, 1H, CH_2 *sn*-1/3), 4.00 (dd, J=11.9, 5.9 Hz, 1H, CH_2 *sn*-1/3), 3.70 (q, *J*=7.1 Hz, 1H, C*H*CH_3), 2.87-2.78 (m, 8H, =CHC*H*$_2$CH=), 2.44 (d, *J*=7.2 Hz, 2H, C*H*$_2$CH(CH_3)$_2$), 2.31-2.20 (m, 4H, CH_2COO EPA, CH_2COO SFA), 2.13-2.03 (m, 4H, CH_2C*H*$_2$CH= and =CHC*H*$_2$$CH_3$), 1.84 (nonet, *J*=6.9 Hz, 1H, C*H*(CH_3)$_2$), 1.69-1.61 (m, 4H, C*H*$_2$$CH_2$COO SFA and C*H*$_2$$CH_2$COO EPA), 1.49 (d, *J*=7.1 Hz, 3H, CHC*H*$_3$), 1.32-1.19 (m, 12H, CH_2), 0.97 (t, *J*=7.5 Hz, 3H, CH_3 EPA), 0.89 (d, *J*=6.7 Hz, 6H, CH(C*H*$_3$)$_2$), 0.88 (t, *J*=7.0 Hz, 3H, CH_3 SFA) ppm. ^{13}C{H} NMR (101 MHz, $CDCl_3$) δ_C: 174.3 (C=O Ibu), 173.3 (C=O SFA), 172.6 (C=O EPA), 140.8, 137.4, 132.2, 129.5 (2), 129.1, 129.0, 128.7, 128.4, 128.4, 128.3, 128.2, 128.0, 127.3 (2), 127.2, 69.1, 62.4, 62.1, 45.2, 45.1, 34.3, 34.2, 33.7, 32.0, 30.3, 29.6, 29.4, 29.3, 26.6, 25.8 (2), 25.7, 25.0, 24.8, 22.8 (2), 22.5, 20.7, 18.4, 14.4, 14.2 ppm. HRMS (ESI) *m/z*: $[M + Na]^+$ calcd for $C_{46}H_{70}O_6Na$ 741.5065; found, 741.5047.

2.2.3. Synthesis of 1-dodecanoyl-2-[5Z,8Z,11Z,14Z,17Z)-eicosa-5,8,11,14,17-pentaenoyl]-3-[(*S*)-2-(4-isobut-ylphenyl)propanoyl]-*sn*-glycerol, (*R,S'*)-**11d**

The same procedure was followed as described for (*R,S'*)-**11a** using 1-dodecanoyl-3-[(*S*)-2-(4-isobutylphenyl)propanoyl]-*sn*-glycerol (*R,S'*)-**9d** (15 mg, 0.032 mmol), EPA (11 mg, 0.035 mmol), CH_2Cl_2 (1.6 mL), DMAP (4 mg, 0.035 mmol) and EDCI (9 mg, 0.047 mmol). Purification on a silica gel chromatography using pet. ether/ethyl acetate (4:1) as eluent afforded the product (*R,S'*)-**11d** as a yellow oil, in 87% yield (20 mg, 0.028 mmol). $[\alpha]^{20}_D$ = +8.35 (c. 1.7, CH_2Cl_2). IR (NaCl, ν_{max} / cm^{-1}): 3013 (vs), 2955 (vs), 2925 (vs), 2854 (vs), 1744 (vs). ^{1}H NMR (400 MHz, $CDCl_3$) δ_H: 7.18 (d, *J*=8.1 Hz, 2H, Ibu-2,6), 7.08 (d, *J*=8.1 Hz, 2H, Ibu-3,5), 5.43-5.30 (m, 10H, =CH), 5.22-5.17 (m, 1H, CH *sn*-2), 4.29 (dd, *J*=11.9, 4.4 Hz, 1H, CH_2 *sn*-1/3), 4.21 (dd, *J*=11.9, 4.4 Hz, 1H, CH_2 *sn*-1/3), 4.14 (dd, J=11.9, 5.8 Hz, 1H, CH_2 *sn*-1/3), 4.00 (dd, J=11.9, 5.9 Hz, 1H, CH_2 *sn*-1/3), 3.70 (q, *J*=7.1 Hz, 1H, C*H*CH_3), 2.87-2.78 (m, 8H, =CHC*H*$_2$CH=), 2.44 (d, *J*=7.2 Hz, 2H, C*H*$_2$CH(CH_3)$_2$), 2.31-2.20 (m, 4H, CH_2COO EPA, CH_2COO SFA), 2.13-2.03 (m, 4H, CH_2C*H*$_2$CH= and =CHC*H*$_2$$CH_3$), 1.84 (nonet, *J*=6.8 Hz, 1H, C*H*(CH_3)$_2$), 1.69-1.61 (m, 4H, C*H*$_2$$CH_2$COO SFA, C*H*$_2$$CH_2$COO EPA), 1.49 (d, *J*=7.1 Hz, 3H, CHC*H*$_3$), 1.32-1.19 (m, 16H, CH_2), 0.97 (t, *J*=7.5 Hz, 3H, CH_3 EPA), 0.89 (d, *J*=6.6 Hz, 6H, CH(C*H*$_3$)$_2$), 0.88 (t, *J*=7.0 Hz, 3H, CH_3 SFA) ppm. ^{13}C{H} NMR (101 MHz, $CDCl_3$) δ_C: 174.3 (C=O Ibu), 173.3 (C=O SFA), 172.6 (C=O EPA), 140.8, 137.4, 132.2, 129.5 (2), 129.1, 129.0, 128.7, 128.44, 128.37, 128.3, 128.2, 128.0, 127.3 (2), 127.2, 69.1, 62.4, 62.1, 45.2, 45.1, 34.3, 34.2, 33.7, 32.0, 29.8, 29.6, 29.5, 29.4, 29.3, 26.6,

25.8 (2), 25.7, 25.0, 24.8, 22.8 (2), 22.5, 20.7, 18.4, 14.4, 14.2 ppm. HRMS (ESI) *m*/*z*: $[M + Na]^+$ calcd for $C_{48}H_{74}O_6Na$ 769.5378; found, 769.5354.

2.2.4. Synthesis of 2-[5Z,8Z,11Z,14Z,17Z)-eicosa-5,8,11,14,17-pentaenoyl]-1-[(*S*)-2-(4-isobutylphenyl)propanoyl]-3-octanoyl-*sn*-glycerol, (*S,S*')-**11b**

The same procedure was followed as described for (*S,S*')-**11a** using 1-[(*S*)-2-(4-isobutylphenyl)propanoyl]-3-octanoyl-*sn*-glycerol (*S,S*')-**9b** (12 mg, 0.030 mmol), EPA (10 mg, 0.033 mmol), CH_2Cl_2 (1.6 mL), DMAP (4 mg, 0.032 mmol) and EDCI (9 mg, 0.044 mmol). Purification on a silica gel chromatography using pet. ether/ethyl acetate (4:1) as eluent afforded the product (*S,S*')-**11b** as a yellow oil, in 90% yield (19 mg, 0.028 mmol). $[\alpha]^{20}_D$ = +8.60 (c. 1.5, CH_2Cl_2). IR (NaCl, ν_{max} / cm^{-1}): 3014 (vs), 2976 (vs), 2937 (vs), 2838 (vs), 1744 (vs). ^1H NMR (400 MHz, $CDCl_3$) δ_H: 7.17 (d, *J*=8.1 Hz, 2H, Ibu-2,6), 7.08 (d, *J*=8.1 Hz, 2H, Ibu-3,5), 5.45-5.26 (m, 10H, =CH), 5.23(m, 1H, CH *sn*-2), 4.29 (dd, *J*=11.9, 4.3 Hz, 1H, CH_2 *sn*-1/3), 4.19 (dd, *J*=11.9, 4.4 Hz, 1H, CH_2 *sn*-1/3), 4.12 (dd, J=11.9, 6.0 Hz, 1H, CH_2 *sn*-1/3), 4.05 (dd, J=11.9, 5.9 Hz, 1H, CH_2 *sn*-1/3), 3.69 (q, *J*=7.2 Hz, 1H, C*H*CH_3), 2.87-2.77 (m, 8H, =CHC*H*$_2$CH=), 2.44 (d, *J*=7.2 Hz, 2H, C*H*$_2$CH(CH_3)$_2$), 2.36-2.219 (m, 4H, CH_2COO EPA, CH_2COO SFA), 2.18-2.03 (m, 4H, CH_2C*H*$_2$CH= and =CHC*H*$_2$$CH_3$), 1.84 (nonet, *J*=6.9 Hz, 1H, C*H*(CH_3)$_2$), 1.63-1.54 (m, 4H, C*H*$_2$$CH_2$COO EPA), C*H*$_2$$CH_2$COO SFA), 1.49 (d, *J*=7.2 Hz, 3H, CHC*H*$_3$), 1.33-1.21 (m, 8H, CH_2), 0.97 (t, *J*=7.5 Hz, 3H, CH_3 EPA), 0.89 (d, *J*=6.8 Hz, 6H, CH(C*H*$_3$)$_2$), 0.88 (t, *J*=7.2 Hz, 3H, CH_3 SFA) ppm. $^{13}C\{H\}$ NMR (101 MHz, $CDCl_3$) δ_C: 174.3 (C=O Ibu), 173.3 (C=O SFA), 172.7 (C=O EPA), 140.8, 137.5, 132.2, 129.5 (2), 129.1, 129.0, 128.7, 128.5, 128.4, 128.3, 128.2, 128.0, 127.3 (2), 127.2, 69.0, 62.6, 62.1, 45.2, 45.1, 34.7, 34.2, 33.7, 31.8, 29.2, 29.1, 26.7, 25.7 (3), 25.0, 24.7, 24.2, 22.8 (2), 22.5, 20.7, 18.4, 14.4, 14.2 ppm. HRMS (ESI) *m*/*z*: $[M + Na]^+$ calcd for $C_{44}H_{66}O_6Na$ 713.4752; found, 713.4748.

2.2.5. Synthesis of 3-decanoyl-2-[5Z,8Z,11Z,14Z,17Z)-eicosa-5,8,11,14,17-pentaenoyl]-1-[(*S*)-2-(4-isobutylphenyl)propanoyl]-*sn*-glycerol, (*S,S*')-**11c**

The same procedure was followed as described for (*S,S*')-**11a** using 3-decanoyl-1-[(*S*)-2-(4-isobutylphenyl)propanoyl]-*sn*-glycerol (*S,S*')-**9c** (25 mg, 0.051 mmol), EPA (19 mg, 0.064 mmol), CH_2Cl_2 (2.5 mL), DMAP (8 mg, 0.063 mmol) and EDCI (16 mg, 0.085 mmol). Purification on a silica gel chromatography using pet. ether/ethyl acetate (4:1) as eluent afforded the product (*S,S*')-**11c** as a yellow oil, in 84% yield (31 mg, 0.043 mmol). $[\alpha]^{20}_D$ = +9.74 (c. 3.0, CH_2Cl_2). IR (NaCl, ν_{max} / cm^{-1}): 3013 (vs), 2967 (vs), 2925 (vs), 2854 (vs), 1743 (vs). ^1H NMR (400 MHz, $CDCl_3$) δ_H: 7.22-7.15 (m, 2H, Ibu-2,6), 7.10-7.04 (m, 2H, Ibu-3,5), 5.44-5.27 (m, 10H, =CH), 5.23 (m, 1H, CH *sn*-2), 4.29 (dd, *J*=11.9, 4.2 Hz, 1H, CH_2 *sn*-1/3), 4.19 (dd, *J*=11.9, 4.4 Hz, 1H, CH_2 *sn*-1/3), 4.12 (dd, J=11.9, 6.1 Hz, 1H, CH_2 *sn*-1/3), 4.04 (dd, J=11.9, 5.9 Hz, 1H, CH_2 *sn*-1/3), 3.69 (q, *J*=7.1 Hz, 1H, C*H*CH_3), 2.87-2.78 (m, 8H, =CHC*H*$_2$CH=), 2.44 (d, *J*=7.2 Hz, 2H, C*H*$_2$CH(CH_3)$_2$), 2.31-2.22 (m, 4H, CH_2COO EPA, CH_2COO SFA), 2.13-2.03 (m, 4H, CH_2C*H*$_2$CH= and =CHC*H*$_2$$CH_3$), 1.84 (nonet, *J*=6.9 Hz, 1H, C*H*(CH_3)$_2$), 1.66 (quint, *J*=7.6 Hz, 2H, C*H*$_2$$CH_2$COO SFA), 1.62-1.55 (m, 2H, C*H*$_2$$CH_2$COO EPA), 1.49 (d, *J*=7.1 Hz, 3H, CHC*H*$_3$), 1.35-1.20 (m, 12H, CH_2), 0.97 (t, *J*=7.5 Hz, 3H, CH_3 EPA), 0.89 (d, *J*=6.7 Hz, 6H, CH(C*H*$_3$)$_2$), 0.88 (t, *J*=7.0 Hz, 3H, CH_3 SFA) ppm. $^{13}C\{H\}$ NMR (101 MHz, $CDCl_3$) δ_C: 174.3 (C=O Ibu), 173.3 (C=O SFA), 172.7 (C=O EPA), 140.8, 137.4, 132.2, 129.5 (2), 129.1, 129.0, 128.7, 128.44, 128.37, 128.3, 128.2, 128.0, 127.3 (2), 127.2, 69.0, 62.6, 62.1, 45.2, 45.1, 34.2, 33.7, 32.0, 30.3, 29.6, 29.4 (2), 29.3, 26.6, 25.8 (3), 25.7, 25.0, 24.9, 22.8, 22.5 (2), 20.7, 18.4, 14.4, 14.3 ppm. HRMS (ESI) *m*/*z*: $[M + Na]^+$ calcd for $C_{46}H_{70}O_6Na$ 741.5065; found, 741.5047.

2.2.6. Synthesis of 3-dodecanoyl-2-[5Z,8Z,11Z,14Z,17Z)-eicosa-5,8,11,14,17-pentaenoyl]-1-[(*S*)-2-(4-isobutylphenyl)propanoyl]-*sn*-glycerol, (*S,S*')-**11d**

The same procedure was followed as described for (*S,S*')-**11a** using 3-dodecanoyl-1-[(*S*)-2-(4-isobutylphenyl)propanoyl]-*sn*-glycerol (*S,S*')-**9d** (11 mg, 0.024 mmol), EPA (8 mg, 0.026 mmol), CH_2Cl_2 (1.3

mL), DMAP (3 mg, 0.026 mmol) and EDCI (7 mg, 0.035 mmol). Purification on a silica gel chromatography using pet. ether/ethyl acetate (4:1) as eluent afforded the product (*S*,*S*')-**11d** as a yellow oil, in 89% yield (16 mg, 0.021 mmol). $[\alpha]^{20}_D$ = +10.4 (c. 1.0, CH_2Cl_2). IR (NaCl, ν_{max} / cm^{-1}): 3013 (vs), 2958 (vs), 2926 (vs), 2854 (vs), 1744 (vs). ^1H NMR (400 MHz, $CDCl_3$) δ_H: 7.17 (d, *J*=8.1 Hz, 2H, Ibu-2,6), 7.08 (d, *J*=8.1 Hz, 2H, Ibu-3,5), 5.45-5.26 (m, 10H, =CH), 5.23 (m, 1H, CH *sn*-2), 4.29 (dd, *J*=11.9, 4.3 Hz, 1H, CH_2 *sn*-1/3), 4.19 (dd, *J*=11.9, 4.5 Hz, 1H, CH_2 *sn*-1/3), 4.12 (dd, J=11.9, 6.0 Hz, 1H, CH_2 *sn*-1/3), 4.04 (dd, J=11.9, 5.9 Hz, 1H, CH_2 *sn*-1/3), 3.70 (q, *J*=7.2 Hz, 1H, C*H*CH_3), 2.89-2.78 (m, 8H, =CHC*H*$_2$CH=), 2.44 (d, *J*=7.2 Hz, 2H, C*H*$_2$CH(CH_3)$_2$), 2.39-2.20 (m, 4H, CH_2COO EPA, CH_2COO SFA), 2.18-2.03 (m, 4H, CH_2C*H*$_2$CH= and =CHC*H*$_2$$CH_3$), 1.84 (nonet, *J*=6.9 Hz, 1H, C*H*(CH_3)$_2$), 1.63-1.54 (m, 4H, C*H*$_2$$CH_2$COO SFA, C*H*$_2$$CH_2$COO EPA), 1.49 (d, *J*=7.2 Hz, 3H, CHC*H*$_3$), 1.33-1.21 (m, 16H, CH_2), 0.97 (t, *J*=7.5 Hz, 3H, CH_3 EPA), 0.89 (d, *J*=6.7 Hz, 6H, CH(C*H*$_3$)$_2$), 0.88 (t, *J*=7.2 Hz, 3H, CH_3 SFA) ppm. $^{13}C\{H\}$ NMR (101 MHz, $CDCl_3$) δ_C: 174.3 (C=O Ibu), 173.3 (C=O SFA), 172.7 (C=O EPA), 140.8, 137.5, 132.2, 129.5 (2), 129.1, 129.0, 128.7, 128.5, 128.4, 128.3, 128.2, 128.0, 127.3 (2), 127.2, 69.0, 62.6, 62.1, 45.2, 45.1, 34.7, 34.2, 33.7, 32.1, 30.3, 29.8, 29.6, 29.4, 29.3, 26.7, 25.8 (3), 25.7, 25.0, 24.9, 22.8 (2), 22.5, 20.7, 18.4, 14.4, 14.3 ppm. HRMS (ESI) *m*/*z*: $[M + Na]^+$ calcd for $C_{48}H_{74}O_6Na$ 769.5378; found, 769.5360.

2.2.7. Synthesis of 2-[5*Z*,8*Z*,11*Z*,14*Z*,17*Z*)-eicosa-5,8,11,14,17-pentaenoyl]-3-[(*S*)-2-(6-methoxynaphthalen-2-yl)propanoyl]-1-octanoyl-*sn*-glycerol, (*R*,*S*')-**12b**

The same procedure was followed as described for (*R*,*S*')-**12a** using 3-[(*S*)-2-(6-methoxynaphthalen-2-yl)propanoyl]-1-octanoyl-*sn*-glycerol (*R*,*S*')-**10b** (10 mg, 0.025 mmol), EPA (7 mg, 0.025 mmol), CH_2Cl_2 (1.5 mL), DMAP (3 mg, 0.025 mmol) and EDCI (7 mg, 0.034 mmol). Purification on a silica gel chromatography using pet. ether/ethyl acetate (4:1) as eluent afforded the product (*R*,*S*')-**12b** as a yellow oil, in 78% yield (13 mg, 0.018 mmol). $[\alpha]^{20}_D$ = +9.17 (c. 1.4, CH_2Cl_2). IR (NaCl, ν_{max} / cm^{-1}): 3013 (vs), 2974 (vs), 2940 (vs), 2853 (vs), 1743 (vs), 1635 (s), 1607 (vs). ^1H NMR (400 MHz, $CDCl_3$) δ_H: 7.72-7.66 (m, 2H, Nap-4,8), 7.64 (d, *J*=1.9 Hz, 1H, Nap-1), 7.37 (dd, *J*=8.5, 1.9 Hz, 1H, Nap-3), 7.14 (dd, *J*=8.9, 2.5 Hz, 1H, Nap-7), 7.10 (d, *J*=2.5 Hz, 1H, Nap-5), 5.45-5.25 (m, 10H, =CH), 5.23-5.17 (m, 1H, CH *sn*-2), 4.30 (dd, *J*=11.9, 4.3 Hz, 1H, CH_2 *sn*-1/3), 4.22 (dd, *J*=11.9, 4.4 Hz, 1H CH_2 *sn*-1/3), 4.16 (dd, *J*=11.9, 5.9 Hz, 1H, CH_2 *sn*-1/3), 4.03 (dd, J=11.9, 5.8 Hz, 1H, CH_2 *sn*-1/3), 3.91 (s, 3H, OCH_3), 3.86 (q, *J*=7.2 Hz, 1H, C*H*CH_3), 2.89-2.75 (m, 8H, =CHC*H*$_2$CH=), 2.28-2.22 (m, 2H, CH_2COO EPA), 2.20-2.11 (m, 2H, CH_2COO SFA), 2.08 (td, *J*=7.4, 1.6 Hz, 2H, CH_2C*H*$_2$CH=), 2.05-1.96 (m, 2H, =CHC*H*$_2$$CH_3$), 1.83-1.75 (m, 2H, C*H*$_2$$CH_2$COO EPA), 1.60-1.51 (m, 5H, C*H*$_2$$CH_2$COO SFA and CHC*H*$_3$), 1.34-1.21 (m, 8H, CH_2), 0.98 (t, *J*=7.5 Hz, 3H, CH_3 EPA), 0.88 (t, *J*=7.0 Hz, 3H, CH_3 SFA) ppm. $^{13}C\{H\}$ NMR (101 MHz, $CDCl_3$) δ_C: 174.2 (C=O Nap), 173.3 (C=O SFA), 172.6 (C=O EPA), 157.9, 135.3, 133.9, 132.2, 129.4, 129.01, 128.96, 128.7, 128.6, 128.44, 128.36, 128.3, 128.2, 128.0, 127.3, 127.2, 126.3, 126.1, 119.2, 105.7, 69.1, 62.5, 62.1, 55.4, 45.5, 34.1, 33.6, 31.8, 29.2, 29.0, 26.6, 25.8 (3), 25.7, 24.7, 24.2, 22.4, 20.7, 18.4, 14.4, 14.2 ppm. HRMS (ESI) *m*/*z*: $[M + Na]^+$ calcd for $C_{45}H_{62}O_7Na$ 737.4388; found, 737.4375.

2.2.8. Synthesis of 1-decanoyl-2-[5*Z*,8*Z*,11*Z*,14*Z*,17*Z*)-eicosa-5,8,11,14,17-pentaenoyl]-3-[(*S*)-2-(6-methoxy-naphthalen-2-yl)propanoyl]-*sn*-glycerol, (*R*,*S*')-**12c**

The same procedure was followed as described for (*R*,*S*')-**12a** using 1-decanoyl-3-[(*S*)-2-(6-methoxynaphthalen-2-yl)propanoyl]-*sn*-glycerol (*R*,*S*')-**10c** (10 mg, 0.022 mmol), EPA (7 mg, 0.024 mmol), CH_2Cl_2 (1.5 mL), DMAP (3 mg, 0.024 mmol) and EDCI (6 mg, 0.032 mmol). Purification on a silica gel chromatography using pet. ether/ethyl acetate (4:1) as eluent afforded the product (*R*,*S*')-**12c** as a yellow oil, in 77% yield (12 mg, 0.017 mmol). $[\alpha]^{20}_D$ = +9.62 (c. 1.3, CH_2Cl_2). IR (NaCl, ν_{max} / cm^{-1}): 3012 (vs), 2974 (vs), 2941 (vs), 2875 (vs), 1740 (vs), 1635 s), 1607 (vs). ^1H NMR (400 MHz, $CDCl_3$) δ_H: 7.72-7.66 (m, 2H, Nap-4,8), 7.64 (d, *J*=1.9 Hz, 1H, Nap-1), 7.37 (dd, *J*=8.5, 1.9 Hz, 1H, Nap-3), 7.14 (dd, *J*=8.9, 2.5 Hz, 1H, Nap-7), 7.10 (d, *J*=2.5 Hz, 1H, Nap-5), 5.48-5.27 (m, 10H, =CH), 5.23-5.17 (m, 1H, CH *sn*-2), 4.30 (dd, *J*=11.9, 4.3 Hz, 1H, CH_2 *sn*-1/3), 4.22 (dd, *J*=11.9, 4.4 Hz, 1H CH_2 *sn*-1/3), 4.16 (dd, *J*=11.9, 6.0 Hz, 1H, CH_2 *sn*-1/3), 4.03 (dd, J=11.9, 5.8 Hz, 1H, CH_2 *sn*-1/3), 3.91 (s, 3H, OCH_3), 3.86 (q, *J*=7.2 Hz, 1H, C*H*CH_3), 2.90-2.75 (m, 8H, =CHC*H*$_2$CH=), 2.24 (t, *J*=7.5 Hz, 2H, CH_2COO EPA), 2.12-2.04 (m, 2H, CH_2COO SFA), 2.08 (td, *J*=7.4, 1.6 Hz,

2H, $CH_2CH_2CH=$), 2.05-1.96 (m, 2H, $=CHCH_2CH_3$), 1.83-1.75 (m, 2H, CH_2CH_2COO EPA), 1.60-1.51 (m, 5H, CH_2CH_2COO SFA and $CHCH_3$), 1.34-1.21 (m, 12H, CH_2), 0.98 (t, *J*=7.5 Hz, 3H, CH_3 EPA), 0.88 (t, *J*=7.0 Hz, 3H, CH_3 SFA) ppm. $^{13}C\{H\}$ NMR (101 MHz, $CDCl_3$) δ_C: 174.2 (C=O Nap), 173.3 (C=O SFA), 172.6 (C=O EPA), 157.9, 135.3, 133.9, 132.2, 129.4, 129.1, 129.0, 128.7, 128.6, 128.5, 128.4, 128.3, 128.2, 128.0, 127.3, 127.2, 126.3, 126.1, 119.2, 105.7, 69.0, 62.5, 62.1, 55.4, 45.5, 34.1, 33.6, 31.4, 29.9, 29.6, 29.4, 29.4, 26.4, 25.8 (3), 25.7, 24.6, 24.2, 22.4, 20.7, 18.4, 14.1, 14.0 ppm. HRMS (ESI) *m*/*z*: $[M + Na]^+$ calcd for $C_{47}H_{66}O_7Na$ 765.4701; found, 765.4687.

2.2.9. Synthesis of 1-dodecanoyl-2-[5Z,8Z,11Z,14Z,17Z)-eicosa-5,8,11,14,17-pentaenoyl]-3-[(*S*)-2-(6-methoxynaphthalen-2-yl)propanoyl]-*sn*-glycerol, (*R*,*S*')-**12d**

The same procedure was followed as described for (*R*,*S*')-**12a** using 1-dodecanoyl-3-[(*S*)-2-(6-methoxynaphthalen-2-yl)propanoyl]-*sn*-glycerol (*R*,*S*')-**10d** (10 mg, 0.021 mmol), EPA (8 mg, 0.024 mmol), CH_2Cl_2 (3 mL), DMAP (3 mg, 0.023 mmol) and EDCI (6 mg, 0.031 mmol). Purification on a silica gel chromatography using pet. ether/ethyl acetate (4:1) as eluent afforded the product (*R*,*S*')-**12d** as a yellow oil, in 86% yield (14 mg, 0.018 mmol). $[\alpha]^{20}_D$ = +5.38 (c. 1.4, CH_2Cl_2). IR (NaCl, ν_{max} / cm^{-1}): 3013 (vs), 2972 (vs), 2942 (vs), 2884 (vs), 2833 (vs), 1741 (vs), 1636 (s), 1607 (vs). ^1H NMR (400 MHz, $CDCl_3$) δ_H: 7.72-7.66 (m, 2H, Nap-4,8), 7.64 (d, *J*=1.9 Hz, 1H, Nap-1), 7.37 (dd, *J*=8.5, 1.9 Hz, 1H, Nap-3), 7.14 (dd, *J*=8.9, 2.5 Hz, 1H, Nap-7), 7.10 (d, *J*=2.5 Hz, 1H, Nap-5), 5.48-5.27 (m, 10H, =CH), 5.23-5.17 (m, 1H, CH *sn*-2), 4.30 (dd, *J*=11.9, 4.3 Hz, 1H, CH_2 *sn*-1/3), 4.22 (dd, *J*=11.9, 4.4 Hz, 1H CH_2 *sn*-1/3), 4.16 (dd, *J*=11.9, 6.0 Hz, 1H, CH_2 *sn*-1/3), 4.03 (dd, J=11.9, 5.8 Hz, 1H, CH_2 *sn*-1/3), 3.91 (s, 3H, OCH_3), 3.86 (q, *J*=7.2 Hz, 1H, $CHCH_3$), 2.90-2.75 (m, 8H, $=CHCH_2CH=$), 2.24 (t, *J*=7.5 Hz, 2H, CH_2COO EPA), 2.12-2.04 (m, 2H, CH_2COO SFA), 2.08 (td, *J*=7.4, 1.6 Hz, 2H, $CH_2CH_2CH=$), 2.05-1.96 (m, 2H, $=CHCH_2CH_3$), 1.83-1.75 (m, 2H, CH_2CH_2COO EPA), 1.60-1.51 (m, 5H, CH_2CH_2COO SFA and $CHCH_3$), 1.34-1.21 (m, 16H, CH_2), 0.98 (t, *J*=7.5 Hz, 3H, CH_3 EPA), 0.88 (t, *J*=7.0 Hz, 3H, CH_3 SFA) ppm. $^{13}C\{H\}$ NMR (101 MHz, $CDCl_3$) δ_C: 174.2 (C=O Nap), 173.3 (C=O SFA), 172.6 (C=O EPA), 157.9, 135.3, 133.9, 132.2, 129.4, 129.1, 129.0, 128.7, 128.6, 128.5, 128.4, 128.3, 128.2, 128.0, 127.3, 127.2, 126.3, 126.1, 119.2, 105.7, 69.0, 62.5, 62.1, 55.4, 45.5, 34.1, 33.6, 31.4, 29.8, 29.6, 29.5, 29.4, 29.3, 26.4, 25.8 (3), 25.7, 24.6, 24.2, 22.4, 20.7, 18.4, 14.4, 14.3 ppm. HRMS (ESI) *m*/*z*: $[M + Na]^+$ calcd for $C_{49}H_{70}O_7Na$ 793.5014; found, 793.5007.

2.2.10. Synthesis of 2-[5Z,8Z,11Z,14Z,17Z)-eicosa-5,8,11,14,17-pentaenoyl]-1-[(*S*)-2-(6-methoxynaphthalen-2-yl)propanoyl]-3-octanoyl-*sn*-glycerol, (*S*,*S*')-**12b**

The same procedure was followed as described for (*S*,*S*')-**12a** using 1-[(*S*)-2-(6-methoxynaphthalen-2-yl)propanoyl]-3-octanoyl-*sn*-glycerol (*S*,*S*')-**10b** (11 mg, 0.026 mmol), EPA (9 mg, 0.029 mmol), CH_2Cl_2 (1.3 mL), DMAP (4 mg, 0.028 mmol) and EDCI (7 mg, 0.038 mmol). Purification on a silica gel chromatography using pet. ether/ethyl acetate (4:1) as eluent afforded the product (*S*,*S*')-**12b** as a yellow oil, in 95% yield (19 mg, 0.025 mmol). $[\alpha]^{20}_D$ = +11.2 (c. 1.5, CH_2Cl_2). IR (NaCl, ν_{max} / cm^{-1}): 3013 (vs), 2981 (vs), 2942 (vs), 2939 (vs), 2883 (vs), 1735 (vs), 1635 (s), 1607 (vs). ^1H NMR (400 MHz, $CDCl_3$) δ_H: 7.72-7.66 (m, 2H, Nap-4,8), 7.64 (d, *J*=1.9 Hz, 1H, Nap-1), 7.37 (dd, *J*=8.5, 1.9 Hz, 1H, Nap-3), 7.14 (dd, *J*=8.9, 2.5 Hz, 1H, Nap-7), 7.10 (d, *J*=2.5 Hz, 1H, Nap-5), 5.44-5.27 (m, 10H, =CH), 5.24 (m, 1H, CH *sn*-2), 4.30 (dd, *J*=11.9, 4.1 Hz, 1H, CH_2 *sn*-1/3), 4.20 (dd, *J*=11.9, 4.5 Hz, 1H CH_2 *sn*-1/3), 4.13 (dd, *J*=11.9, 6.3 Hz, 1H, CH_2 *sn*-1/3), 4.03 (dd, J=11.9, 5.8 Hz, 1H, CH_2 *sn*-1/3), 3.91 (s, 3H, OCH_3), 3.85 (q, *J*=7.2 Hz, 1H, $CHCH_3$), 2.87-2.75 (m, 8H, $=CHCH_2CH=$), 2.24 (t, *J*=7.6 Hz, 2H, CH_2COO EPA), 2.19-1.98 (m, 2H, CH_2COO SFA), 2.07 (td, *J*=7.5, 1.4 Hz, 2H, $CH_2CH_2CH=$), 2.05-1.97 (m, 2H, $=CHCH_2CH_3$), 1.83-1.75 (m, 2H, CH_2CH_2COO EPA), 1.60-1.53 (m, 2H, CH_2CH_2COO SFA), 1.58 (d, *J*=7.2 Hz, 3H, $CHCH_3$), 1.32-1.21 (m, 8H, CH_2), 0.97 (t, *J*=7.5 Hz, 3H, CH_3 EPA), 0.88 (t, *J*=6.9 Hz, 3H, CH_3 SFA) ppm. $^{13}C\{H\}$ NMR (101 MHz, $CDCl_3$) δ_C: 174.2 (C=O Nap), 173.3 (C=O SFA), 172.7 (C=O EPA), 157.9, 135.4, 133.9, 132.2, 129.4, 129.1, 129.02, 128.97, 128.7, 128.5, 128.4, 128.3, 128.2, 128.0, 127.3, 127.2, 126.3, 126.1, 119.2, 105.7, 69.0, 62.7, 62.1, 55.5, 45.4, 34.1, 33.6, 31.8, 29.2, 29.0, 26.6, 25.8, 25.8 (2), 25.7, 25.0, 24.8, 22.7, 20.7, 18.5, 14.4, 14.2 ppm. HRMS (ESI) *m*/*z*: $[M + Na]^+$ calcd for $C_{45}H_{62}O_7Na$ 737.4388; found, 737.4381.

2.2.11. Synthesis of 3-decanoyl-2-[5Z,8Z,11Z,14Z,17Z)-eicosa-5,8,11,14,17-pentaenoyl]-1-[(*S*)-2-(6-methoxynaphthalen-2-yl)propanoyl]-*sn*-glycerol, (*S*,*S*')-**12c**

The same procedure was followed as described for (*S*,*S*')-**12a** using 3-decanoyl-1-[(*S*)-2-(6-methoxynaphthalen-2-yl)propanoyl]-*sn*-glycerol (*S*,*S*')-**10c** (23 mg, 0.056 mmol), EPA (18 mg, 0.061 mmol), CH_2Cl_2 (2.5 mL), DMAP (7 mg, 0.060 mmol) and EDCI (15 mg, 0.080 mmol). Purification on a silica gel chromatography using pet. ether/ethyl acetate (4:1) as eluent afforded the product (*S*,*S*')-**12c** as a yellow oil, in 86% yield (31 mg, 0.043 mmol). $[\alpha]^{20}_D$ = +10.5 (c. 2.7, CH_2Cl_2). IR (NaCl, ν_{max} / cm^{-1}): 3012 (vs), 2947 (vs), 2941 (vs), 2875 (vs), 1740 (vs), 1635 s), 1607 (vs). ^{1}H NMR (400 MHz, $CDCl_3$) δ_H: 7.72-7.66 (m, 2H, Nap-4,8), 7.64 (d, *J*=1.7 Hz, 1H, Nap-1), 7.37 (dd, *J*=8.4, 1.7 Hz, 1H, Nap-3), 7.13 (dd, *J*=8.9, 2.4 Hz, 1H, Nap-7), 7.10 (d, *J*=2.4 Hz, 1H, Nap-5), 5.43-5.27 (m, 10H, =CH), 5.27-5.20 (m, 1H, CH *sn*-2), 4.30 (dd, *J*=11.9, 4.1 Hz, 1H, CH_2 *sn*-1/3), 4.18 (dd, *J*=11.9, 4.5 Hz, 1H CH_2 *sn*-1/3), 4.13 (dd, *J*=11.9, 6.2 Hz, 1H, CH_2 *sn*-1/3), 4.06 (dd, J=11.9, 5.7 Hz, 1H, CH_2 *sn*-1/3), 3.91 (s, 3H, OCH_3), 3.85 (q, *J*=7.2 Hz, 1H, C*H*CH_3), 2.88-2.77 (m, 8H, =CHC*H*$_2$CH=), 2.32-2.16 (m, 6H, CH_2COO EPA), CH_2COO SFA, CH_2C*H*$_2$CH=), 2.12-2.03 (m, 2H, =CHC*H*$_2$$CH_3$), 1.83-1.75 (m, 2H, C*H*$_2$$CH_2$COO EPA), 1.60-1.53 (m, 2H, C*H*$_2$$CH_2$COO SFA), 1.58 (d, *J*=7.2 Hz, 3H, CHC*H*$_3$), 1.32-1.23 (m, 12H, CH_2), 0.97 (t, *J*=7.5 Hz, 3H, CH_3 EPA), 0.88 (t, *J*=6.8 Hz, 3H, CH_3 SFA) ppm. ^{13}C{H} NMR (101 MHz, $CDCl_3$) δ_C: 174.2 (C=O Nap), 173.3 (C=O SFA), 172.7 (C=O EPA), 157.9, 135.4, 133.9, 132.2, 129.4, 129.1, 129.02, 128.97, 128.7, 128.5, 128.4, 128.3, 128.2, 128.0, 127.3, 127.2, 126.3, 126.1, 119.2, 105.7, 69.0, 62.7, 62.1, 55.5, 45.4, 34.1, 33.6, 31.8, 29.2, 29.0, 29.0, 26.6, 25.8, 25.8 (3), 25.7, 25.0, 24.8, 22.7, 20.7, 18.5, 14.4, 14.2 ppm. HRMS (ESI) *m*/*z*: [M + Na]$^+$ calcd for $C_{47}H_{66}O_7Na$ 765.4701; found, 765.4692.

2.2.12. Synthesis of 3-dodecanoyl-2-[5Z,8Z,11Z,14Z,17Z)-eicosa-5,8,11,14,17-pentaenoyl]-1-[(*S*)-2-(6-methoxynaphthalen-2-yl)propanoyl]-*sn*-glycerol, (*S*,*S*')-**12d**

The same procedure was followed as described for (*S*,*S*')-**12a** using 3-dodecanoyl-1-[(*S*)-2-(6-methoxynaphthalen-2-yl)propanoyl]-*sn*-glycerol (*S*,*S*')-**10d** (23 mg, 0.056 mmol), EPA (18 mg, 0.061 mmol), CH_2Cl_2 (2.5 mL), DMAP (7 mg, 0.060 mmol) and EDCI (15 mg, 0.080 mmol). Purification on a silica gel chromatography using pet. ether/ethyl acetate (4:1) as eluent afforded the product (*S*,*S*')-**12d** as a yellow oil, in 86% yield (31 mg, 0.043 mmol). $[\alpha]^{20}_D$ = +9.60 (c. 1.8, CH_2Cl_2). IR (NaCl, ν_{max} / cm^{-1}): 3012 (vs), 2968 (vs), 2938 (vs), 2854 (vs), 1740 (vs), 1635 (s), 1607 (vs). ^{1}H NMR (400 MHz, $CDCl_3$) δ_H: 7.72-7.66 (m, 2H, Nap-4,8), 7.64 (d, *J*=1.9 Hz, 1H, Nap-1), 7.37 (dd, *J*=8.5, 1.9 Hz, 1H, Nap-3), 7.14 (dd, *J*=8.9, 2.5 Hz, 1H, Nap-7), 7.10 (d, *J*=2.5 Hz, 1H, Nap-5), 5.43-5.27 (m, 10H, =CH), 5.27-5.20 (m, 1H, CH *sn*-2), 4.30 (dd, *J*=11.9, 4.1 Hz, 1H, CH_2 *sn*-1/3), 4.19 (dd, *J*=11.9, 4.5 Hz, 1H CH_2 *sn*-1/3), 4.13 (dd, *J*=11.9, 6.3 Hz, 1H, CH_2 *sn*-1/3), 4.06 (dd, J=11.9, 5.8 Hz, 1H, CH_2 *sn*-1/3), 3.91 (s, 3H, OCH_3), 3.87-2.75 (m, 1H, C*H*CH_3), 2.90-2.75 (m, 8H, =CHC*H*$_2$CH=), 2.24 (t, *J*=7.5 Hz, 2H, CH_2COO EPA), 2.21-1.98 (m, 4H, CH_2COO SFA), CH_2C*H*$_2$CH=), 2.12-2.03 (m, 2H, =CHC*H*$_2$$CH_3$), 1.83-1.75 (m, 2H, C*H*$_2$$CH_2$COO EPA), 1.60-1.52 (m, 2H, C*H*$_2$$CH_2$COO SFA), 1.58 (d, *J*=7.2 Hz, 3H,CHC*H*$_3$), 1.32-1.23 (m, 16H, CH_2), 0.97 (t, *J*=7.5 Hz, 3H, CH_3 EPA), 0.88 (t, *J*=6.8 Hz, 3H, CH_3 SFA) ppm. ^{13}C{H} NMR (101 MHz, $CDCl_3$) δ_C: 174.2 (C=O Nap), 173.3 (C=O SFA), 172.6 (C=O EPA), 1579, 135.4, 133.9, 132.2, 129.4, 129.1, 129.02, 128.97, 128.7, 128.5, 128.4, 128.3, 128.2, 128.0, 127.3, 127.2, 126.3, 126.1, 119.2, 105.7, 69.0, 62.7, 62.1, 55.5, 45.4, 34.1, 33.6, 32.1, 29.8, 29.6, 29.5, 29.4, 29.3, 26.6, 25.79, 25.76 (3), 25.7, 25.0, 24.8, 22.8, 20.7, 18.5, 14.4, 14.3 ppm. HRMS (ESI) *m*/*z*: [M + Na]$^+$ calcd for $C_{49}H_{70}O_7Na$ 793.5014; found, 793.5009.

*2.3. Coupling of DHA: Synthesis of (R,S')-**13b–d**, (S,S')-**13b–d**, (R,S')-**14b–d** and (S,S')-**14b–d***

2.3.1. Synthesis of 2-[4Z,7Z,10Z,13Z,16Z,19Z)-docosa-4,7,10,13,16,19-hexaenoyl]-3-[(*S*)-2-(4-isobutylphenyl)propanoyl]-1-octanoyl-*sn*-glycerol, (*R*,*S*')-**13b**

The same procedure was followed as described for (*R*,*S*')-**13a** using 3-[(*S*)-2-(4-isobutylphenyl)-propanoyl]-1-octanoyl-*sn*-glycerol (*R*,*S*')-**9b** (15 mg, 0.037 mmol), DHA (14 mg, 0.041 mmol), CH_2Cl_2 (2 mL),

DMAP (5 mg, 0.041 mmol) and EDCI (11 mg, 0.054 mmol). Purification on a silica gel chromatography using pet. ether/ethyl acetate (9:1) as eluent afforded the product (*R*,*S*')-**13b** as a yellow oil, in 85% yield (23 mg, 0.032 mmol). $[\alpha]^{20}_{D}$ = +5.77 (c. 1.3, CH_2Cl_2). IR (NaCl, ν_{max} / cm^{-1}): 3013 (vs), 2959 (vs), 2931 (vs), 2872 (vs), 1739 (vs). ^1H NMR (400 MHz, $CDCl_3$) δ_H: 7.18 (d, *J*=7.8 Hz, 2H, Ibu-2,6), 7.08 (d, *J*=7.8 Hz, 2H, Ibu-3,5), 5.50-5.24 (m, 12H, =CH), 5.23-5.17 (m, 1H, CH *sn*-2), 4.29 (dd, *J*=11.9, 4.3 Hz, 1H, CH_2 *sn*-1/3), 4.21 (dd, *J*=11.9, 4.3 Hz, 1H, CH_2 *sn*-1/3), 4.14 (dd, J=11.9, 5.7 Hz, 1H, CH_2 *sn*-1/3), 4.01 (dd, J=11.9, 5.9 Hz, 1H, CH_2 *sn*-1/3), 3.70 (q, *J*=7.2 Hz, 1H, C*H*CH_3), 2.89-2.79 (m, 10H, =CHC*H*$_2$CH=), 2.44 (d, *J*=7.2 Hz, 2H, C*H*$_2$CH(CH_3)$_2$), 2.37-2.19 (m, 6H, CH_2CH_2COO DHA, CH_2COO SFA), 2.08 (quint., *J*=7.6 Hz, 2H, =CHC*H*$_2$$CH_3$), 1.83 (nonet, *J*=6.8 Hz, 1H, C*H*(CH_3)$_2$), 1.62-1.56 (m, 2H, C*H*$_2$$CH_2$COO SFA), 1.49 (d, *J*=7.2 Hz, 3H, CHC*H*$_3$), 1.36-1.13 (m, 8H, CH_2), 0.97 (t, *J*=7.5 Hz, 3H, CH_3 DHA), 0.89 (d, *J*=6.4 Hz, 6H, CH(C*H*$_3$)$_2$), 0.88 (t, *J*=7.0 Hz, 3H, CH_3 SFA) ppm. $^{13}C\{H\}$ NMR (101 MHz, $CDCl_3$) δ_C: 174.3 (C=O Ibu), 173.3 (C=O SFA), 172.9 (C=O DHA), 140.8, 137.4, 132.2, 129.5 (2), 128.7, 128.5, 128.4, 128.3, 128.2, 128.1, 128.0, 127.9, 127.8, 127.6 (2), 127.3, 127.2, 69.0, 62.5, 62.1, 45.2, 45.1, 34.2, 34.1, 31.4, 30.3, 29.6, 29.3, 25.8 (3), 25.7, 25.5, 24.7, 22.7, 22.5 (2), 22.4, 20.7, 18.4, 14.4, 14.0 ppm. HRMS (ESI) *m*/*z*: $[M + Na]^+$ calcd for $C_{46}H_{68}O_6Na$ 739.4908; found, 739.4876.

2.3.2. Synthesis of 1-decanoyl-2-[4*Z*,7*Z*,10*Z*,13*Z*,16*Z*,19*Z*)-docosa-4,7,10,13,16,19-hexaenoyl]-3-[(*S*)-2-(4-iso-butylphenyl)propanoyl]-*sn*-glycerol, (*R*,*S*')-**13c**

The same procedure was followed as described for (*R*,*S*')-**13a** using 1-decanoyl-3-[(*S*)-2-(4-isobutylphenyl)propanoyl]-*sn*-glycerol (*R*,*S*')-**9c** (25 mg, 0.058 mmol), DHA (21 mg, 0.065 mmol), CH_2Cl_2 (2.5 mL), DMAP (8 mg, 0.063 mmol) and EDCI (16 mg, 0.085 mmol). Purification on a silica gel chromatography using pet. ether/ethyl acetate (9:1) as eluent afforded the product (*R*,*S*')-**11c** as a yellow oil, in 72% yield (31 mg, 0.042 mmol). $[\alpha]^{20}_{D}$ = +7.91 (c. 1.1, CH_2Cl_2). IR (NaCl, ν_{max} / cm^{-1}): 3013 (vs), 2958 (vs), 2927 (vs), 2855 (vs), 1744 (vs). ^1H NMR (400 MHz, $CDCl_3$) δ_H: 7.18 (d, *J*=7.8 Hz, 2H, Ibu-2,6), 7.08 (d, *J*=7.8 Hz, 2H, Ibu-3,5), 5.50-5.24 (m, 12H, =CH), 5.23-5.17 (m, 1H, CH *sn*-2), 4.29 (dd, *J*=11.9, 4.3 Hz, 1H, CH_2 *sn*-1/3), 4.21 (dd, *J*=11.9, 4.3 Hz, 1H, CH_2 *sn*-1/3), 4.14 (dd, J=11.9, 5.7 Hz, 1H, CH_2 *sn*-1/3), 4.01 (dd, J=11.9, 5.9 Hz, 1H, CH_2 *sn*-1/3), 3.70 (q, *J*=7.2 Hz, 1H, C*H*CH_3), 2.89-2.79 (m, 10H, =CHC*H*$_2$CH=), 2.44 (d, *J*=7.2 Hz, 2H, C*H*$_2$CH(CH_3)$_2$), 2.37-2.19 (m, 6H, CH_2CH_2COO DHA, CH_2COO SFA), 2.08 (quint., *J*=7.6 Hz, 2H, =CHC*H*$_2$$CH_3$), 1.83 (nonet, *J*=6.8 Hz, 1H, C*H*(CH_3)$_2$), 1.62-1.56 (m, 2H, C*H*$_2$$CH_2$COO SFA), 1.49 (d, *J*=7.2 Hz, 3H, CHC*H*$_3$), 1.36-1.13 (m, 12H, CH_2), 0.97 (t, *J*=7.5 Hz, 3H, CH_3 DHA), 0.89 (d, *J*=6.4 Hz, 6H, CH(C*H*$_3$)$_2$), 0.88 (t, *J*=7.0 Hz, 3H, CH_3 SFA) ppm. $^{13}C\{H\}$ NMR (101 MHz, $CDCl_3$) δ_C: 174.3 (C=O Ibu), 173.3 (C=O SFA), 172.9 (C=O DHA), 140.8, 137.4, 132.2, 129.5 (2), 128.7, 128.5, 128.4, 128.3, 128.2, 128.1, 128.0, 127.9, 127.8, 127.6 (2), 127.3, 127.2, 69.0, 62.5, 62.1, 45.2, 45.1, 34.2, 34.1, 31.4, 30.3, 29.6, 29.44, 29.42, 29.3, 25.8 (3), 25.7, 25.5, 24.7, 22.7, 22.5 (2), 22.4, 20.7, 18.4, 14.4, 14.0 ppm. HRMS (ESI) *m*/*z*: $[M + Na]^+$ calcd for $C_{48}H_{72}O_6Na$ 767.5221; found, 767.5193.

2.3.3. Synthesis of 2-[4*Z*,7*Z*,10*Z*,13*Z*,16*Z*,19*Z*)-docosa-4,7,10,13,16,19-hexaenoyl]-1-dodecanoyl-3-[(*S*)-2-(4-isobutylphenyl)propanoyl]-*sn*-glycerol, (*R*,*S*')-**13d**

The same procedure was followed as described for (*R*,*S*')-**13a** using 1-dodecanoyl-3-[(*S*)-2-(4-isobutylphenyl)propanoyl]-*sn*-glycerol (*R*,*S*')-**9d** (15 mg, 0.032 mmol), DHA (11 mg, 0.033 mmol), CH_2Cl_2 (1.6 mL), DMAP (4 mg, 0.035 mmol) and EDCI (9 mg, 0.047 mmol). Purification on a silica gel chromatography using pet. ether/ethyl acetate (4:1) as eluent afforded the product (*R*,*S*')-**13d** as a yellow oil, in 84% yield (21 mg, 0.027 mmol). $[\alpha]^{20}_{D}$ = +5.42 (c. 1.2, CH_2Cl_2). IR (NaCl, ν_{max} / cm^{-1}): 3013 (vs), 2958 (vs), 2924 (vs), 2854 (vs), 1743 (vs). ^1H NMR (400 MHz, $CDCl_3$) δ_H: 7.18 (d, *J*=7.8 Hz, 2H, Ibu-2,6), 7.08 (d, *J*=7.8 Hz, 2H, Ibu-3,5), 5.50-5.24 (m, 12H, =CH), 5.23-5.17 (m, 1H, CH *sn*-2), 4.29 (dd, *J*=11.9, 4.3 Hz, 1H, CH_2 *sn*-1/3), 4.21 (dd, *J*=11.9, 4.3 Hz, 1H, CH_2 *sn*-1/3), 4.14 (dd, J=11.9, 5.7 Hz, 1H, CH_2 *sn*-1/3), 4.01 (dd, J=11.9, 5.9 Hz, 1H, CH_2 *sn*-1/3), 3.70 (q, *J*=7.2 Hz, 1H, C*H*CH_3), 2.89-2.79 (m, 10H, =CHC*H*$_2$CH=), 2.44 (d, *J*=7.2 Hz, 2H, C*H*$_2$CH(CH_3)$_2$), 2.37-2.19 (m, 6H, CH_2CH_2COO DHA, CH_2COO SFA), 2.08 (quint., *J*=7.6 Hz, 2H, =CHC*H*$_2$$CH_3$), 1.83 (nonet, *J*=6.8 Hz, 1H, C*H*(CH_3)$_2$), 1.62-1.56 (m, 2H, C*H*$_2$$CH_2$COO SFA), 1.49 (d, *J*=7.2 Hz,

3H, $CHCH_3$), 1.36-1.13 (m, 16H, CH_2), 0.97 (t, *J*=7.5 Hz, 3H, CH_3 DHA), 0.89 (d, *J*=6.4 Hz, 6H, $CH(CH_3)_2$), 0.88 (t, *J*=7.0 Hz, 3H, CH_3 SFA) ppm. $^{13}C\{H\}$ NMR (101 MHz, $CDCl_3$) δ_C: 174.3 (C=O Ibu), 173.3 (C=O SFA), 172.9 (C=O DHA), 140.8, 137.4, 132.2, 129.5 (2), 128.7, 128.5, 128.4, 128.3, 128.2, 128.1, 128.0, 127.9, 127.8, 127.6 (2), 127.3, 127.2, 69.0, 62.5, 62.1, 45.2, 45.1, 34.2, 34.1, 31.4, 30.3, 29.8, 29.6, 29.5, 29.4, 29.3, 25.8 (3), 25.7, 25.5, 24.7, 22.7, 22.5 (2), 22.4, 20.72 18.4, 14.4, 14.0 ppm. HRMS (ESI) *m*/*z*: $[M + Na]^+$ calcd for $C_{50}H_{76}O_6Na$ 795.5534; found, 795.5512.

2.3.4. Synthesis of 2-[4*Z*,7*Z*,10*Z*,13*Z*,16*Z*,19*Z*)-docosa-4,7,10,13,16,19-hexaenoyl]-1-[(*S*)-2-(4-isobutylphenyl)propanoyl]-3-octanoyl-*sn*-glycerol, (*S*,*S*')-**13b**

The same procedure was followed as described for (*S*,*S*')-**13a** using 1-[(*S*)-2-(4-isobutylphenyl)propanoyl]-3-octanoyl-*sn*-glycerol (*S*,*S*')-**9b** (12 mg, 0.032 mmol), DHA (11 mg, 0.033 mmol), CH_2Cl_2 (1.6 mL), DMAP (4 mg, 0.032 mmol) and EDCI (9 mg, 0.044 mmol). Purification on a silica gel chromatography using pet. ether/ethyl acetate (4:1) as eluent afforded the product (*S*,*S*')-**13b** as a yellow oil, in 77% yield (17 mg, 0.024 mmol). $[\alpha]^{20}_D$ = +8.57 (c. 0.7, CH_2Cl_2). IR (NaCl, ν_{max} / cm^{-1}): 3013 (vs), 2956 (vs), 2925 (vs), 2855 (vs), 1744 (vs). ^1H NMR (400 MHz, $CDCl_3$) δ_H: 7.18 (d, *J*=7.8 Hz, 2H, Ibu-2,6), 7.08 (d, *J*=7.8 Hz, 2H, Ibu-3,5), 5.46-5.27 (m, 12H, =CH), 5.23 (tt, *J*=5.9, 4.3 Hz, 1H, CH *sn*-2), 4.29 (dd, *J*=11.9, 4.3 Hz, 1H, CH_2 *sn*-1/3), 4.19 (dd, *J*=11.9, 4.3 Hz, 1H, CH_2 *sn*-1/3), 4.12 (dd, J=11.9, 6.1 Hz, 1H, CH_2 *sn*-1/3), 4.05 (dd, J=11.9, 5.9 Hz, 1H, CH_2 *sn*-1/3), 3.69 (q, *J*=7.2 Hz, 1H, $CHCH_3$), 2.90-2.76 (m, 10H, $=CHCH_2CH=$), 2.44 (d, *J*=7.2 Hz, 2H, $CH_2CH(CH_3)_2$), 2.39-2.18 (m, 6H, CH_2CH_2COO DHA, CH_2COO SFA), 2.12-2.03 (m, 2H, $=CHCH_2CH_3$), 1.83 (nonet, *J*=6.8 Hz, 1H, $CH(CH_3)_2$), 1.64-1.56 (m, 2H, CH_2CH_2COO SFA), 1.49 (d, *J*=7.2 Hz, 3H, $CHCH_3$), 1.36-1.20 (m, 8H, CH_2), 0.97 (t, *J*=7.5 Hz, 3H, CH_3 DHA), 0.89 (d, *J*=6.4 Hz, 6H, $CH(CH_3)_2$), 0.88 (t, *J*=7.0 Hz, 3H, CH_3 SFA) ppm. $^{13}C\{H\}$ NMR (101 MHz, $CDCl_3$) δ_C: 174.5 (C=O Ibu), 173.3 (C=O SFA), 172.2 (C=O DHA), 140.9, 137.5, 132.2, 129.5 (2), 128.7, 128.50, 128.45, 128.3, 128.2, 128.1, 128.0, 127.9, 127.8, 127.6 (2), 127.3, 127.2, 69.1, 62.5, 62.1, 45.2, 45.1, 34.2, 34.1, 31.8, 30.3, 29.2, 29.1, 25.8 (3), 25.8, 25.7, 25.0, 22.8, 22.5 (2), 22.4, 20.7, 18.4, 14.4, 14.2 ppm. HRMS (ESI) *m*/*z*: $[M + Na]^+$ calcd for $C_{46}H_{68}O_6Na$ 739.4908; found, 739.4896.

2.3.5. Synthesis of 3-decanoyl-2-[4*Z*,7*Z*,10*Z*,13*Z*,16*Z*,19*Z*)-docosa-4,7,10,13,16,19-hexaenoyl]-1-[(*S*)-2-(4-isobutylphenyl)propanoyl]-*sn*-glycerol, (*S*,*S*')-**13c**

The same procedure was followed as described for (*S*,*S*')-**13a** using 3-decanoyl-1-[(*S*)-2-(4-isobutylphenyl)propanoyl]-*sn*-glycerol (*S*,*S*')-**9c** (15 mg, 0.035 mmol), DHA (21 mg, 0.064 mmol), CH_2Cl_2 (2.5 mL), DMAP (8 mg, 0.063 mmol) and EDCI (16 mg, 0.083 mmol). Purification on a silica gel chromatography using pet. ether/ethyl acetate (9:1) as eluent afforded the product (*S*,*S*')-**13c** as a yellow oil, in 80% yield (21 mg, 0.028 mmol). $[\alpha]^{20}_D$ = +9.60 (c. 1.5, CH_2Cl_2). IR (NaCl, ν_{max} / cm^{-1}): 3013 (vs), 2957 (vs), 2926 (vs), 2855 (vs), 1744 (vs). ^1H NMR (400 MHz, $CDCl_3$) δ_H: 7.20-7.16 (m, 2H, Ibu-2,6), 7.10-7.06 (m, 2H, Ibu-3,5), 5.46-5.25 (m, 12H, =CH), 5.23 (tt, *J*=5.9, 4.3 Hz, 1H, CH *sn*-2), 4.29 (dd, *J*=11.9, 4.3 Hz, 1H, CH_2 *sn*-1/3), 4.19 (dd, *J*=11.9, 4.4 Hz, 1H, CH_2 *sn*-1/3), 4.12 (dd, J=11.9, 6.0 Hz, 1H, CH_2 *sn*-1/3), 4.05 (dd, J=11.9, 5.9 Hz, 1H, CH_2 *sn*-1/3), 3.69 (q, *J*=7.2 Hz, 1H, $CHCH_3$), 2.88-2.79 (m, 10H, $=CHCH_2CH=$), 2.44 (d, *J*=7.2 Hz, 2H, $CH_2CH(CH_3)_2$), 2.36-2.24 (m, 6H, CH_2CH_2COO DHA, CH_2COO SFA), 2.14-2.02 (m, 2H, $=CHCH_2CH_3$), 1.84 (nonet, *J*=6.8 Hz, 1H, $CH(CH_3)_2$), 1.62-1.54 (m, 2H, CH_2CH_2COO SFA), 1.49 (d, *J*=7.2 Hz, 3H, $CHCH_3$), 1.33-1.23 (m, 12H, CH_2), 0.97 (t, *J*=7.5 Hz, 3H, CH_3 DHA), 0.89 (d, *J*=6.8 Hz, 6H, $CH(CH_3)_2$), 0.88 (t, *J*=7.1 Hz, 3H, CH_3 SFA) ppm. $^{13}C\{H\}$ NMR (101 MHz, $CDCl_3$) δ_C: 174.3 (C=O Ibu), 173.3 (C=O SFA), 172.2 (C=O DHA), 140.8, 137.4, 132.2, 129.6, 129.5 (3), 128.7, 128.5, 128.4, 128.2, 128.1, 128.0, 127.8, 127.3 (3), 127.2, 69.1, 62.5, 62.0, 45.2, 45.1, 34.2, 32.0, 30.3, 29.6, 29.4 (3), 29.3, 25.79 (3), 25.76, 25.7, 25.0, 22.8,, 22.7, 22.5 (2), 20.7, 18.4, 14.4, 14.3 ppm. HRMS (ESI) *m*/*z*: $[M + Na]^+$ calcd for $C_{48}H_{72}O_6Na$ 767.5221; found, 767.5217.

2.3.6. Synthesis of 2-[4Z,7Z,10Z,13Z,16Z,19Z)-docosa-4,7,10,13,16,19-hexaenoyl]-3-dodecanoyl-1-[(*S*)-2-(4-isobutylphenyl)propanoyl]-*sn*-glycerol, (*S*,*S*')-**13d**

The same procedure was followed as described for (*S*,*S*')-**13a** using 3-dodecanoyl-1-[(*S*)-2-(4-isobutylphenyl)propanoyl]-*sn*-glycerol (*S*,*S*')-**9d** (11 mg, 0.024 mmol), DHA (9 mg, 0.026 mmol), CH_2Cl_2 (1.3 mL), DMAP (3 mg, 0.026 mmol) and EDCI (7 mg, 0.035 mmol). Purification on a silica gel chromatography using pet. ether/ethyl acetate (4:1) as eluent afforded the product (*S*,*S*')-**13d** as a yellow oil, in 79% yield (15 mg, 0.019 mmol). $[\alpha]^{20}_D$ = +9.67 (c. 0.9, CH_2Cl_2). IR (NaCl, ν_{max} / cm^{-1}): 3013 (vs), 2956 (vs), 2925 (vs), 2854 (vs), 1744 (vs). ^{1}H NMR (400 MHz, $CDCl_3$) δ_H: 7.18 (d, *J*=8.0 Hz, 2H, Ibu-2,6), 7.08 (d, *J*=8.0 Hz, 2H, Ibu-3,5), 5.46-5.26 (m, 12H, =CH), 5.23 (tt, *J*=6.0, 4.3 Hz, 1H, CH *sn*-2), 4.28 (dd, *J*=11.9, 4.3 Hz, 1H, CH_2 *sn*-1/3), 4.19 (dd, *J*=11.9, 4.3 Hz, 1H, CH_2 *sn*-1/3), 4.15 (dd, J=11.9, 6.0 Hz, 1H, CH_2 *sn*-1/3), 4.05 (dd, J=11.9, 5.9 Hz, 1H, CH_2 *sn*-1/3), 3.69 (q, *J*=7.2 Hz, 1H, C*H*CH_3), 2.90-2.76 (m, 10H, =CHC*H*$_2$CH=), 2.44 (d, *J*=7.2 Hz, 2H, C*H*$_2$CH(CH_3)$_2$), 2.38-2.18 (m, 6H, CH_2CH_2COO DHA, CH_2COO SFA), 2.11-2.02 (m, 2H, =CHC*H*$_2$$CH_3$), 1.84 (nonet, *J*=6.9 Hz, 1H, C*H*(CH_3)$_2$), 1.64-1.56 (m, 2H, C*H*$_2$$CH_2$COO SFA), 1.49 (d, *J*=7.2 Hz, 3H, CHC*H*$_3$), 1.36-1.20 (m, 16H, CH_2), 0.97 (t, *J*=7.5 Hz, 3H, CH_3 DHA), 0.89 (d, *J*=6.4 Hz, 6H, CH(C*H*$_3$)$_2$), 0.88 (t, *J*=7.0 Hz, 3H, CH_3 SFA) ppm. ^{13}C{H} NMR (101 MHz, $CDCl_3$) δ_C: 174.3 (C=O Ibu), 173.3 (C=O SFA), 172.9 (C=O DHA), 140.8, 137.5, 132.2, 129.5 (2), 128.7, 128.5, 128.4, 128.23, 128.15, 128.1, 128.0, 127.84, 127.82, 127.29 (3), 127.2, 69.1, 62.6, 62.1, 45.2, 45.1, 34.3, 34.2, 32.1, 30.3, 29.9, 29.80, 29.77, 29.7, 29.4, 29.3, 25.80 (3), 25.77, 25.7, 25.0, 22.8, 22.5 (2), 22.4, 20.7, 18.4, 14.4, 14.3 ppm. HRMS (ESI) *m*/*z*: [M + Na]$^+$ calcd for $C_{50}H_{76}O_6Na$ 795.5534; found, 795.5508.

2.3.7. Synthesis of 2-[4Z,7Z,10Z,13Z,16Z,19Z)-docosa-4,7,10,13,16,19-hexaenoyl]-3-[(*S*)-2-(6-methoxy-naphthalen-2-yl)propanoyl]-1-octanoyl-*sn*-glycerol, (*R*,*S*')-**14b**

The same procedure was followed as described for (*R*,*S*')-**14a** using 3-[(*S*)-2-(6-methoxynaphthalen-2-yl)propanoyl]-1-octanoyl-*sn*-glycerol (*R*,*S*')-**10b** (10 mg, 0.023 mmol), DHA (8 mg, 0.025 mmol), CH_2Cl_2 (1.5 mL), DMAP (3 mg, 0.025 mmol) and EDCI (7 mg, 0.037 mmol). Purification on a silica gel chromatography using pet. ether/ethyl acetate (4:1) as eluent afforded the product (*R*,*S*')-**14b** as a yellow oil, in 83% yield (14 mg, 0.019 mmol). $[\alpha]^{20}_D$ = +8.37 (c. 0.8, CH_2Cl_2). IR (NaCl, ν_{max} / cm^{-1}): 3013 (vs), 2975 (vs), 2925 (vs), 2873 (vs), 1740 (vs), 1635 (s), 1607 (vs). ^{1}H NMR (400 MHz, $CDCl_3$) δ_H: 7.72-7.66 (m, 2H, Nap-4,8), 7.65 (d, *J*=1.9 Hz, 1H, Nap-1), 7.37 (dd, *J*=8.5, 1.9 Hz, 1H, Nap-3), 7.14 (dd, *J*=8.9, 2.5 Hz, 1H, Nap-7), 7.10 (d, *J*=2.5 Hz, 1H, Nap-5), 5.43-5.24 (m, 12H, =CH), 5.21 (tt, *J*=5.9, 4.5 Hz, 1H, CH *sn*-2), 4.30 (dd, *J*=11.9, 4.4 Hz, 1H, CH_2 *sn*-1/3), 4.21 (dd, *J*=11.9, 4.4 Hz, 1H, CH_2 *sn*-1/3), 4.16 (dd, J=11.9, 5.9 Hz, 1H, CH_2 *sn*-1/3), 4.03 (dd, J=11.9, 5.9 Hz, 1H, CH_2 *sn*-1/3), 3.91 (s, 3H, OCH_3), 3.87 (q, *J*=7.2 Hz, 1H, C*H*CH_3), 2.88-2.75 (m, 10H, =CHC*H*$_2$CH=), 2.32-2.17 (m, 6H, CH_2CH_2COO DHA, =CHC*H*$_2$$CH_3$), 2.13-2.03 (m, 2H, CH_2COO SFA), 1.63-1.50 (m, 2H, C*H*$_2$$CH_2$COO SFA), 1.58 (d, *J*=7.1 Hz, 3H, CHC*H*$_3$), 1.35-1.21 (m, 8H, CH_2), 0.97 (t, *J*=7.5 Hz, 3H, CH_3 DHA), 0.88 (t, *J*=7.0 Hz, 3H, CH_3 SFA) ppm. ^{13}C{H} NMR (101 MHz, $CDCl_3$) δ_C: 174.2 (C=O Nap), 173.3 (C=O SFA), 172.1 (C=O DHA), 157.9, 135.3, 133.9, 132.2, 129.5, 129.4, 129.1, 128.7, 128.47 (2), 128.45, 128.4, 128.3, 128.23, 128.16, 128.0, 127.8, 127.3, 127.2, 126.3, 126.1, 119.2, 105.8, 69.2, 62.5, 62.0, 55.5, 45.5, 34.1, 34.0, 31.8, 29.2, 29.0, 25.8 (3), 25.73, 25.70, 25.0, 22.74, 22.65, 20.7, 18.4, 14.4, 14.2 ppm. HRMS (ESI) *m*/*z*: [M + Na]$^+$ calcd for $C_{47}H_{64}O_7Na$ 763.4544; found, 763.4530.

2.3.8. Synthesis of 1-decanoyl-2-[4Z,7Z,10Z,13Z,16Z,19Z)-docosa-4,7,10,13,16,19-hexaenoyl]-3-[(*S*)-2-(6-methoxynaphthalen-2-yl)propanoyl]-*sn*-glycerol, (*R*,*S*')-**14c**

The same procedure was followed as described for (*R*,*S*')-**14a** using 1-decanoyl-3-[(*S*)-2-(6-methoxynaphthalen-2-yl)propanoyl]-*sn*-glycerol (*R*,*S*')-**10c** (10 mg, 0.022 mmol), DHA (8 mg, 0.024 mmol), CH_2Cl_2 (1.5 mL), DMAP (3 mg, 0.024 mmol) and EDCI (6 mg, 0.032 mmol). Purification on a silica gel chromatography using pet. ether/ethyl acetate (4:1) as eluent afforded the product (*R*,*S*')-**14c** as a yellow oil, in 91% yield (15 mg, 0.020 mmol). $[\alpha]^{20}_D$ = +4.20 (c. 0.5, CH_2Cl_2). IR (NaCl, ν_{max} / cm^{-1}): 3013 (vs), 2974

(vs), 2877 (vs), 2435 (vs), 1740 (vs), 1635 (s), 1605 (vs). ^{1}H NMR (400 MHz, $CDCl_3$) δ_H: 7.72-7.66 (m, 2H, Nap-4,8), 7.64 (d, *J*=1.9 Hz, 1H, Nap-1), 7.37 (dd, *J*=8.5, 1.9 Hz, 1H, Nap-3), 7.14 (dd, *J*=8.9, 2.5 Hz, 1H, Nap-7), 7.10 (d, *J*=2.5 Hz, 1H, Nap-5), 5.45-5.25 (m, 12H, =CH), 5.20 (tt, *J*=5.9, 4.4 Hz, 1H, CH *sn*-2), 4.30 (dd, *J*=11.9, 4.4 Hz, 1H, CH_2 *sn*-1/3), 4.22 (dd, *J*=11.9, 4.4 Hz, 1H, CH_2 *sn*-1/3), 4.16 (dd, J=11.9, 5.9 Hz, 1H, CH_2 *sn*-1/3), 4.03 (dd, J=11.9, 5.9 Hz, 1H, CH_2 *sn*-1/3), 3.91 (s, 3H, OCH_3), 3.86 (q, *J*=7.2 Hz, 1H, C*H*CH_3), 2.90-2.74 (m, 10H, =CHC*H*$_2$CH=), 2.29-2.20 (m, 4H, CH_2CH_2COO DHA), 2.19-2.11 (m, 2H, =CHC*H*$_2CH_3$), 2.10-2.05 (m, 2H, CH_2COO SFA), 1.60-1.51 (m, 2H, C*H*$_2CH_2$COO SFA), 1.58 (d, *J*=7.1 Hz, 3H, CHC*H*$_3$), 1.33-1.20 (m, 12H, CH_2), 0.97 (t, *J*=7.5 Hz, 3H, CH_3 DHA), 0.88 (t, *J*=7.0 Hz, 3H, CH_3 SFA) ppm. ^{13}C{H} NMR (101 MHz, $CDCl_3$) δ_C: 174.2 (C=O Nap), 173.3 (C=O SFA), 172.6 (C=O DHA), 157.9, 135.3, 133.9, 132.2, 129.5, 129.4, 129.1, 128.7, 128.6, 128.44, 128.42, 128.36, 128.3, 128.2, 128.0, 127.8, 127.3, 127.2, 126.3, 126.1, 119.2, 105.7, 69.1, 62.5, 62.1, 55.4, 45.5, 34.1, 33.6, 32.0, 29.9, 29.6, 29.41, 29.39, 25.78 (3), 25.75, 25.7, 24.7, 22.7, 22.4, 20.7, 18.4, 14.4, 14.2 ppm. HRMS (ESI) *m/z*: $[M + Na]^+$ calcd for $C_{49}H_{68}O_7Na$ 791.4857; found, 791.4854.

2.3.9. Synthesis of 2-[4Z,7Z,10Z,13Z,16Z,19Z)-docosa-4,7,10,13,16,19-hexaenoyl]-1-dodecanoyl-3-[(*S*)-2-(6-methoxynaphthalen-2-yl)propanoyl]-*sn*-glycerol, (*R,S*')-**14d**

The same procedure was followed as described for (*R,S*')-**14a** using 1-dodecanoyl-3-[(*S*)-2-(6-methoxynaphthalen-2-yl)propanoyl]-*sn*-glycerol (*R,S*')-**10d** (10 mg, 0.021 mmol), DHA (8 mg, 0.023 mmol), CH_2Cl_2 (1.5 mL), DMAP (3 mg, 0.023 mmol) and EDCI (6 mg, 0.031 mmol). Purification on a silica gel chromatography using pet. ether/ethyl acetate (4:1) as eluent afforded the product (*R,S*')-**14d** as a yellow oil, in 86% yield (14 mg, 0.018 mmol). $[\alpha]^{20}_D$ = +6.71 (c. 1.4, CH_2Cl_2). IR (NaCl, ν_{max} / cm^{-1}): 3013 (vs), 2960 (vs), 2926 (vs), 2873 (vs), 1735 (vs), 1606 (vs). ^{1}H NMR (400 MHz, $CDCl_3$) δ_H: 7.72-7.66 (m, 2H, Nap-4,8), 7.65 (d, *J*=1.9 Hz, 1H, Nap-1), 7.37 (dd, *J*=8.5, 1.9 Hz, 1H, Nap-3), 7.14 (dd, *J*=8.9, 2.5 Hz, 1H, Nap-7), 7.10 (d, *J*=2.5 Hz, 1H, Nap-5), 5.44-5.24 (m, 12H, =CH), 5.21 (tt, *J*=5.9, 4.5 Hz, 1H, CH *sn*-2), 4.30 (dd, *J*=11.9, 4.4 Hz, 1H, CH_2 *sn*-1/3), 4.21 (dd, *J*=11.9, 4.4 Hz, 1H, CH_2 *sn*-1/3), 4.16 (dd, J=11.9, 5.9 Hz, 1H, CH_2 *sn*-1/3), 4.03 (dd, J=11.9, 5.9 Hz, 1H, CH_2 *sn*-1/3), 3.91 (s, 3H, OCH_3), 3.87 (q, *J*=7.2 Hz, 1H, C*H*CH_3), 2.89-2.76 (m, 10H, =CHC*H*$_2$CH=), 2.31-2.16 (m, 6H, CH_2CH_2COO DHA, =CHC*H*$_2CH_3$), 2.13-2.03 (m, 2H, CH_2COO SFA), 1.63-1.50 (m, 2H, C*H*$_2CH_2$COO SFA), 1.58 (d, *J*=7.1 Hz, 3H, CHC*H*$_3$), 1.33-1.20 (m, 16H, CH_2), 0.97 (t, *J*=7.5 Hz, 3H, CH_3 DHA), 0.88 (t, *J*=7.0 Hz, 3H, CH_3 SFA) ppm. ^{13}C{H} NMR (101 MHz, $CDCl_3$) δ_C: 174.2 (C=O Nap), 173.3 (C=O SFA), 172.1 (C=O DHA), 157.9, 135.3, 133.9, 132.2, 129.5, 129.4, 129.1, 128.7, 128.47 (2), 128.45, 128.4, 128.3, 128.23, 128.16, 128.0, 127.8, 127.3, 127.2, 126.3, 126.1, 119.2, 105.8, 69.2, 62.5, 62.1, 55.5, 45.5, 34.1, 34.0, 31.4, 29.8, 29.6, 29.5, 29.4, 29.3, 25.8 (3), 25.73, 25.70, 24.6, 22.7, 22.4, 20.7, 18.4, 14.4, 14.0 ppm. HRMS (ESI) *m/z*: $[M + Na]^+$ calcd for $C_{51}H_{72}O_7Na$ 819.5170; found, 819.5165.

2.3.10. Synthesis of 2-[4Z,7Z,10Z,13Z,16Z,19Z)-docosa-4,7,10,13,16,19-hexaenoyl]-1-[(*S*)-2-(6-methoxy-naphthalen-2-yl)propanoyl]-3-octanoyl-*sn*-glycerol, (*S,S*')-**14b**

The same procedure was followed as described for (*S,S*')-**14a** using 1-[(*S*)-2-(6-methoxynaphthalen-2-yl)propanoyl]-3-octanoyl-*sn*-glycerol (*S,S*')-**10b** (11 mg, 0.026 mmol), DHA (10 mg, 0.030 mmol), CH_2Cl_2 (1.5 mL), DMAP (3 mg, 0.025 mmol) and EDCI (7 mg, 0.037 mmol). Purification on a silica gel chromatography using pet. ether/ethyl acetate (4:1) as eluent afforded the product (*S,S*')-**14b** as a yellow oil, in 68% yield (13 mg, 0.018 mmol). $[\alpha]^{20}_D$ = +11.8 (c. 0.4, CH_2Cl_2). IR (NaCl, ν_{max} / cm^{-1}): 3013 (vs), 2980 (vs), 2944 (vs), 2875 (vs), 1742 (vs). ^{1}H NMR (400 MHz, $CDCl_3$) δ_H: 7.72-7.66 (m, 2H, Nap-4,8), 7.64 (d, *J*=1.9 Hz, 1H, Nap-1), 7.37 (dd, *J*=8.5, 1.9 Hz, 1H, Nap-3), 7.14 (dd, *J*=8.9, 2.5 Hz, 1H, Nap-7), 7.10 (d, *J*=2.5 Hz, 1H, Nap-5), 5.44-5.27 (m, 12H, =CH), 5.24 (tt, *J*=6.0, 4.1 Hz, 1H, CH *sn*-2), 4.30 (dd, *J*=11.9, 4.2 Hz, 1H, CH_2 *sn*-1/3), 4.19 (dd, *J*=11.9, 4.5 Hz, 1H, CH_2 *sn*-1/3), 4.16 (dd, J=11.9, 5.9 Hz, 1H, CH_2 *sn*-1/3), 4.03 (dd, J=11.9, 5.9 Hz, 1H, CH_2 *sn*-1/3), 3.91 (s, 3H, OCH_3), 3.85 (q, *J*=7.2 Hz, 1H, C*H*CH_3), 2.87-2.76 (m, 10H, =CHC*H*$_2$CH=), 2.31-2.17 (m, 6H, CH_2CH_2COO DHA, =CHC*H*$_2CH_3$), 2.12-2.03 (m, 2H, CH_2COO SFA), 1.59-1.45 (m, 2H, C*H*$_2CH_2$COO SFA), 1.58 (d, *J*=7.1 Hz, 3H, CHC*H*$_3$), 1.27-1.15 (m, 8H, CH_2), 0.97 (t, *J*=7.5 Hz, 3H, CH_3 DHA), 0.87 (t, *J*=6.9 Hz, 3H, CH_3 SFA) ppm. ^{13}C{H} NMR (101 MHz, $CDCl_3$) δ_C: 174.5 (C=O Nap), 174.3 (C=O SFA),

172.5 (C=O DHA), 138.3, 135.0, 133.9, 132.2, 129.5, 129.4, 129.1, 128.7, 128.48 (2), 128.45, 128.4, 128.3, 128.2, 128.0, 127.8, 127.3, 127.2, 126.3, 126.1, 119.2, 105.8, 69.1, 62.8, 62.1, 55.5, 45.5, 34.1, 34.0, 31.8, 29.2, 29.0, 25.8 (3), 25.74, 25.71, 25.0, 22.8, 22.7, 20.7, 18.5, 14.4, 14.2 ppm. HRMS (ESI) *m*/*z*: $[M + Na]^+$ calcd for $C_{47}H_{64}O_7Na$ 763.4544; found, 763.4519.

2.3.11. Synthesis of 3-decanoyl-2-[4*Z*,7*Z*,10*Z*,13*Z*,16*Z*,19*Z*)-docosa-4,7,10,13,16,19-hexaenoyl]-1-[(*S*)-2-(6-methoxynaphthalen-2-yl)propanoyl]-*sn*-glycerol, (*S*,*S*')-**14c**

The same procedure was followed as described for (*S*,*S*')-**14a** using 3-decanoyl-1-[(*S*)-2-(6-methoxynaphthalen-2-yl)propanoyl]-*sn*-glycerol (*S*,*S*')-**10c** (17 mg, 0.037 mmol), DHA (20 mg, 0.061 mmol), CH_2Cl_2 (2.5 mL), DMAP (7 mg, 0.060 mmol) and EDCI (15 mg, 0.080 mmol). Purification on a silica gel chromatography using pet. ether/ethyl acetate (4:1) as eluent afforded the product (*S*,*S*')-**14c** as a yellow oil, in 79% yield (22 mg, 0.029 mmol). $[\alpha]^{20}_D$ = +10.8 (c. 1.3, CH_2Cl_2). IR (NaCl, ν_{max} / cm^{-1}): 012 (vs), 2984 (vs), 2925 (vs), 2854 (vs), 1743 (vs), 1635 (s), 1606 (vs). ^1H NMR (400 MHz, $CDCl_3$) δ_H: 7.72-7.66 (m, 2H, Nap-4,8), 7.64 (d, *J*=1.9 Hz, 1H, Nap-1), 7.37 (dd, *J*=8.5, 1.9 Hz, 1H, Nap-3), 7.13 (dd, *J*=8.9, 2.5 Hz, 1H, Nap-7), 7.10 (d, *J*=2.5 Hz, 1H, Nap-5), 5.43-5.27 (m, 12H, =CH), 5.23 (tt, *J*=6.0, 4.1 Hz, 1H, CH *sn*-2), 4.30 (dd, *J*=11.9, 4.1 Hz, 1H, CH_2 *sn*-1/3), 4.19 (dd, *J*=11.9, 4.5 Hz, 1H, CH_2 *sn*-1/3), 4.13 (dd, J=11.9, 6.2 Hz, 1H, CH_2 *sn*-1/3), 4.06 (dd, J=11.9, 5.7 Hz, 1H, CH_2 *sn*-1/3), 3.91 (s, 3H, OCH_3), 3.86 (q, *J*=7.1 Hz, 1H, C*H*CH_3), 2.88-2.77 (m, 10H, =CHC*H*$_2$CH=), 2.32-2.16 (m, 6H, CH_2CH_2COO DHA, =CHC*H*$_2CH_3$), 2.12-2.03 (m, 2H, CH_2COO SFA), 1.59-1.45 (m, 2H, C*H*$_2CH_2COO$ SFA), 1.58 (d, *J*=7.1 Hz, 3H, CHC*H*$_3$), 1.32-1.23 (m, 12H, CH_2), 0.97 (t, *J*=7.5 Hz, 3H, CH_3 DHA), 0.88 (t, *J*=6.8 Hz, 3H, CH_3 SFA) ppm. $^{13}C\{H\}$ NMR (101 MHz, $CDCl_3$) δ_C: 174.5 (C=O Nap), 174.3 (C=O SFA), 172.5 (C=O DHA), 138.3, 135.0, 133.9, 132.2, 129.5, 129.4, 129.1, 128.7, 128.48 (2), 128.45, 128.4, 128.3, 128.2, 128.0, 127.8, 127.3, 127.2, 126.3, 126.1, 119.2, 105.8, 69.1, 62.8, 62.1, 55.5, 45.5, 34.1, 34.0, 31.8, 29.8, 29.6, 29.3, 29.2, 25.8 (3), 25.74, 25.71, 25.0, 22.8, 22.7, 20.7, 18.5, 14.4, 14.2 ppm. HRMS (ESI) *m*/*z*: $[M + Na]^+$ calcd for $C_{49}H_{68}O_7Na$ 791.4857; found, 791.4852.

2.3.12. Synthesis of 2-[4*Z*,7*Z*,10*Z*,13*Z*,16*Z*,19*Z*)-docosa-4,7,10,13,16,19-hexaenoyl]-3-dodecanoyl-1-[(*S*)-2-(6-methoxynaphthalen-2-yl)propanoyl]-*sn*-glycerol, (*S*,*S*')-**14d**

The same procedure was followed as described for (*S*,*S*')-**14a** using 3-dodecanoyl-1-[(*S*)-2-(6-methoxynaphthalen-2-yl)propanoyl]-*sn*-glycerol (*S*,*S*')-**10d** (15 mg, 0.031 mmol), DHA (11 mg, 0.034 mmol), CH_2Cl_2 (1.5 mL), DMAP (4 mg, 0.033 mmol) and EDCI (9 mg, 0.045 mmol). Purification on a silica gel chromatography using pet. ether/ethyl acetate (4:1) as eluent afforded the product (*S*,*S*')-**14d** as a yellow oil, in 83% yield (20 mg, 0.025 mmol). $[\alpha]^{20}_D$ = +10.0 (c. 0.2, CH_2Cl_2). IR (NaCl, ν_{max} / cm^{-1}): 3012 (vs), 2977 (vs), 2941 (vs), 2878 (vs), 2834 (vs), 1742 (vs), 1635 (s), 1607 (vs). ^1H NMR (400 MHz, $CDCl_3$) δ_H: 7.72-7.66 (m, 2H, Nap-4,8), 7.64 (d, *J*=1.9 Hz, 1H, Nap-1), 7.37 (dd, *J*=8.5, 1.9 Hz, 1H, Nap-3), 7.14 (dd, *J*=8.9, 2.5 Hz, 1H, Nap-7), 7.10 (d, *J*=2.5 Hz, 1H, Nap-5), 5.44-5.28 (m, 12H, =CH), 5.27-5.20 (m, 1H, CH *sn*-2), 4.30 (dd, *J*=11.9, 4.2 Hz, 1H, CH_2 *sn*-1/3), 4.19 (dd, *J*=11.9, 4.5 Hz, 1H, CH_2 *sn*-1/3), 4.13 (dd, J=11.9, 6.3 Hz, 1H, CH_2 *sn*-1/3), 4.06 (dd, J=11.9, 5.8 Hz, 1H, CH_2 *sn*-1/3), 3.91 (s, 3H, OCH_3), 3.85 (q, *J*=7.2 Hz, 1H, C*H*CH_3), 2.88-2.77 (m, 10H, =CHC*H*$_2$CH=), 2.30-2.16 (m, 6H, CH_2CH_2COO DHA, =CHC*H*$_2CH_3$), 2.11-2.03 (m, 2H, CH_2COO SFA), 1.59-1.45 (m, 2H, C*H*$_2CH_2COO$ SFA), 1.58 (d, *J*=7.1 Hz, 3H, CHC*H*$_3$), 1.31-1.18 (m, 16H, CH_2), 0.97 (t, *J*=7.5 Hz, 3H, CH_3 DHA), 0.88 (t, *J*=6.8 Hz, 3H, CH_3 SFA) ppm. $^{13}C\{H\}$ NMR (101 MHz, $CDCl_3$) δ_C: 174.2 (C=O Nap), 173.3 (C=O SFA), 172.3 (C=O DHA), 138.3, 135.0, 133.9, 132.2, 129.5, 129.4, 129.1, 128.7, 128.48 (2), 128.45, 128.4, 128.3, 128.2, 128.0, 127.8, 127.4, 127.2, 126.3, 126.1, 119.2, 105.8, 69.1, 62.3, 62.1, 55.5, 45.5, 34.1, 34.0, 32.1, 29.8, 29.2, 29.5, 29.4, 29.3, 25.8 (3), 25.74, 25.71, 25.0, 22.8, 22.7, 20.7, 18.5, 14.4, 14.3 ppm. HRMS (ESI) *m*/*z*: $[M + Na]^+$ calcd for $C_{51}H_{72}O_7Na$ 819.5170; found, 819.5161.

3. NMR Spectra

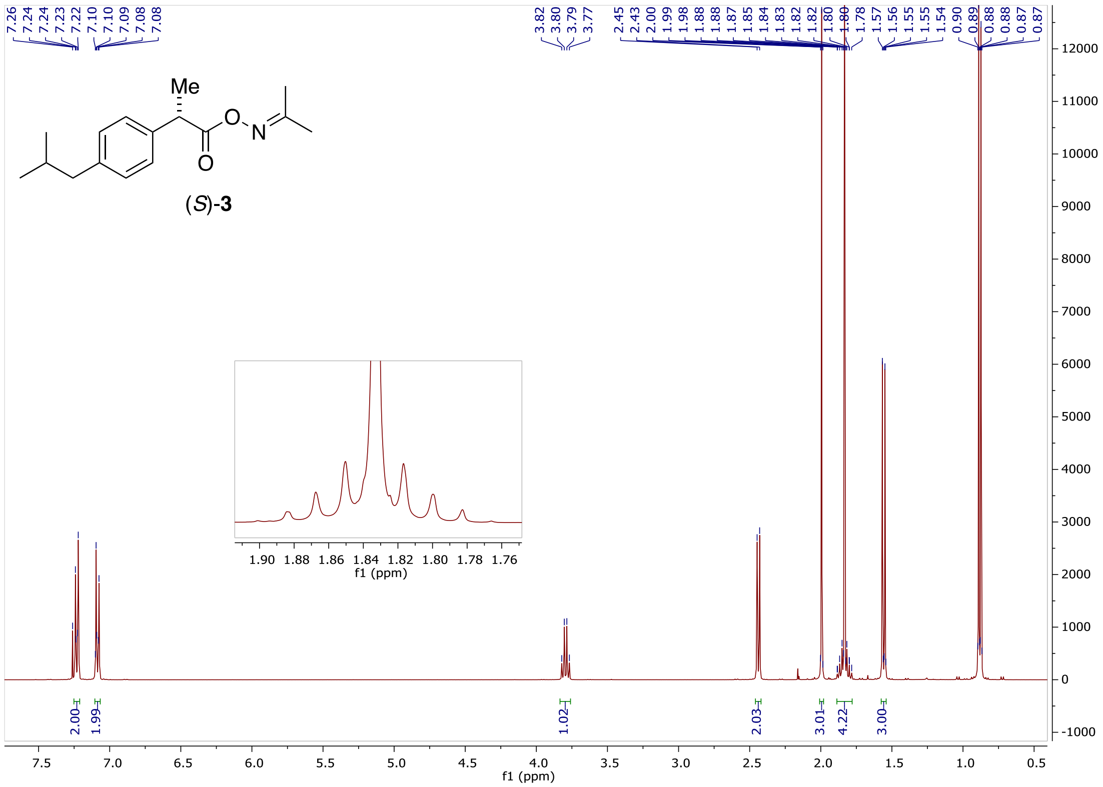

^{1}H NMR (400 MHz, $CDCl_3$) of compound (*S*)-**3**

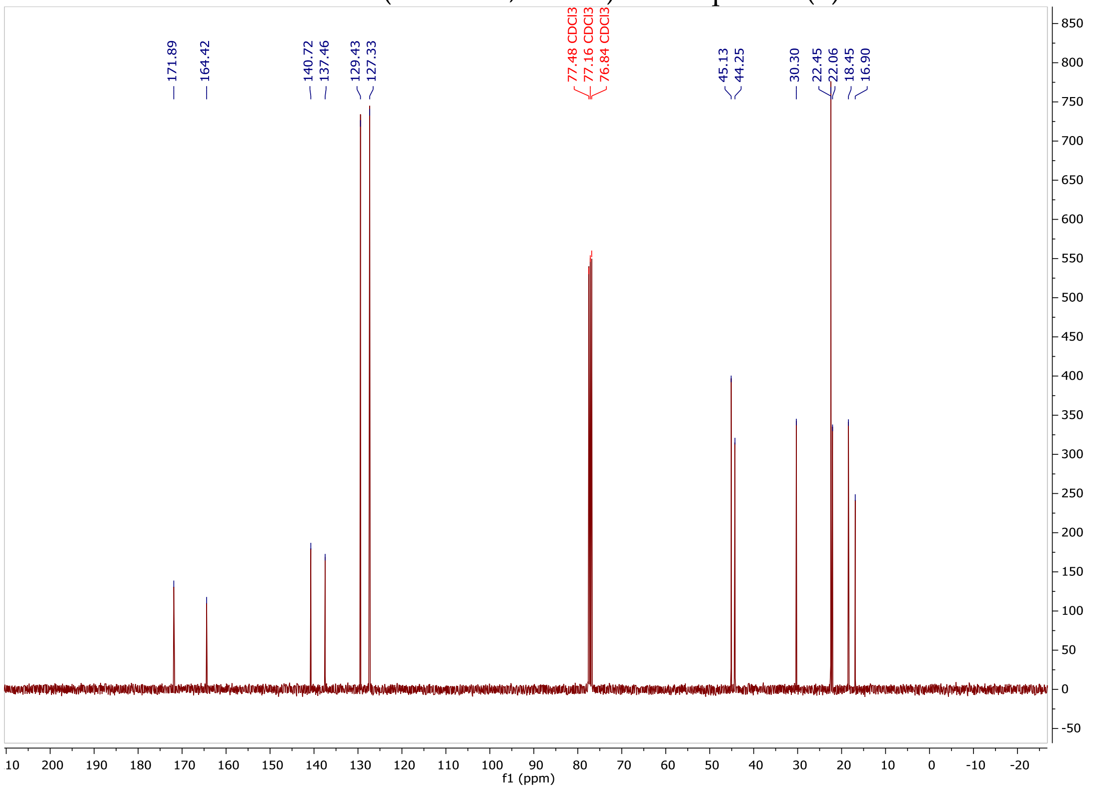

^{13}C{H} NMR (101 MHz, $CDCl_3$) of compound (*S*)-**3**

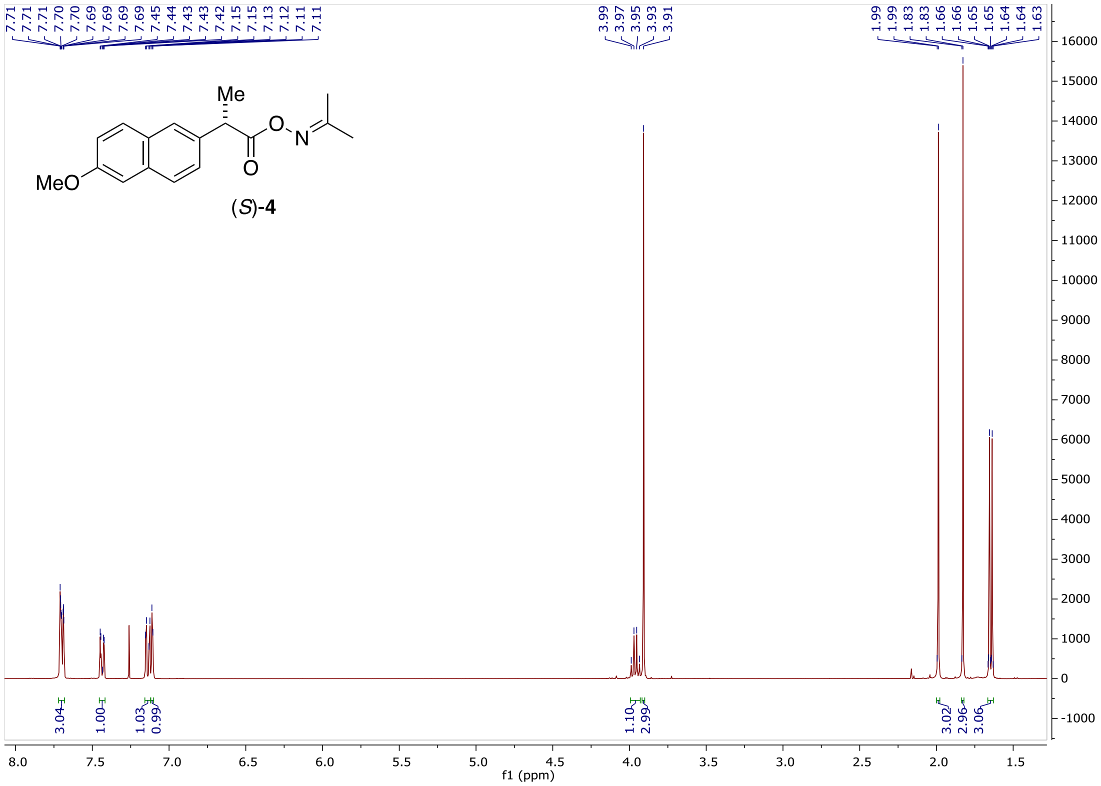

^{1}H NMR (400 MHz, $CDCl_3$) of compound (*S*)-**4**

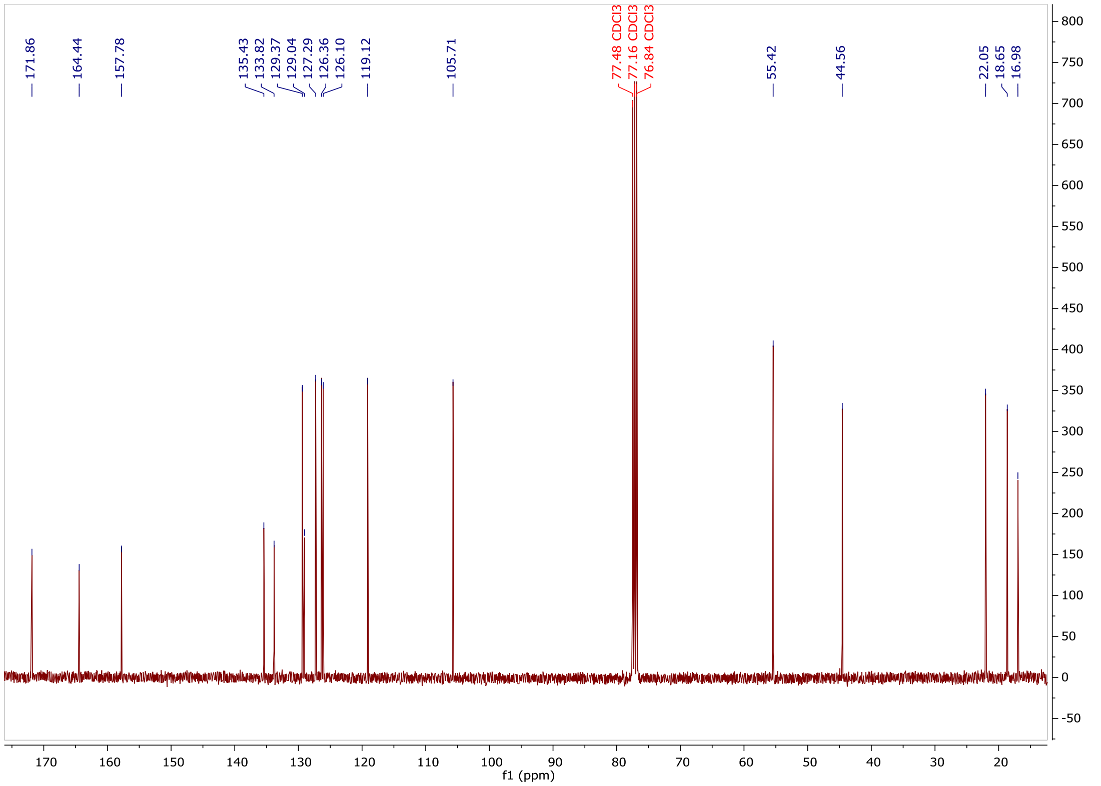

$^{13}C\{H\}$ NMR (101 MHz, $CDCl_3$) of compound (*S*)-**4**

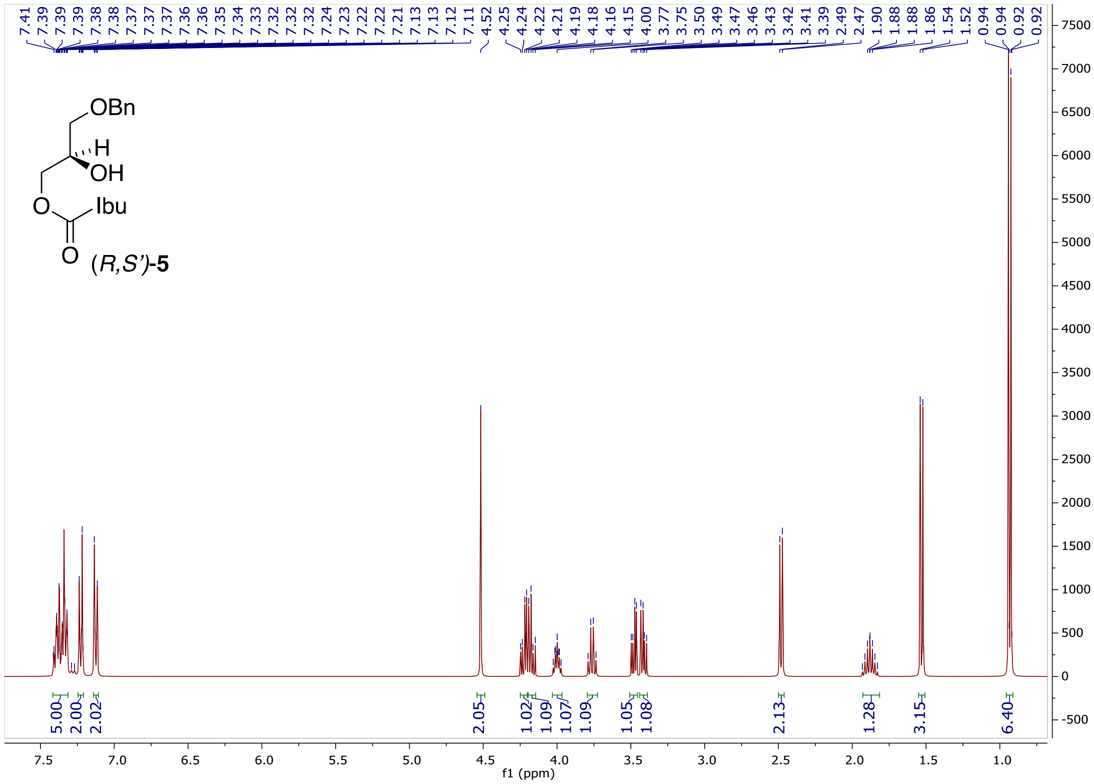

^{1}H NMR (400 MHz, $CDCl_3$) of compound **(*R*,*S'*)-5**

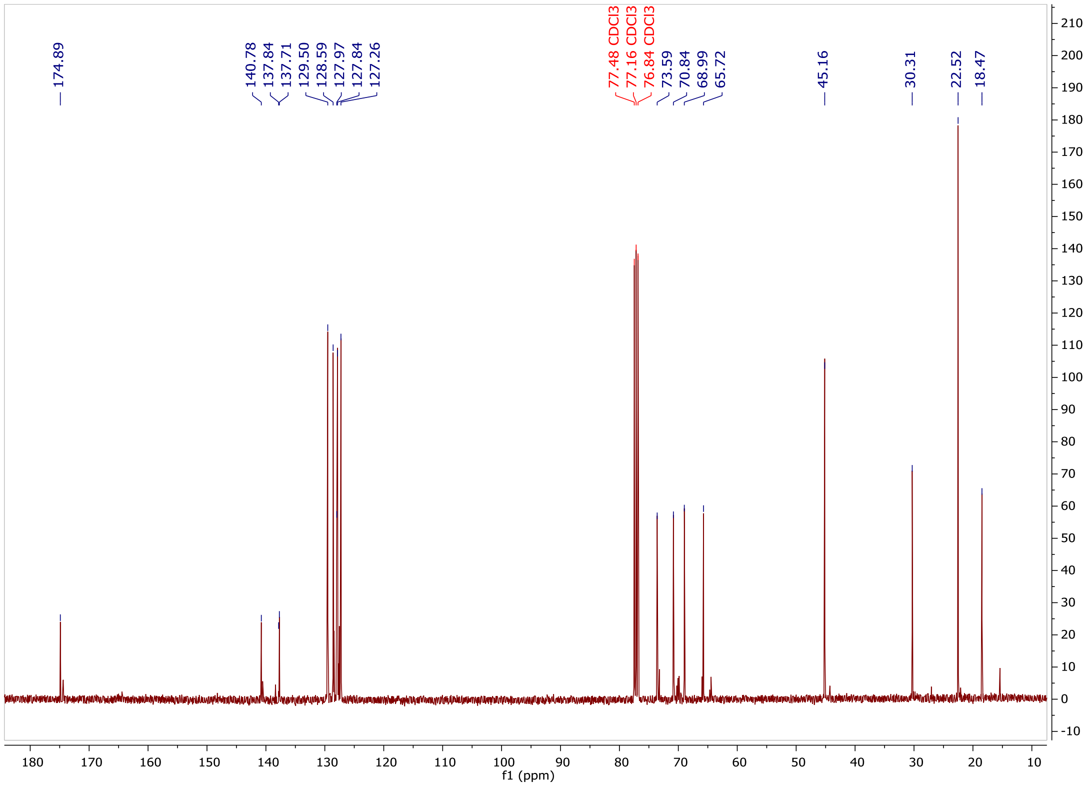

^{13}C{H} NMR (101 MHz, $CDCl_3$) of compound **(*R*,*S'*)-5**

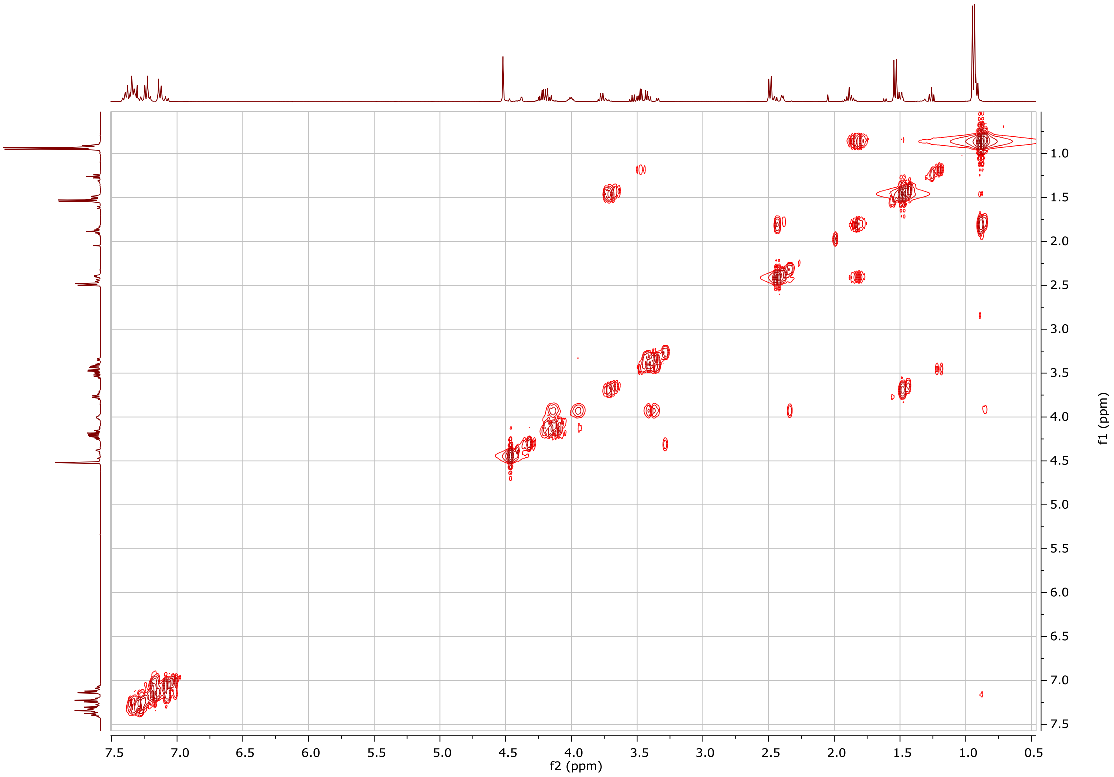

^1H-^1H COSY of compound **(*R*,*S′*)-5**

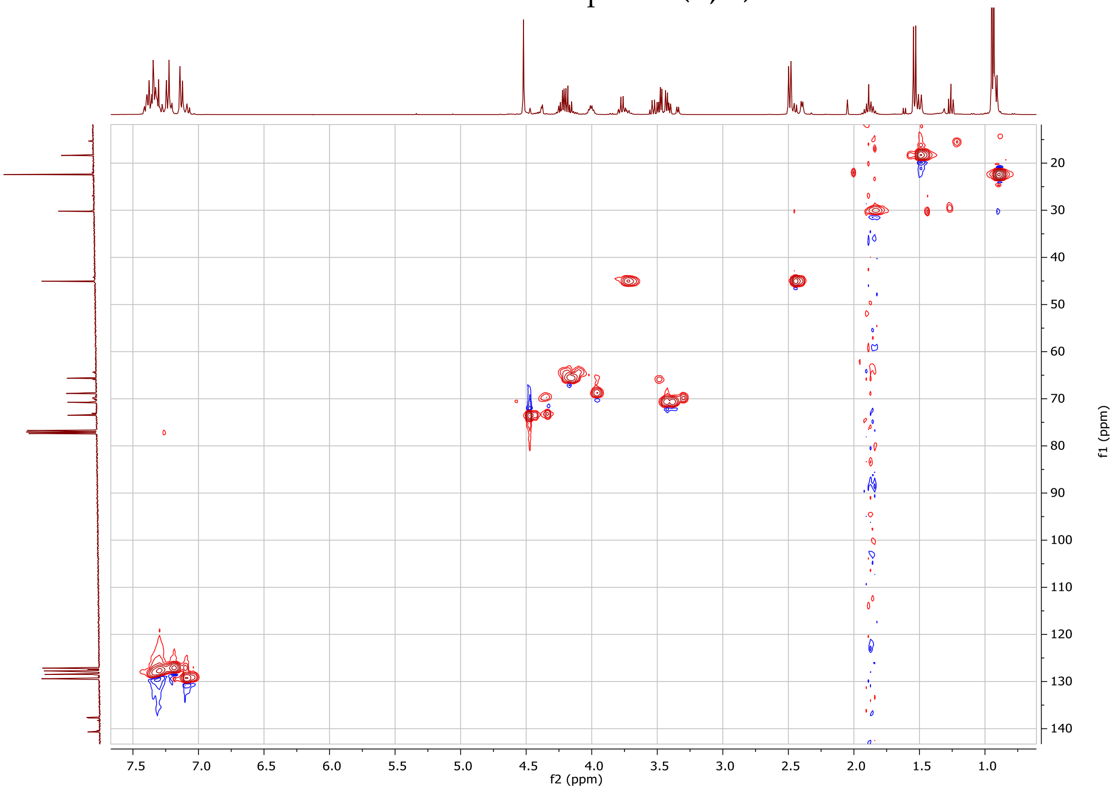

^{13}C-^1H HSQC of compound **(*R*,*S′*)-5**

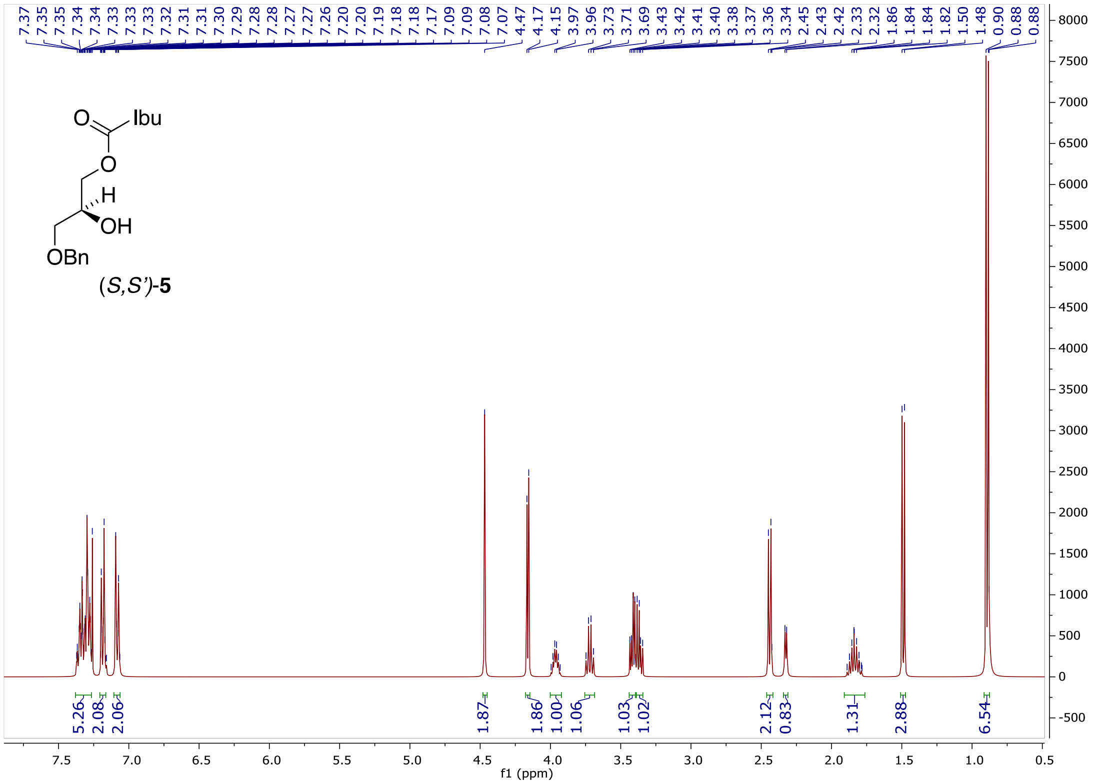

^{1}H NMR (400 MHz, $CDCl_3$) of compound **(*S,S'*)-5**

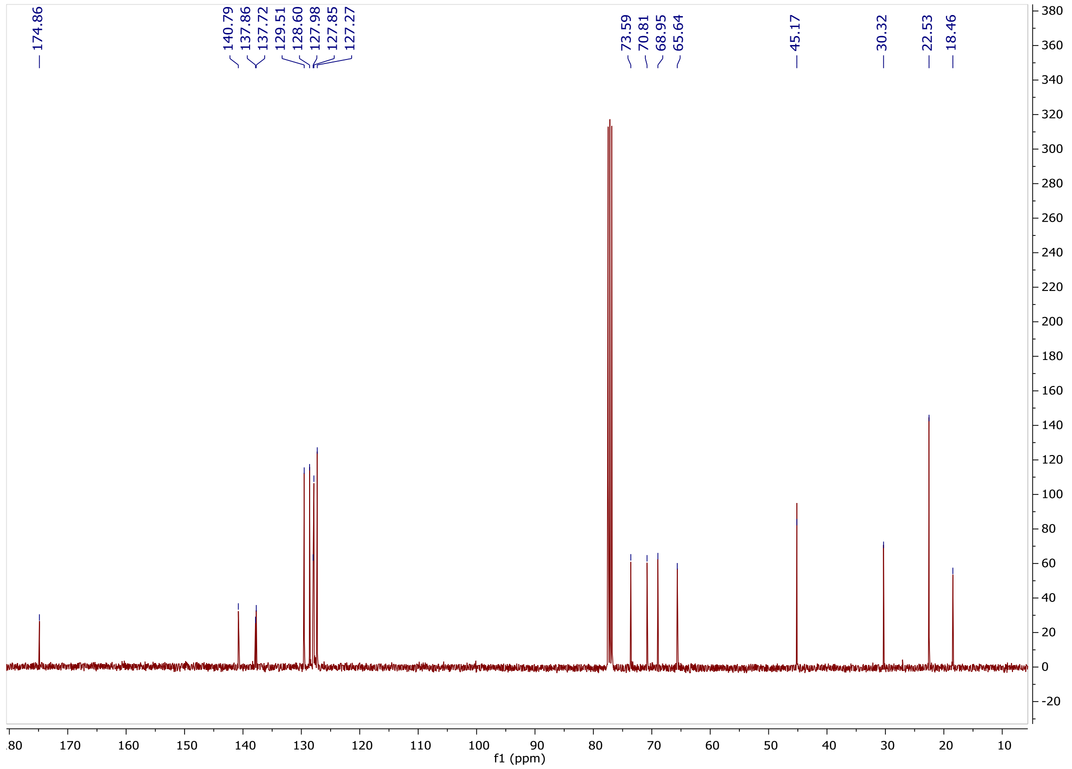

^{13}C{H} NMR (101 MHz, $CDCl_3$) of compound **(*S,S'*)-5**

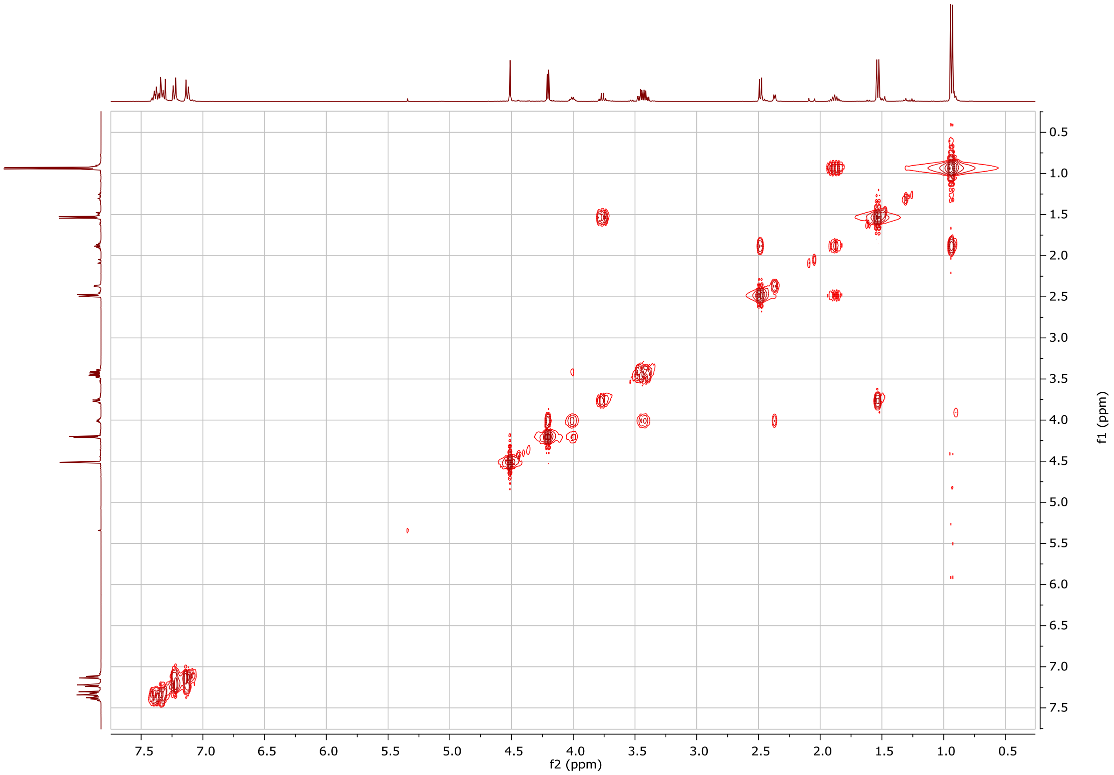

^{1}H-^{1}H COSY of compound **(*S,S′*)-5**

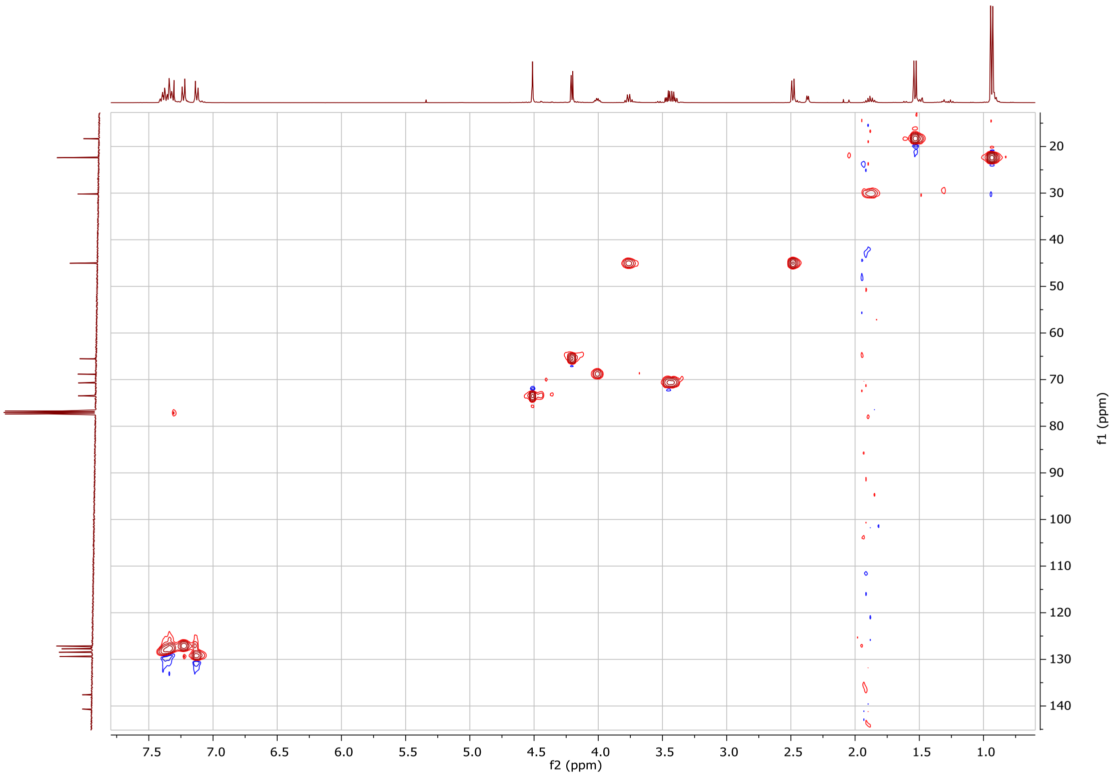

^{13}C-^{1}H HSQC of compound **(*S,S′*)-5**

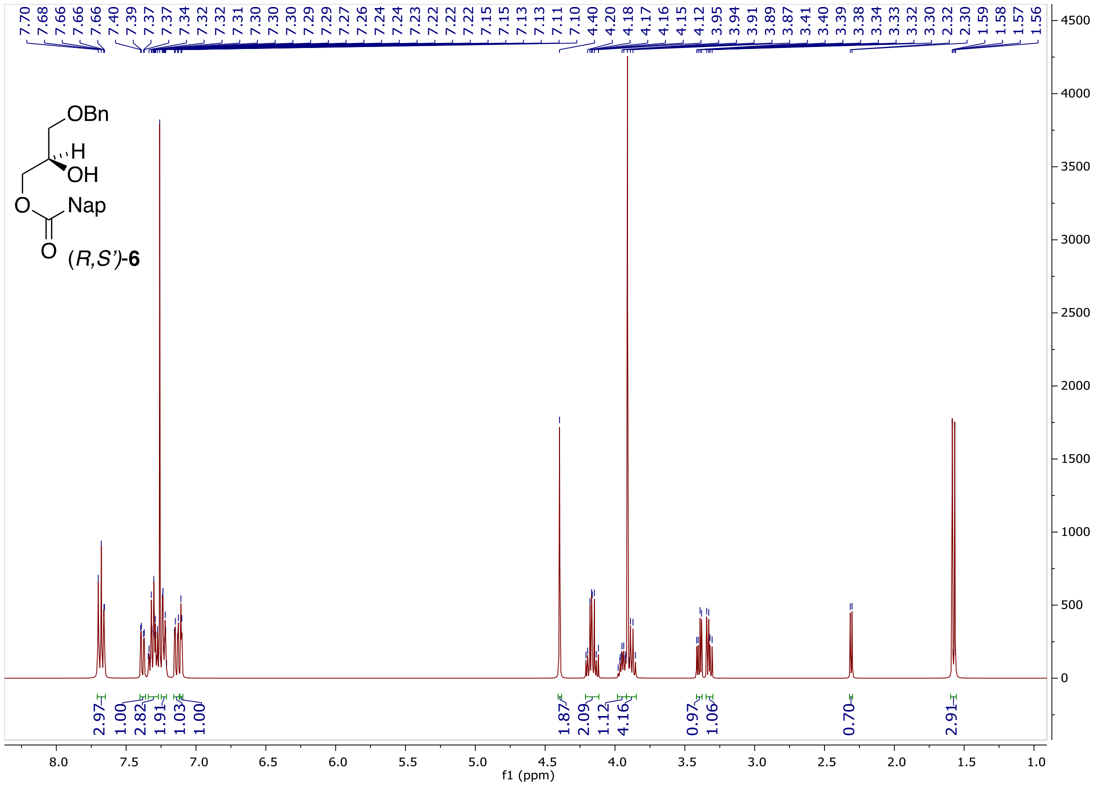

¹H NMR (400 MHz, $CDCl_3$) of compound **(*R,S′*)-6**

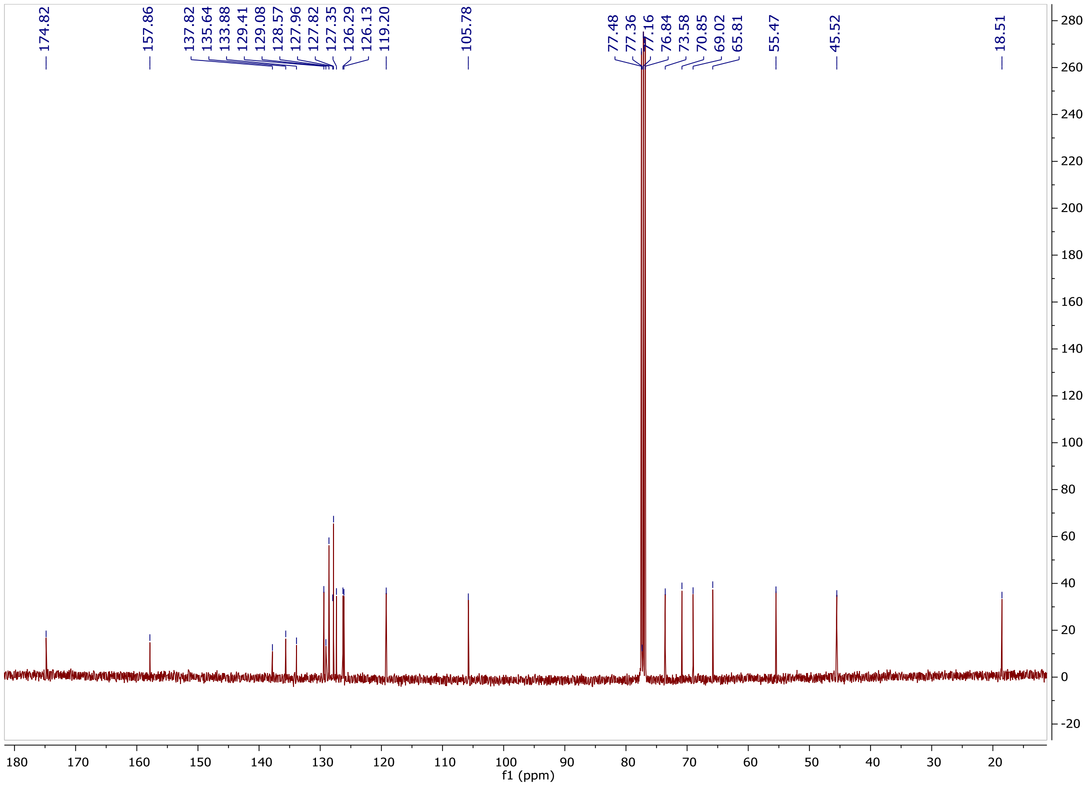

$^{13}C\{H\}$ NMR (101 MHz, $CDCl_3$) of compound **(*R,S′*)-6**

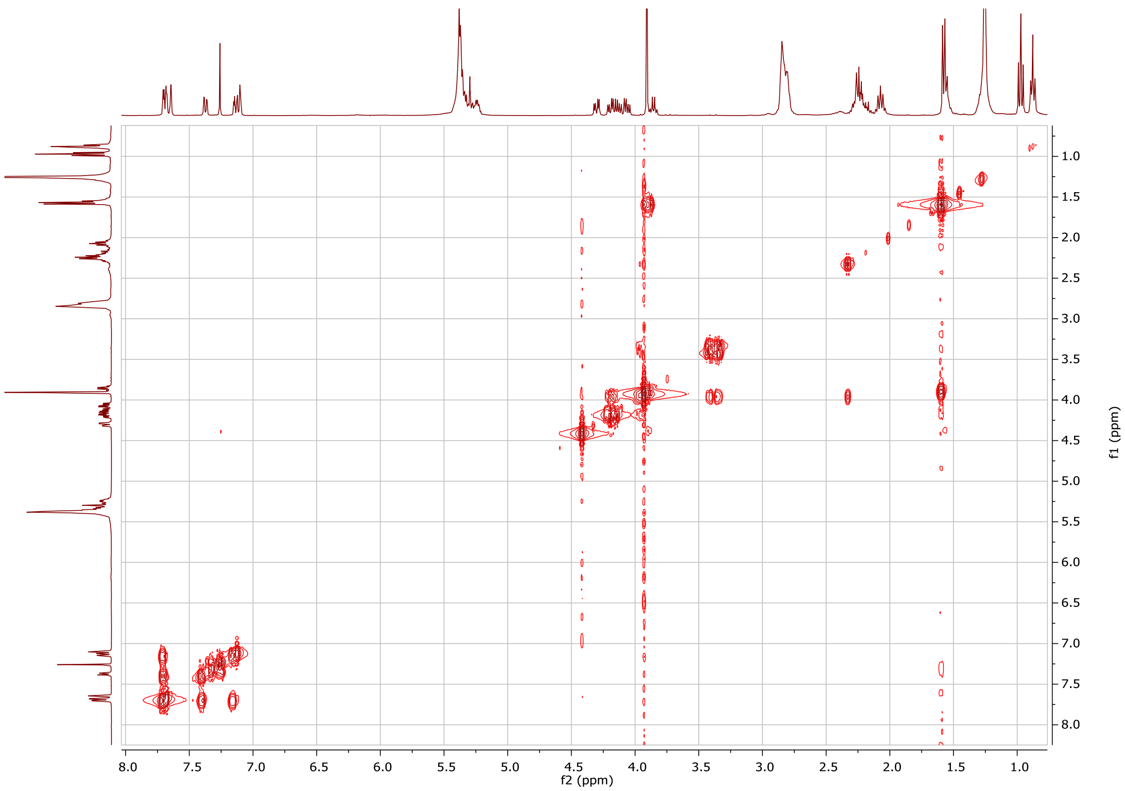

^{1}H-^{1}H COSY of compound **(*R*,*S'*)-6**

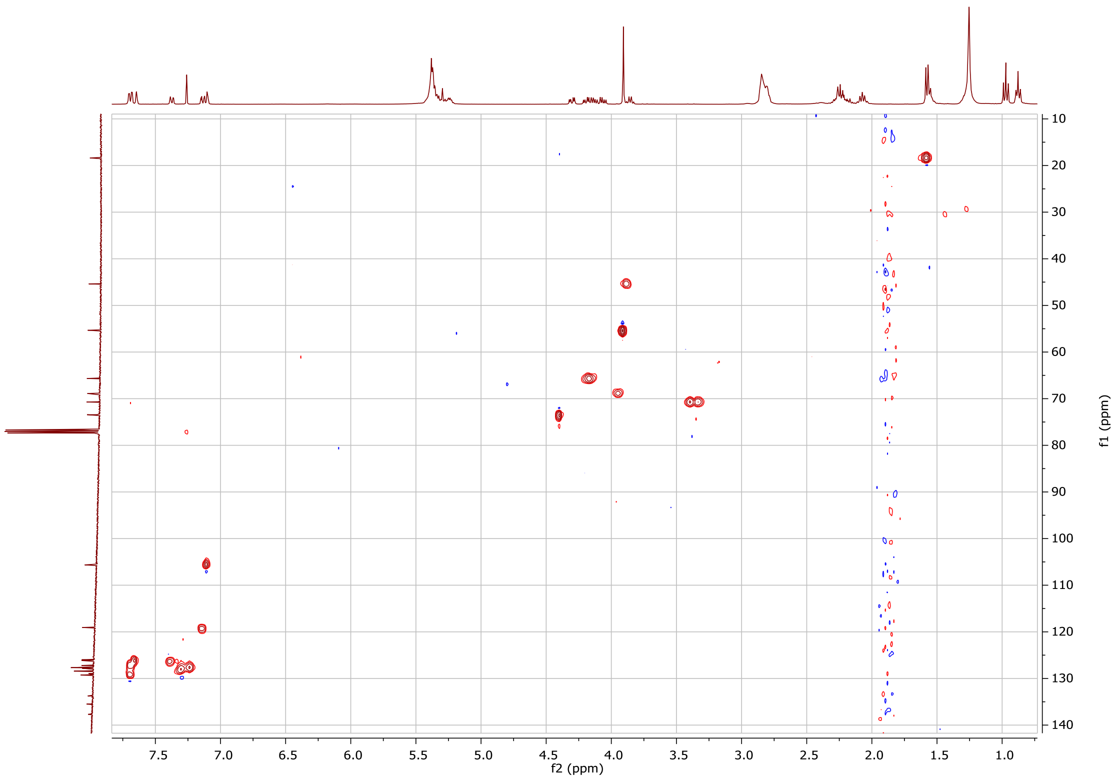

^{13}C-^{1}H HSQC of compound **(*R*,*S'*)-6**

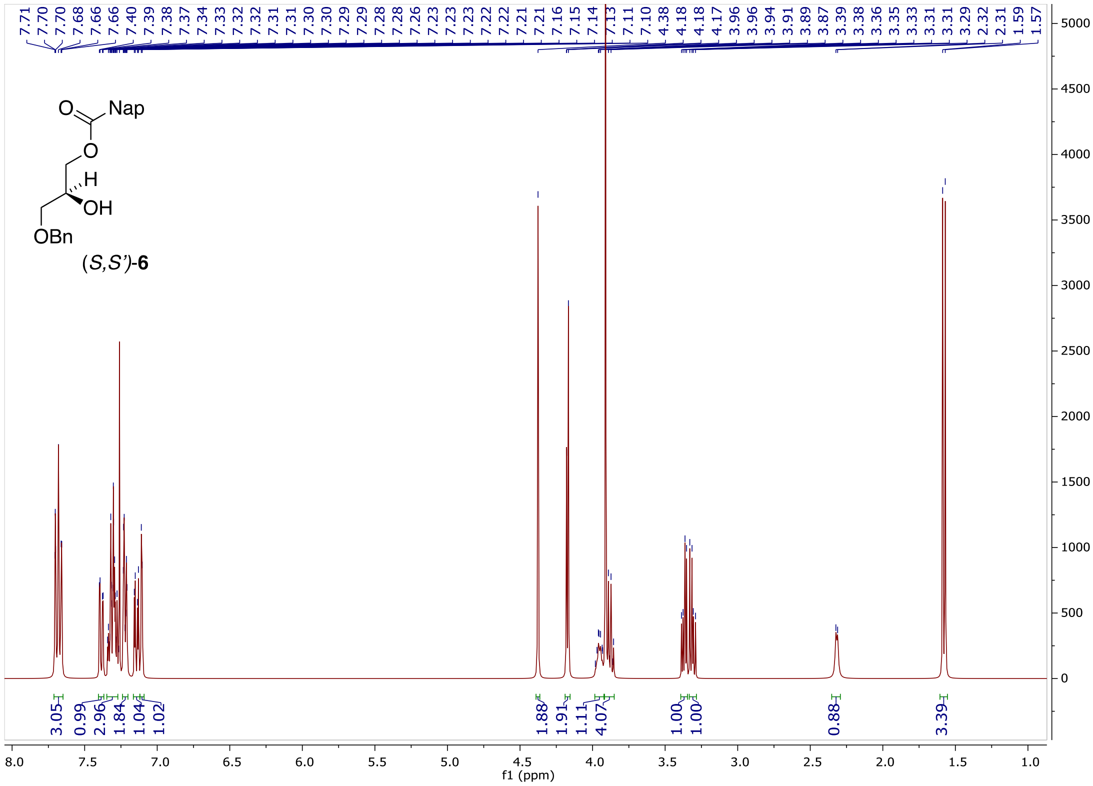

^{1}H NMR (400 MHz, $CDCl_3$) of compound **(*S,S′*)-6**

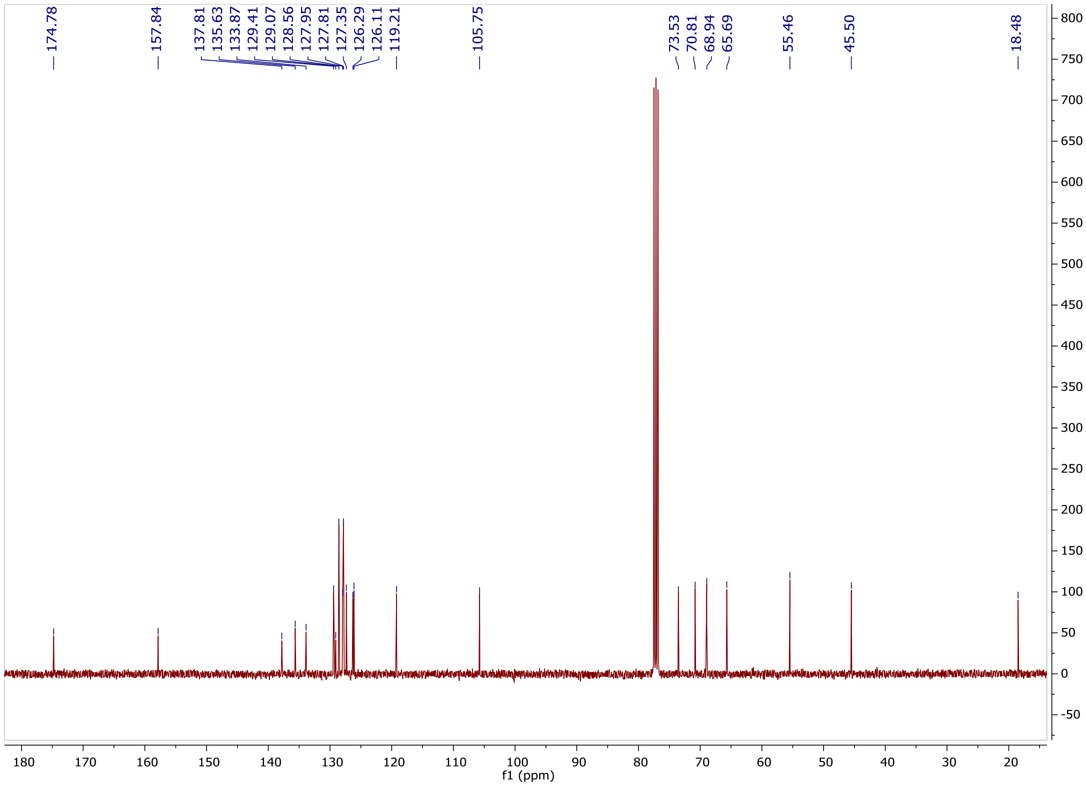

$^{13}C\{H\}$ NMR (101 MHz, $CDCl_3$) of compound **(*S,S′*)-6**

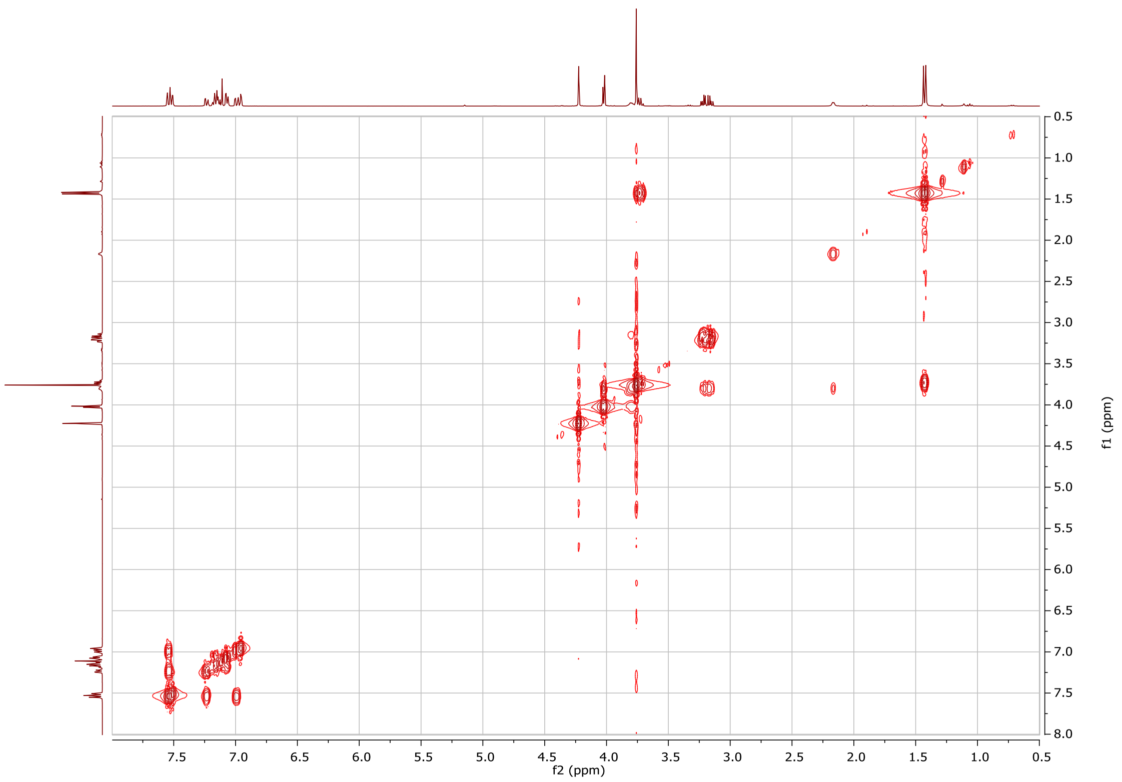

¹H-¹H COSY of compound **(*S*,*S'*)-6**

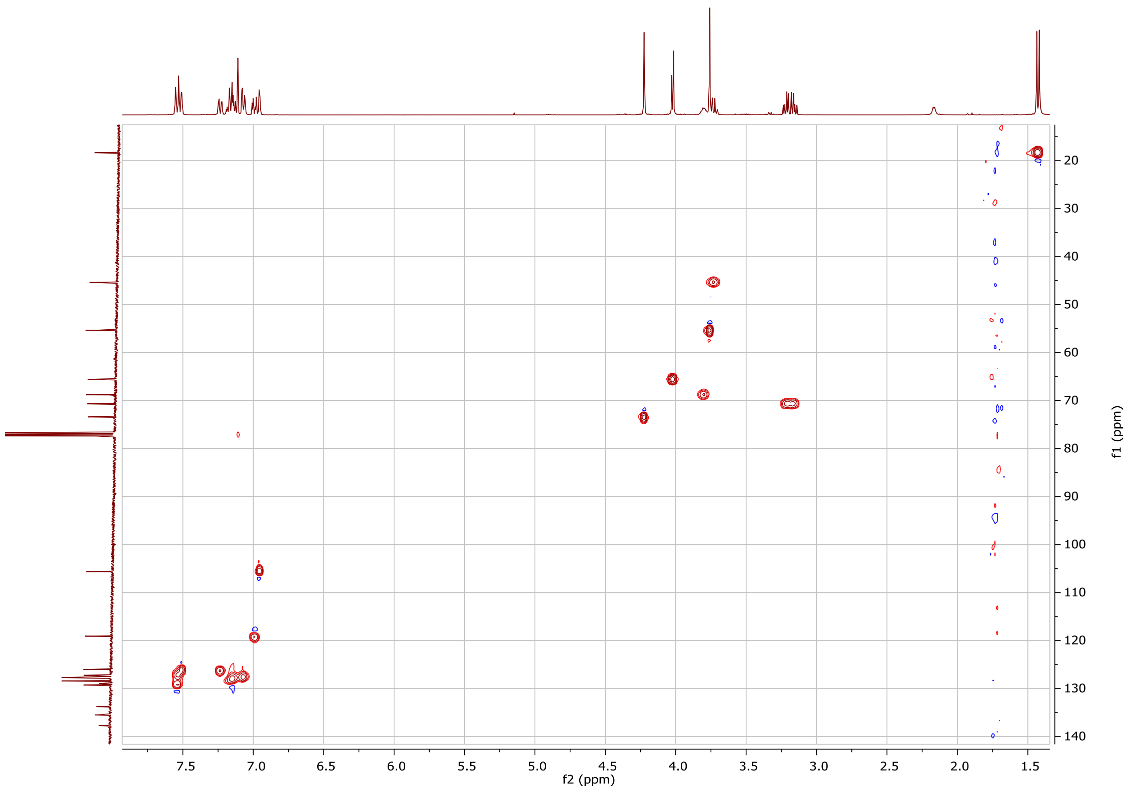

¹³C-¹H HSQC of compound **(*S*,*S'*)-6**

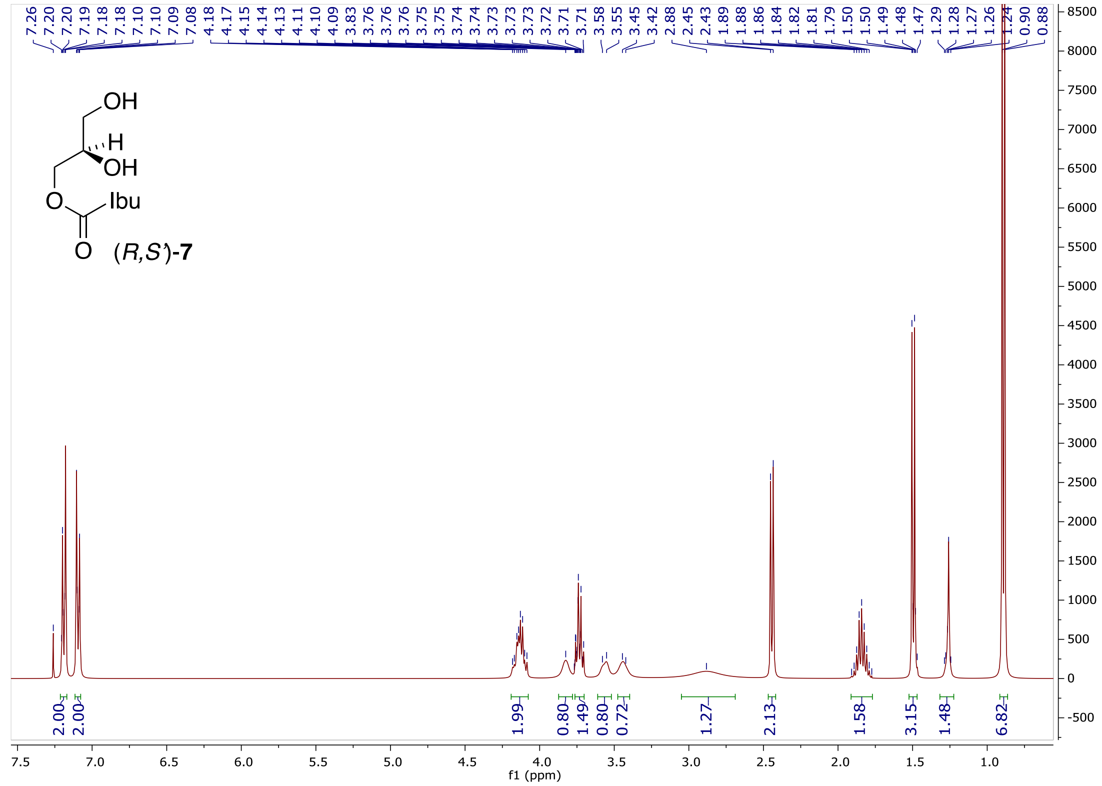

^{1}H NMR (400 MHz, $CDCl_3$) of compound **(*R*,*S′*)-7**

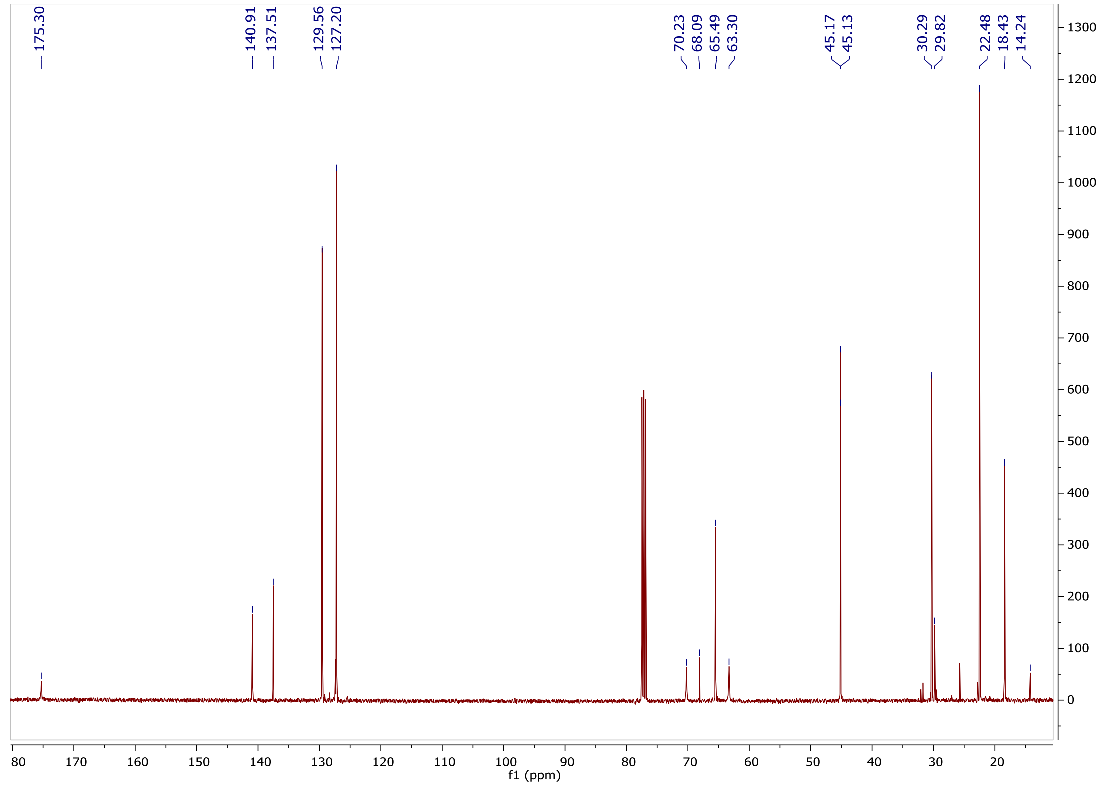

^{13}C{H} NMR (101 MHz, $CDCl_3$) of compound **(*R*,*S′*)-7**

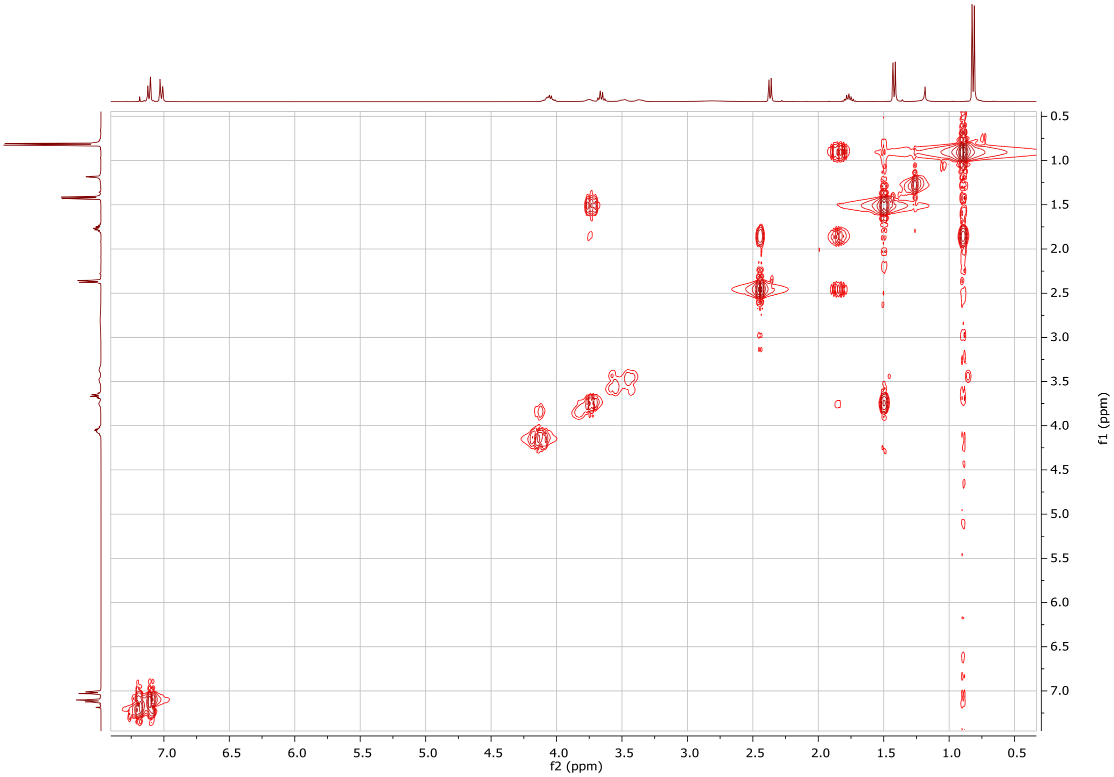

^{1}H-^{1}H COSY of compound **(*R*,*S′*)-7**

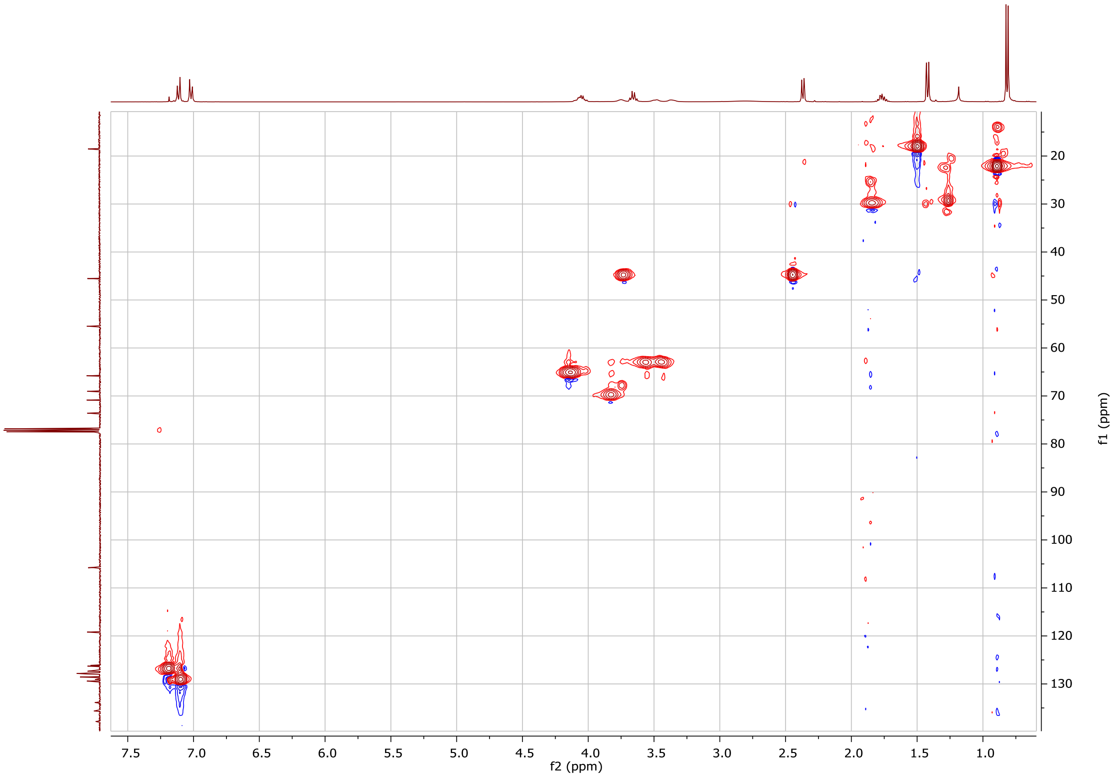

^{13}C-^{1}H HSQC of compound **(*R*,*S′*)-7**

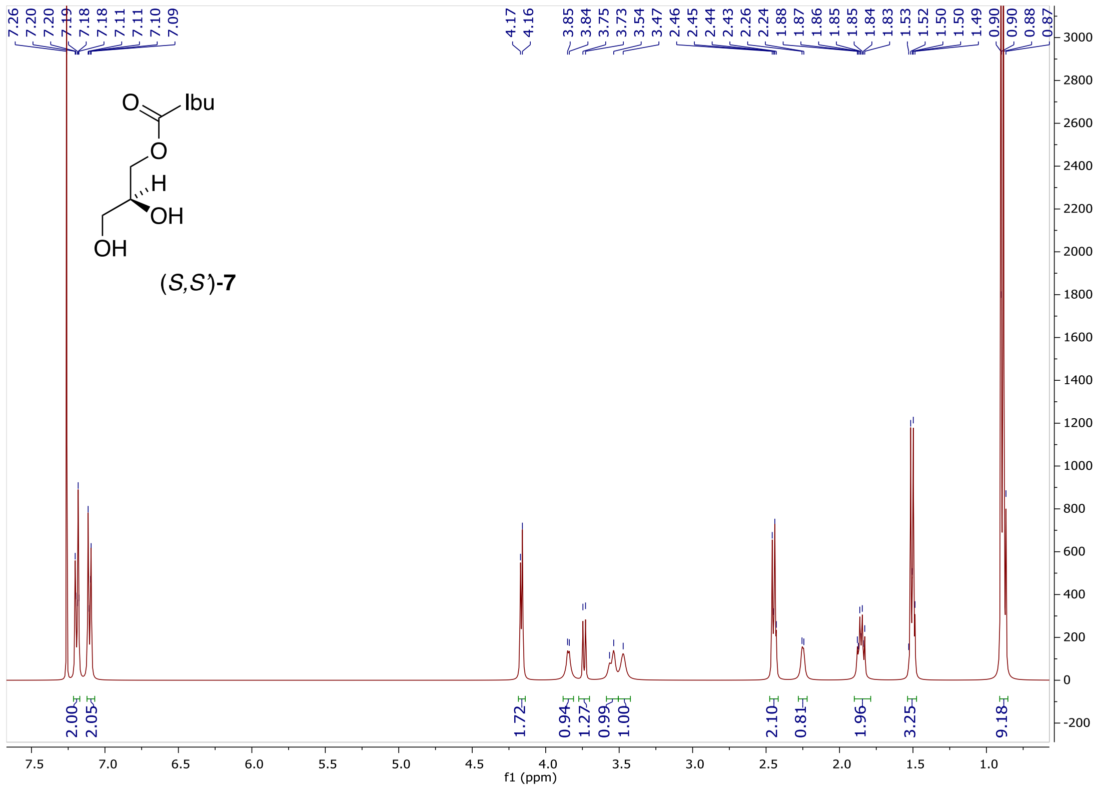

^{1}H NMR (400 MHz, CDCl$_3$) of compound **(*S*,*S'*)-7**

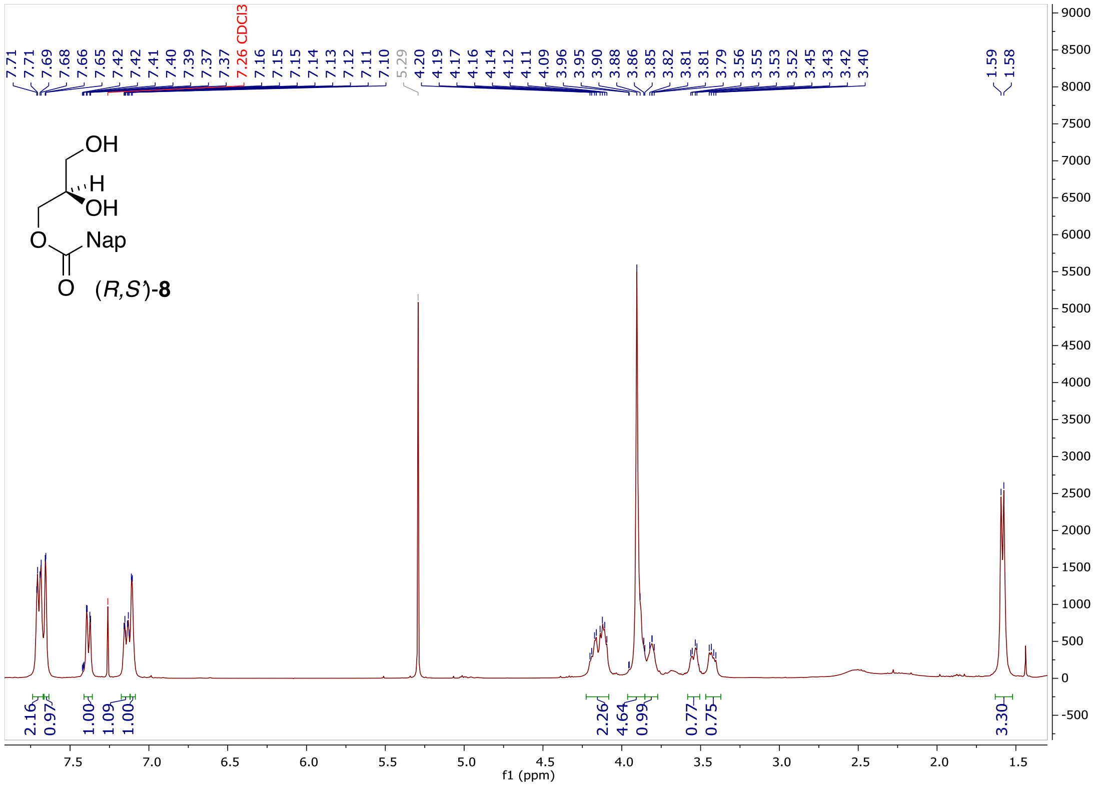

^{1}H NMR (400 MHz, $CDCl_3$) of compound ***(R,S′)*-8**

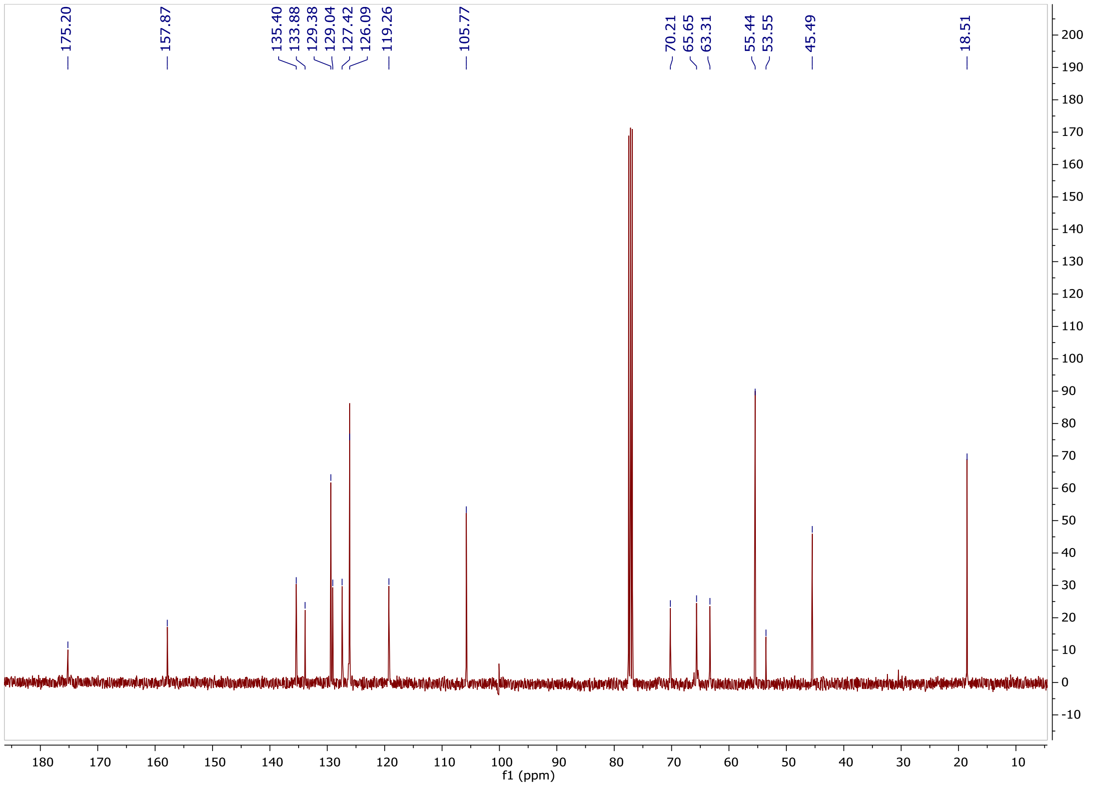

$^{13}C\{H\}$ NMR (101 MHz, $CDCl_3$) of compound ***(R,S′)*-8**

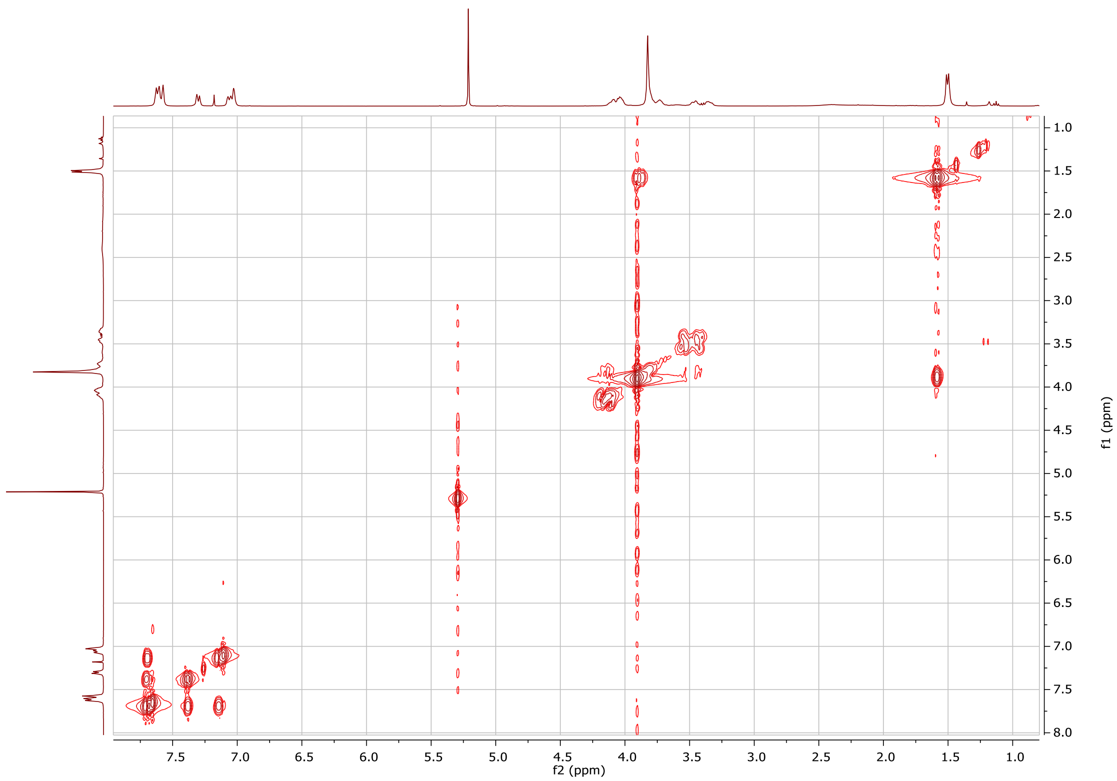

^{1}H-^{1}H COSY of compound **(*R*,*S′*)-8**

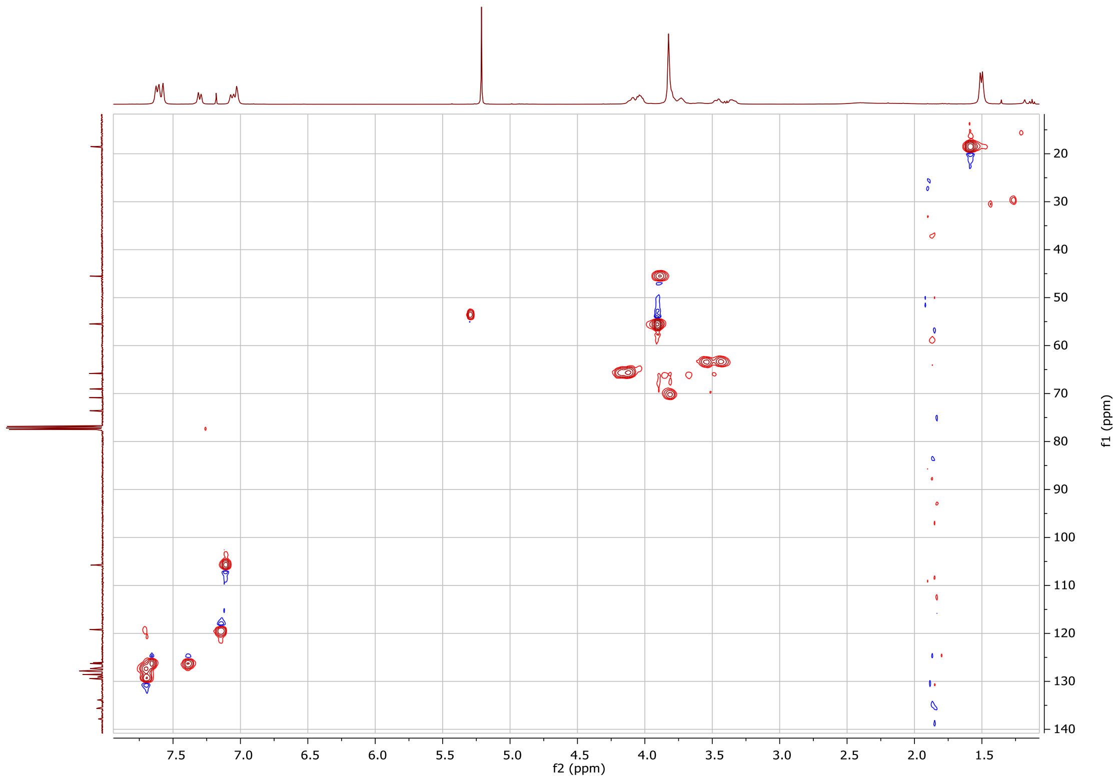

^{13}C-^{1}H HSQC of compound **(*R*,*S′*)-8**

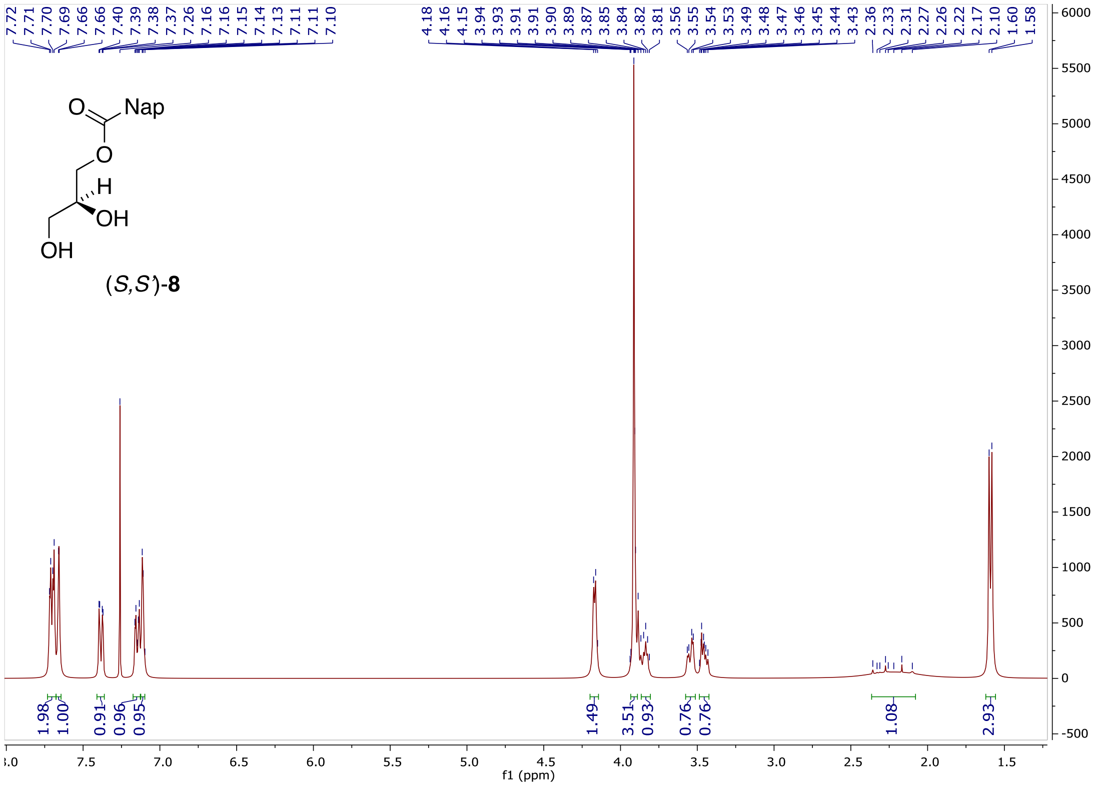

^{1}H NMR (400 MHz, CDCl$_3$) of compound **(*S,S′*)-8**

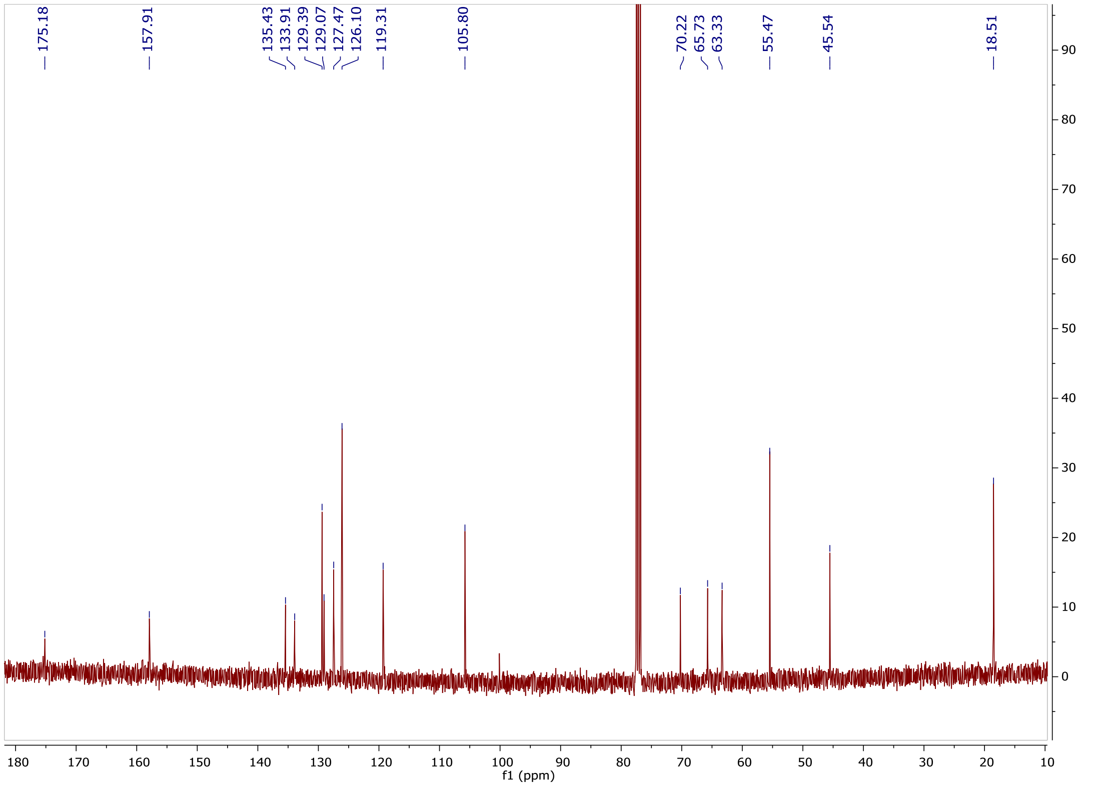

^{13}C{H} NMR (101 MHz, CDCl$_3$) of compound **(*S,S′*)-8**

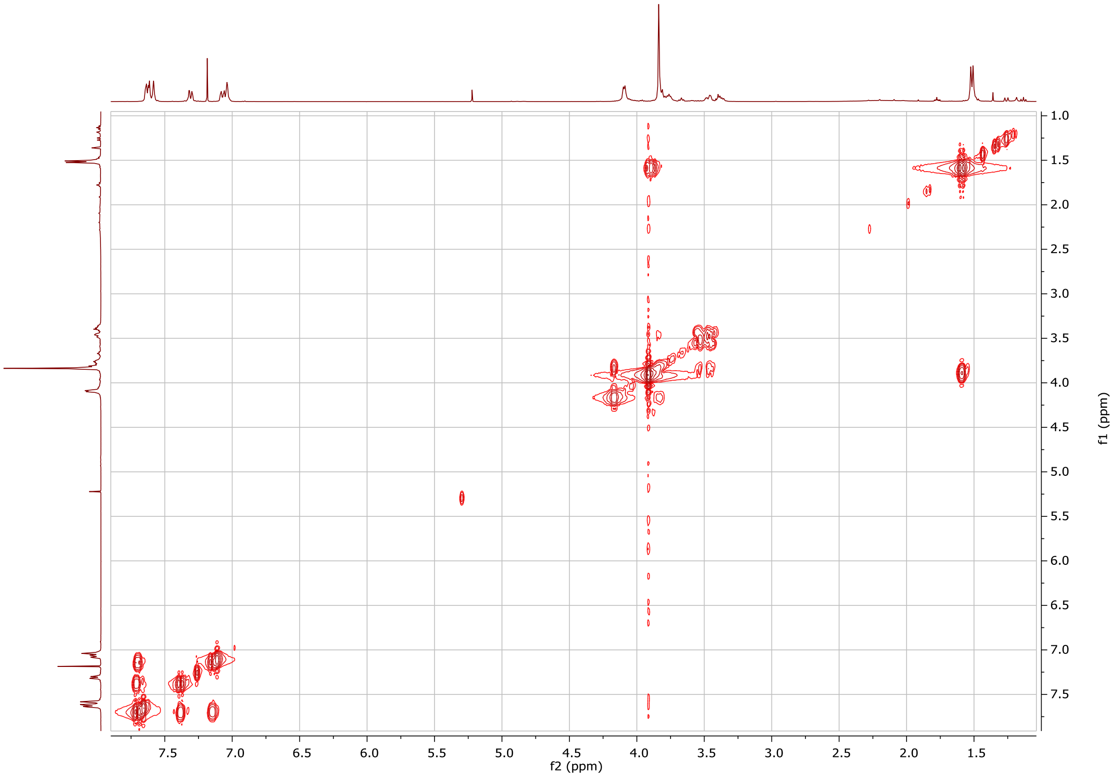

^1H-^1H COSY of compound **(*S,S'*)-8**

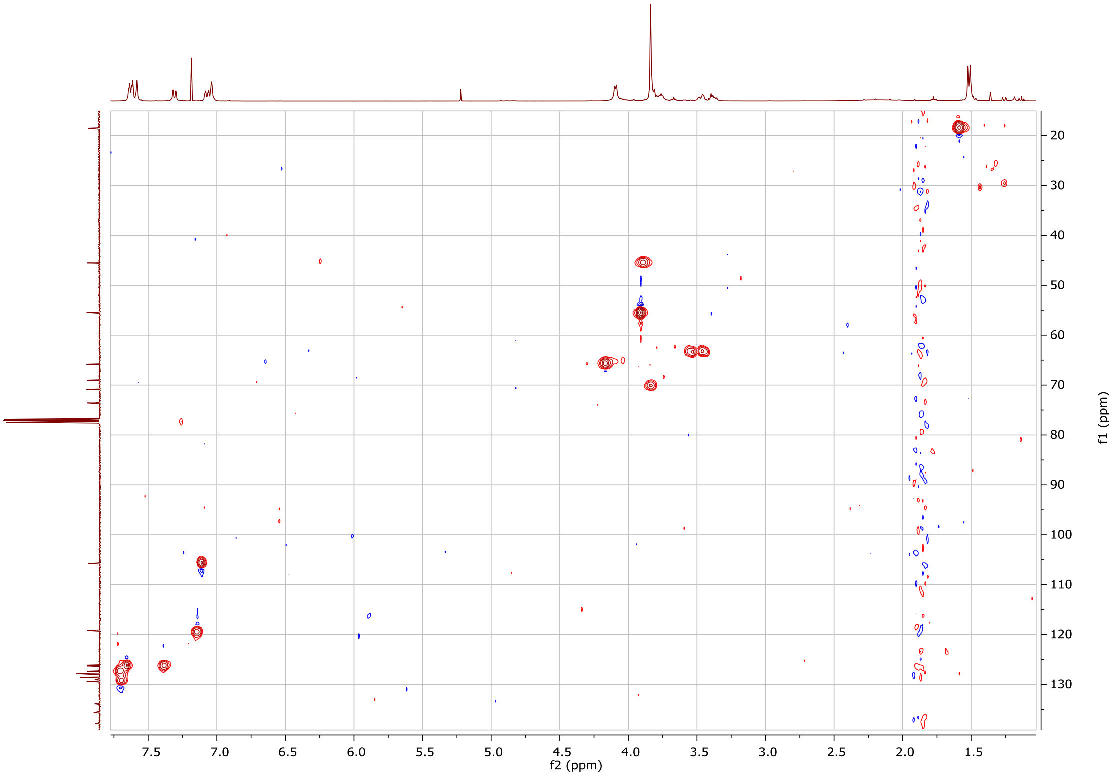

^{13}C-^1H HSQC of compound **(*S,S'*)-8**

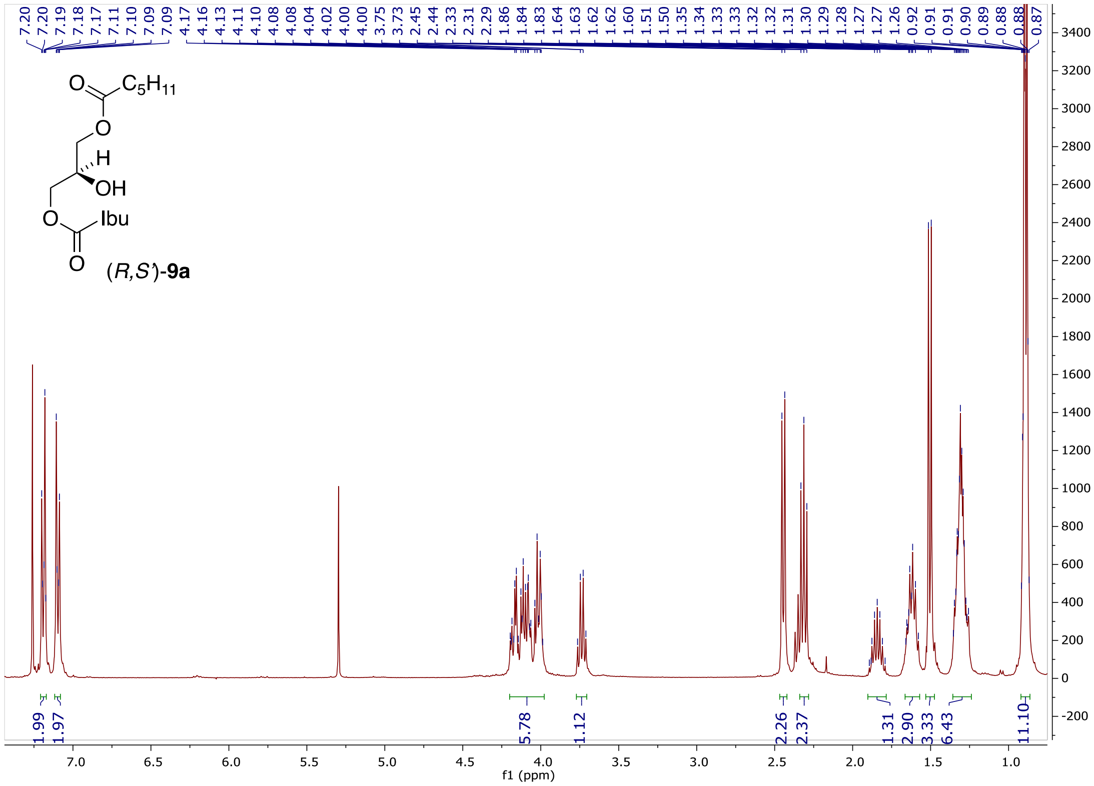

^{1}H NMR (400 MHz, $CDCl_3$) of compound **(*R*,*S′*)-9a**

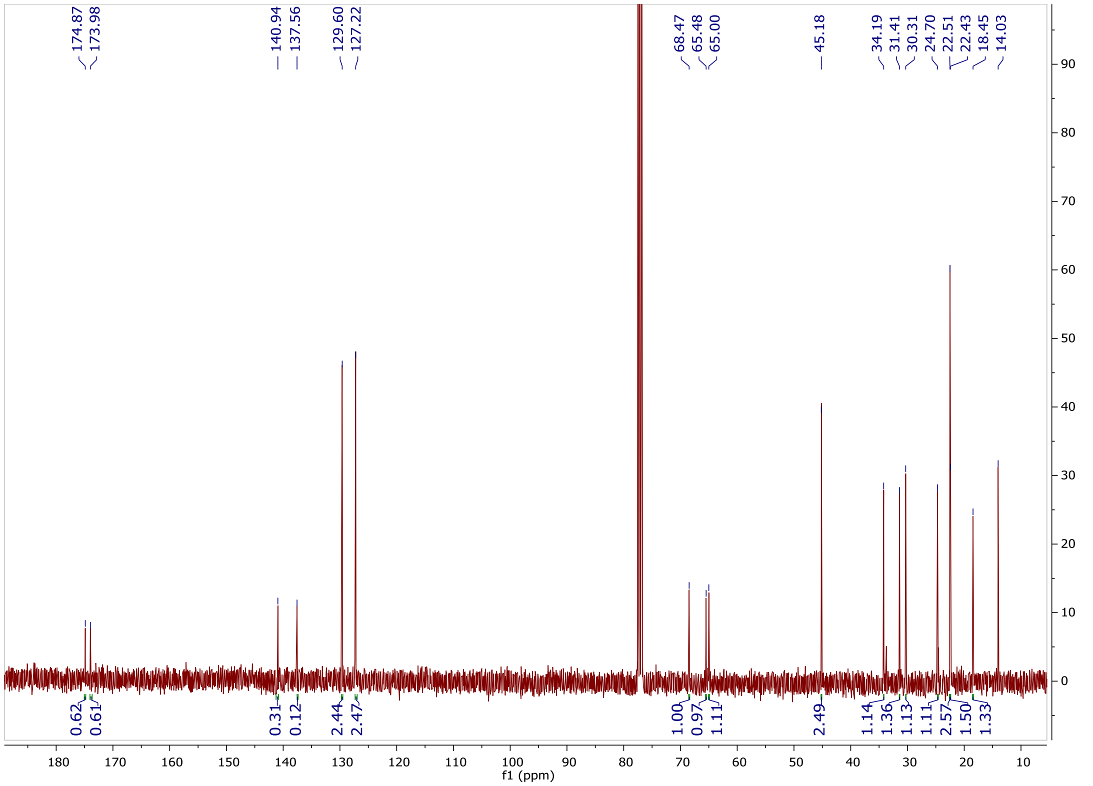

^{13}C{H} NMR (101 MHz, $CDCl_3$) of compound **(*R*,*S′*)-9a**

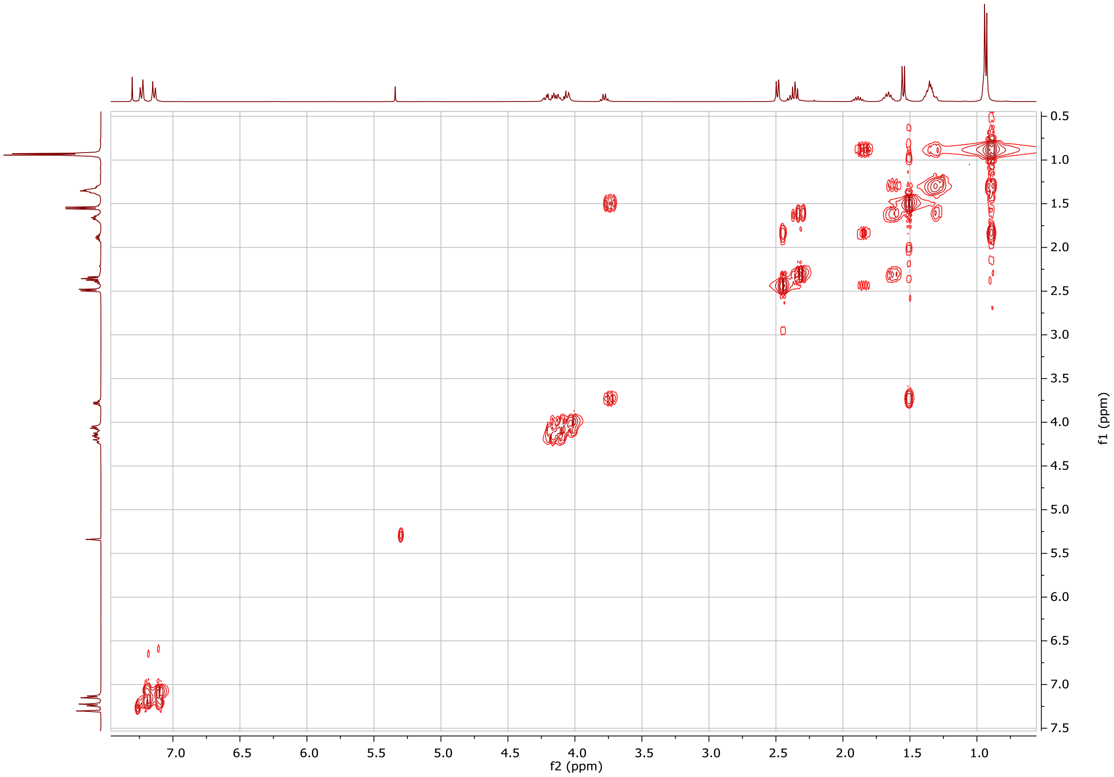

^{1}H-^{1}H COSY of compound **(*R*,*S*′)-9a**

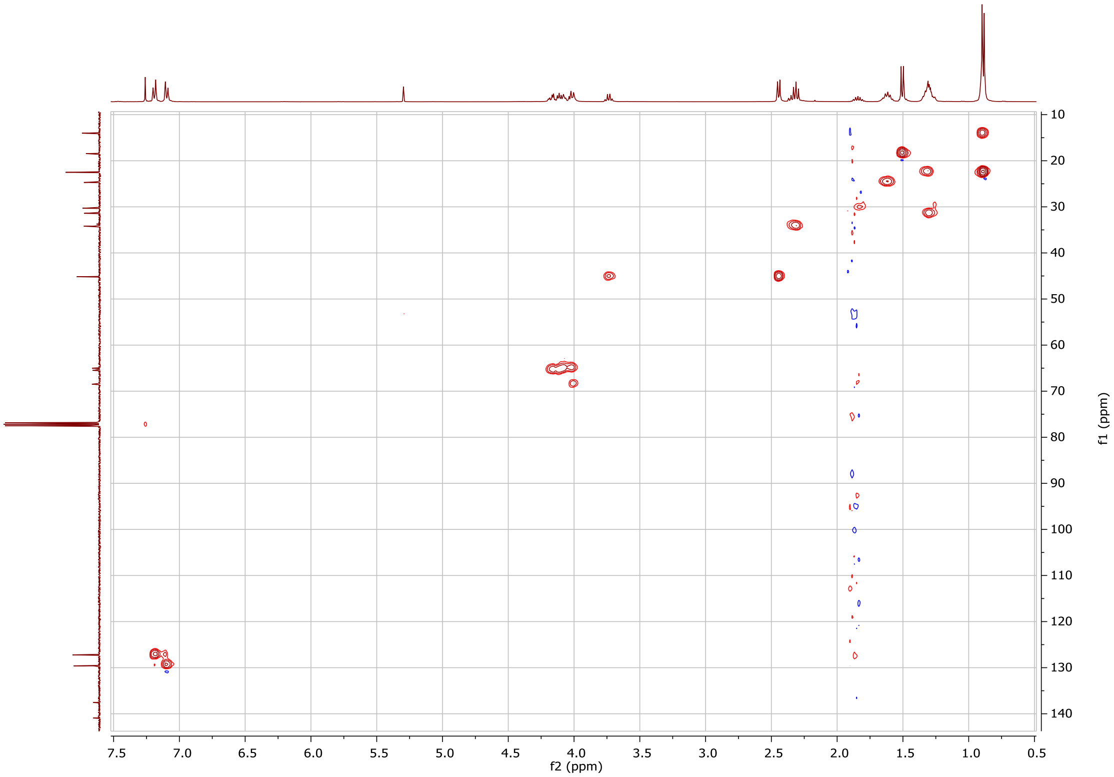

^{13}C-^{1}H HSQC of compound **(*R*,*S*′)-9**

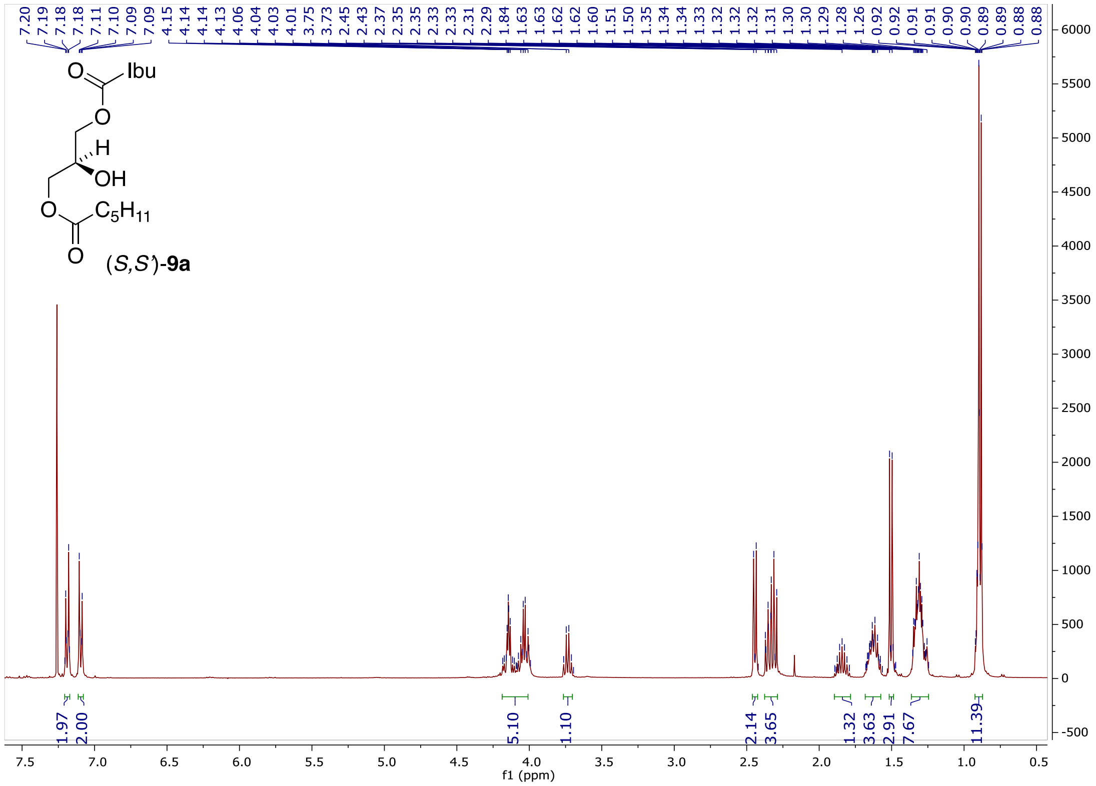

^{1}H NMR (400 MHz, $CDCl_3$) of compound **(*S,S′*)-9a**

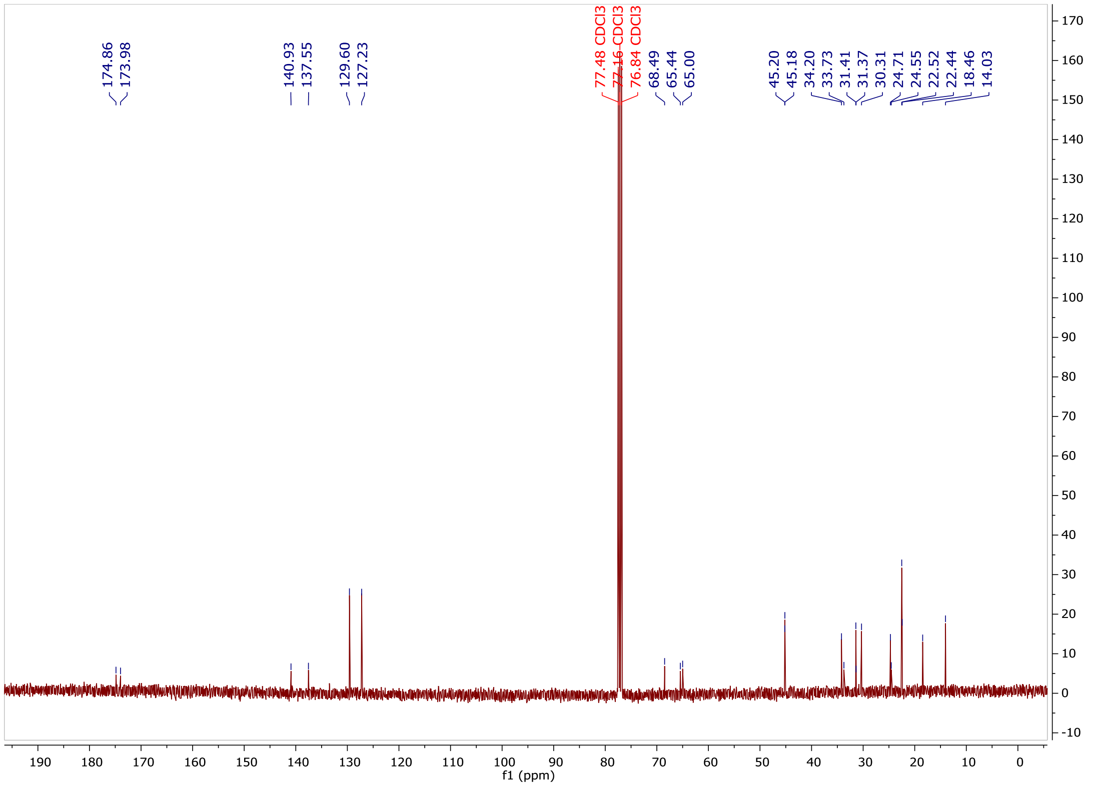

^{13}C{H} NMR (101 MHz, $CDCl_3$) of compound **(*S,S′*)-9a**

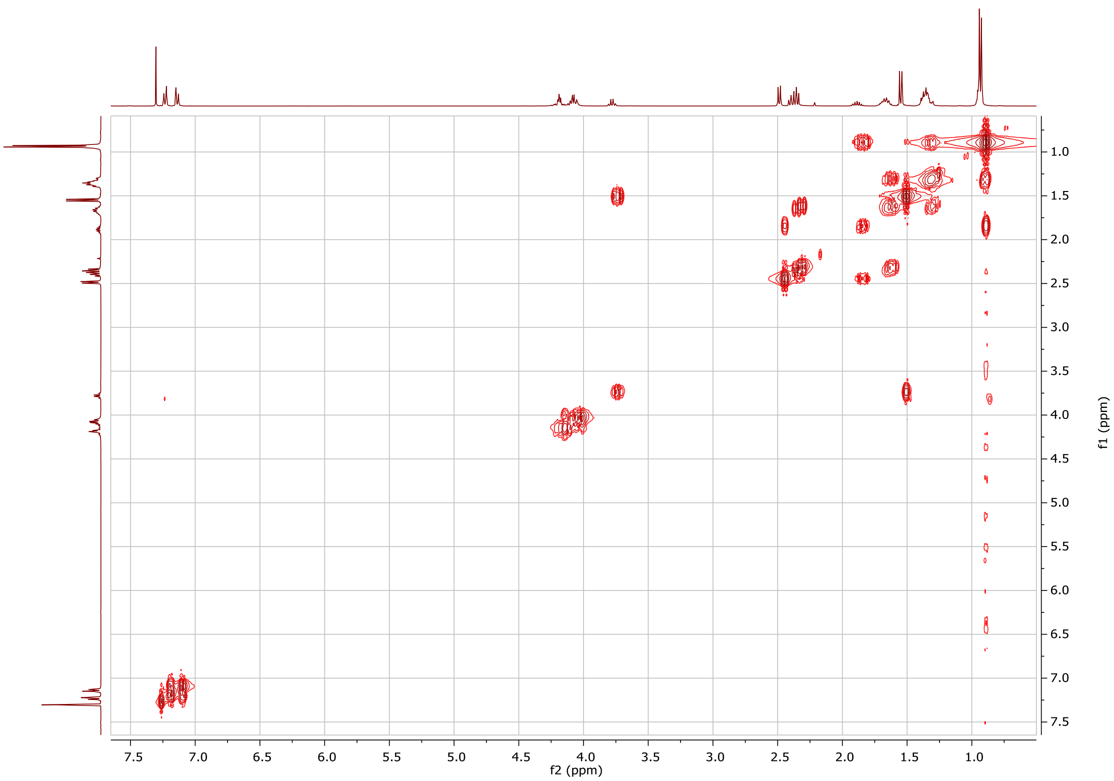

^{1}H-^{1}H COSY of compound **(*S*,*S′*)-9a**

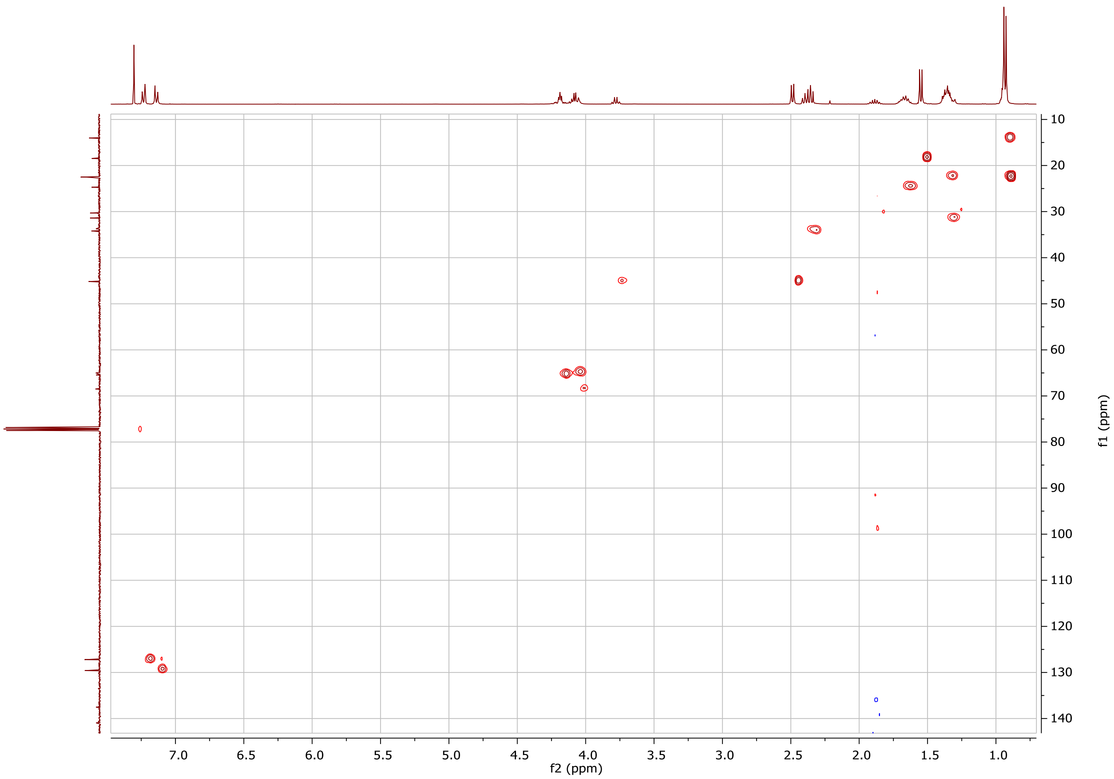

^{13}C-^{1}H HSQC of compound **(*S*,*S′*)-9a**

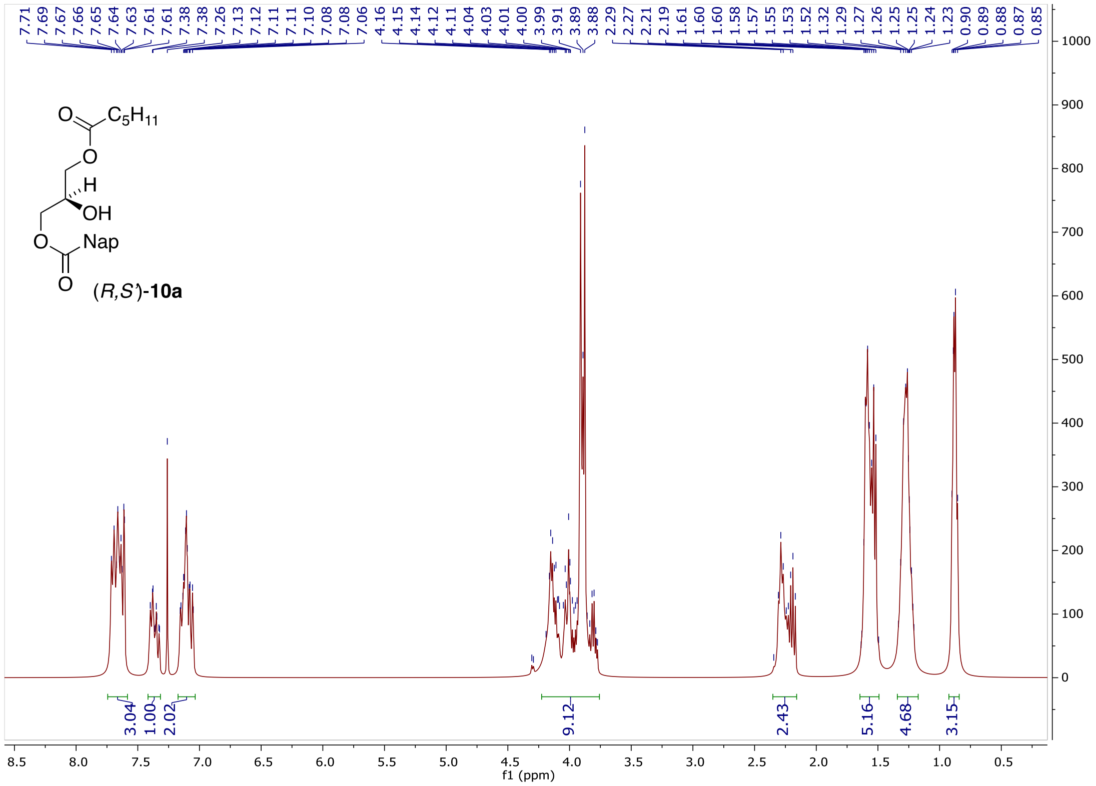

^{1}H NMR (400 MHz, $CDCl_3$) of compound ***(R,S′)*-10a**

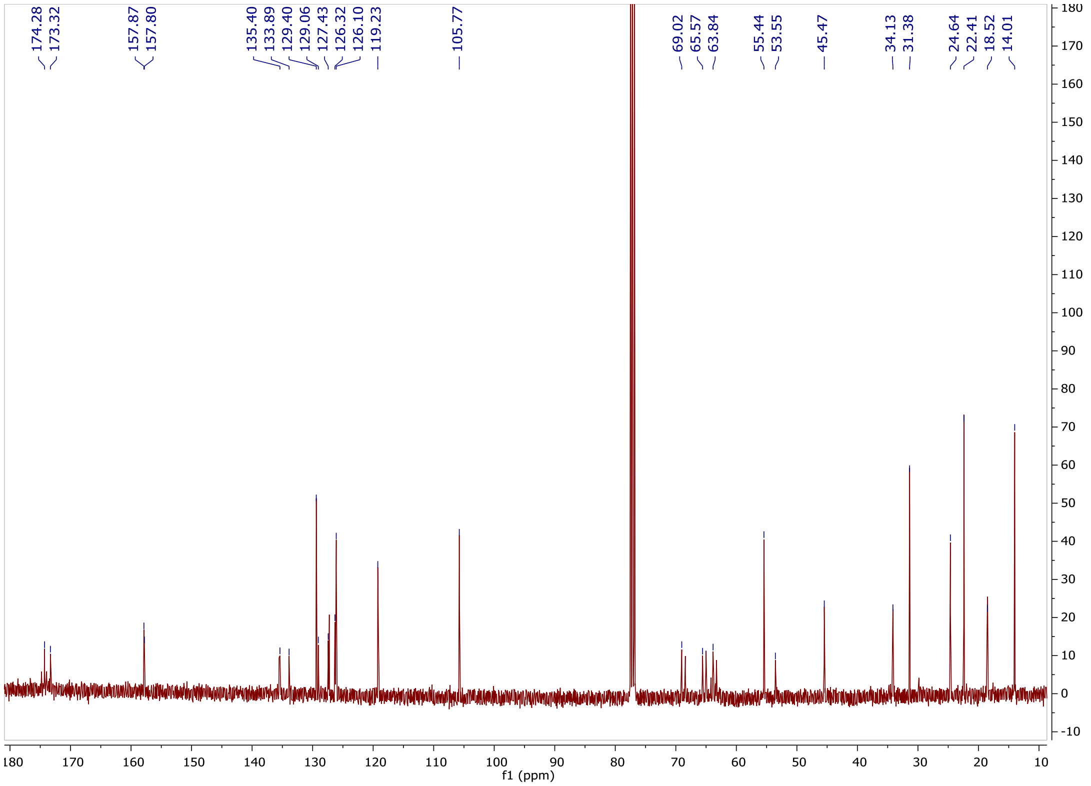

^{13}C{H} NMR (101 MHz, $CDCl_3$) of compound ***(R,S′)*-10a**

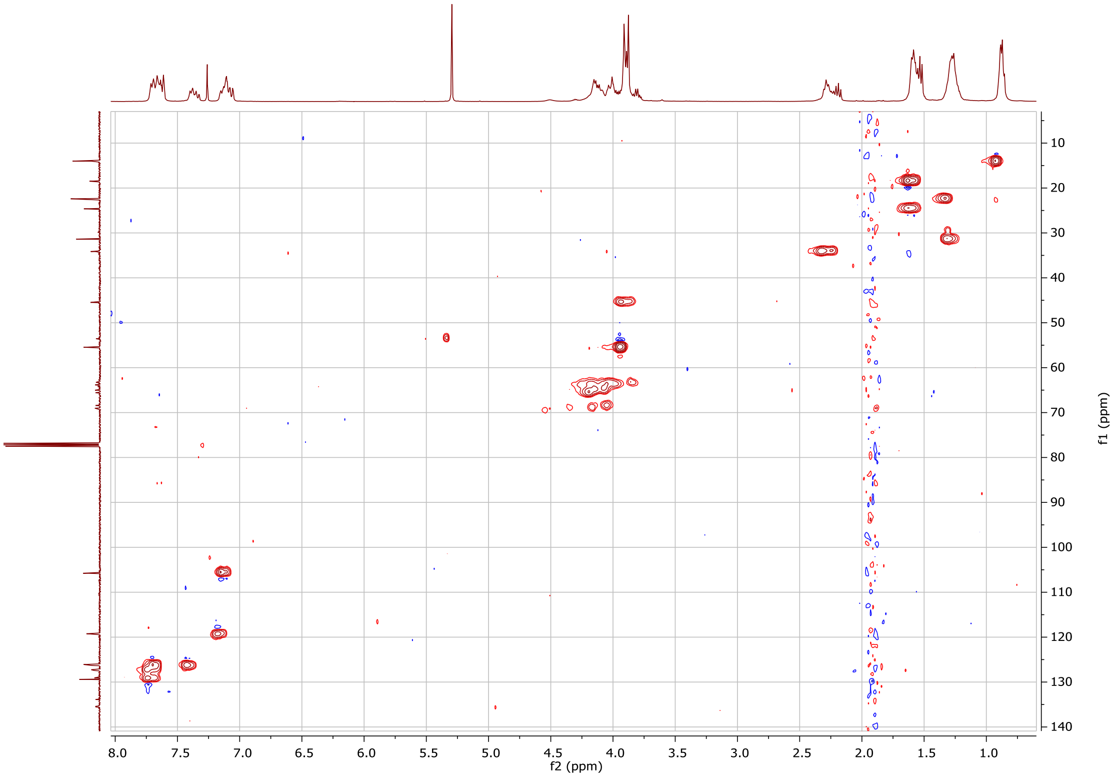

^{1}H-^{1}H COSY of compound **(*R*,*S′*)-10a**

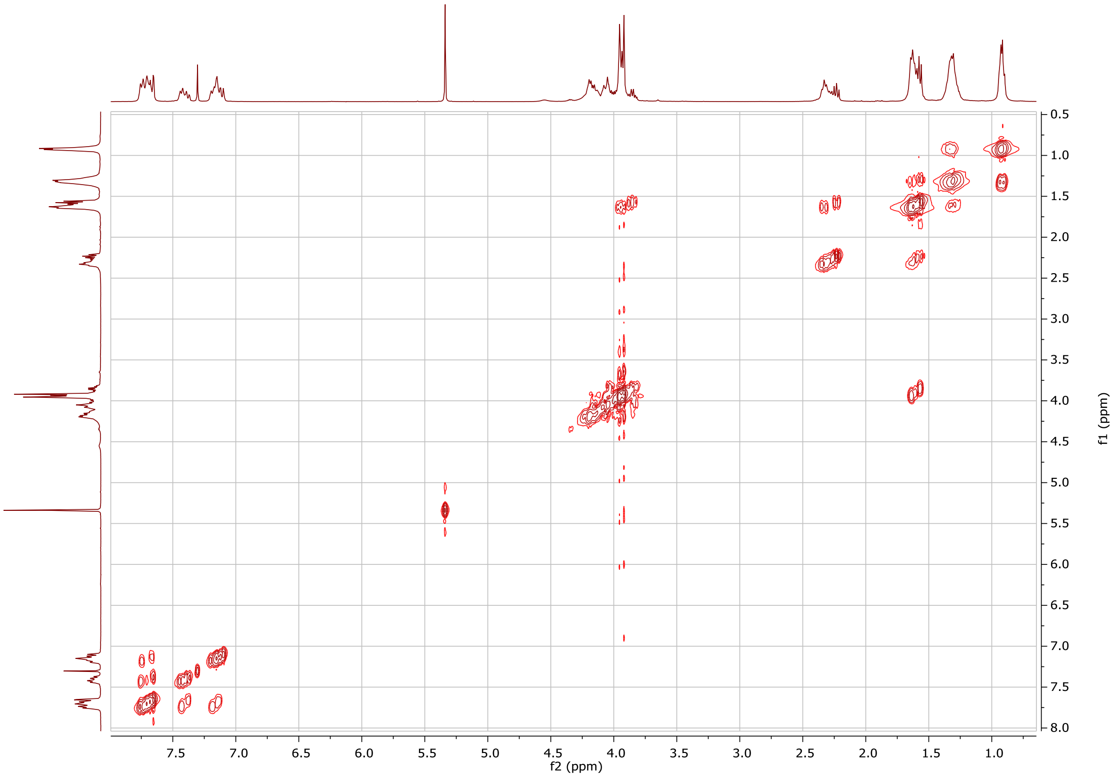

^{13}C-^{1}H HSQC of compound **(*R*,*S′*)-10a**

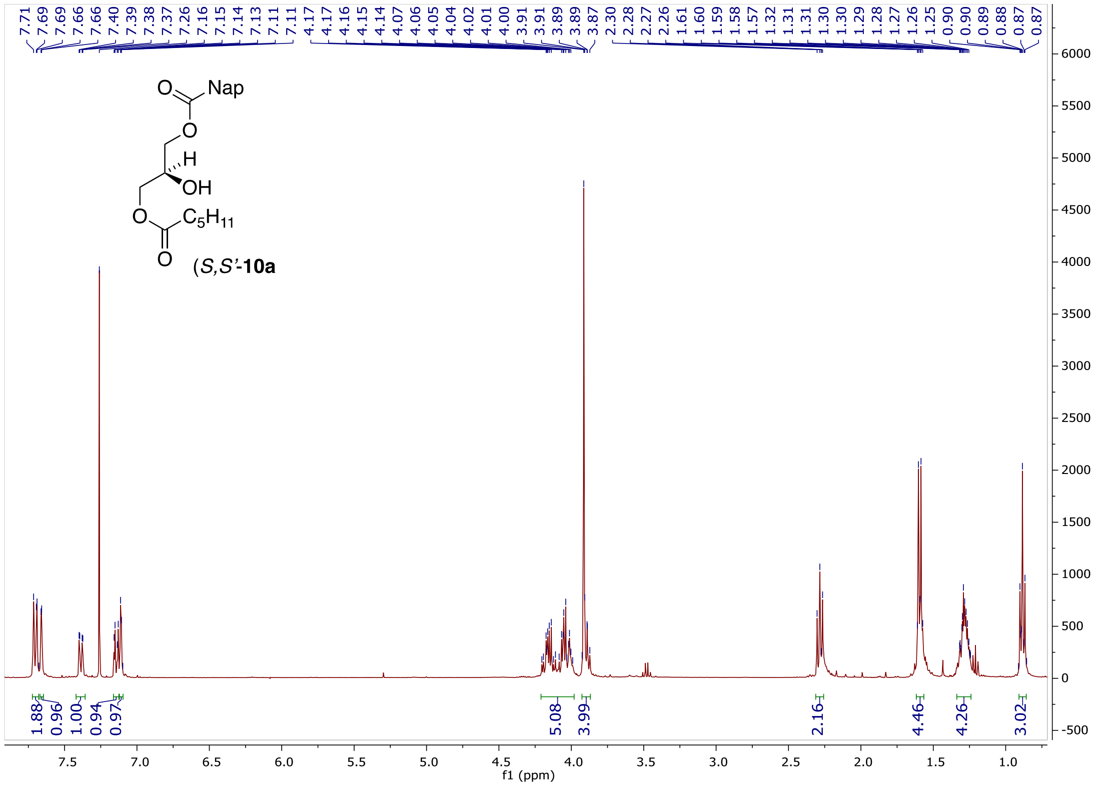

^{1}H NMR (400 MHz, $CDCl_3$) of compound **(*S*,*S′*)-10a**

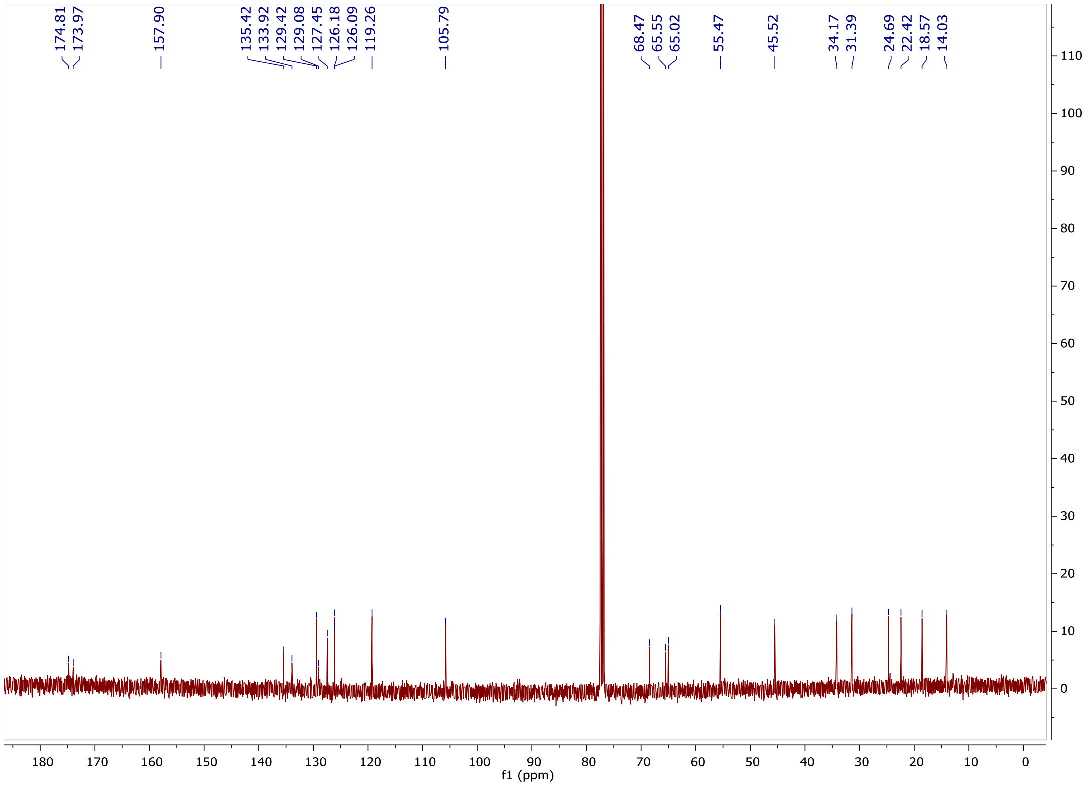

^{13}C{H} NMR (101 MHz, $CDCl_3$) of compound **(*S*,*S′*)-10a**

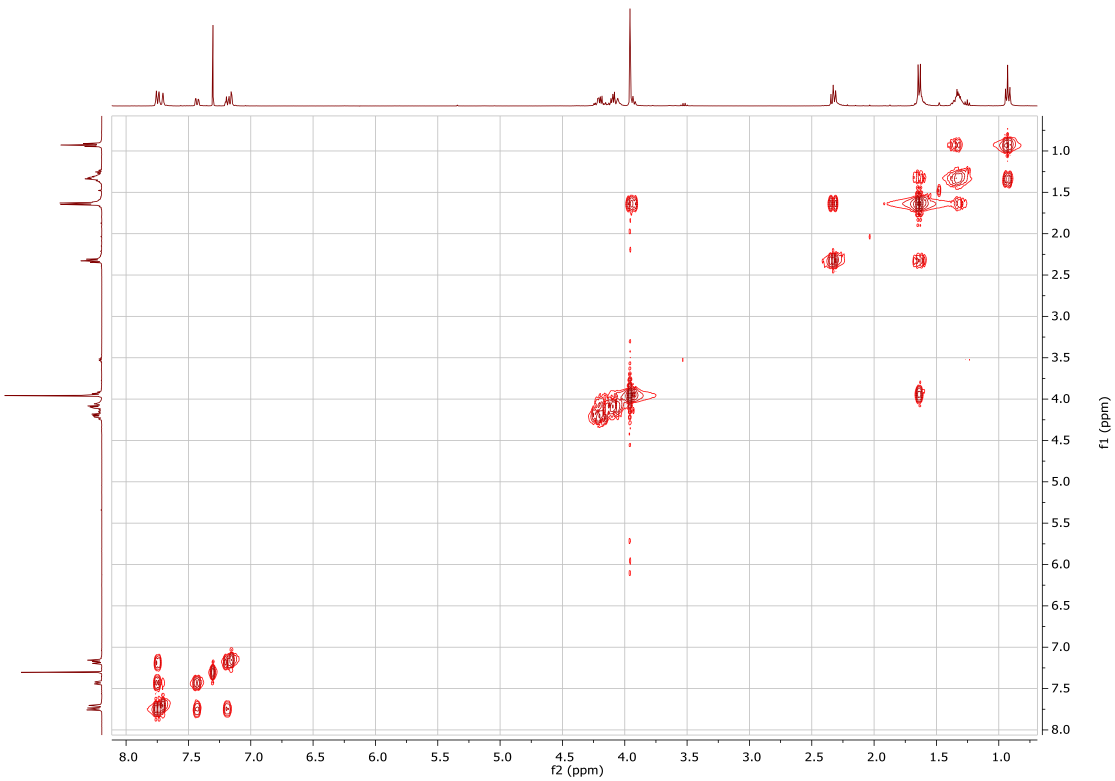

^{1}H-^{1}H COSY of compound **(*S*,*S'*)-10a**

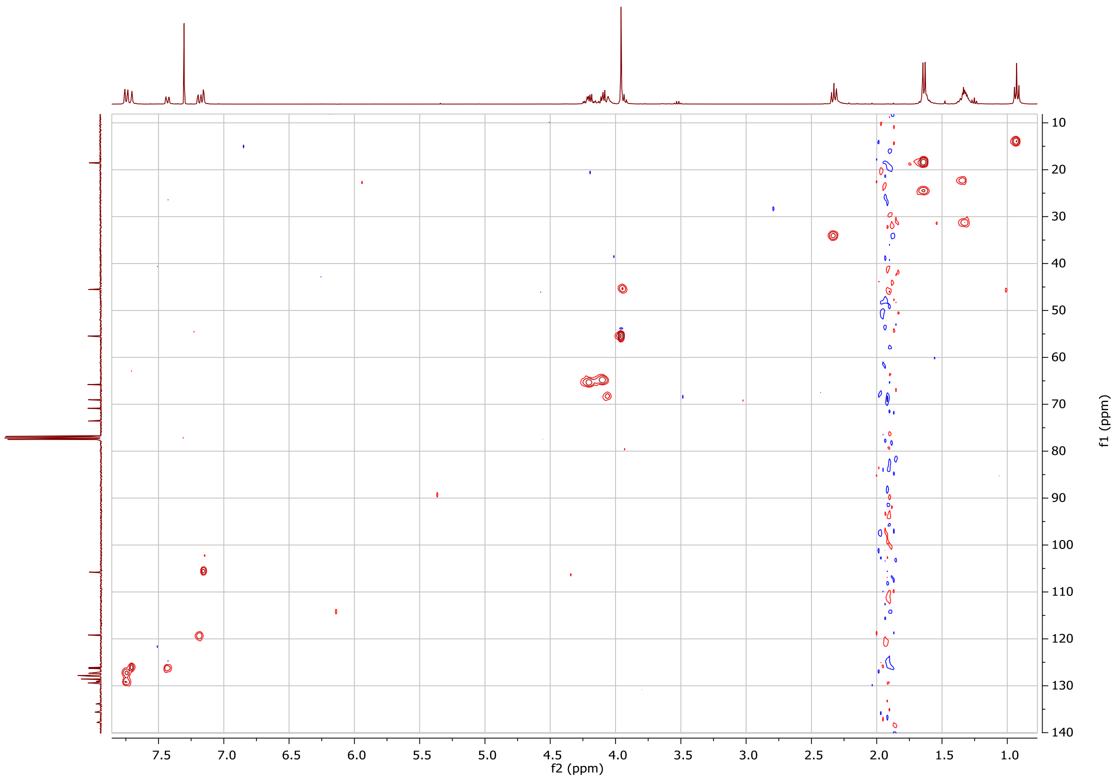

^{13}C-^{1}H HSQC of compound **(*S*,*S'*)-10a**

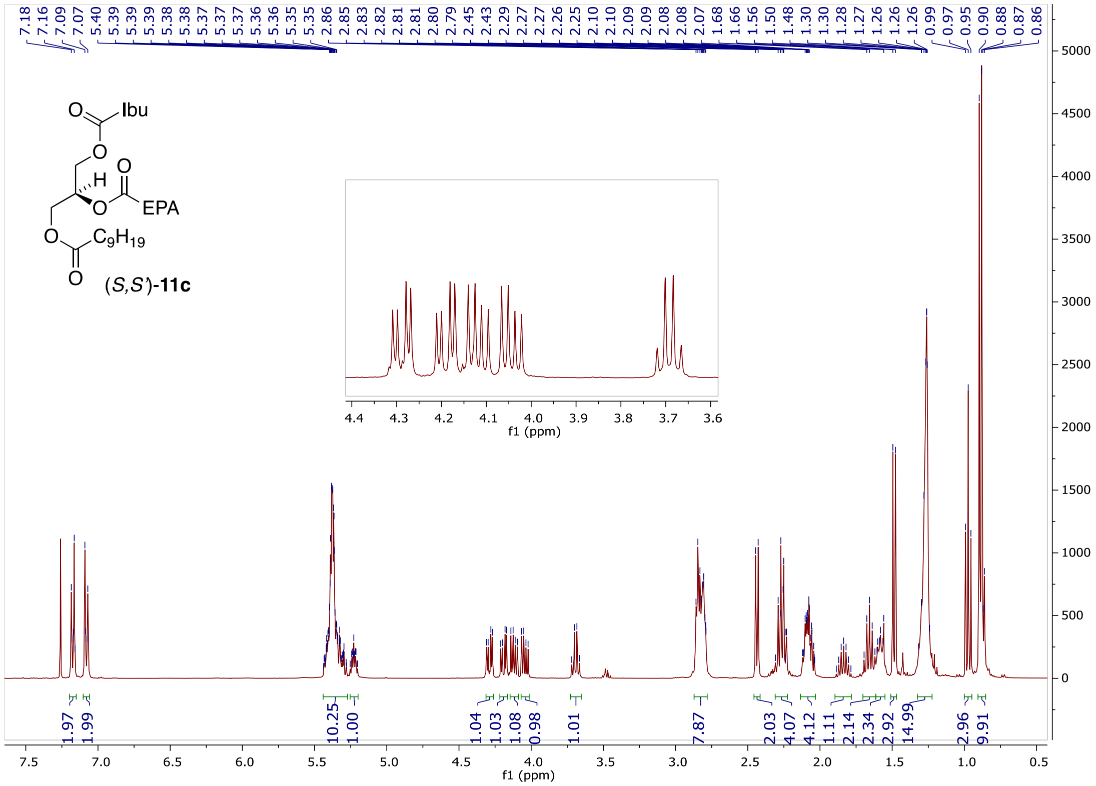

^{1}H NMR (400 MHz, $CDCl_3$) of compound **(*S*,*S′*)-11c**

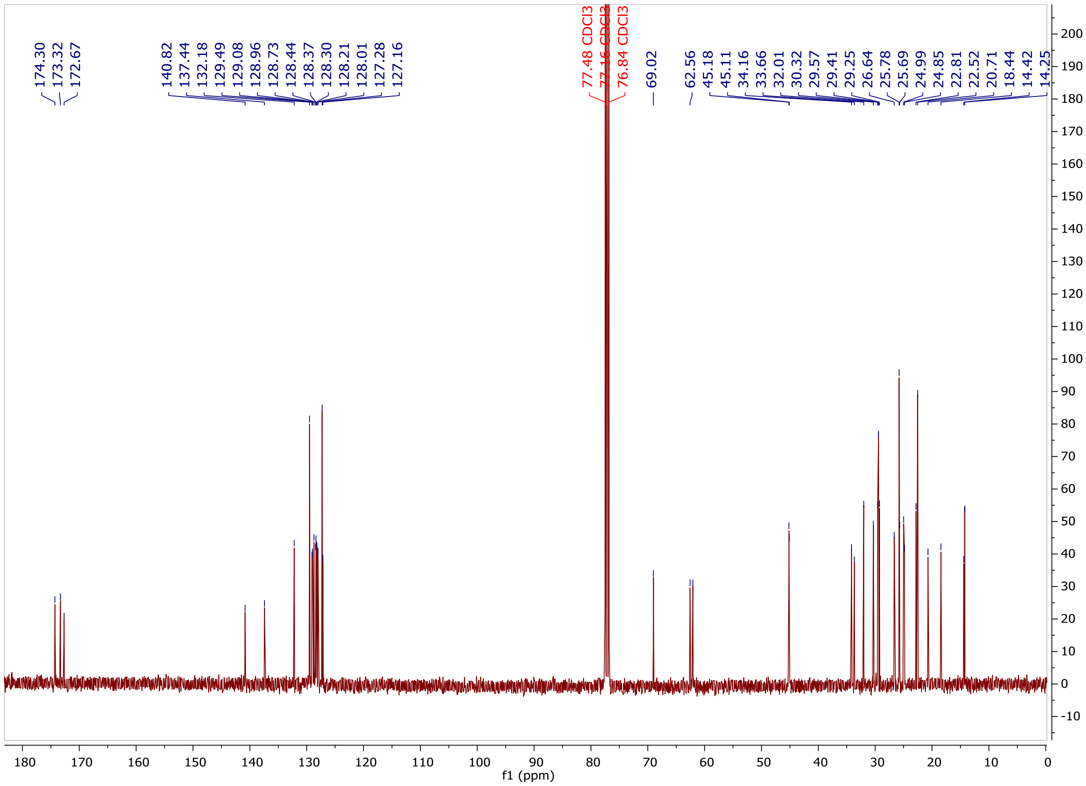

^{13}C{H} NMR (101 MHz, $CDCl_3$) of compound **(*S*,*S′*)-11c**

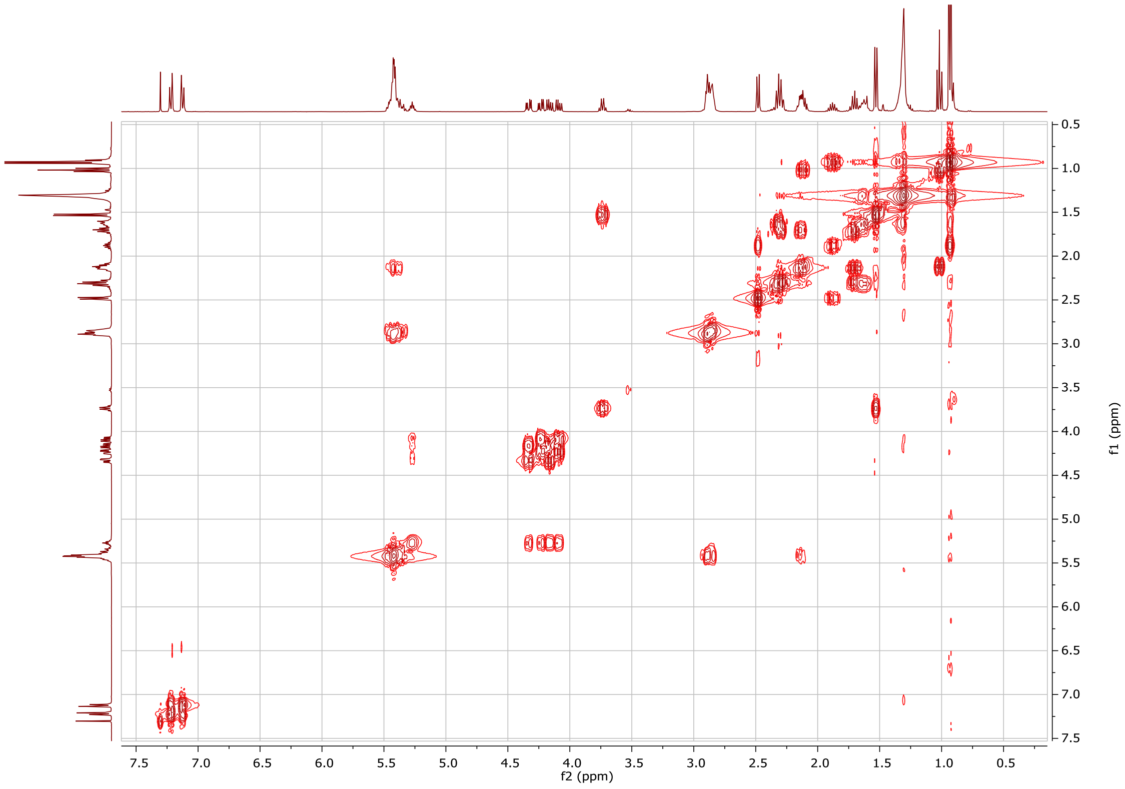

^{1}H-^{1}H COSY of compound **(*S*,*S′*)-11c**

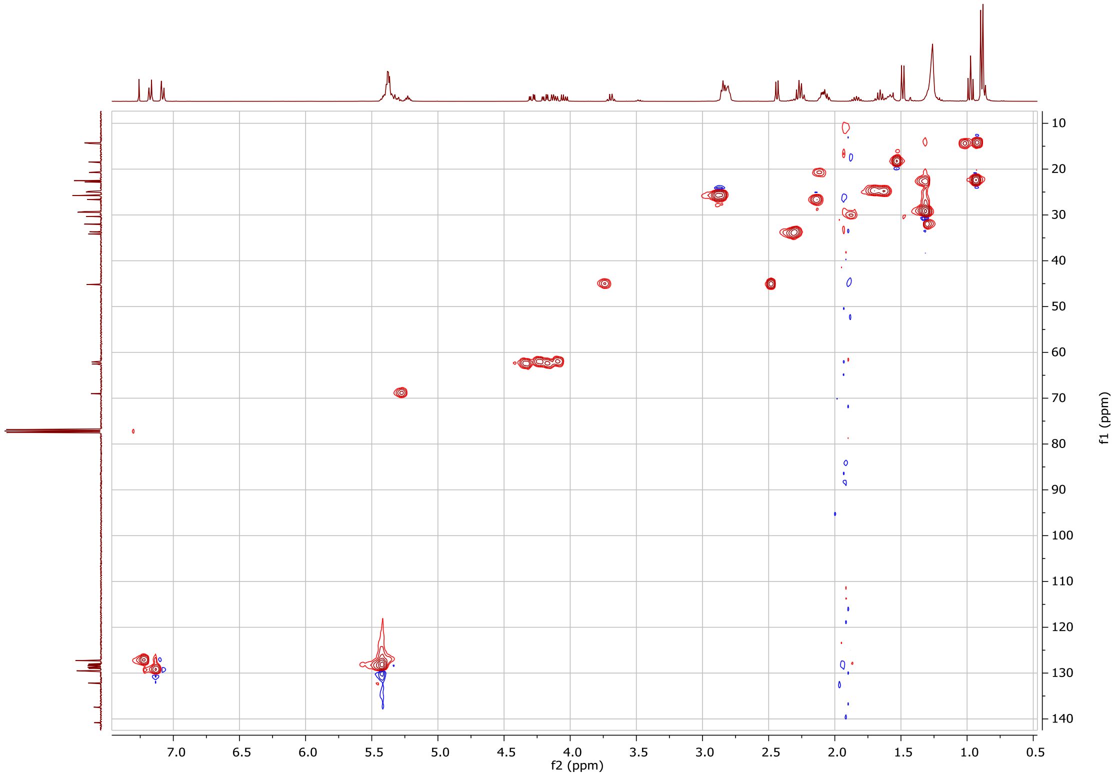

^{13}C-^{1}H HSQC of compound **(*S*,*S′*)-11c**

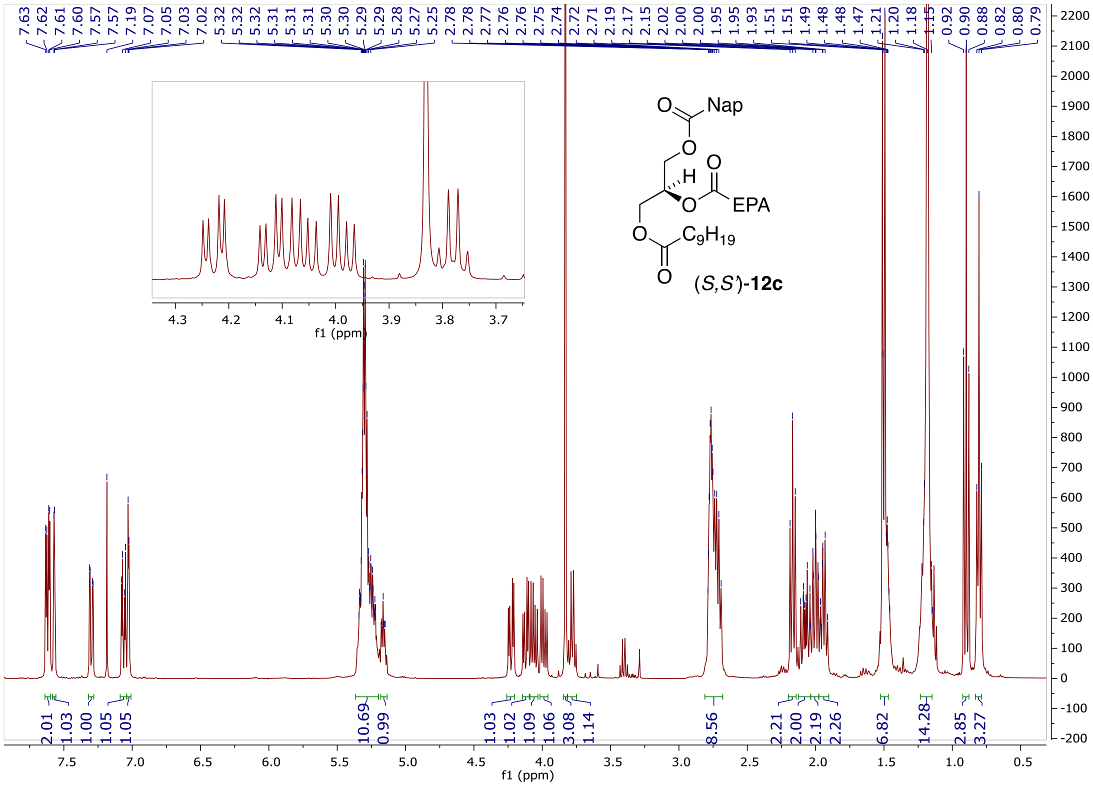

^{1}H NMR (400 MHz, $CDCl_3$) of compound **(*S,S'*)-12c**

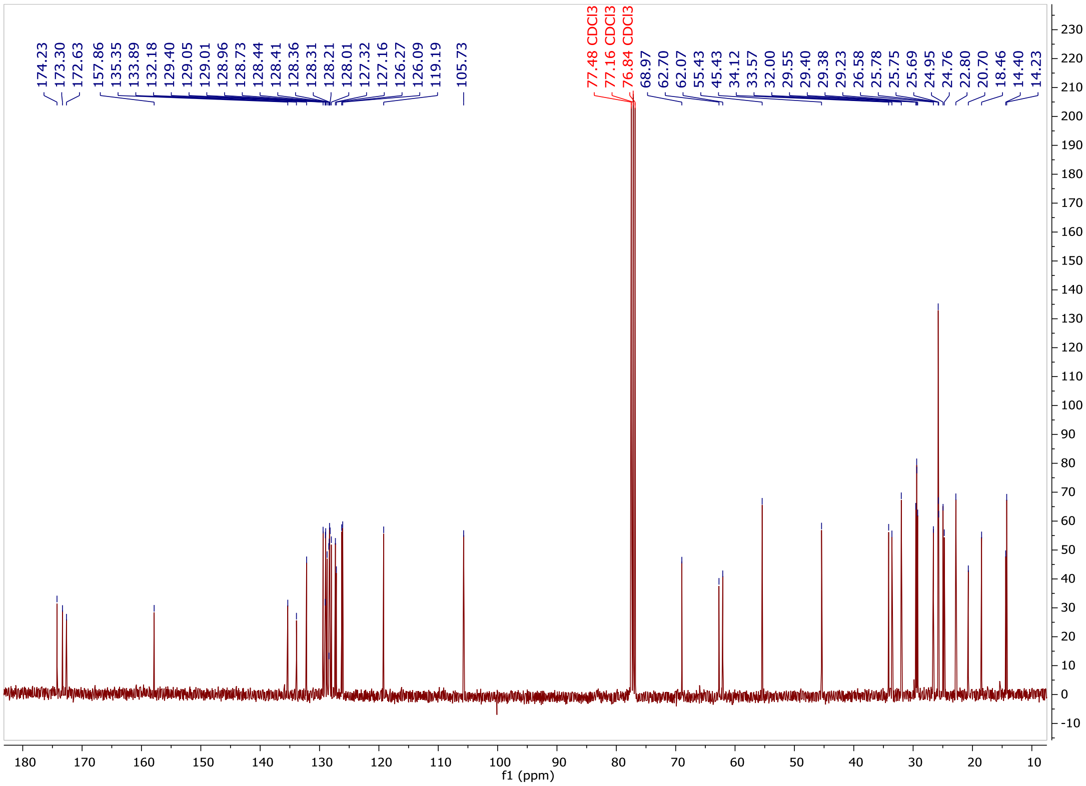

^{13}C{H} NMR (101 MHz, $CDCl_3$) of compound **(*S,S'*)-12c**

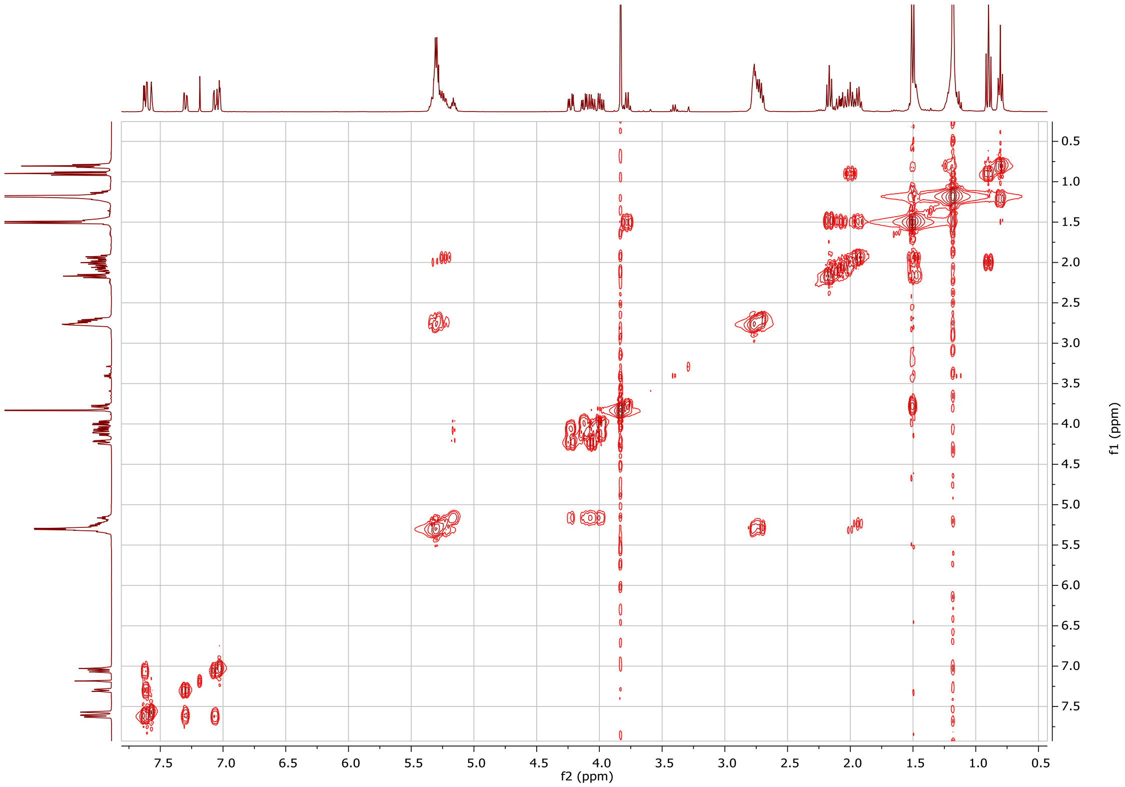

^{1}H-^{1}H COSY of compound **(*S,S′*)-12c**

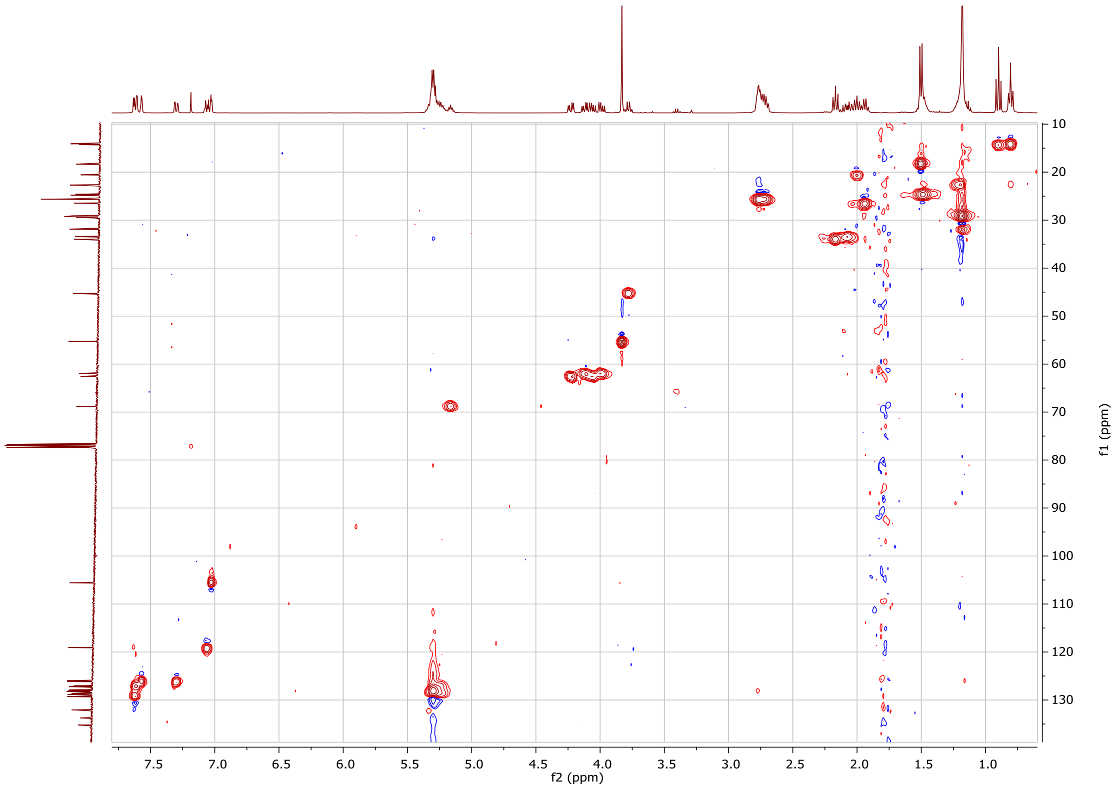

^{13}C-^{1}H HSQC of compound **(*S,S′*)-12c**

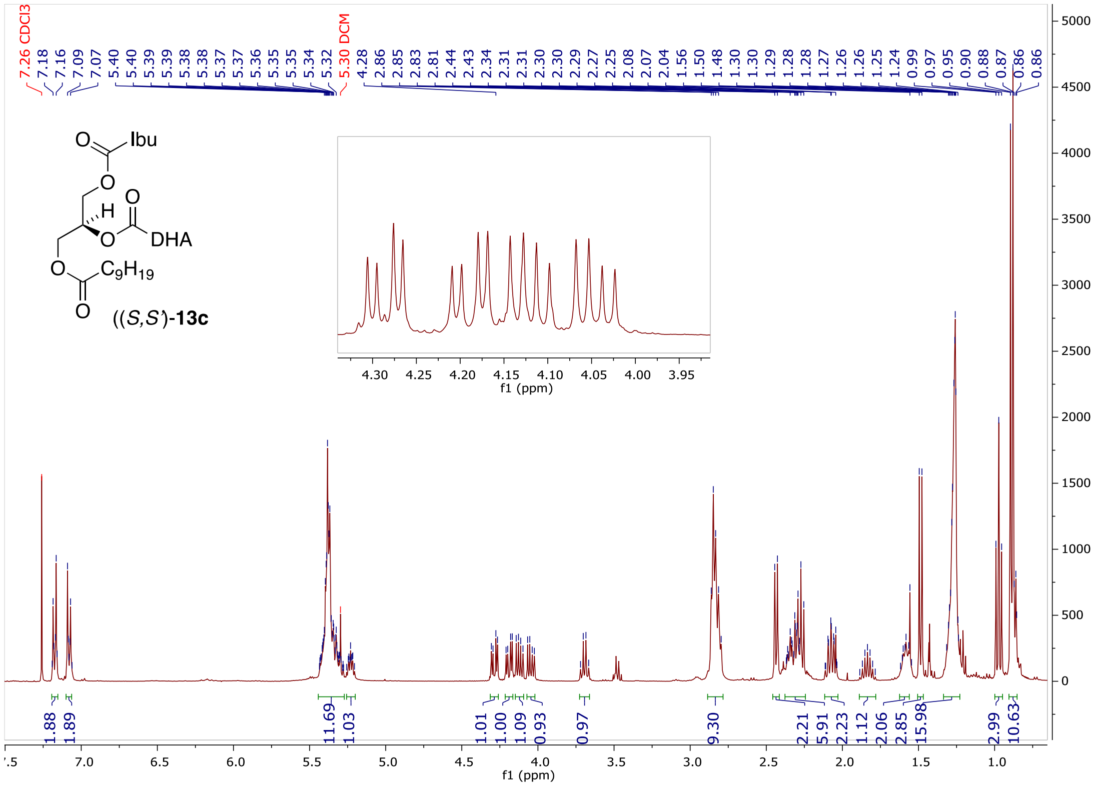

^{1}H NMR (400 MHz, $CDCl_3$) of compound **(*S,S′*)-13c**

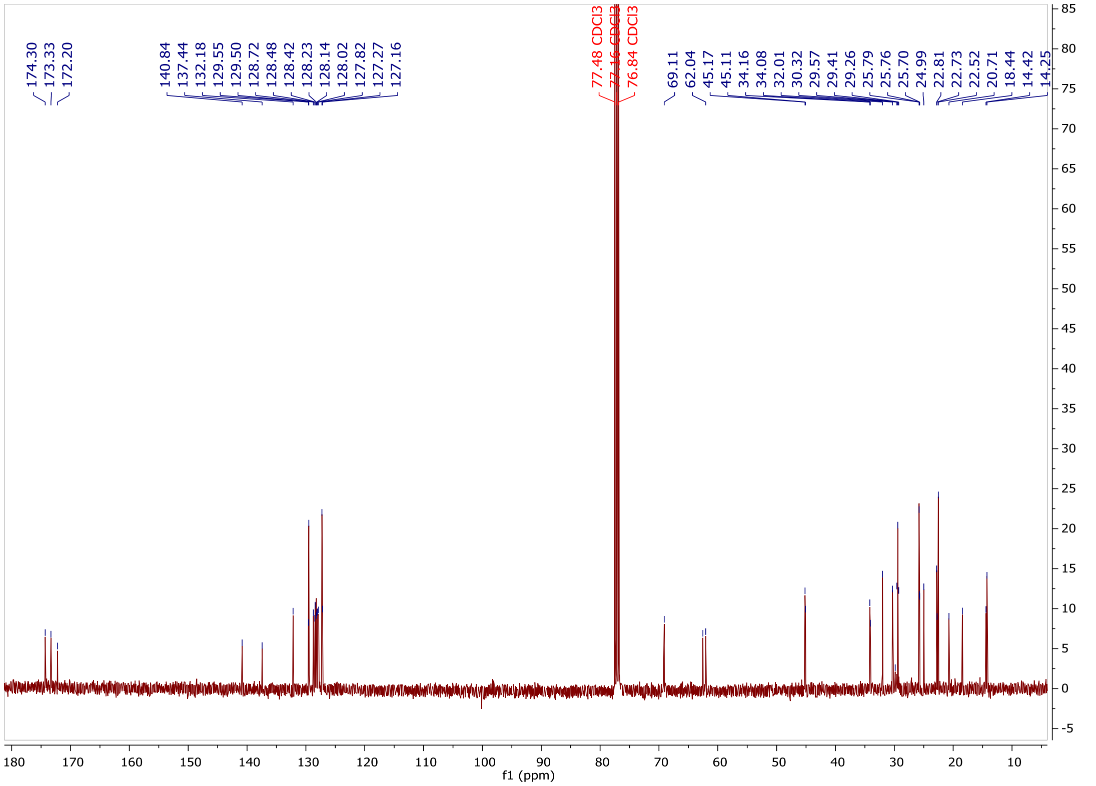

^{13}C{H} NMR (101 MHz, $CDCl_3$) of compound **(*S,S′*)-13c**

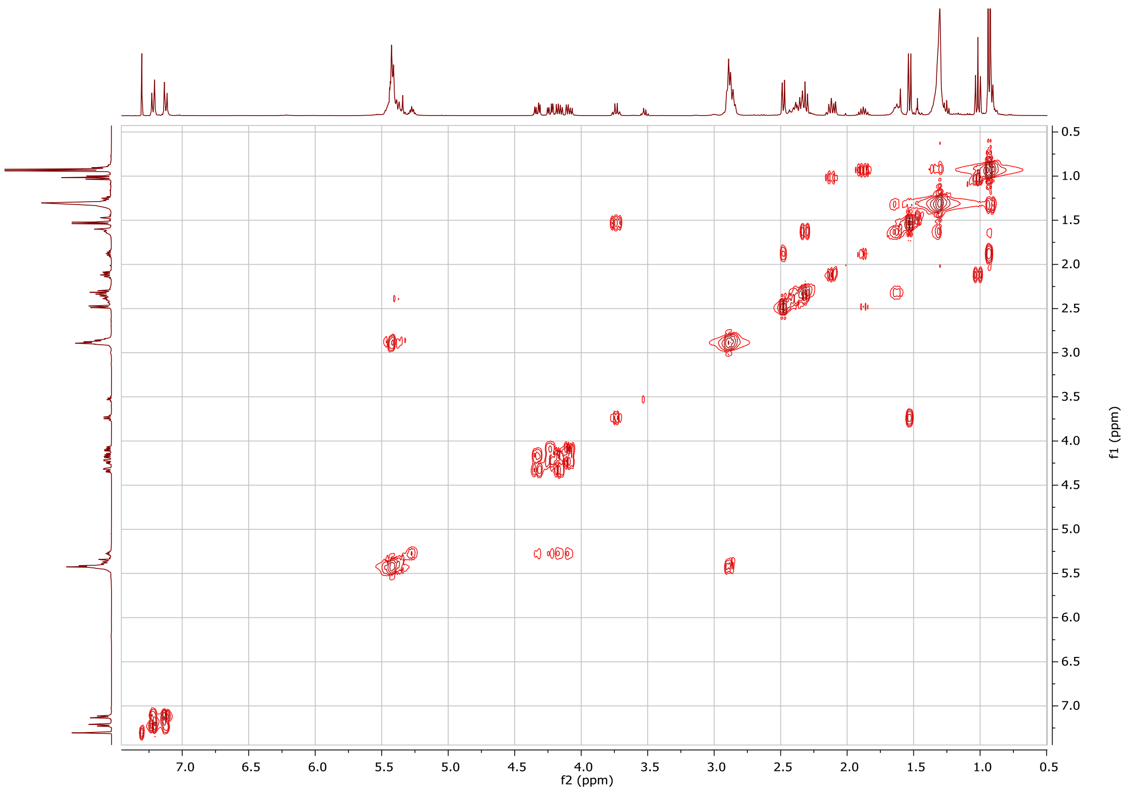

^{1}H-^{1}H COSY of compound **(*S*,*S'*)-13c**

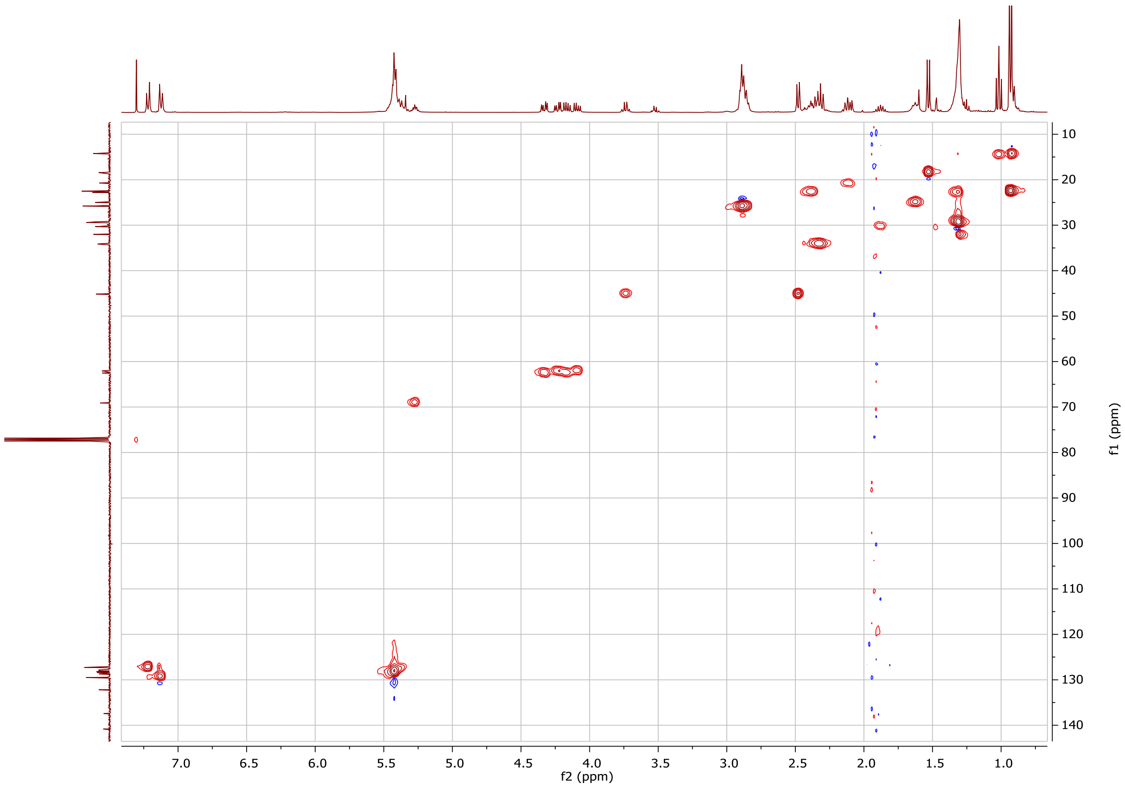

^{13}C-^{1}H HSQC of compound **(*S*,*S'*)-13c**

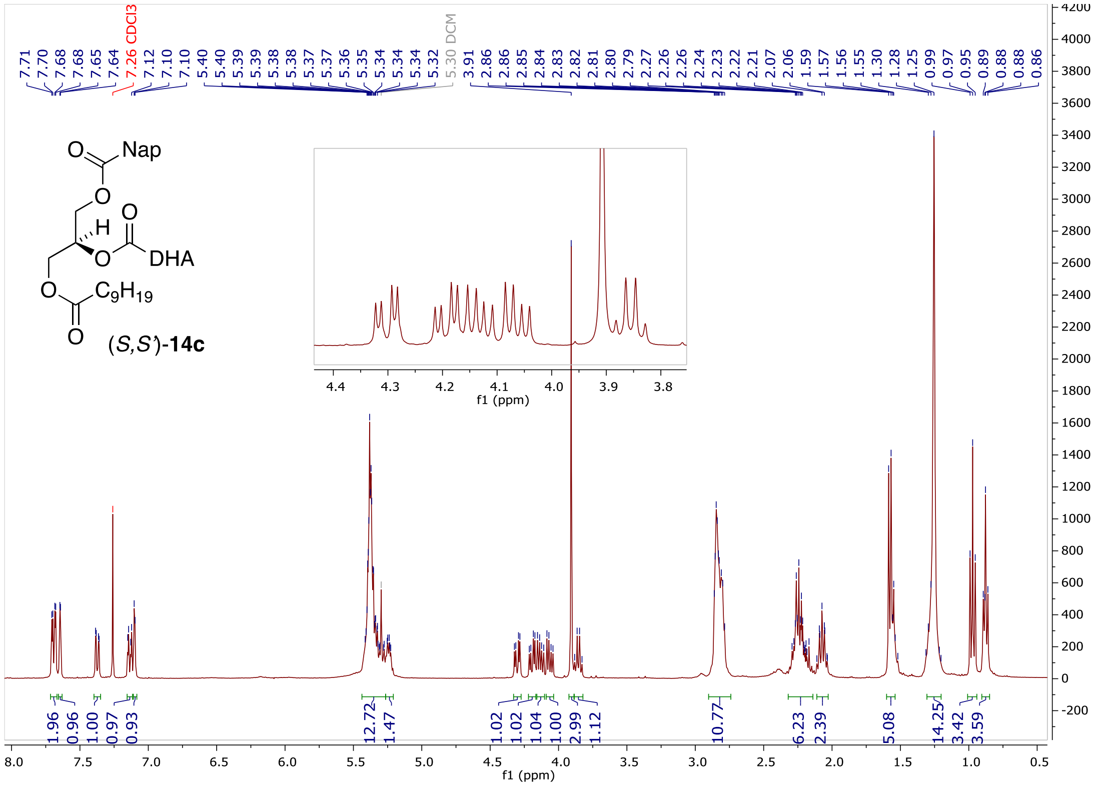

^{1}H NMR (400 MHz, $CDCl_3$) of compound **(*S*,*S'*)-14c**

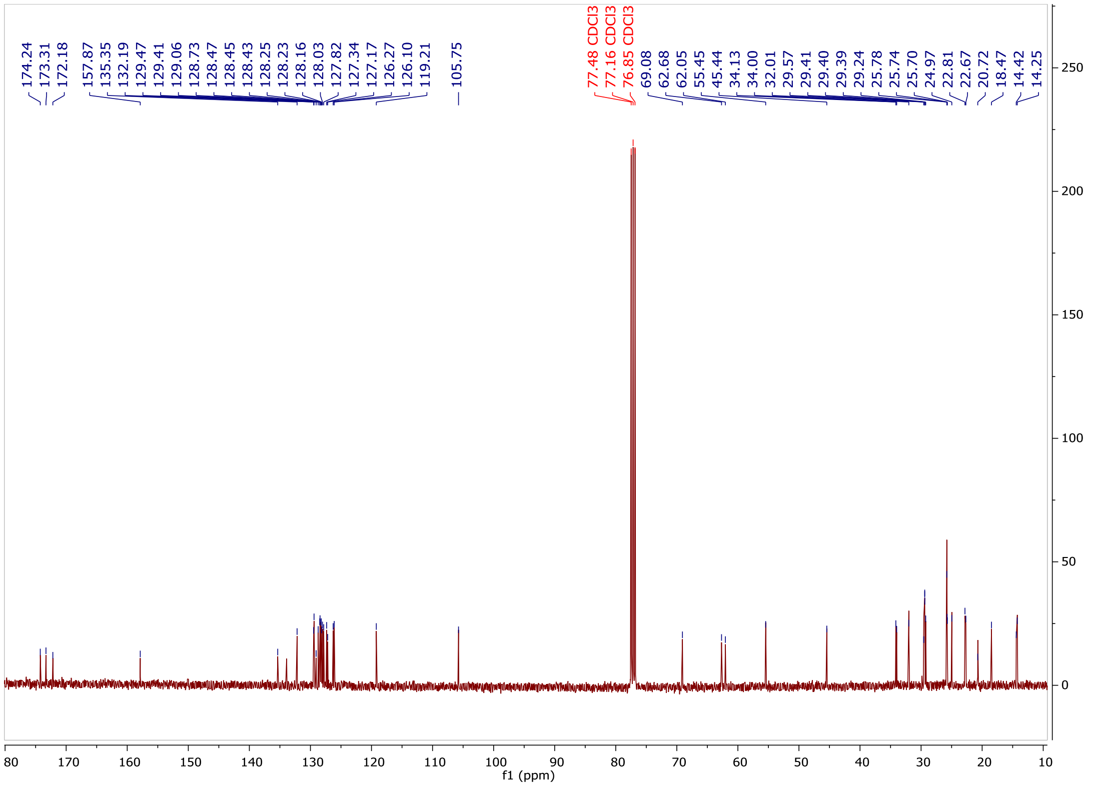

$^{13}C\{H\}$ NMR (101 MHz, $CDCl_3$) of compound **(*S*,*S'*)-14c**

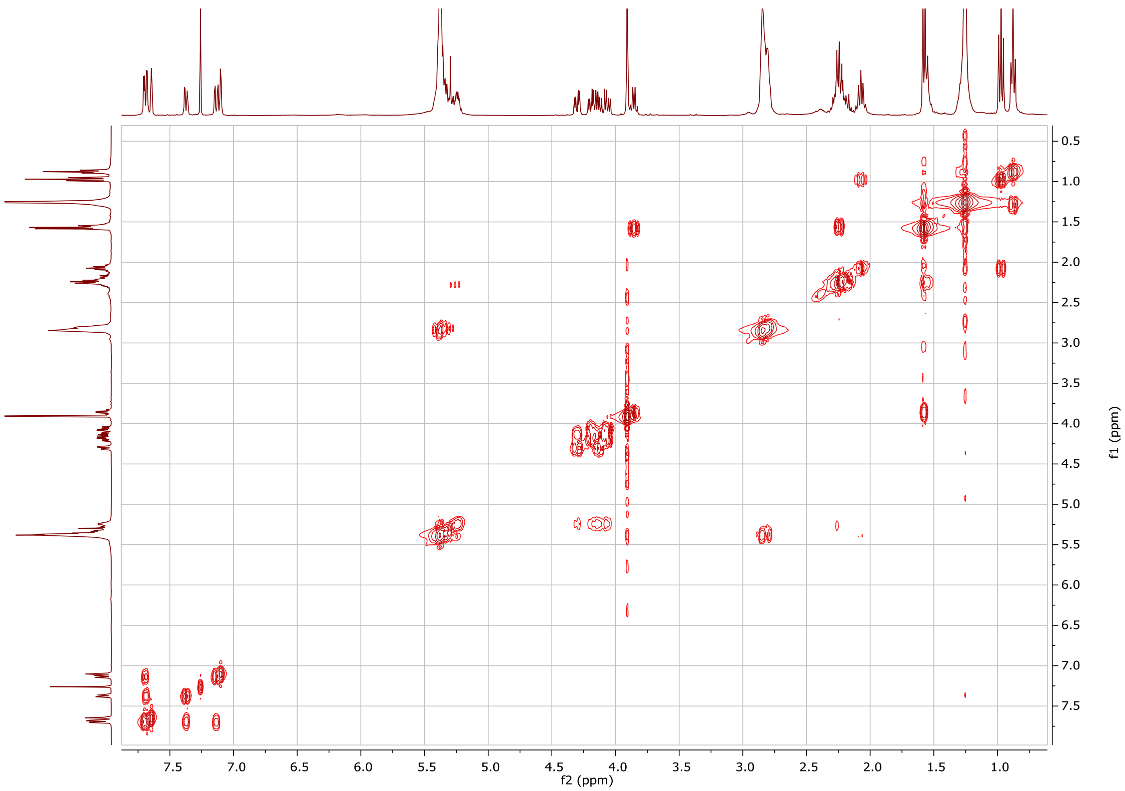

^{1}H-^{1}H COSY of compound **(*S,S′*)-14c**

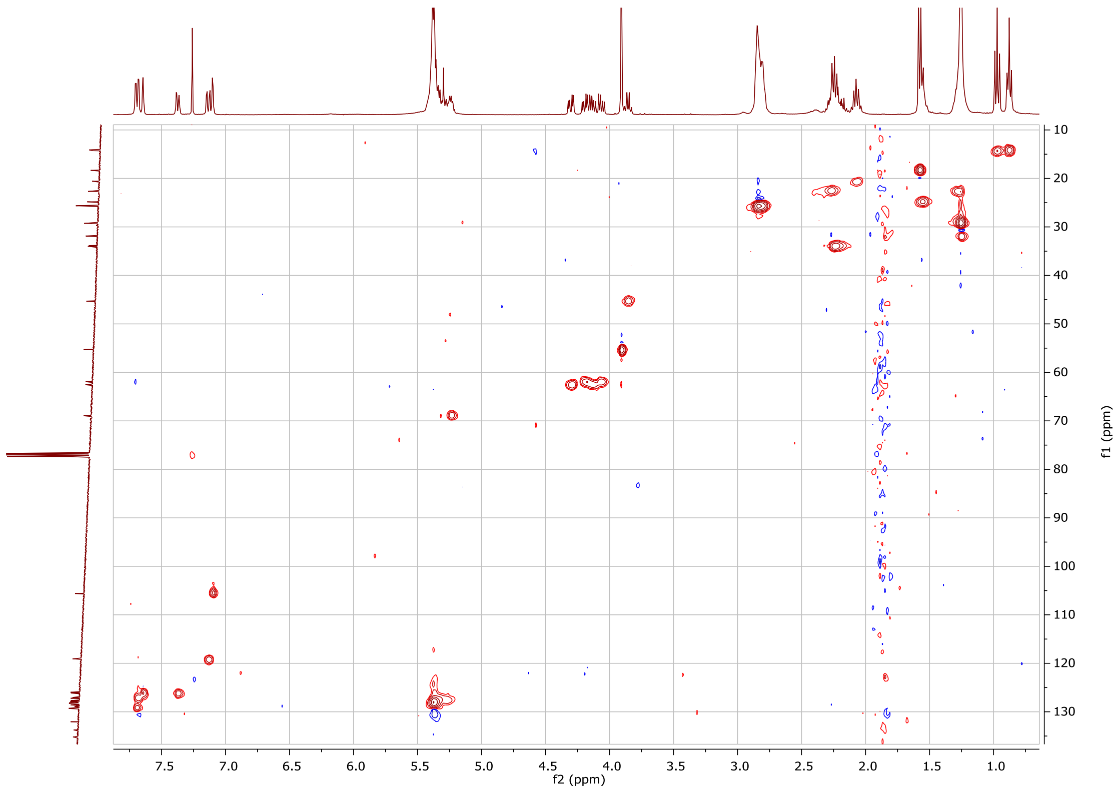

^{13}C-^{1}H HSQC of compound **(*S,S′*)-14c**
